# Gut microbiota, lipid metabolism, and PCOS: A Mendelian randomization and mediation analysis

**DOI:** 10.1097/MD.0000000000047177

**Published:** 2026-01-16

**Authors:** Wenyi Li, Junhong Gan, Junyao Jiao, Jianling Li, Lin Xu, Dongxiao Li, Yutao Geng, Mengqi Shen, Jing Liang, Hanmei Lin, Lu Zhong

**Affiliations:** a Guangxi University of Chinese Medicine, Guangxi International Zhuang Medicine Hospital, Nanning, Guangxi, China; b Graduate School, Guangxi University of Chinese Medicine, Guangxi International Zhuang Medicine Hospital, Nanning, Guangxi, China; c Graduate School, Guangxi University of Chinese Medicine, Nanning, Guangxi, China; d Faculty of Chinese Medicine Science, Guangxi University of Chinese Medicine, Nanning, Guangxi, China; e Ruikang Clinical Medical College, Guangxi University of Chinese Medicine, Nanning, Guangxi, China; f Department of Gynecology, The First Affiliated Hospital of Guangxi University of Chinese Medicine, Nanning, Guangxi, China.

**Keywords:** gut microbiota, lipid metabolism, mediation analysis, Mendelian randomization, polycystic ovary syndrome

## Abstract

Polycystic ovary syndrome (PCOS) is a common reproductive and metabolic disorder. Gut microbiota (GM) and lipid metabolism are increasingly implicated in its pathogenesis. We aimed to evaluate their causal roles and the potential mediating effect of lipid metabolites on PCOS. We conducted a 2-sample Mendelian randomization using GWAS summary statistics for 211 GM taxa, lipid metabolites, and PCOS cases from the FinnGen consortium. Instrumental variables were selected at genome-wide significance (*P* < 5 × 10⁻⁸, linkage disequilibrium *r*² < 0.001). Causal effects were estimated with inverse-variance weighted as the primary method, complemented by MR-Egger and weighted median. Mediation analysis quantified the indirect effect of lipids. Twenty-nine GM taxa showed significant causal associations with PCOS, including protective taxa (*Acetobacterales*, *Bifidobacterium longum*) and risk-enhancing taxa (*Proteus*, *Methanobacterium B*). Seventeen lipid metabolites were linked to PCOS, with phosphatidylcholines and triacylglycerols increasing risk, while sterol ester (SE [27:1/14:0]) and phosphatidylethanolamine (PE [O-16:1_20:4]) were protective. Mediation analysis indicated that taxa such as *Gordonibacter* exerted indirect effects on PCOS through phospholipid pathways, with mediation proportions up to 30%. This study provides evidence for a GM-lipid metabolism-PCOS axis. Dysbiosis and lipid disturbances jointly contribute to PCOS risk. Gut microbial and lipidomic signatures may serve as targets for early diagnosis and intervention. Further validation in diverse populations and experimental models is required.

## 1. Introduction

Polycystic ovary syndrome (PCOS) is the most common reproductive endocrine disorder among women of reproductive age, with a prevalence of 7% to 12%.^[1]^ Beyond its impact on reproductive function, PCOS also affects metabolic and psychological health, imposing significant burdens on women’s overall well-being. Clinically, PCOS is highly heterogeneous, with hallmark features including chronic anovulation, hyperandrogenism, and polycystic ovarian morphology as observed by ultrasound.^[2,3]^ Additionally, PCOS is frequently associated with psychological issues such as anxiety and depression.^[4]^

In addition to its reproductive and psychological implications, PCOS presents a sustained metabolic health risk. Due to disturbances in glucose metabolism, women with PCOS are more susceptible to developing type 2 diabetes,^[5]^ and they also face an elevated risk of cardiovascular diseases.^[6]^ Notably, the global prevalence of PCOS has been rising in recent years. From 1990 to 2019, the age-standardized incidence rate of PCOS increased by 30.4%, reaching 1677.8 cases per 1,00,000 females worldwide.^[7]^ The escalating health burden of PCOS not only affects individuals but also imposes a substantial economic and societal burden. In the United States alone, medical expenditures related to PCOS were estimated to reach as high as US $8 billion in 2020.^[8]^ PCOS has thus emerged as a pressing global public health concern. Elucidating its pathogenesis is crucial for developing more effective early interventions and personalized treatment strategies to mitigate the long-term health burden associated with PCOS.

Current research indicates that women with PCOS exhibit significant gut microbiota (GM) dysbiosis, characterized by a reduction in beneficial microbes, an increase in opportunistic pathogens, and decreased microbial diversity. These alterations may exacerbate metabolic dysfunction via several mechanisms, including impaired short-chain fatty acid (SCFA) production, altered bile acid metabolism, and induction of systemic inflammation.^[9,10]^ Moreover, lipid metabolic dysfunction is a key pathological feature of PCOS, manifesting as altered levels of free fatty acids, phospholipids, cholesterol, and triglycerides. These changes, especially in the context of insulin resistance and inflammation, may contribute to the development and progression of PCOS. There is increasing evidence of close interplay between GM and lipid metabolism, forming what is referred to as the “gut microbiota–lipid metabolism axis.” However, its specific role and causal relationship in PCOS remain unclear.^[11,12]^ Conventional observational studies are limited in their ability to distinguish causation from correlation. Mendelian randomization (MR), which uses genetic variants as instrumental variables (IVs), enables more accurate inference of causal relationships between exposures and outcomes, thereby reducing the risk of reverse causation bias.^[13]^

## 2. Materials and methods

### 2.1. Study design

This study utilized a 2-sample MR approach to explore the causal relationships between GM, lipid metabolism, and PCOS, and to assess the mediating role of lipid metabolism. The inverse-variance weighted (IVW) method was employed as the primary statistical approach to evaluate the causal effect of exposures on PCOS, with Mendelian Randomization Egger regression (MR-Egger) conducted as a complementary sensitivity analysis.

In the initial analysis, GM features were considered as exposure variables, and single nucleotide polymorphisms (SNPs) significantly associated with these microbial traits were selected as IVs, while PCOS served as the outcome variable. Subsequently, gut microbial traits that were identified in the first step as significantly associated with PCOS were further used as exposures to assess their causal effects on lipid metabolism traits. This helped to identify lipid metabolites most closely associated with PCOS, which were then included in the mediation analysis. The causal relationships between these lipid traits and PCOS were also examined, followed by the estimation of their mediating effects in the relationship between GM and PCOS. MR analysis must satisfy the following 3 core assumptions^[14]^: the selected SNPs are strongly associated with the exposure variable (GM or lipid metabolism); the selected SNPs are not affected by potential confounders – that is, they are independent of confounding factors between the exposure and outcome; and the SNPs influence the outcome variable (PCOS) exclusively through the exposure variable, without any direct effects.

### 2.2. Data sources

This study integrated data from multiple large-scale cohort studies to systematically investigate the causal relationships between GM, lipid metabolism, and PCOS. PCOS data were obtained from the FinnGen consortium. Specifically, the R9_E4_PCOS cohort included 3,92,423 female participants, among whom 1424 were diagnosed with PCOS.^[15]^ GM data were derived from a population-based study in Finland involving 5959 participants. This study conducted a genome-wide association analysis to assess the associations between host genetic variation and GM abundance. It integrated human genotyping data and fecal metagenomic sequencing, and evaluated the potential links among host genetics, dietary habits, microbial abundance, and disease risk based on dietary and health records.^[16]^ Lipid metabolism data were sourced from the study by Ottensmann et al, which systematically analyzed 179 lipid metabolites spanning 13 lipid categories.^[17]^ These included 4 major lipid classes: glycerolipids, glycerophospholipids, sphingolipids, and sterols. All study participants were of European ancestry and provided comprehensive metabolic profiling data, ensuring consistency across datasets. All data used in this analysis are de-identified and publicly accessible; as such, ethical approval and informed consent were not necessary.

### 2.3. SNPs selection

In this study, SNPs that were genome-wide significantly associated with the exposure (*P* < 5 × 10⁻⁸) were selected. To reduce the influence of linkage disequilibrium on IVs, linkage disequilibrium clumping was performed using the following parameters: *r*² = 0.001 and a window size of 5000 kb, ensuring the independence of selected IVs. To meet the second and third core assumptions of MR, we used the R package to query the PhenoScanner database to screen for potential confounders. SNPs associated with known confounding factors or directly associated with the outcome were excluded to ensure that the selected IVs were valid and not pleiotropic.^[18,19]^

During the SNP filtering process, we first removed SNPs with significant heterogeneity as identified by heterogeneity tests. The remaining SNPs that were significantly associated with the exposure traits (GM or lipid metabolism) were retained as valid IVs. Subsequently, the *F*-statistic was calculated to assess the strength of each IV. SNPs with *F* > 10 were considered strong instruments suitable for causal inference, while SNPs with *F* < 10 were subject to further scrutiny or exclusion to minimize the influence of weak instruments. The *F*-statistic was calculated using the following formula: *F* = [*R*² × (N − *k* − 1)]/[*k* × (1 − *R*²)], where *R*² denotes the coefficient of determination (explained variance), *k* is the number of predictors (including the intercept), and N is the sample size.^[20]^

### 2.4. Mendelian randomization analysis

In this study, the IVW method was used as the primary approach for MR analysis. The IVW method assigns weights to each estimate based on the inverse of their variance (or standard error), giving more weight to estimates with higher precision and thereby improving the overall accuracy of the causal effect estimation.

In addition, MR-Egger regression was applied as a supplementary analysis. Unlike the IVW method, MR-Egger introduces an intercept term to detect and adjust for potential directional pleiotropy, which can bias the causal estimates. This method allows for pleiotropy under the assumption that the magnitude of the pleiotropic effects is independent of the strength of the instruments assumption, thus providing a robustness check for the MR findings.^[21]^

### 2.5. Mediation analysis

**Figure 1.**
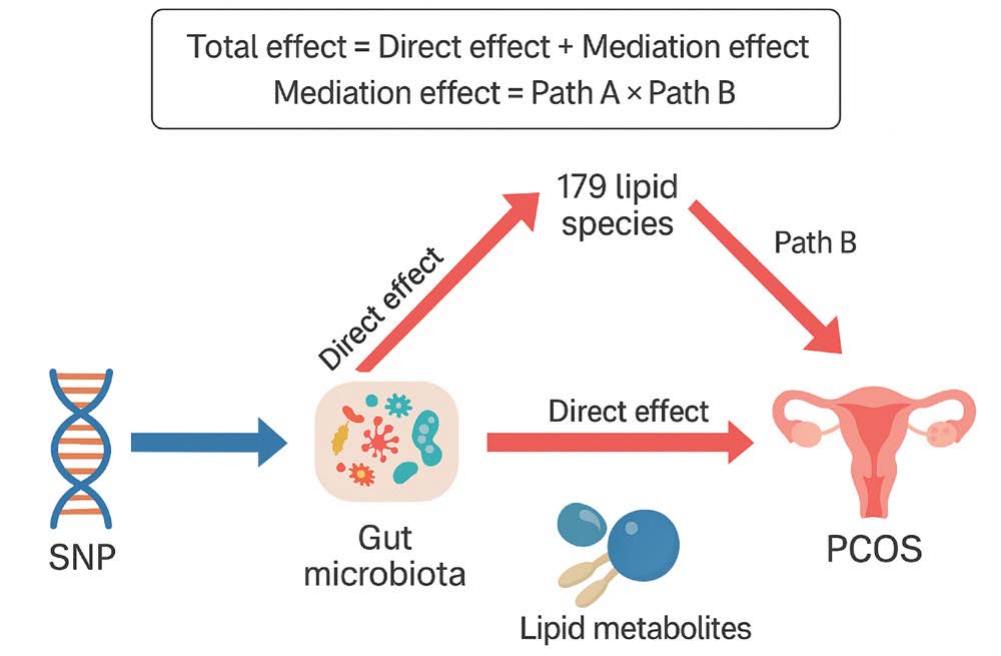
Study design of the mediating role of the gut microbiota–lipid metabolism axis in PCOS. This figure illustrates the study framework using Mendelian randomization and mediation analysis to evaluate the potential mediating effect of lipid metabolism in the relationship between gut microbiota and PCOS. Path A represents the causal effect of gut microbiota on lipid metabolism, path B represents the effect of lipid metabolism on PCOS, and the mediation effect is calculated as path A × path B. PCOS = polycystic ovary syndrome, SNP = single nucleotide polymorphism..

Building on the MR findings, a 2-sample MR design was further employed to perform mediation analysis, aiming to explore the mediating role of lipid metabolism in the relationship between GM and PCOS. Within the framework of causal inference, the total effect (total β) can be decomposed into a direct effect (direct β) and an indirect effect (indirect β), expressed mathematically as: total β = direct β + indirect β. Specifically, the total effect of GM on PCOS includes: direct effect (path A): the direct influence of GM on PCOS; indirect effect (path B): the effect of GM on PCOS mediated through lipid metabolism. The indirect effect is calculated as: indirect β = path A × path B (see Fig. 1).^[22]^

### 2.6. Sensitivity analysis

To ensure the robustness and validity of the IVs, this study employed multiple sensitivity analysis methods, including MR-Egger regression, Cochran’s *Q* test, and leave-one-out analysis. MR-Egger regression was used to evaluate horizontal pleiotropy. If the intercept of the MR-Egger regression is close to 0, it suggests that the MR-Egger estimate aligns closely with the IVW result, indicating minimal pleiotropic bias. A significant deviation of the intercept from 0 may imply the presence of horizontal pleiotropy among the SNPs. Cochran’s *Q* test was applied to detect heterogeneity among the SNPs. A significant *Q* statistic indicates that the SNPs may have heterogeneous effects, with varying directions or magnitudes of influence on the outcome. Leave-one-out analysis involves sequentially removing 1 SNP at a time and recalculating the overall effect estimate using the remaining SNPs. This method helps identify whether any single SNP disproportionately influences the causal estimate. By integrating these sensitivity analyses, the study enhances the robustness of the MR findings and strengthens the reliability of the causal inferences drawn between GM, lipid metabolism, and PCOS.^[23-25]^

## 3. Results

### 3.1. MR results

#### 3.1.1. Mendelian randomization analysis of gut microbiota and PCOS

A total of 530 SNPs were selected as IVs for GM traits in the MR analysis. All SNPs had *F*-statistics > 10, with an average *F*-value of 21.42, indicating sufficient instrument strength and minimizing the risk of weak instrument bias. All SNPs passed harmonization procedures, including palindromic and ambiguous variant filtering, and were retained for the final analysis (Table S1, Supplemental Digital Content, https://links.lww.com/MD/R163).

**Figure 2.**
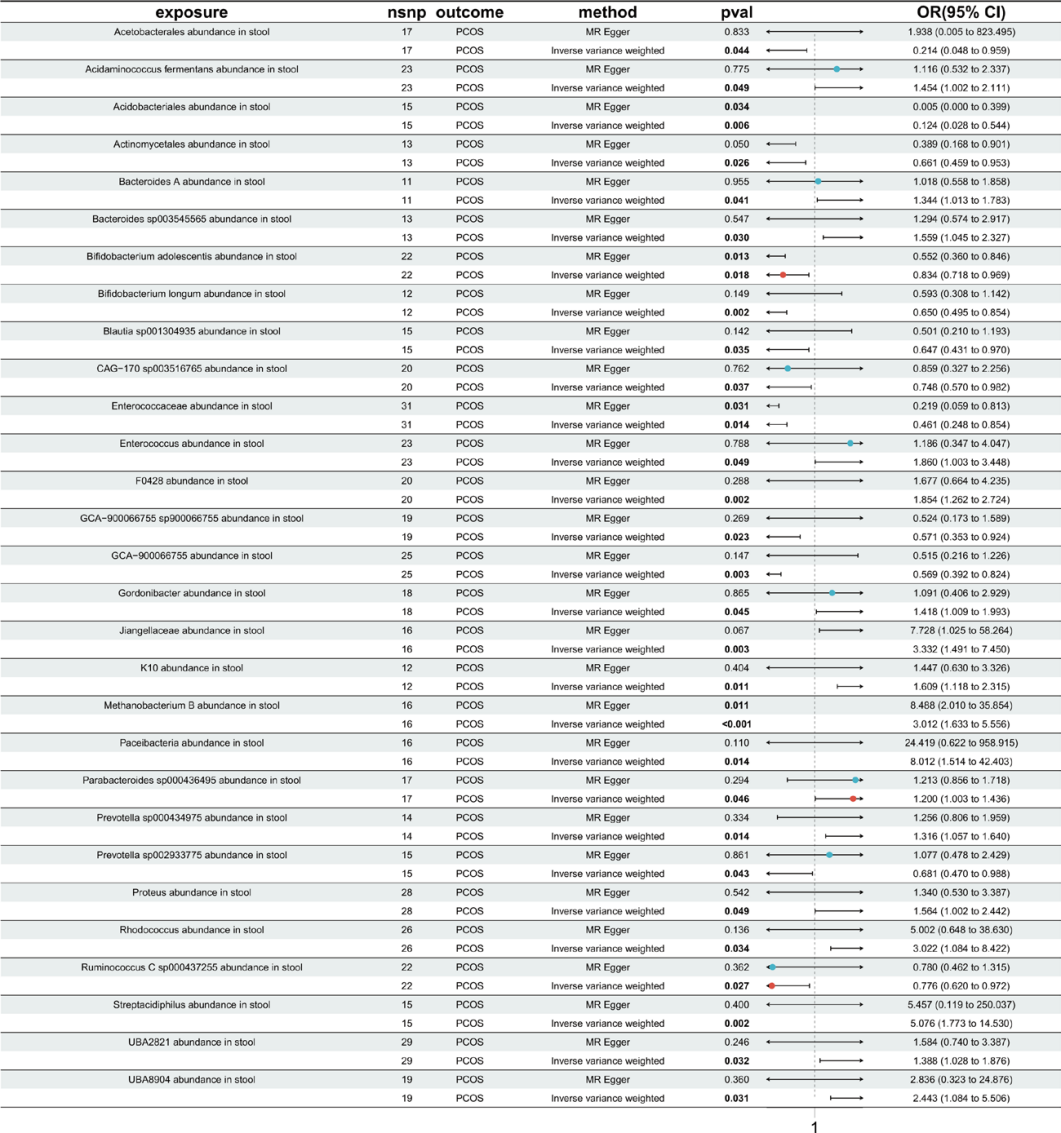
Mendelian randomization results between gut microbiota and PCOS. This figure presents the results of Mendelian randomization analyses assessing the causal relationships between various gut microbiota taxa (exposures) and PCOS (outcome). Two analytical methods – MR-Egger and inverse-variance weighted – were used. The figure reports the odds ratios with corresponding 95% confidence intervals and significance *P*-values for each association. MR = Mendelian randomization, PCOS = polycystic ovary syndrome.

We identified 29 GM taxa significantly associated with PCOS, of which 12 taxa showed negative associations and 17 taxa showed positive associations (Fig. 2). Among the negatively associated taxa, notable examples included *Acetobacterales* (odds ratio [OR] = 0.214; 95% CI: 0.048–0.959; *P* = .044) and *Acidobacteriales* (OR = 0.124; 95% CI: 0.028–0.544; *P* = .006), suggesting potential protective roles in maintaining host metabolic homeostasis.

In contrast, several taxa were positively associated with PCOS, including *Acidaminococcus fermentans* (OR = 1.454; 95% CI: 1.002–2.111; *P* = .049), *Proteus* (OR = 1.564; 95% CI: 1.002–2.442; *P* = .049), and K10 (OR = 1.609; 95% CI: 1.118–2.315; *P* = .011), indicating possible contributions to PCOS pathogenesis.

To assess potential reverse causality, we conducted reverse MR analyses treating PCOS as the exposure and GM taxa as outcomes. Across all tested taxa, IVW estimates revealed no significant associations (all *P* > .19), with effect sizes close to null, suggesting limited evidence that PCOS causally alters GM composition (Table S2, Supplemental Digital Content, https://links.lww.com/MD/R163).

#### 3.1.2. Mendelian randomization analysis of lipid metabolites and PCOS

**Figure 3.**
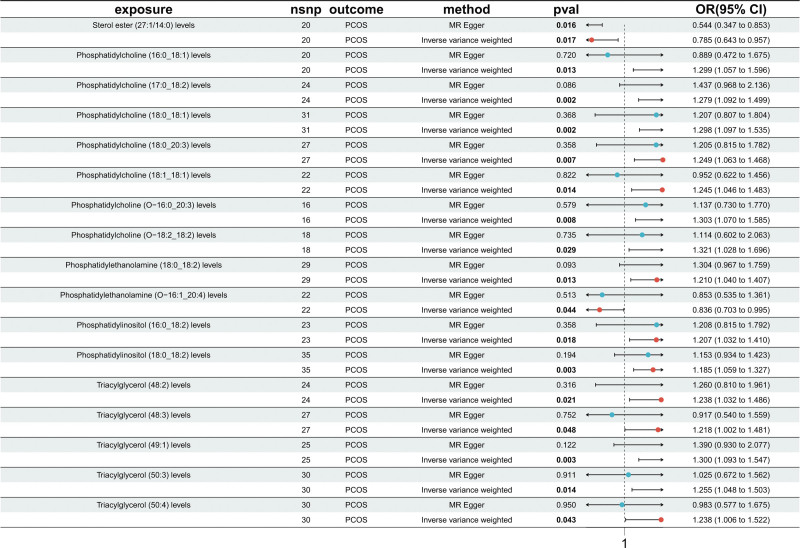
Mendelian randomization results between lipid metabolites and PCOS. This figure presents the causal association analysis between specific lipid metabolites (such as PC, PI, and TG) and PCOS. The effects of each lipid metabolite (exposure) on PCOS (outcome) were evaluated using both MR-Egger and inverse-variance weighted methods. The figure reports *P*-values and odds ratios with 95% confidence intervals. MR = Mendelian randomization, PC = phosphatidylcholine, PI = phosphatidylinositol, PCOS = polycystic ovary syndrome, TG = triacylglycerol.

A total of 431 SNPs were included as IVs in the MR analysis. All SNPs had *F*-statistics >10, with an average *F*-value of 24.14, indicating good instrument strength and ruling out potential bias from weak instruments (Table S3, Supplemental Digital Content, https://links.lww.com/MD/R163). No palindromic or ambiguous SNPs were identified, and all passed harmonization checks, ensuring comparability and analytical validity. This study identified 17 lipid metabolites significantly associated with PCOS, of which 2 were negatively associated and 15 were positively associated. Among the negatively associated metabolites, higher levels of sterol ester (SE; 27:1/14:0; OR = 0.785; 95% CI: 0.643–0.957; *P* = .017) and phosphatidylethanolamine (PE; O-16:1_20:4; OR = 0.836; 95% CI: 0.703–0.995; *P* = .044) were linked to a reduced risk of PCOS, suggesting potential protective roles. In contrast, several subtypes of phosphatidylcholine (PC) showed positive associations with PCOS, including PC (16:0_18:1; OR = 1.299; 95% CI: 1.057–1.596; *P* = .013), PC (17:0_18:2; OR = 1.279; 95% CI: 1.092–1.499; *P* = .002), and PC (18:0_18:1; OR = 1.298; 95% CI: 1.097–1.535; *P *= .002). Similarly, multiple TG subtypes also exhibited significant positive associations with PCOS, such as TG (48:2; OR = 1.238; 95% CI: 1.032–1.486; *P* = .021), TG (48:3; OR = 1.218; 95% CI: 1.002–1.481; *P* = .048), and TG (49:1; OR = 1.300; 95% CI: 1.093–1.547; *P* = .003; Fig. 3). Among all methods and metabolites, only the weighted median estimate for GCST90277281 indicated a nominally significant association (OR = 0.93, 95% CI: 0.87–0.99, *P* = .031), suggesting a possible weak inverse effect. However, the majority of associations were non-significant, with wide confidence intervals (CIs) including the null, indicating limited evidence for reverse causality from PCOS to lipid metabolism dysregulation (Table S4, Supplemental Digital Content, https://links.lww.com/MD/R163).

#### 3.1.3. Mediation screening analysis results

**Figure 4.**
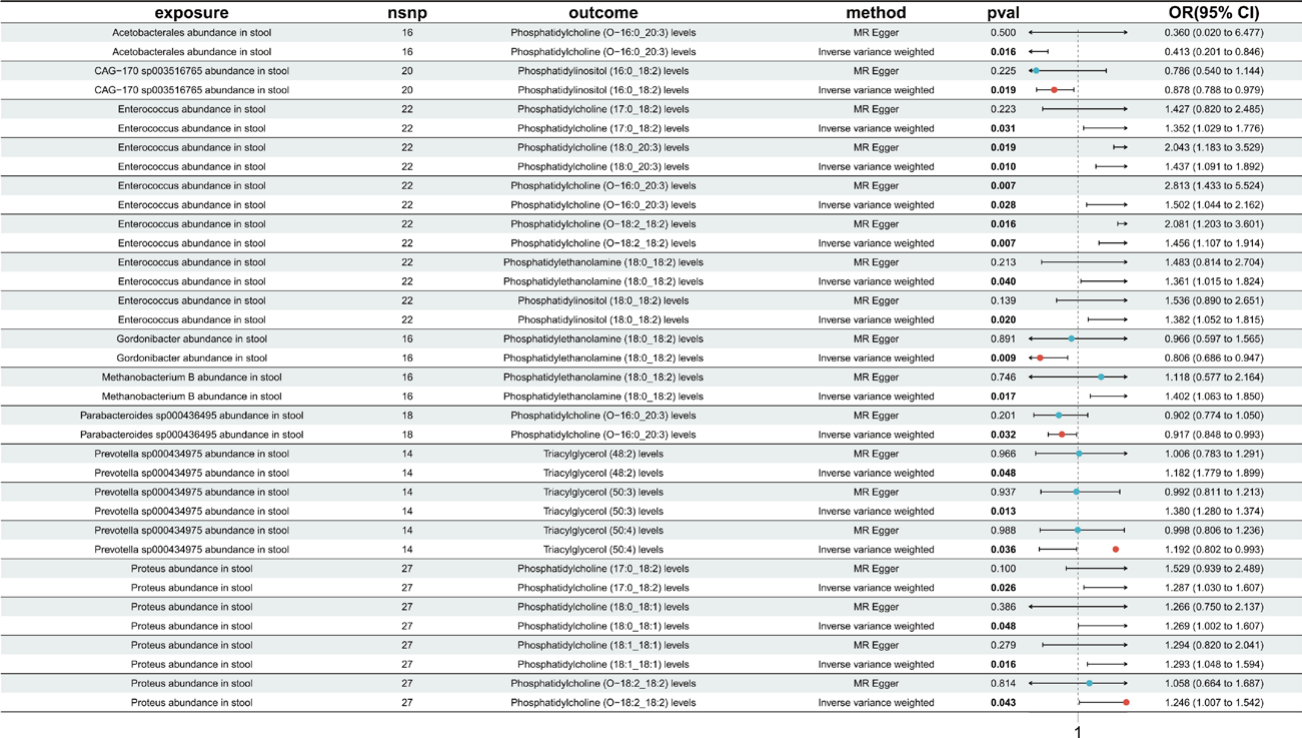
MR results between gut microbiota and lipid metabolites. This figure shows the causal effects of gut microbiota (exposures) on specific lipid metabolites (outcomes), assessed using MR-Egger and IVW methods. It reports the number of SNPs, *P*-values, and ORs (95% CI) for each analysis. CI = confidence interval, IVW = inverse-variance weighted, MR = Mendelian randomization, OR = odds ratio, SNP = single nucleotide polymorphism.

To further investigate the causal influence of lipid metabolism on GM, a 2-sample MR analysis was conducted using lipid metabolites as exposure variables and GM as outcome variables. A total of 375 SNPs were selected as IVs for lipid metabolites. All SNPs passed screening for palindromic and ambiguous sites as well as harmonization checks, meeting the criteria for inclusion. The *F*-statistics for all instruments exceeded 10, with an average *F*-value of ~20.9, indicating strong instrument strength and minimizing the risk of bias from weak instruments (Table S5, Supplemental Digital Content, https://links.lww.com/MD/R163). A total of 15 significant causal associations were identified. Heterogeneity and horizontal pleiotropy analyses revealed no significant evidence of either, supporting the robustness and reliability of the MR models (Fig. 4).

#### 3.1.4. Proportion of mediation effects

This study quantified the mediation effects of GM on PCOS by calculating the total effect, direct effect, and indirect effect. *Gordonibacter* abundance in stool influenced PC (O-18:2_18:2) levels, with a mediation effect accounting for 29.92% of the total effect. *Prevotella sp000434975* abundance mediated 23.45% of its effect on PCOS through PE (18:0_18:2; *P *= .197). Additionally, *Enterococcus* abundance affected multiple lipid metabolites, including PC (O-16:0_20:3; 17.35% mediation) and PC (18:0_20:3; 13.01% mediation). *Proteus* abundance mediated 12.29% of its effect on PCOS through phosphatidylinositol (18:0_18:2; Fig. 5).

**Figure 5.**
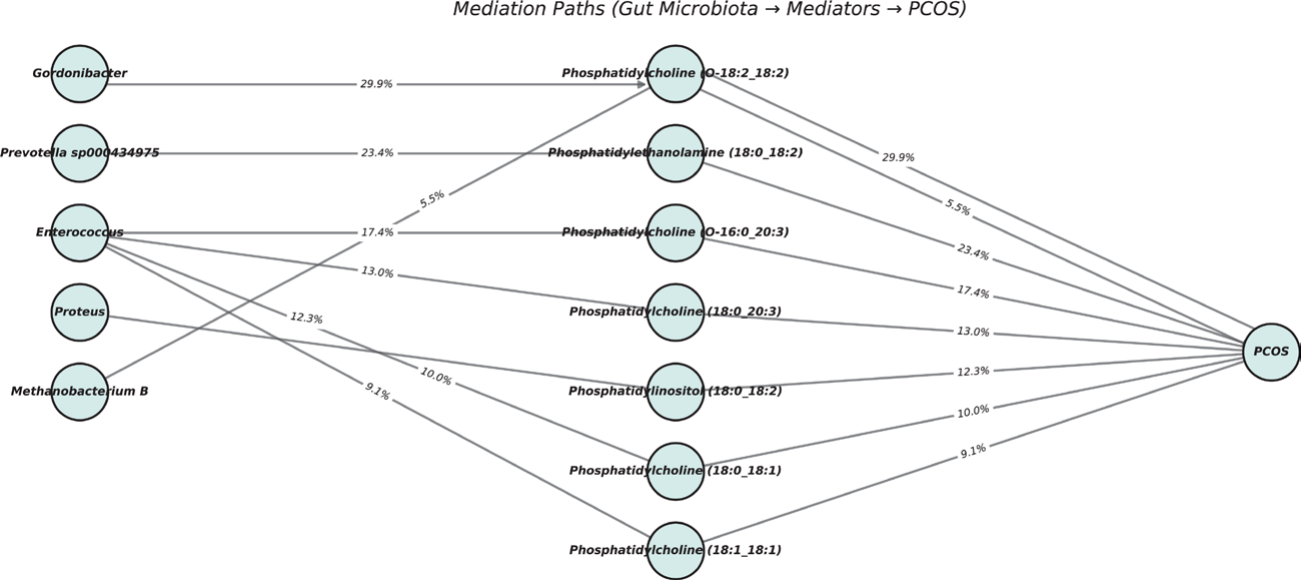
Representative causal mediation network from gut microbiota to PCOS via lipid metabolites. Nodes represent microbial taxa, lipid mediators, and outcome, arranged in directional layers. PCOS = polycystic ovary syndrome.

### 3.2. Sensitivity analysis results

In the analysis between GM and PCOS most taxa showed MR-Egger, Cochran’s *Q*, and MR-PRESSO *P*-values above .05, indicating no substantial evidence of pleiotropy or heterogeneity and suggesting relatively stable causal inference results (Table S6 and PDF 1, Supplemental Digital Content, https://links.lww.com/MD/R163; https://links.lww.com/MD/R164). However, for *Rhodococcus*, the *P*-values from the *Q* test and MR-PRESSO were close to significance, indicating potential heterogeneity interference; thus, its results should be interpreted with caution alongside the main effect estimates.

In the analysis of lipid metabolites and PCOS, sensitivity indicators were generally acceptable. Nevertheless, PE (18:0_18:2) showed low *P*-values in both Cochran’s *Q* and MR-PRESSO tests, suggesting possible heterogeneity or invalid instruments. Similarly, PC (O-18:2_18:2) had a marginally low *Q* test *P*-value (Table S7 and PDF 2, Supplemental Digital Content, https://links.lww.com/MD/R163; https://links.lww.com/MD/R164). In the analysis between GM and lipid metabolites (Table S8, Supplemental Digital Content, https://links.lww.com/MD/R163), overall sensitivity results did not indicate major abnormalities. Notably, *Enterococcus* abundance and PC (O-16:0_20:3) yielded *P*-values below .05 across all 3 sensitivity tests, which may reflect potential pleiotropy or instrument bias and should be further evaluated in conjunction with main results. Additionally, the *Q* test result for *Prevotella sp000434975* and TG (48:2) was borderline significant and merits attention (Table S8 and PDF 3, Supplemental Digital Content, https://links.lww.com/MD/R163; https://links.lww.com/MD/R164).

## 4. Discussion

This study systematically explored the causal relationships between GM, lipid metabolites, and PCOS using MR analysis. In addition, mediation analysis was conducted to further elucidate how GM may influence PCOS development through lipid metabolism. A total of 29 gut microbial taxa were identified as significantly associated with PCOS, with 12 taxa showing negative associations and 17 showing positive associations. Meanwhile, 17 lipid metabolites were found to have a causal relationship with PCOS. Among them, SE (27:1/14:0) and PE (O-16:1_20:4) were associated with a reduced risk of PCOS, suggesting protective effects, whereas several PC and TG subtypes were positively associated with increased PCOS risk. The mediation analysis further supported the existence of a microbiota–lipid axis in PCOS pathophysiology, providing more robust causal evidence for the metabolic disturbances and disease mechanisms underlying PCOS. These findings extend previous observational studies by providing causal evidence, highlighting the importance of the GM-lipid axis as a novel etiological pathway in PCOS.

The impact of GM on PCOS has attracted increasing attention, as patients with PCOS exhibit significant alterations in GM composition, including a reduction in beneficial microbes, enrichment of potentially pathogenic bacteria, and decreased microbial diversity. These changes may exacerbate metabolic disturbances and endocrine imbalance in PCOS through mechanisms involving bile acid metabolism, SCFA production, and estrogen homeostasis regulation.^[26]^ In the present study, *Enterococcus*, *Bacteroides*, and *Parabacteroides* were found to be positively associated with PCOS. *Enterococcus* has been reported to secrete a metabolic product, GeIE, which degrades GLP-1 – a key regulator of insulin secretion. Impairment of GLP-1 function may further aggravate insulin resistance and metabolic dysfunction in PCOS patients.^[27]^ Previous studies have shown that *Bacteroides* is enriched in PCOS patients and is associated with reduced levels of glycodeoxycholic acid. *Bacteroides* may influence insulin signaling and ovarian function by modulating bile acid metabolism and suppressing IL-22 production.^[27,28]^ Moreover, Parabacteroides has been reported to correlate positively with body mass index, testosterone, luteinizing hormone, and anti-Müllerian hormone levels. It may induce chronic inflammation via the LPS–TLR4/NF-κB signaling pathway, thereby impairing insulin signaling and ovarian function.^[29]^ Additionally, Parabacteroides may contribute to PCOS related metabolic abnormalities by regulating bile acid metabolism through the FXR/TGR5 signaling pathway. In contrast, this study found that *Bifidobacterium* longum and *Bifidobacterium adolescentis* were negatively associated with PCOS. Previous studies have shown that *Bifidobacterium*, as a probiotic genus, may improve metabolic abnormalities in PCOS by promoting SCFA production, optimizing bile acid metabolism, maintaining intestinal barrier integrity, reducing systemic inflammation, and influencing estrogen metabolism. These findings are consistent with our results and suggest that specific probiotics may have potential therapeutic value in PCOS prevention and treatment.^[30-32]^ Lipid metabolism abnormalities play a central role in PCOS. This study identified several PCs, triacylglycerols, phosphatidylethanolamines, and SEs that were significantly associated with PCOS.^[32]^ PC is crucial in regulating hepatic TG levels and cholesterol metabolism. We found that PC subtypes such as PC (16:0_18:1) and PC (18:0_18:1) were positively associated with PCOS, suggesting that abnormal PC accumulation may contribute to disordered lipid metabolism. Studies have shown that PC, in conjunction with S-adenosylmethionine, can regulate phosphatidylethanolamine *N*-methyltransferase, further affecting cholesterol and TG levels.^[33]^ TG levels are significantly elevated in the follicular fluid of PCOS patients. Although TG serves as an essential energy source for oocyte maturation, excessive accumulation may impair oocyte development, reduce follicular maturation rates, and ultimately affect reproductive function.^[34]^ Moreover, elevated TG levels are negatively associated with high-quality embryo formation, underscoring its critical role in PCOS pathophysiology.^[35]^ Research has also indicated that PE levels are significantly reduced in PCOS patients and are positively associated with the free androgen index, suggesting a role for PE in steroid hormone synthesis and secretion.^[36,37]^ Interestingly, different PE subtypes may exert opposite effects. In this study, elevated PE (18:0_18:2) levels were positively associated with PCOS risk, whereas increased PE (O-16:1_20:4) levels appeared to have a protective effect, possibly due to the differing physiological roles of their constituent fatty acids.

SE may exert protective effects in PCOS. We found that elevated levels of SE (27:1/14:0) were associated with a reduced risk of PCOS. Prior evidence suggests that SE may influence PCOS development by regulating cholesterol homeostasis, improving lipid metabolism, and reducing inflammatory responses. Furthermore, phytosterols and omega-3 fatty acids have been shown to improve lipid metabolism and lower inflammation, supporting the protective role of SE.^[38,39]^

The role of the GM-lipid axis in PCOS was further validated by mediation analysis, demonstrating that specific gut microbes may influence PCOS development via lipid metabolism regulation. We found that Gordonibacter abundance significantly affected PC (O-18:2_18:2) levels, with a mediation effect accounting for 29.92% of its total effect on PCOS, suggesting a regulatory role via PC metabolism in ovarian function and systemic lipid balance.^[40]^ *Prevotella sp000434975* exhibited a significant mediation effect (23.45%) through PE (18:0_18:2), implying potential involvement in energy homeostasis and follicular microenvironment modulation.^[41]^ Moreover, *Enterococcus* abundance strongly influenced several PC subtypes, such as PC (O-16:0_20:3) and PC (18:0_20:3), with mediation effects of 17.35% and 13.01%, respectively, indicating widespread regulation of lipid metabolism and exacerbation of PCOS-related metabolic disturbances. Proteus abundance was associated with Phosphatidylinositol (18:0_18:2) levels (12.29% mediation effect), suggesting a potential role in promoting inflammation and insulin resistance by altering saturated fatty acid ratios.^[42]^

## 5. Limitations

This study has several limitations that warrant consideration. The GWAS datasets were predominantly derived from European ancestry populations, which may restrict the generalizability of our findings. Because GM composition and lipid metabolism are shaped by genetic background, diet, and environmental exposures, replication in multi-ethnic cohorts will be essential to confirm these associations.

Another important limitation concerns the mediation analysis. The statistical power may have been insufficient to detect subtle indirect effects, which could explain why only a subset of GM-lipid pairs demonstrated significant mediation. Larger GWAS resources and more refined analytic approaches are needed to capture weaker yet biologically meaningful pathways.

A further consideration is that although MR provides robust evidence for causality, it does not elucidate the precise biological mechanisms linking gut microbes, lipid metabolites, and PCOS. Whether these effects are driven by microbial metabolites, immune modulation, or endocrine regulation remains unresolved, and experimental validation is necessary to clarify these mechanisms.

It is also noteworthy that all MR analyses depend on the validity of IV assumptions. Despite extensive sensitivity testing, residual horizontal pleiotropy cannot be fully excluded. Moreover, given the bidirectional nature of host–microbiome interactions, it remains difficult to determine whether certain microbial changes are drivers or consequences of PCOS.

## 6. Conclusion

This study provides causal evidence that both GM dysbiosis and lipid metabolic disturbances contribute to the development of PCOS. We identified specific microbial taxa and lipid metabolites with protective or risk-enhancing effects, and mediation analysis further highlighted a GM-lipid axis in PCOS pathogenesis. These findings suggest that targeting gut microbial signatures and lipidomic profiles may offer promising avenues for early diagnosis and therapeutic strategies. However, the generalizability of our results may be limited to populations of European ancestry, and MR cannot fully elucidate the precise biological mechanisms. Future studies involving diverse cohorts, experimental validation, and clinical interventions are needed to confirm these causal pathways and translate them into clinical applications.

## Acknowledgments

We thank the participants and investigators of the contributing cohorts and consortia (FinnGen, and the studies providing gut microbiota and lipid metabolite GWAS) for making their data publicly available.

## Author contributions

**Conceptualization:** Wenyi Li, Junhong Gan, Junyao Jiao.

**Data curation:** Wenyi Li, Jianling Li, Lu Zhong.

**Formal analysis:** Junhong Gan, Junyao Jiao, Dongxiao Li.

**Funding acquisition:** Lu Zhong.

**Investigation:** Jianling Li, Dongxiao Li, Jing Liang.

**Methodology:** Wenyi Li, Junhong Gan, Yutao Geng, Mengqi Shen.

**Project administration:** Lin Xu, Hanmei Lin.

**Resources:** Lin Xu, Hanmei Lin.

**Software:** Junhong Gan, Junyao Jiao, Dongxiao Li.

**Supervision:** Lin Xu, Hanmei Lin.

**Validation:** Jing Liang, Lu Zhong.

**Visualization:** Wenyi Li, Junhong Gan.

**Writing – original draft:** Wenyi Li.

**Writing – review & editing:** Wenyi Li, Junhong Gan, Lu Zhong.

## Supplementary Material




