# Supplementary material for: Gut microbiota, lipid metabolism, and PCOS: A Mendelian randomization and mediation analysis
Source: Medicine (Baltimore). 2026 Jan 16;105(3):e47177. doi: 10.1097/MD.0000000000047177 (PMC12826245; doi:10.1097/MD.0000000000047177)
Supplement: Supplementary file 2 [file medi-105-e47177-s002.pdf]

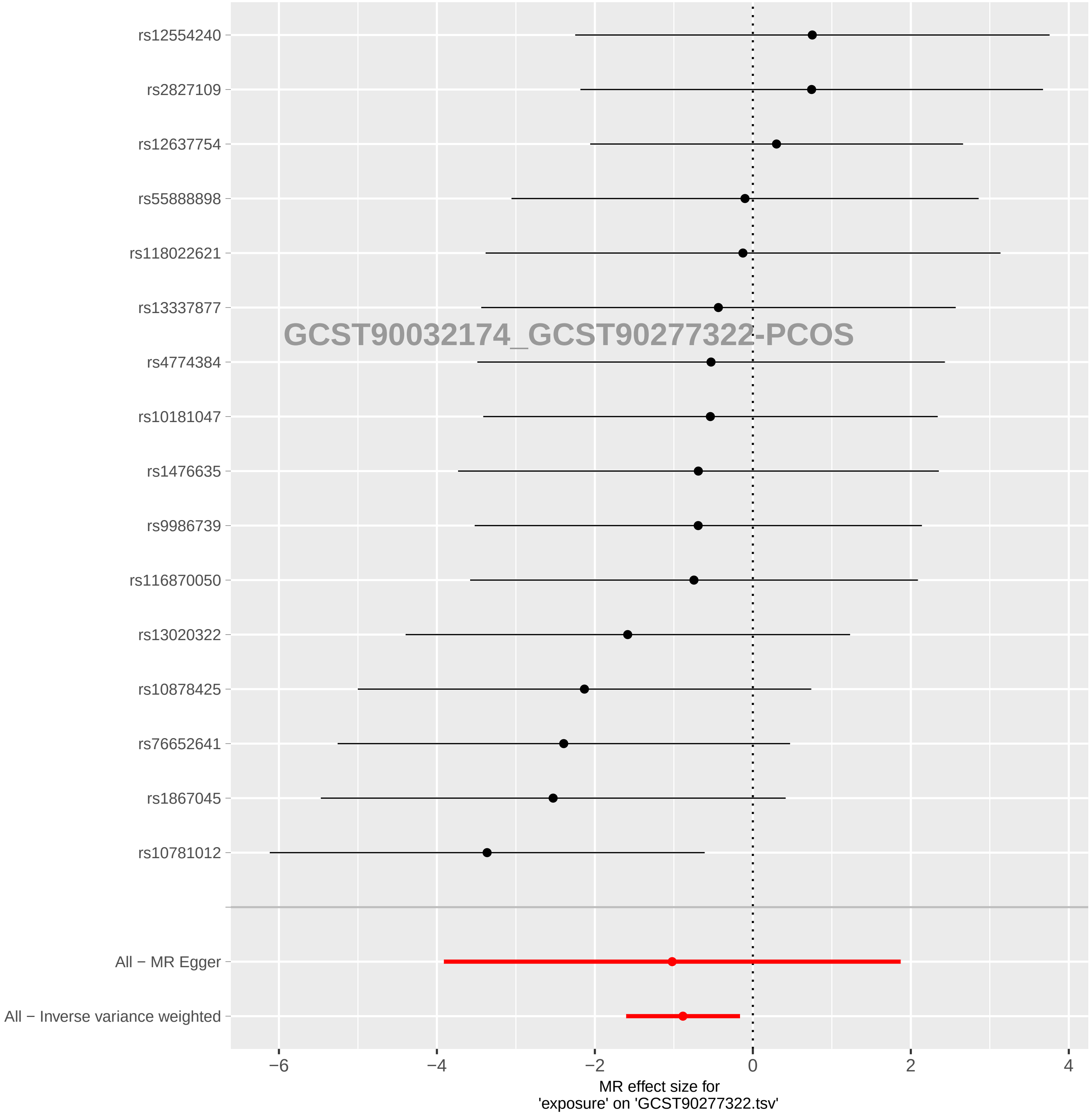

rs12554240
rs2827109
rs12637754
rs55888898
rs118022621
rs13337877
GCST90032174_GCST90277322-PCOS
rs4774384
rs10181047
rs1476635
rs9986739
rs116870050
rs13020322
rs10878425
rs76652641
rs1867045
rs10781012
All − MR Egger
All − Inverse variance weighted
−6
−4
−2
0
2
4
MR effect size for
'exposure' on 'GCST90277322.tsv'

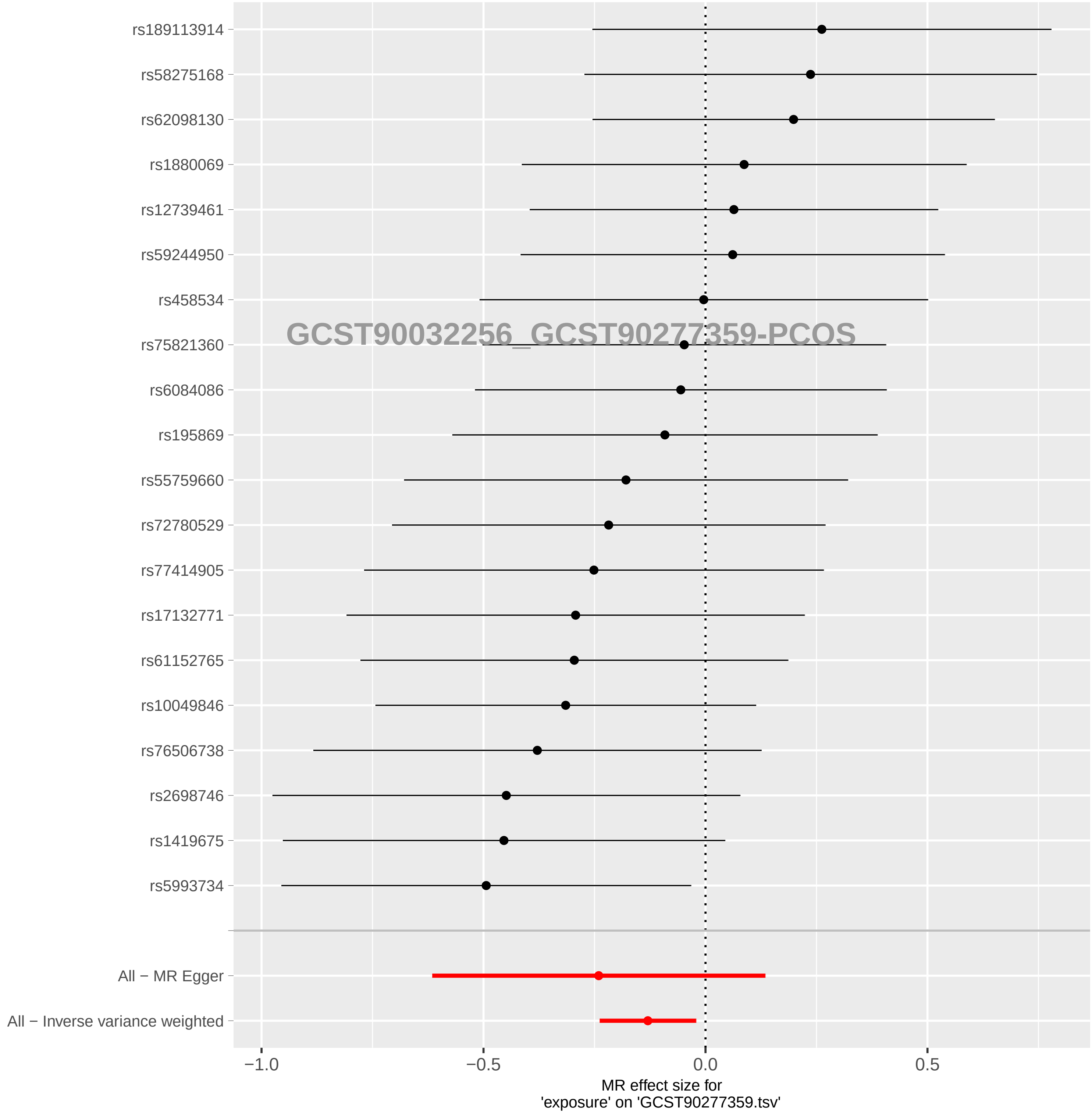
GCST90032256_GCST90277359-PCOS
rs189113914
rs58275168
rs62098130
rs1880069
rs12739461
rs59244950
rs458534
rs75821360
rs6084086
rs195869
rs55759660
rs72780529
rs77414905
rs17132771
rs61152765
rs10049846
rs76506738
rs2698746
rs1419675
rs5993734
All − MR Egger
All − Inverse variance weighted
−1.0
−0.5
0.0
0.5
MR effect size for
'exposure' on 'GCST90277359.tsv'

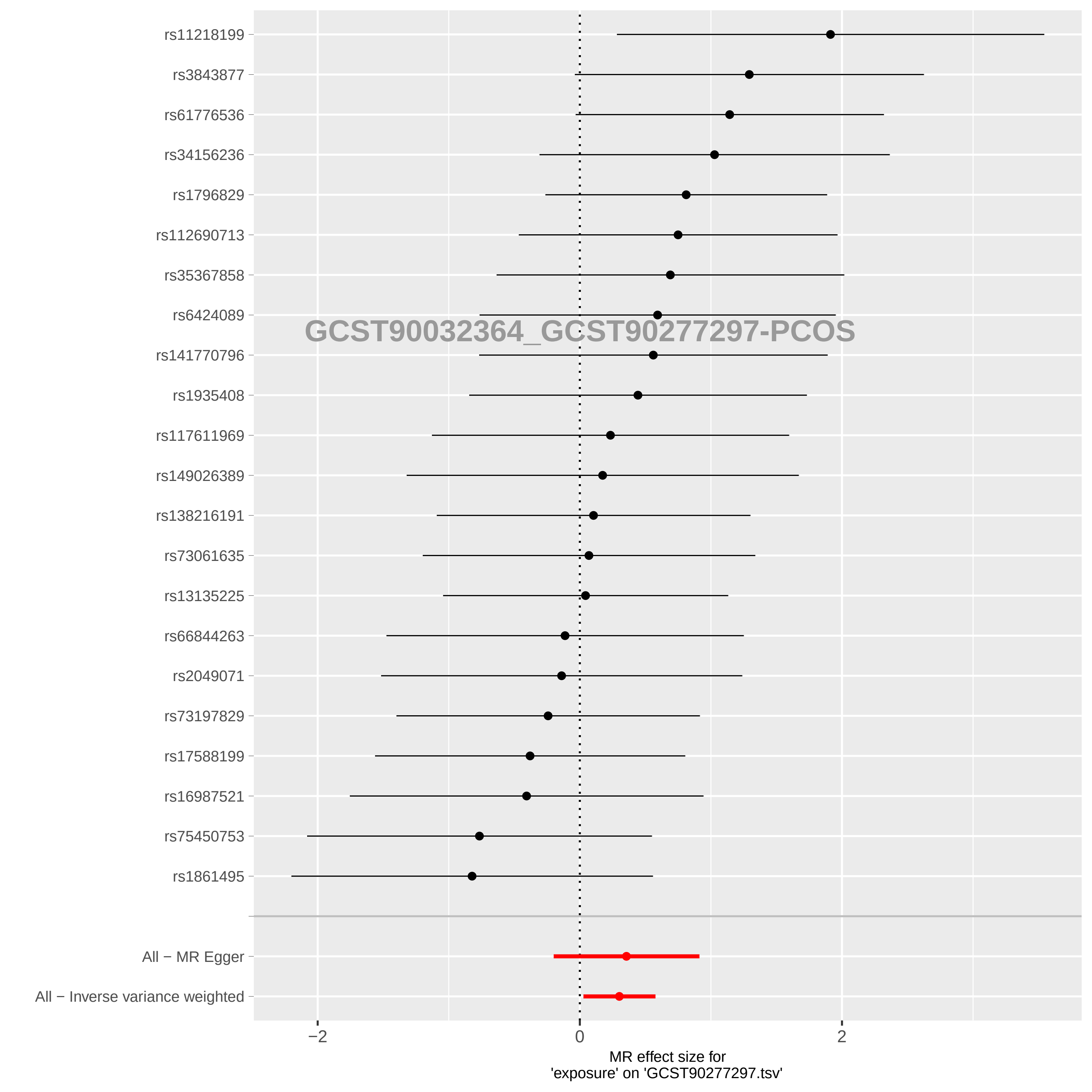
GCST90032364_GCST90277297-PCOS
rs11218199
rs3843877
rs61776536
rs34156236
rs1796829
rs112690713
rs35367858
rs6424089
rs141770796
rs1935408
rs117611969
rs149026389
rs138216191
rs73061635
rs13135225
rs66844263
rs2049071
rs73197829
rs17588199
rs16987521
rs75450753
rs1861495
All − MR Egger
All − Inverse variance weighted
−2
0
2
MR effect size for
'exposure' on 'GCST90277297.tsv'

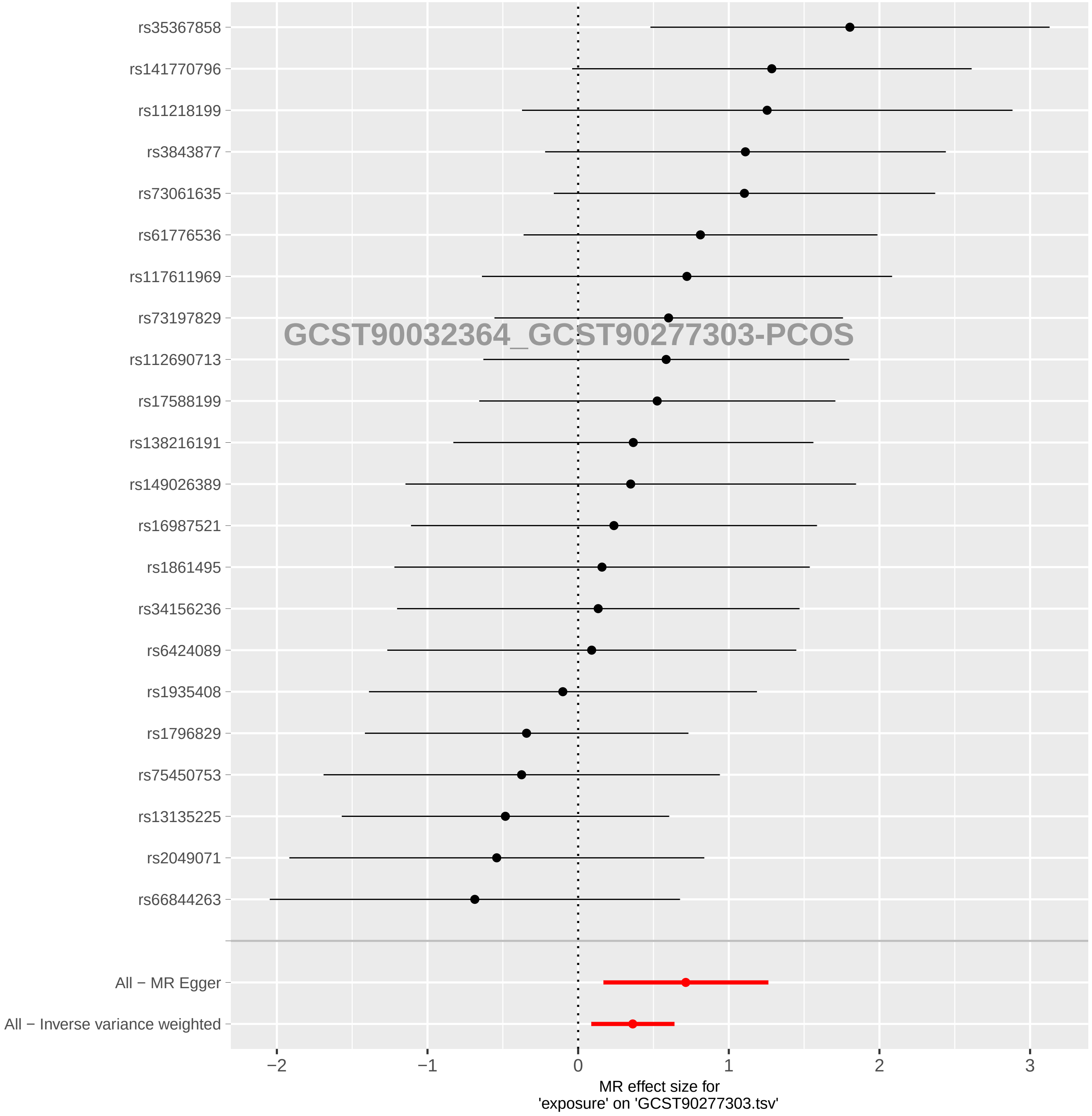
GCST90032364_GCST90277303-PCOS
rs35367858
rs141770796
rs11218199
rs3843877
rs73061635
rs61776536
rs117611969
rs73197829
rs112690713
rs17588199
rs138216191
rs149026389
rs16987521
rs1861495
rs34156236
rs6424089
rs1935408
rs1796829
rs75450753
rs13135225
rs2049071
rs66844263
All − MR Egger
All − Inverse variance weighted
−2
−1
0
1
2
3
MR effect size for
'exposure' on 'GCST90277303.tsv'

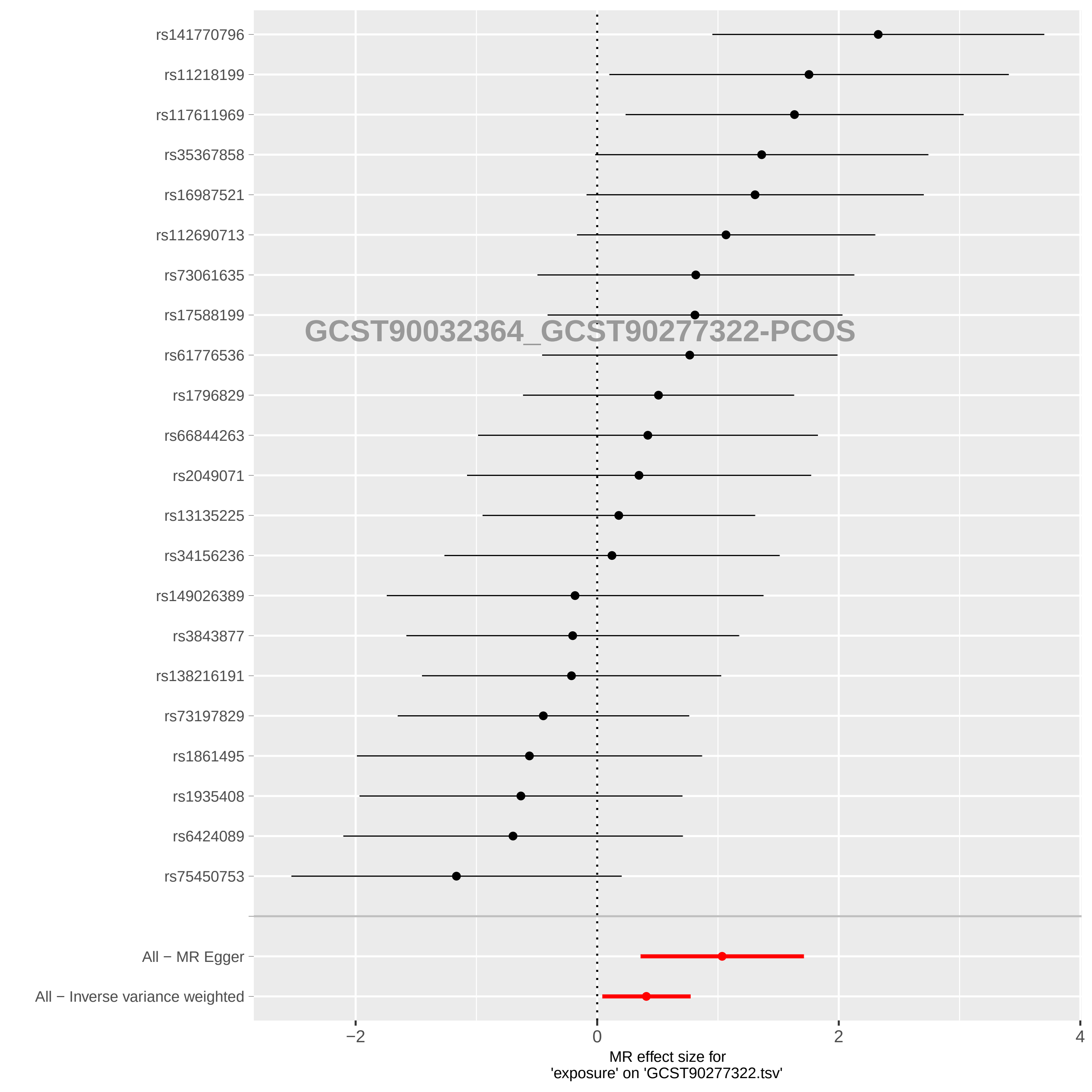

GCST90032364_GCST90277322-PCOS
rs141770796
rs11218199
rs117611969
rs35367858
rs16987521
rs112690713
rs73061635
rs17588199
rs61776536
rs1796829
rs66844263
rs2049071
rs13135225
rs34156236
rs149026389
rs3843877
rs138216191
rs73197829
rs1861495
rs1935408
rs6424089
rs75450753
All − MR Egger
All − Inverse variance weighted
−2
0
2
4
MR effect size for
'exposure' on 'GCST90277322.tsv'

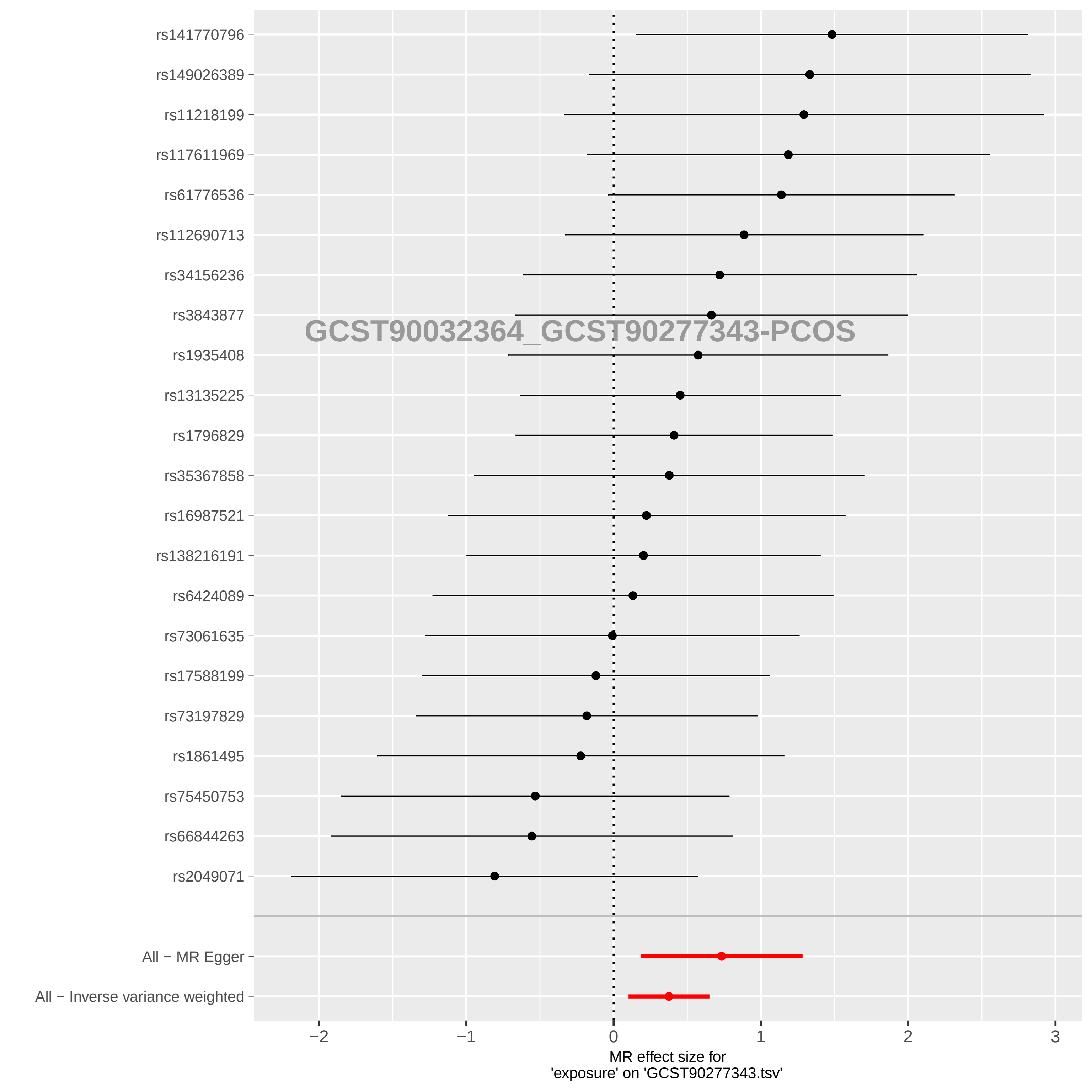

GCST90032364_GCST90277343-PCOS
rs141770796
rs149026389
rs11218199
rs117611969
rs61776536
rs112690713
rs34156236
rs3843877
rs1935408
rs13135225
rs1796829
rs35367858
rs16987521
rs138216191
rs6424089
rs73061635
rs17588199
rs73197829
rs1861495
rs75450753
rs66844263
rs2049071
All − MR Egger
All − Inverse variance weighted
−2
−1
0
1
2
3
MR effect size for
'exposure' on 'GCST90277343.tsv'

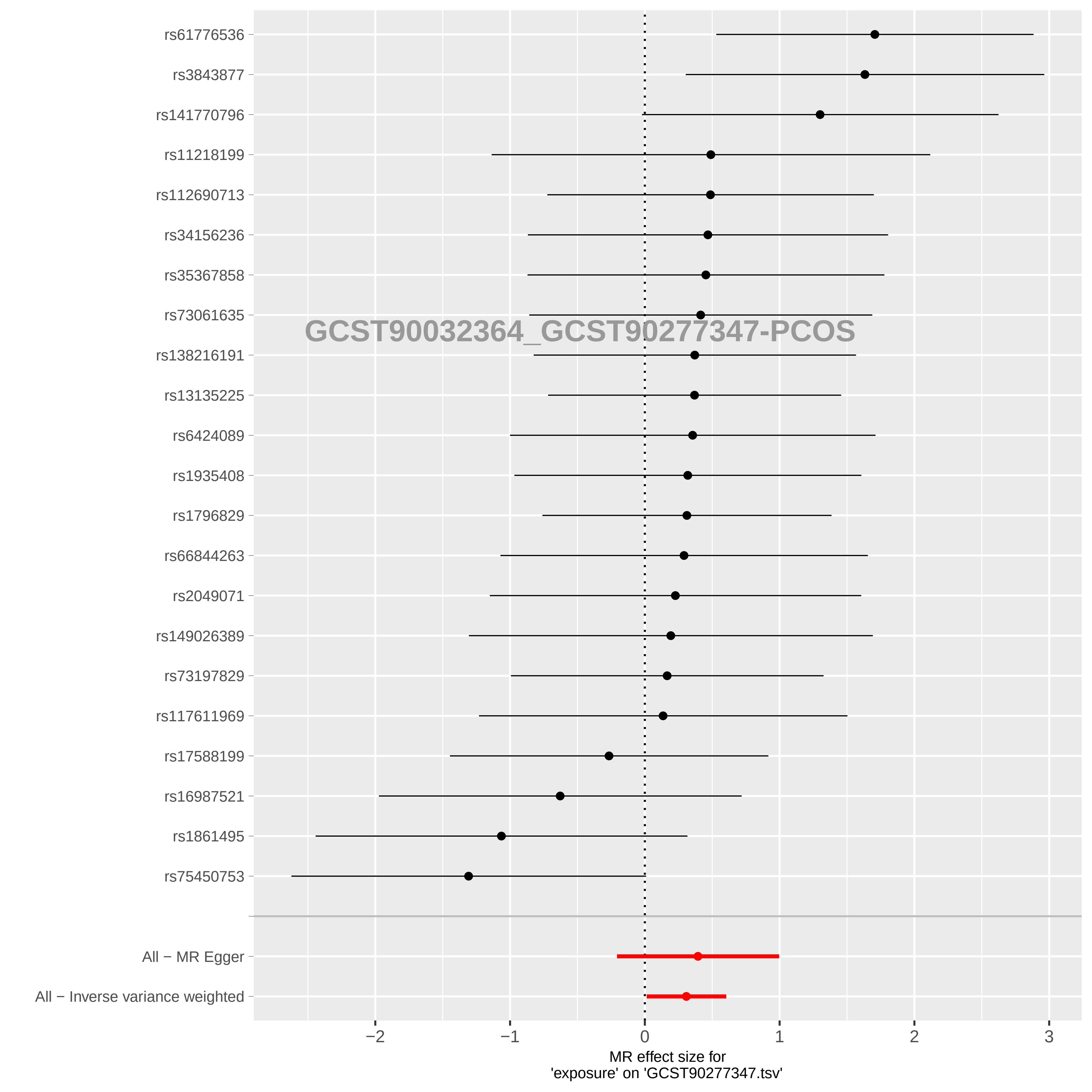

GCST90032364_GCST90277347-PCOS
rs61776536
rs3843877
rs141770796
rs11218199
rs112690713
rs34156236
rs35367858
rs73061635
rs138216191
rs13135225
rs6424089
rs1935408
rs1796829
rs66844263
rs2049071
rs149026389
rs73197829
rs117611969
rs17588199
rs16987521
rs1861495
rs75450753
All − MR Egger
All − Inverse variance weighted
−2
−1
0
1
2
3
MR effect size for
'exposure' on 'GCST90277347.tsv'

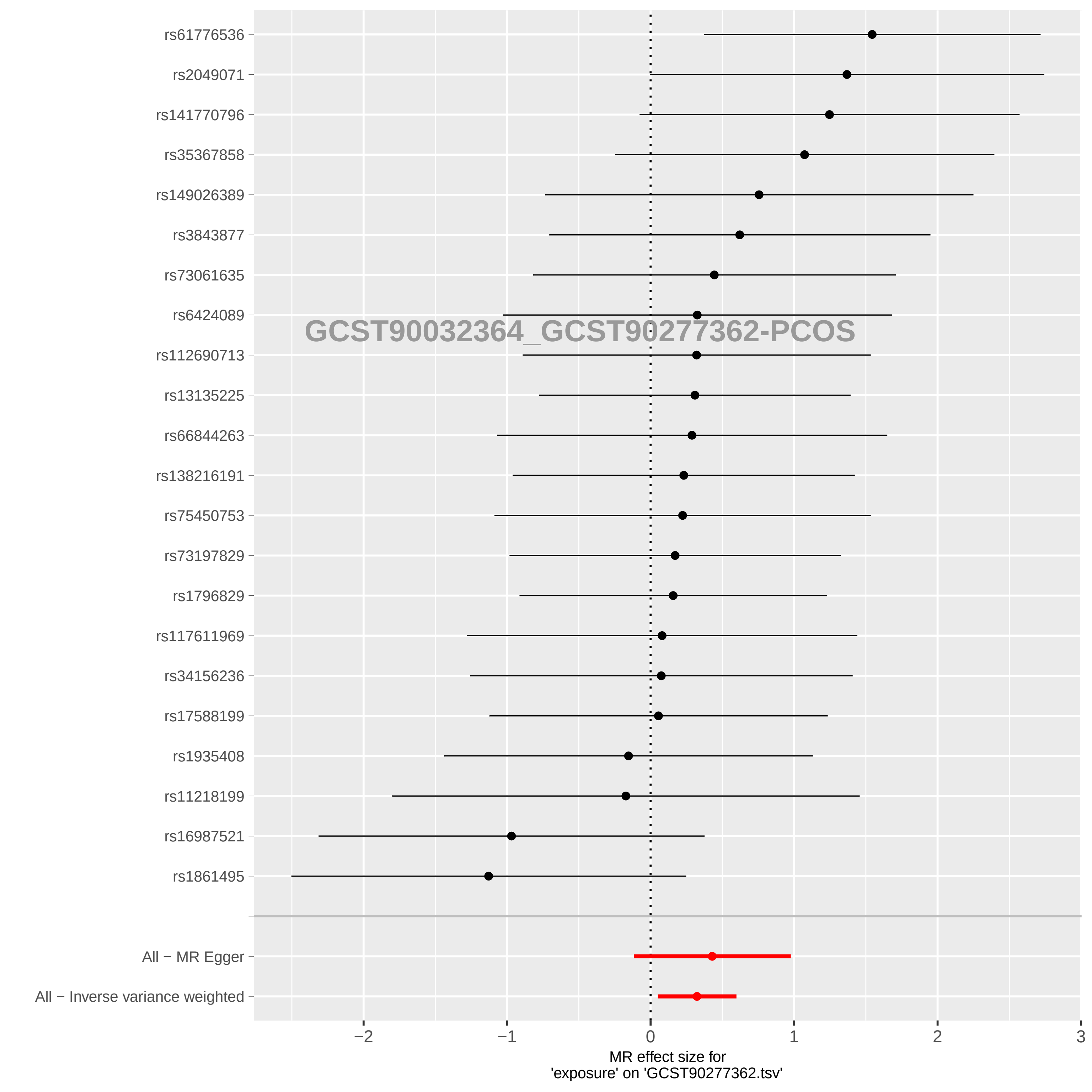

GCST90032364_GCST90277362-PCOS
rs61776536
rs2049071
rs141770796
rs35367858
rs149026389
rs3843877
rs73061635
rs6424089
rs112690713
rs13135225
rs66844263
rs138216191
rs75450753
rs73197829
rs1796829
rs117611969
rs34156236
rs17588199
rs1935408
rs11218199
rs16987521
rs1861495
All − MR Egger
All − Inverse variance weighted
−2
−1
0
1
2
3
MR effect size for
'exposure' on 'GCST90277362.tsv'

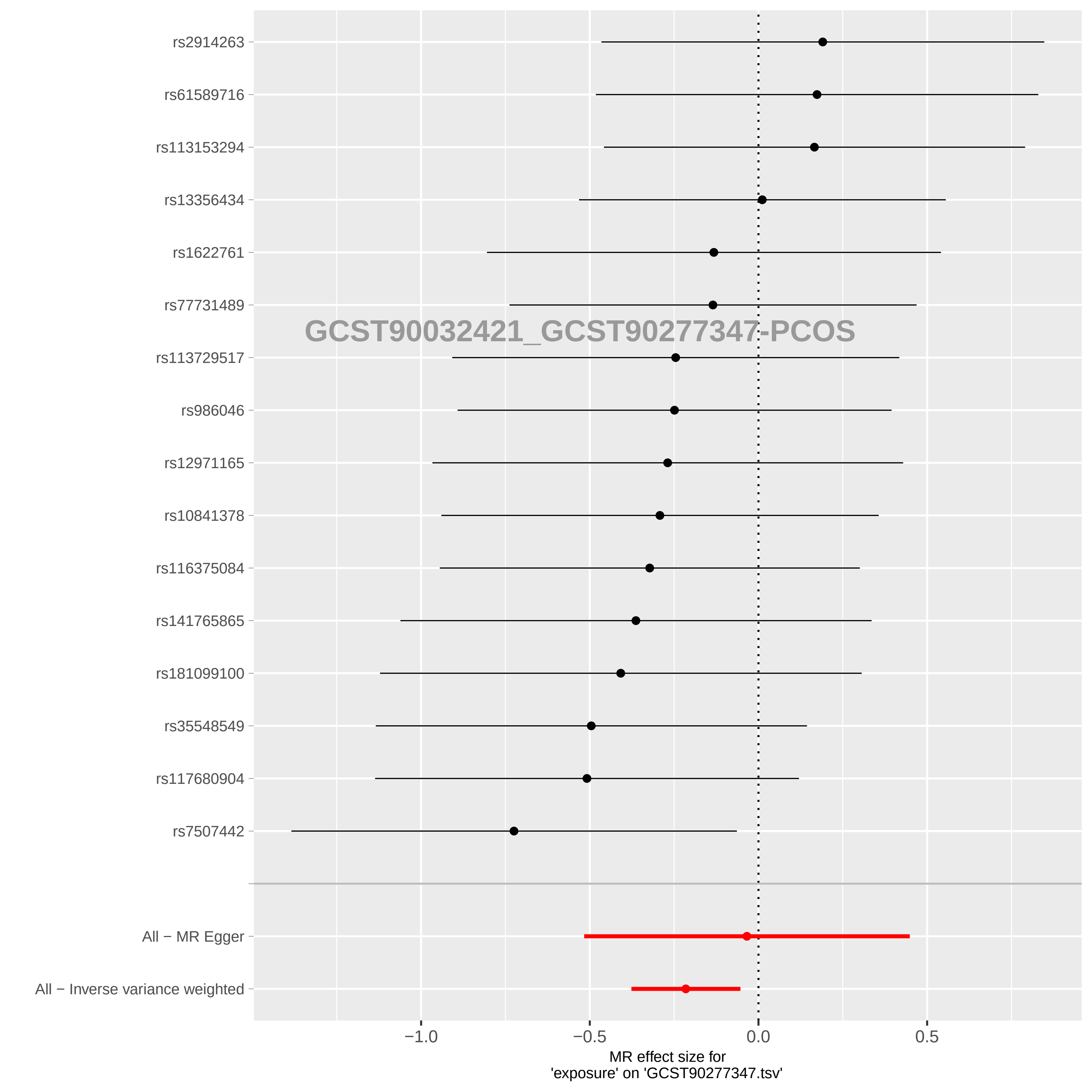
GCST90032421_GCST90277347-PCOS
rs2914263
rs61589716
rs113153294
rs13356434
rs1622761
rs77731489
rs113729517
rs986046
rs12971165
rs10841378
rs116375084
rs141765865
rs181099100
rs35548549
rs117680904
rs7507442
All − MR Egger
All − Inverse variance weighted
−1.0
−0.5
0.0
0.5
MR effect size for
'exposure' on 'GCST90277347.tsv'

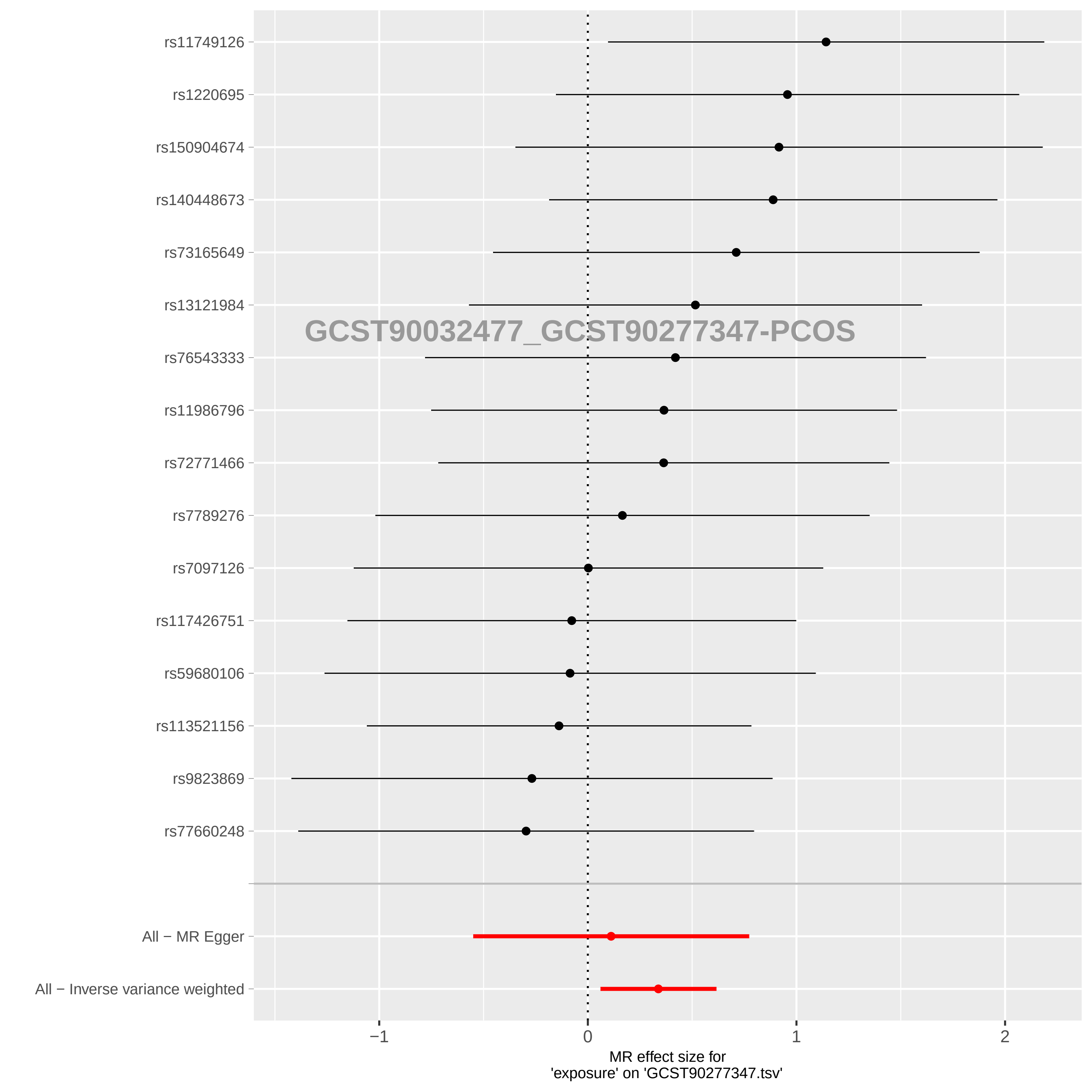
GCST90032477_GCST90277347-PCOS
rs11749126
rs1220695
rs150904674
rs140448673
rs73165649
rs13121984
rs76543333
rs11986796
rs72771466
rs7789276
rs7097126
rs117426751
rs59680106
rs113521156
rs9823869
rs77660248
All − MR Egger
All − Inverse variance weighted
−1
0
1
2
MR effect size for
'exposure' on 'GCST90277347.tsv'

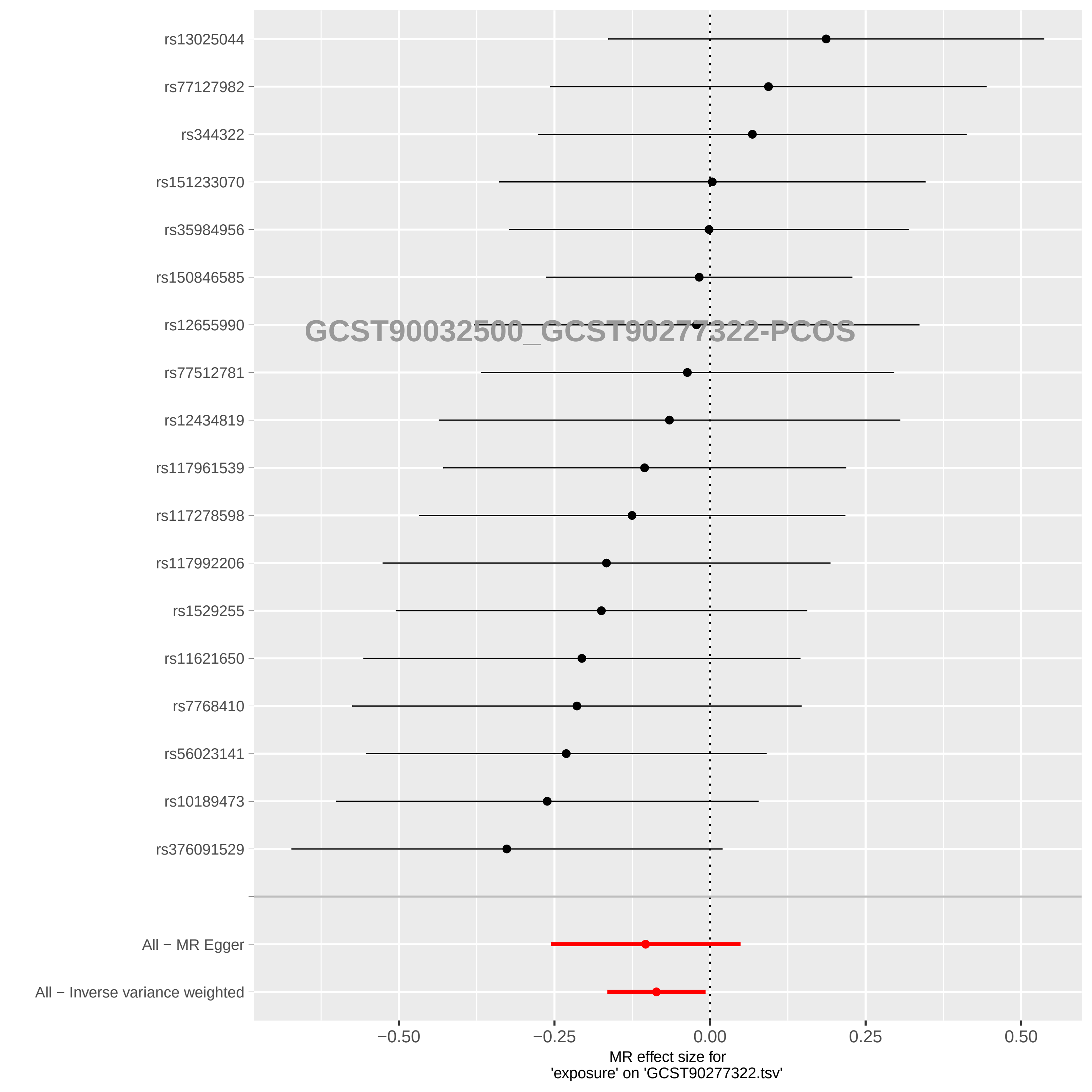

GCST90032500_GCST90277322-PCOS
rs13025044
rs77127982
rs344322
rs151233070
rs35984956
rs150846585
rs12655990
rs77512781
rs12434819
rs117961539
rs117278598
rs117992206
rs1529255
rs11621650
rs7768410
rs56023141
rs10189473
rs376091529
All − MR Egger
All − Inverse variance weighted
−0.50
−0.25
0.00
0.25
0.50
MR effect size for
'exposure' on 'GCST90277322.tsv'

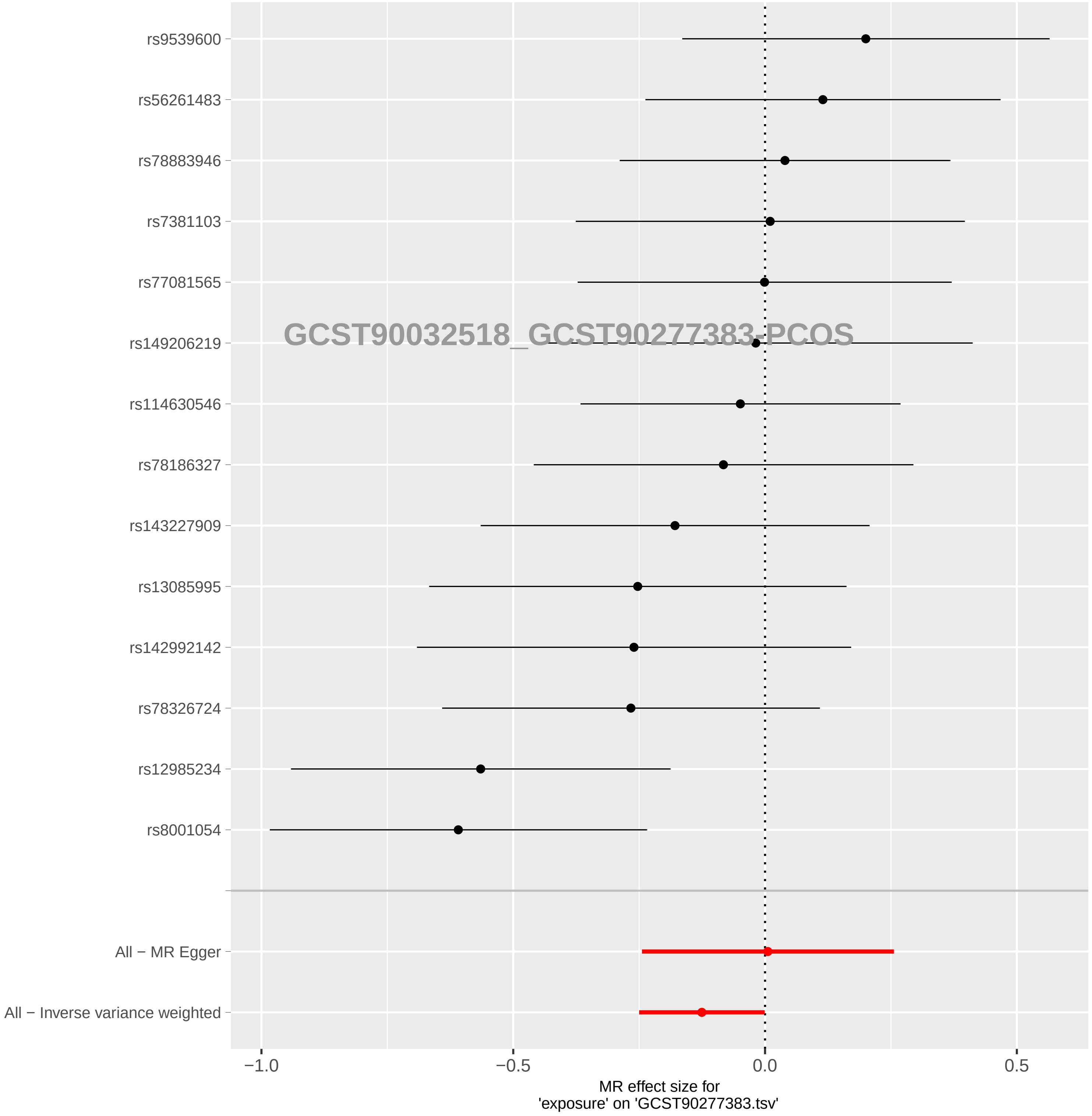
GCST90032518_GCST90277383-PCOS
rs9539600
rs56261483
rs78883946
rs7381103
rs77081565
rs149206219
rs114630546
rs78186327
rs143227909
rs13085995
rs142992142
rs78326724
rs12985234
rs8001054
All − MR Egger
All − Inverse variance weighted
−1.0
−0.5
0.0
0.5
MR effect size for
'exposure' on 'GCST90277383.tsv'

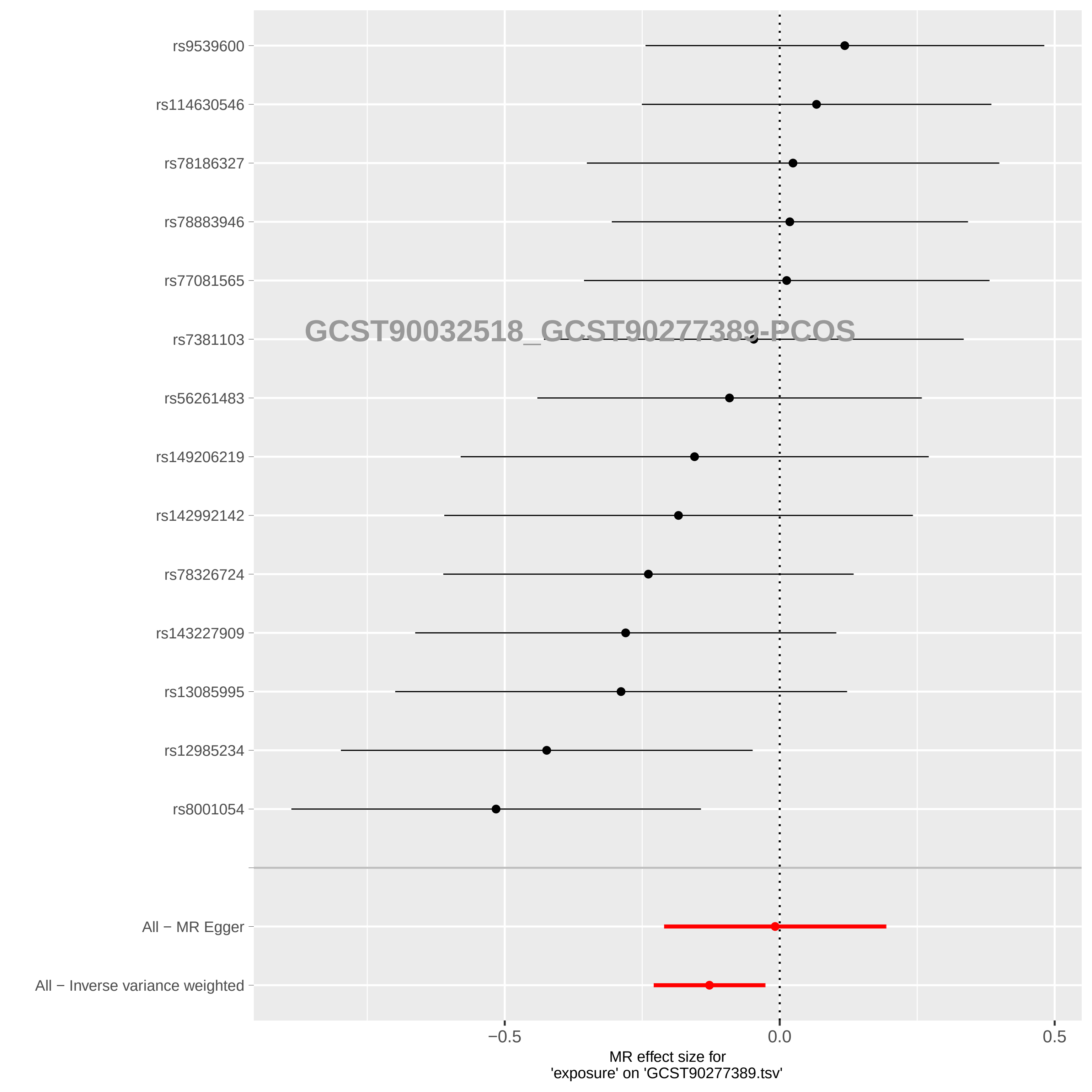
GCST90032518_GCST90277389-PCOS
rs9539600
rs114630546
rs78186327
rs78883946
rs77081565
rs7381103
rs56261483
rs149206219
rs142992142
rs78326724
rs143227909
rs13085995
rs12985234
rs8001054
All − MR Egger
All − Inverse variance weighted
−0.5
0.0
0.5
MR effect size for
'exposure' on 'GCST90277389.tsv'

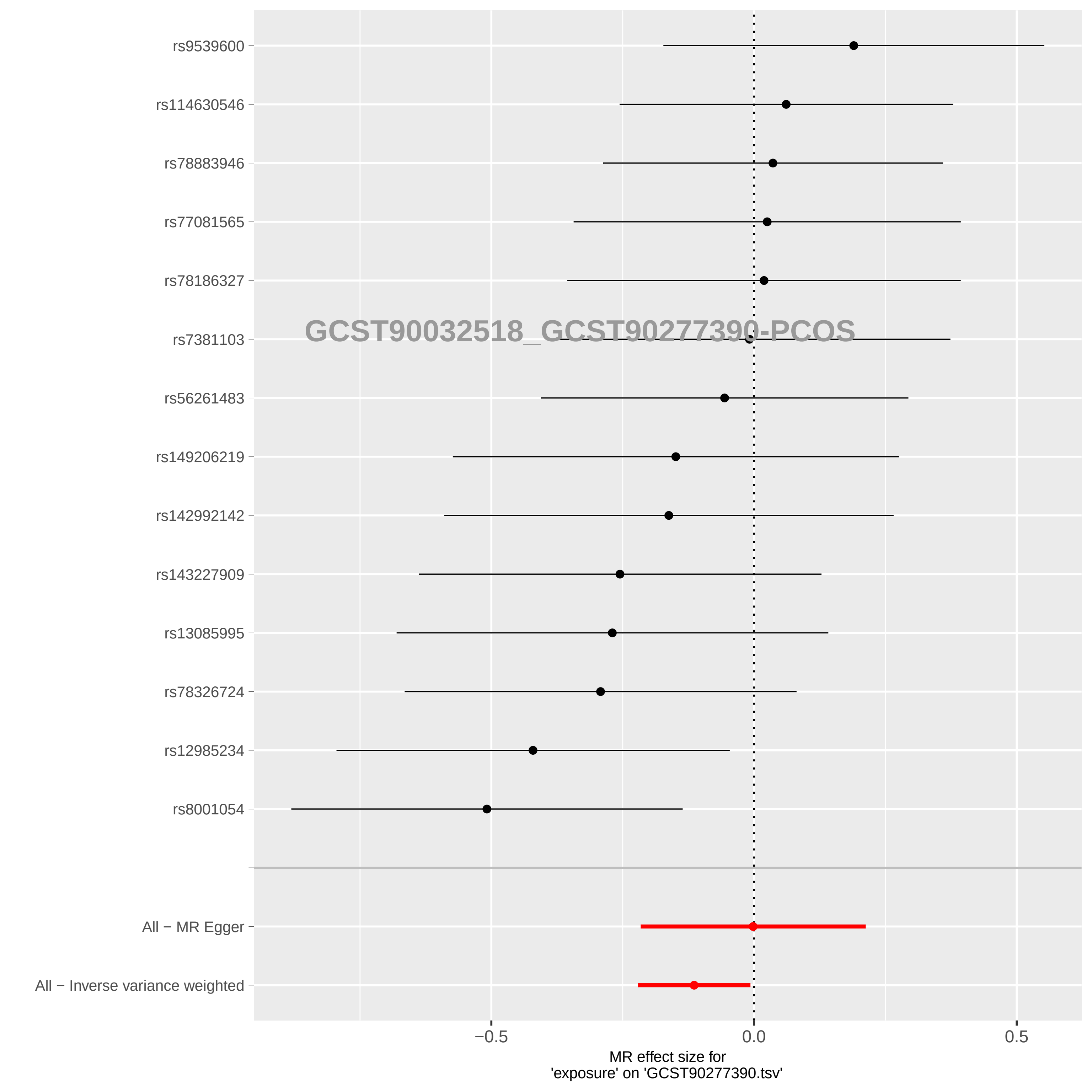

GCST90032518_GCST90277390-PCOS
rs9539600
rs114630546
rs78883946
rs77081565
rs78186327
rs7381103
rs56261483
rs149206219
rs142992142
rs143227909
rs13085995
rs78326724
rs12985234
rs8001054
All − MR Egger
All − Inverse variance weighted
−0.5
0.0
0.5
MR effect size for
'exposure' on 'GCST90277390.tsv'

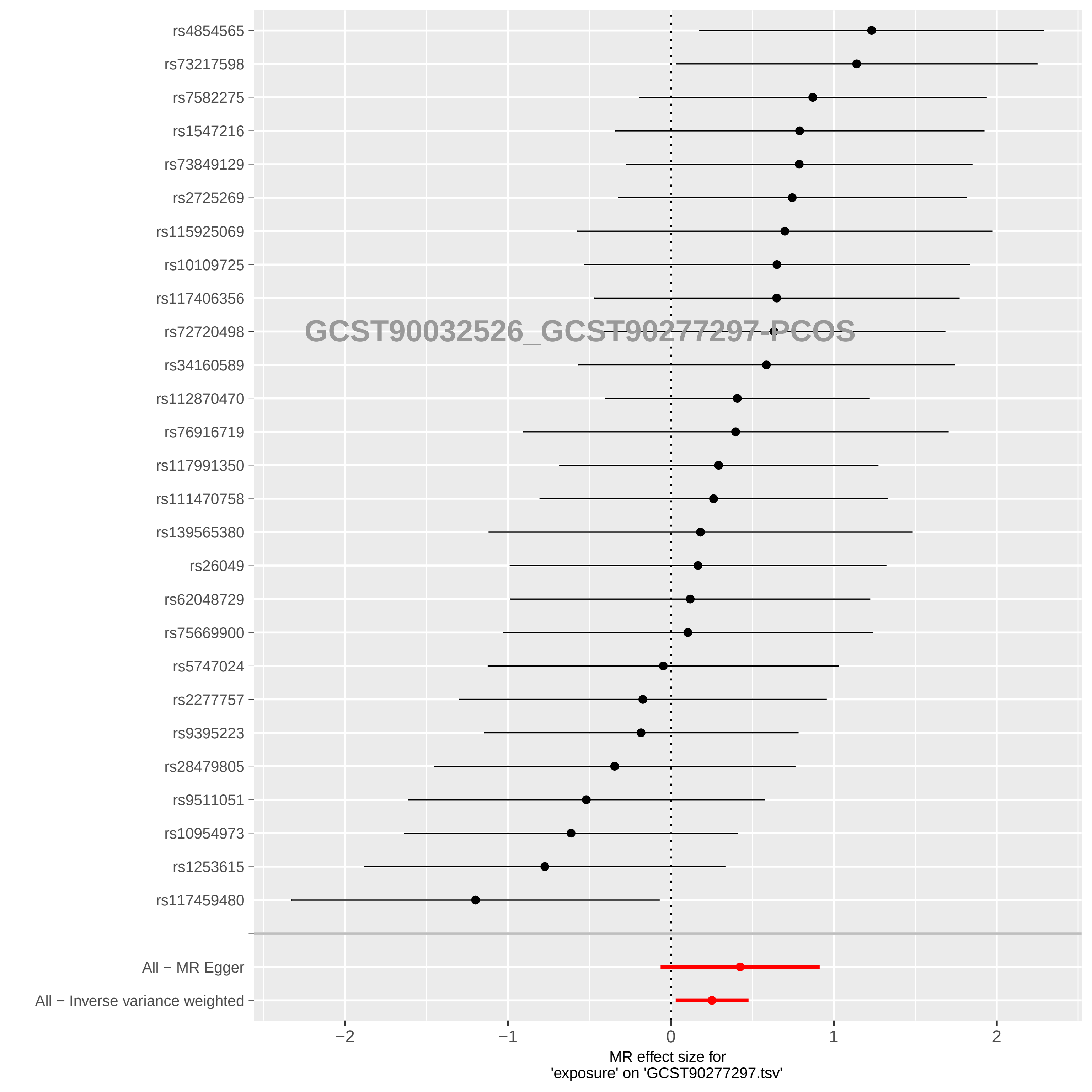

GCST90032526_GCST90277297-PCOS
rs4854565
rs73217598
rs7582275
rs1547216
rs73849129
rs2725269
rs115925069
rs10109725
rs117406356
rs72720498
rs34160589
rs112870470
rs76916719
rs117991350
rs111470758
rs139565380
rs26049
rs62048729
rs75669900
rs5747024
rs2277757
rs9395223
rs28479805
rs9511051
rs10954973
rs1253615
rs117459480
All − MR Egger
All − Inverse variance weighted
−2
−1
0
1
2
MR effect size for
'exposure' on 'GCST90277297.tsv'

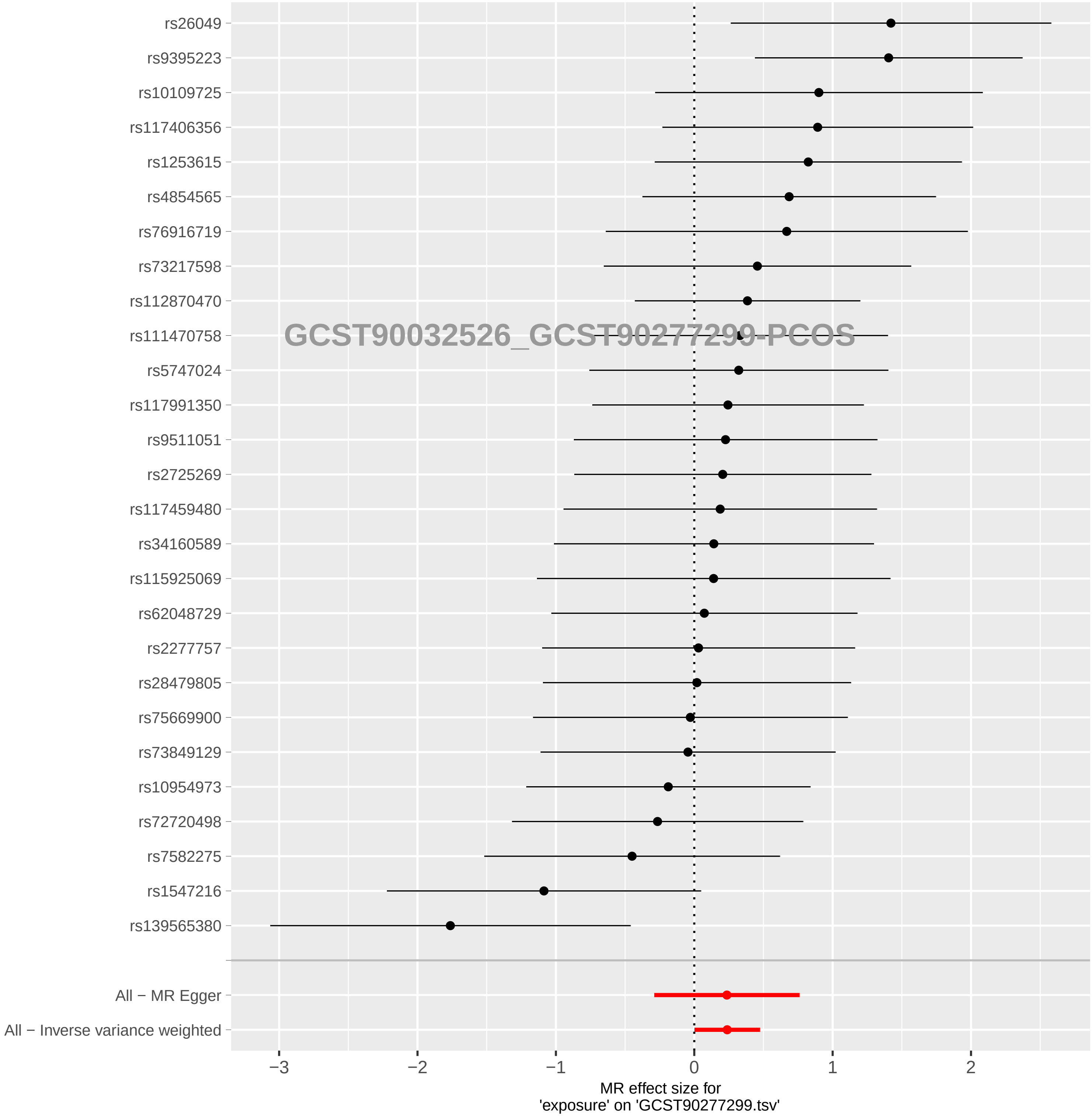

GCST90032526_GCST90277299-PCOS
rs26049
rs9395223
rs10109725
rs117406356
rs1253615
rs4854565
rs76916719
rs73217598
rs112870470
rs111470758
rs5747024
rs117991350
rs9511051
rs2725269
rs117459480
rs34160589
rs115925069
rs62048729
rs2277757
rs28479805
rs75669900
rs73849129
rs10954973
rs72720498
rs7582275
rs1547216
rs139565380
All − MR Egger
All − Inverse variance weighted
−3
−2
−1
0
1
2
MR effect size for
'exposure' on 'GCST90277299.tsv'

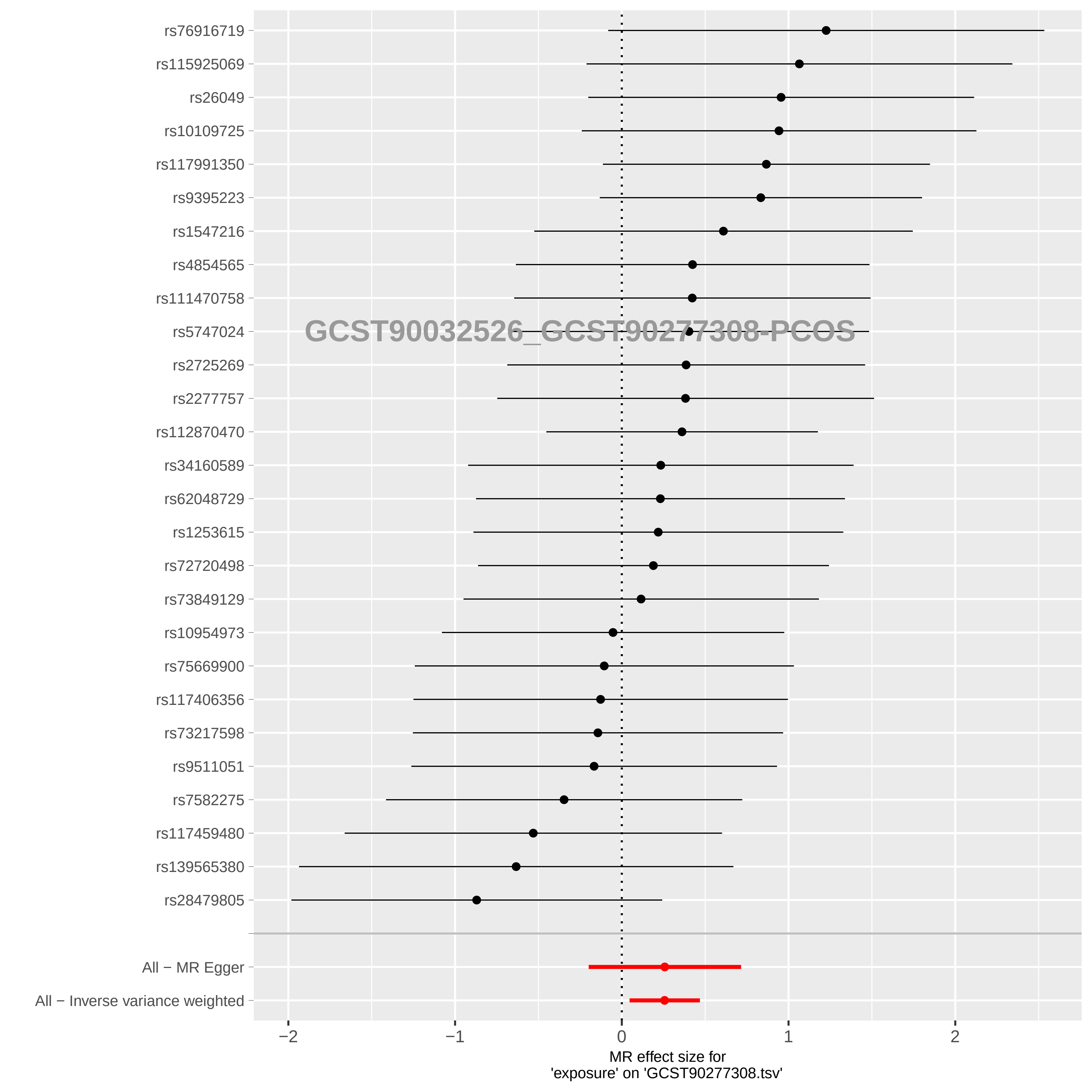

GCST90032526_GCST90277308-PCOS
rs76916719
rs115925069
rs26049
rs10109725
rs117991350
rs9395223
rs1547216
rs4854565
rs111470758
rs5747024
rs2725269
rs2277757
rs112870470
rs34160589
rs62048729
rs1253615
rs72720498
rs73849129
rs10954973
rs75669900
rs117406356
rs73217598
rs9511051
rs7582275
rs117459480
rs139565380
rs28479805
All − MR Egger
All − Inverse variance weighted
−2
−1
0
1
2
MR effect size for
'exposure' on 'GCST90277308.tsv'

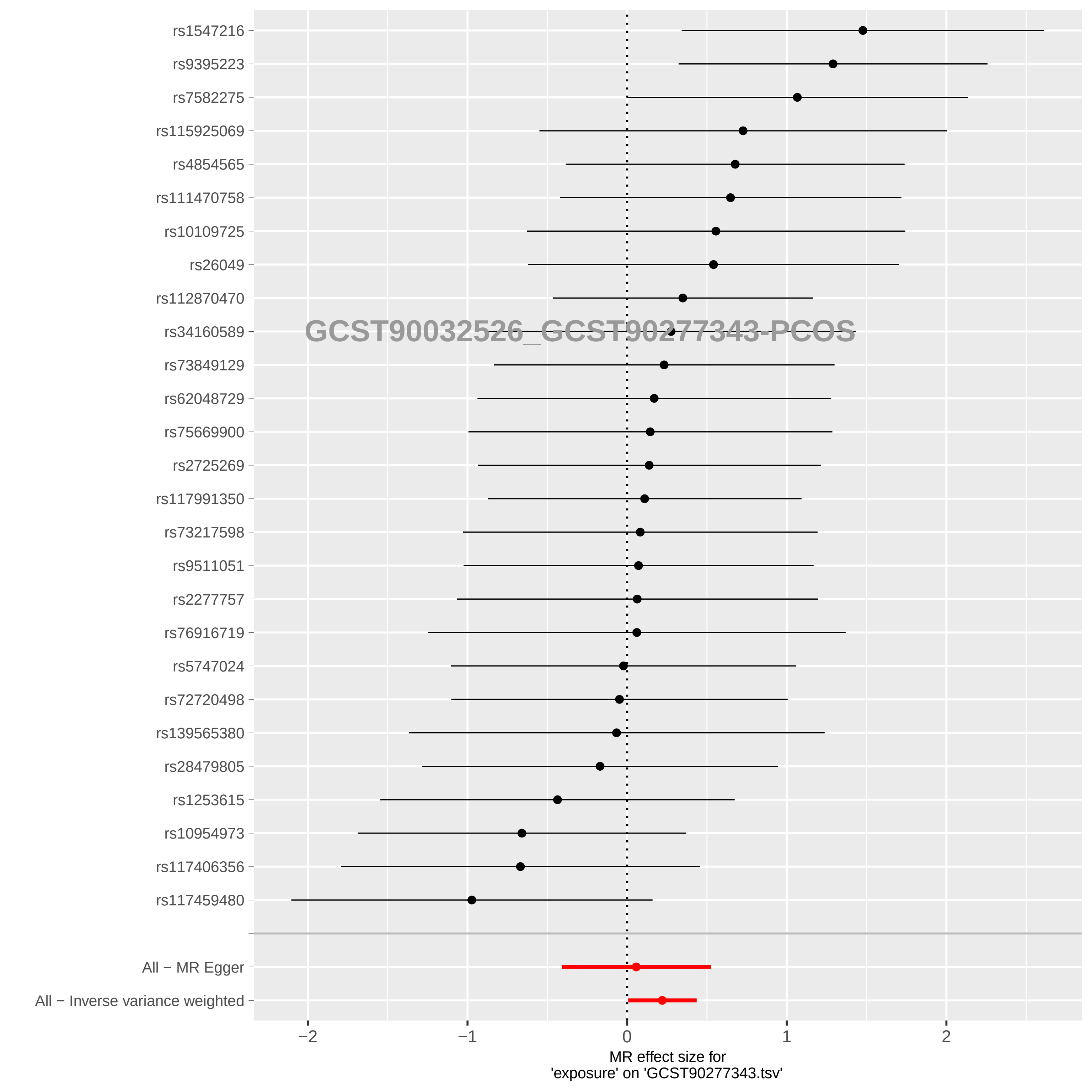

GCST90032526_GCST90277343-PCOS
rs1547216
rs9395223
rs7582275
rs115925069
rs4854565
rs111470758
rs10109725
rs26049
rs112870470
rs34160589
rs73849129
rs62048729
rs75669900
rs2725269
rs117991350
rs73217598
rs9511051
rs2277757
rs76916719
rs5747024
rs72720498
rs139565380
rs28479805
rs1253615
rs10954973
rs117406356
rs117459480
All − MR Egger
All − Inverse variance weighted
−2
−1
0
1
2
MR effect size for
'exposure' on 'GCST90277343.tsv'

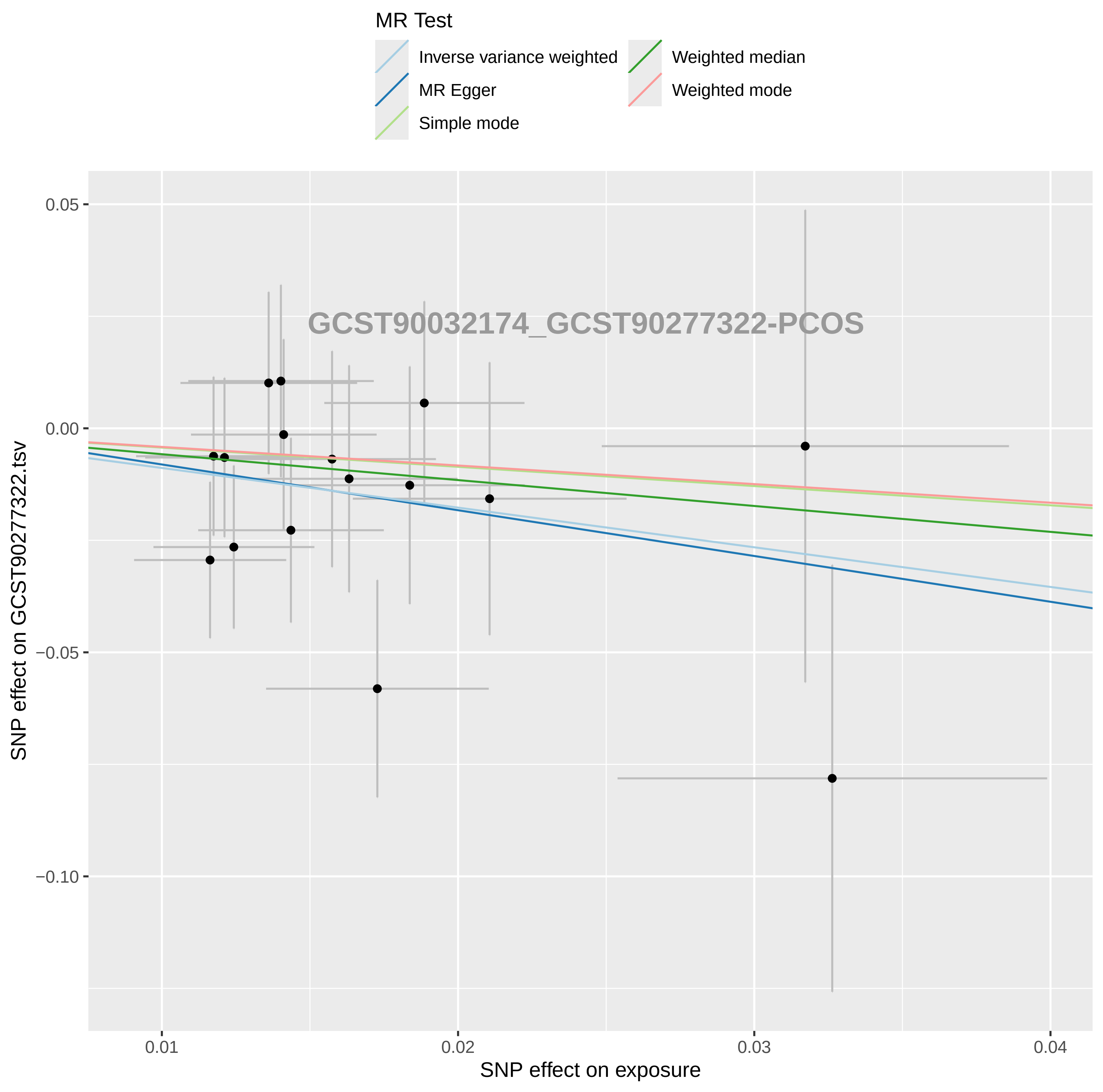

MR Test
Inverse variance weighted
MR Egger
Simple mode
Weighted median
Weighted mode
GCST90032174_GCST90277322-PCOS
0.05
0.00
−0.05
−0.10
SNP effect on GCST90277322.tsv
0.01
0.02
0.03
0.04
SNP effect on exposure

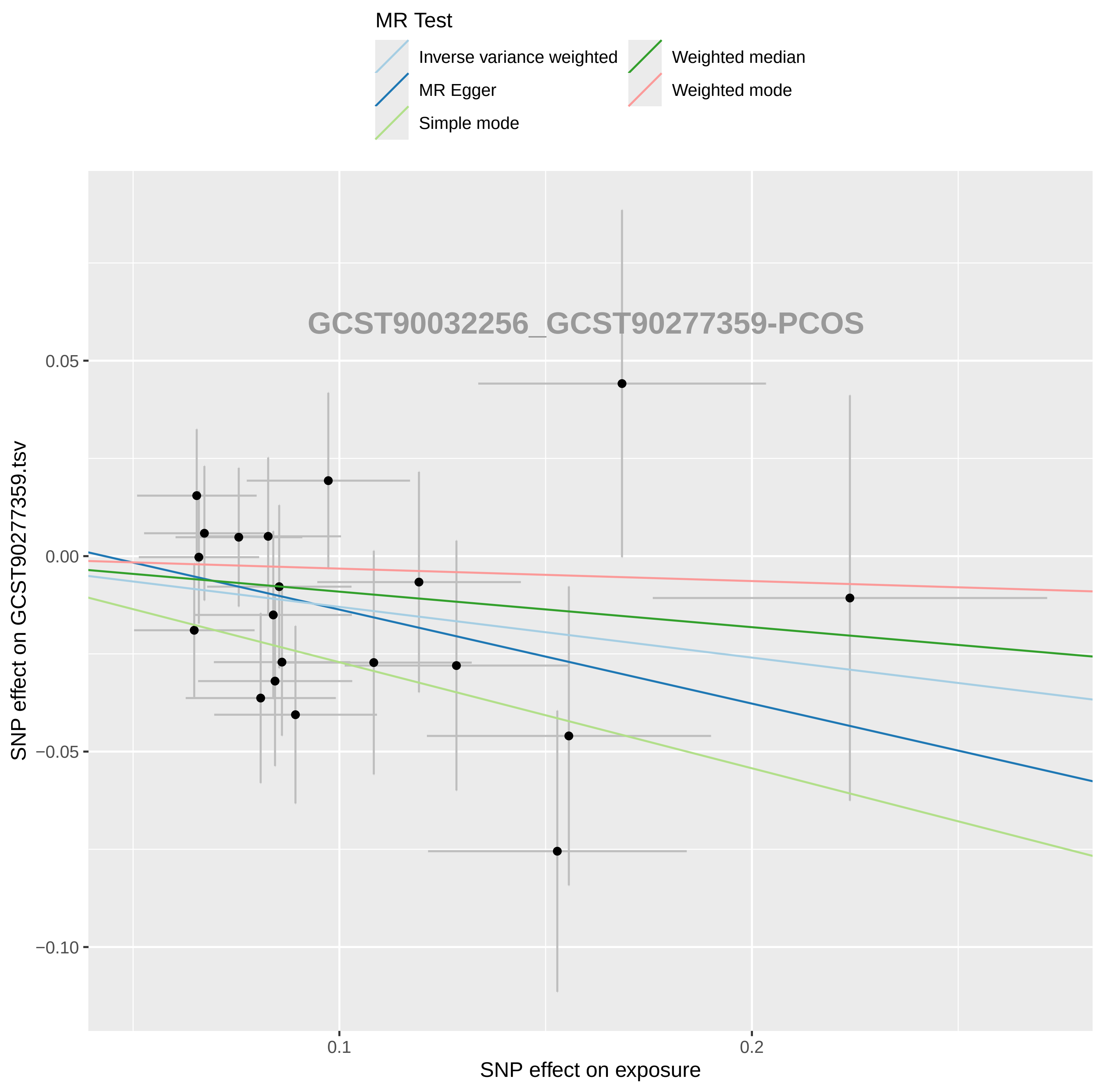

MR Test
Inverse variance weighted
MR Egger
Simple mode
Weighted median
Weighted mode
GCST90032256_GCST90277359-PCOS
0.05
0.00
−0.05
−0.10
0.1
0.2
SNP effect on exposure
SNP effect on GCST90277359.tsv

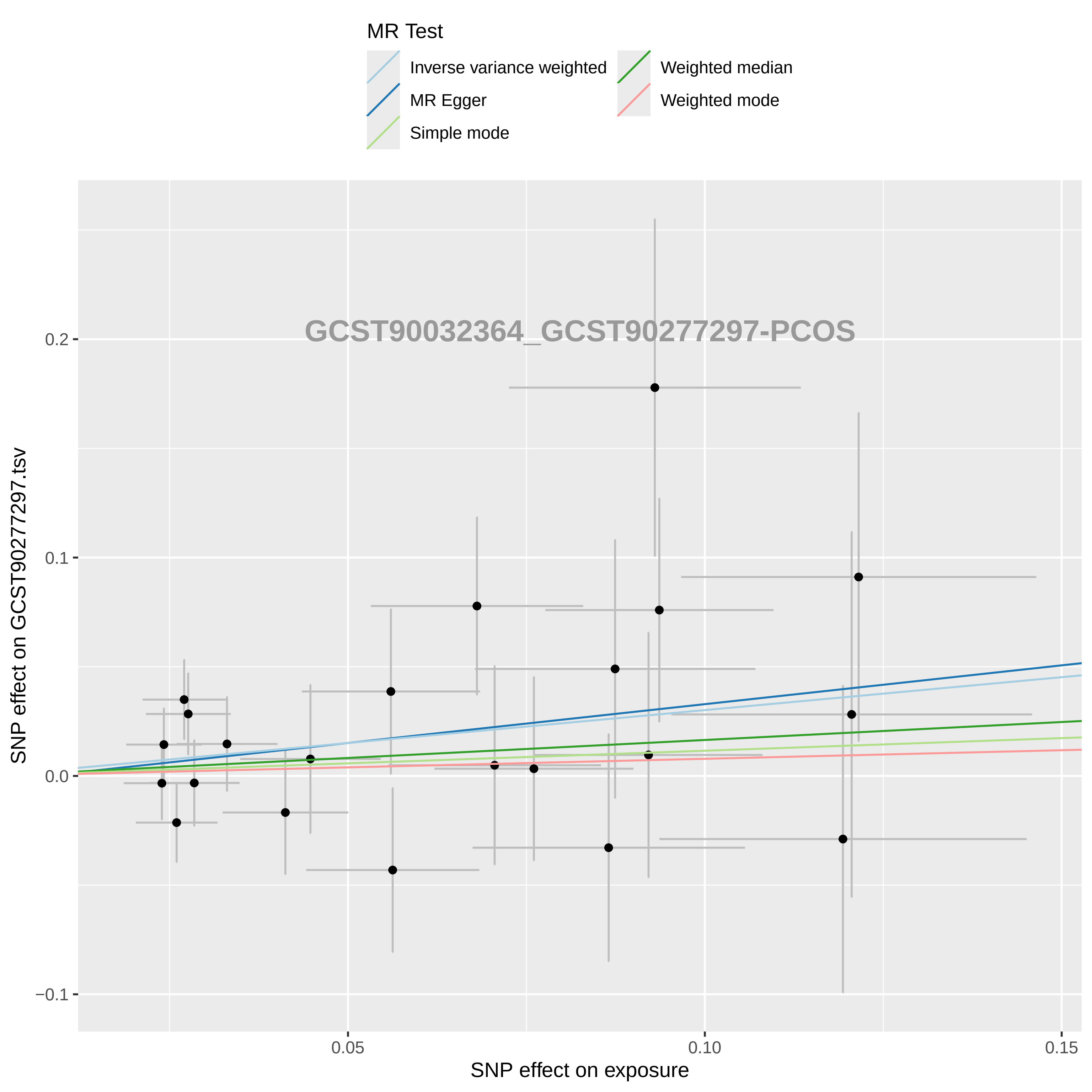

MR Test
Inverse variance weighted
MR Egger
Simple mode
Weighted median
Weighted mode
GCST90032364_GCST90277297-PCOS
SNP effect on GCST90277297.tsv
SNP effect on exposure
0.2
0.1
0.0
−0.1
0.05
0.10
0.15

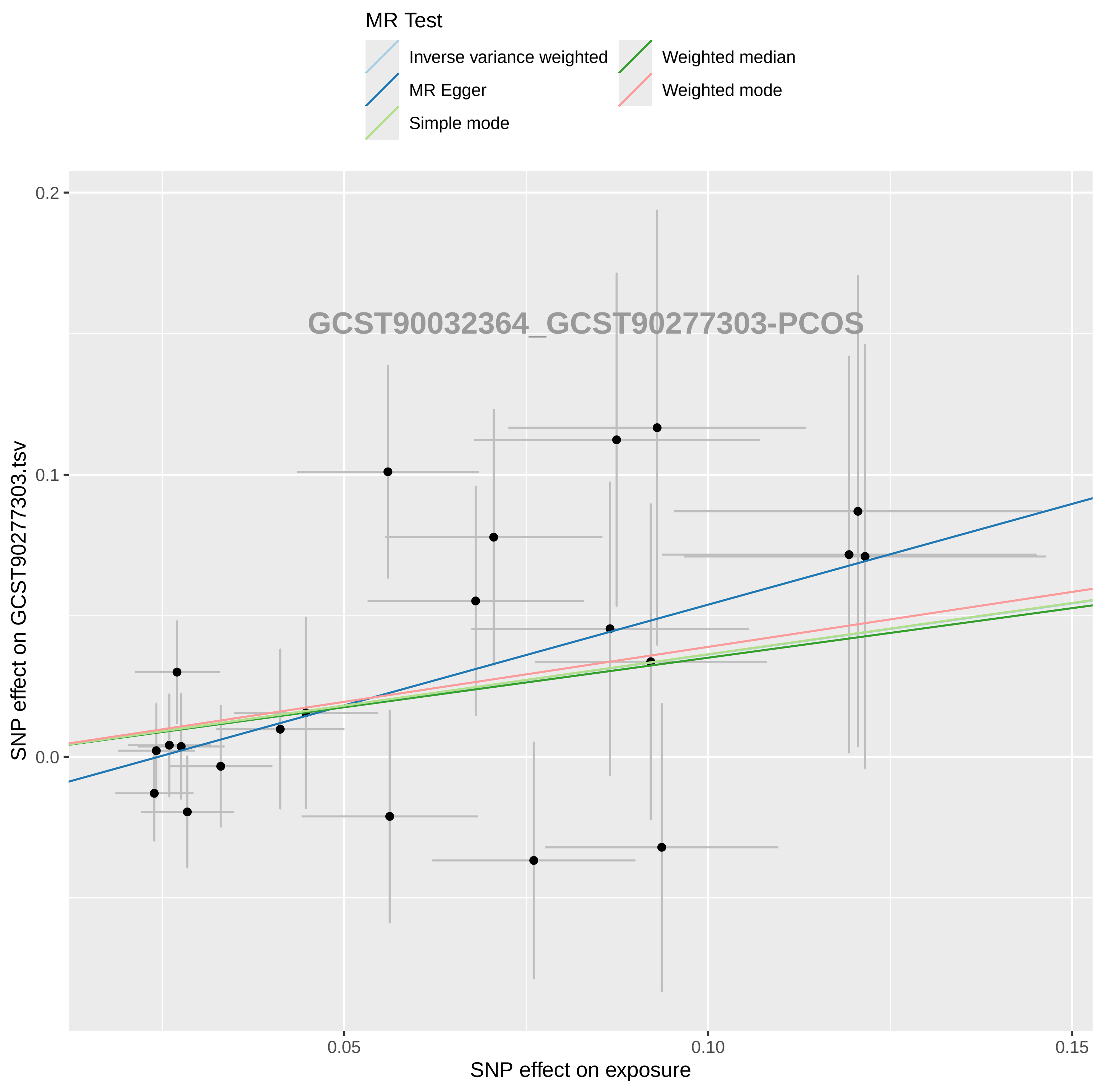
MR Test
Inverse variance weighted
MR Egger
Simple mode
Weighted median
Weighted mode
GCST90032364_GCST90277303-PCOS
0.2
0.1
0.0
0.05
0.10
0.15
SNP effect on GCST90277303.tsv
SNP effect on exposure

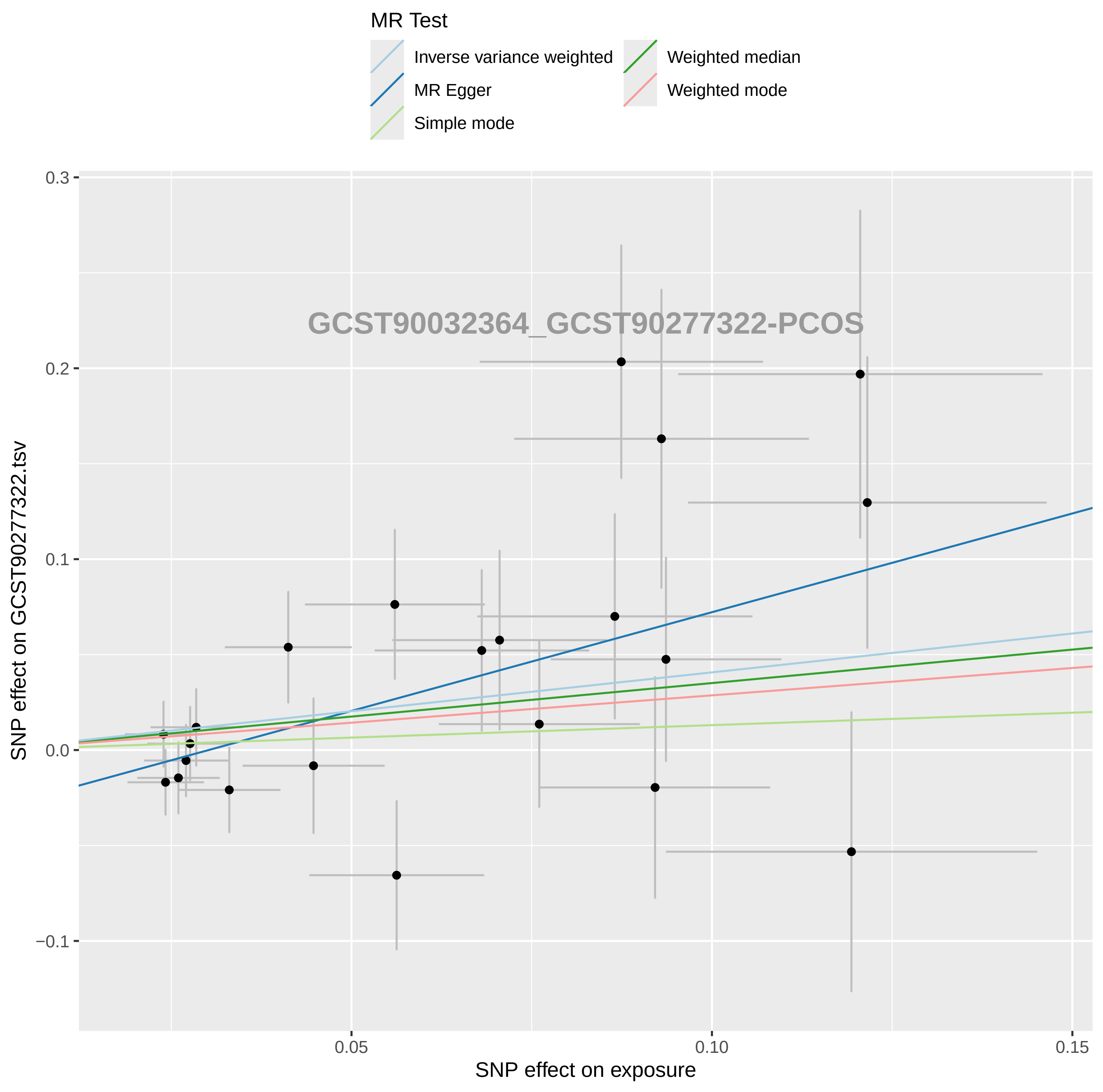
MR Test
Inverse variance weighted
MR Egger
Simple mode
Weighted median
Weighted mode
GCST90032364_GCST90277322-PCOS
SNP effect on GCST90277322.tsv
0.3
0.2
0.1
0.0
−0.1
0.05
0.10
0.15
SNP effect on exposure

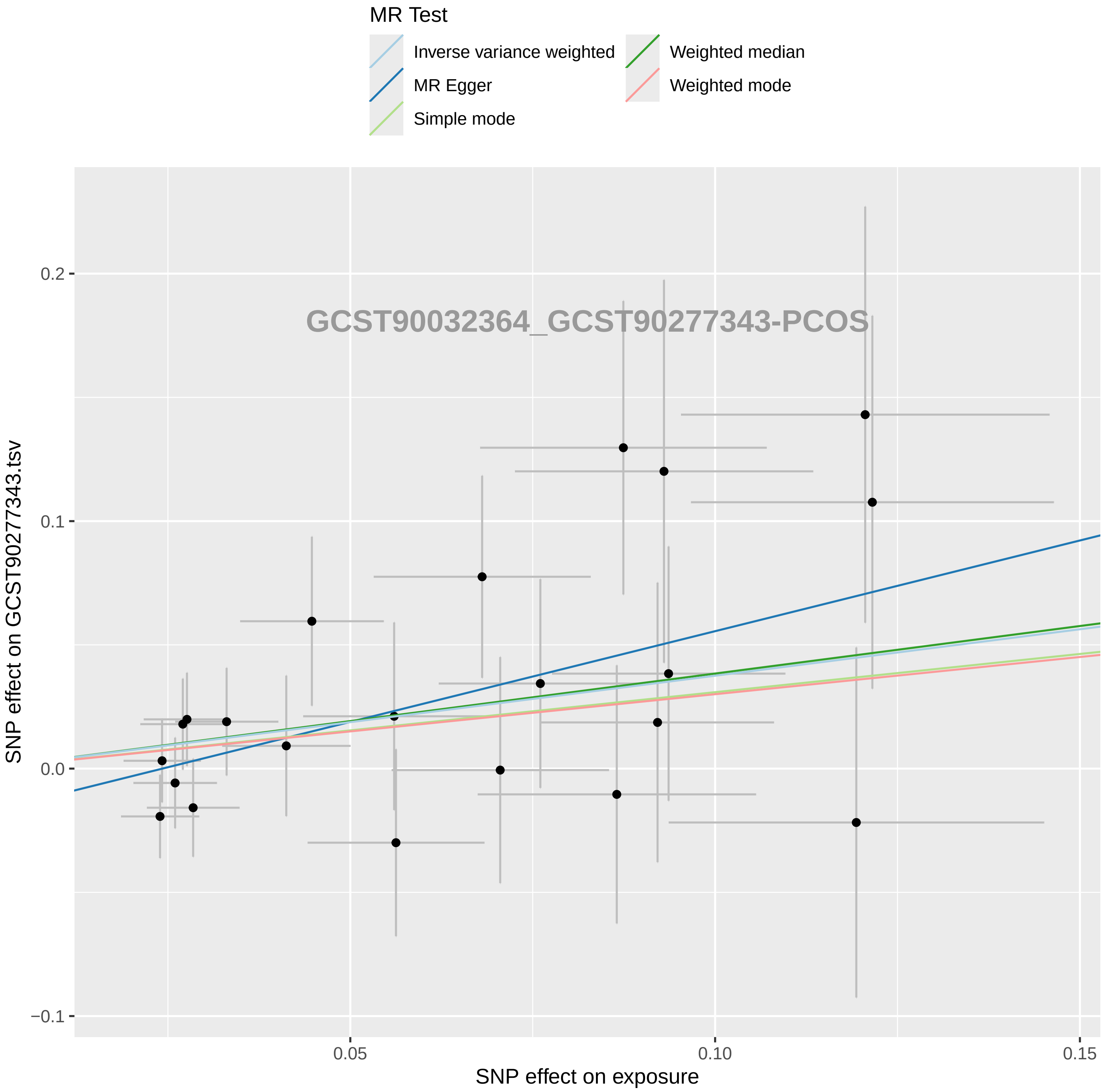
MR Test
Inverse variance weighted
MR Egger
Simple mode
Weighted median
Weighted mode
GCST90032364_GCST90277343-PCOS
SNP effect on GCST90277343.tsv
0.2
0.1
0.0
−0.1
0.05
0.10
0.15
SNP effect on exposure

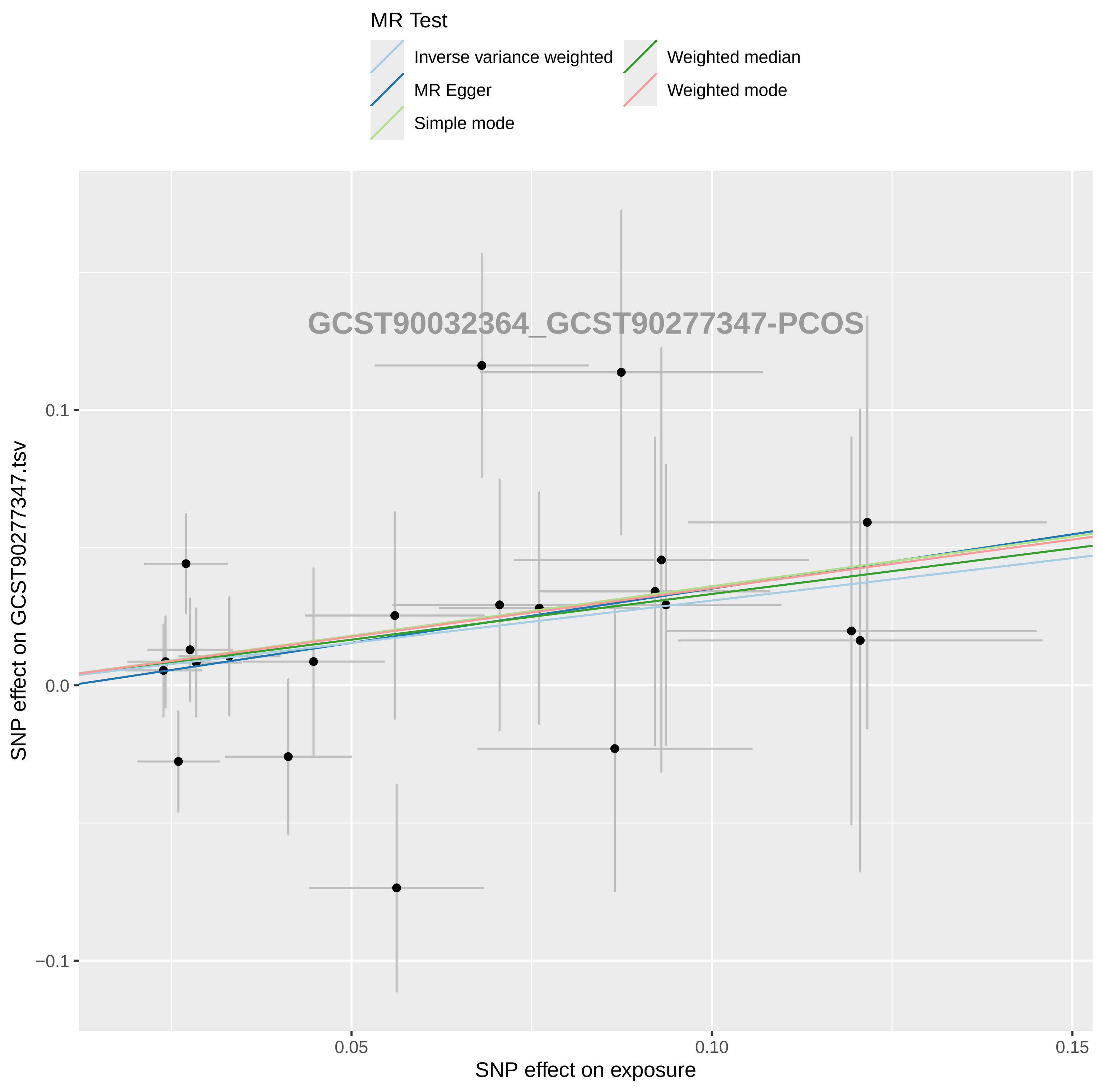

MR Test
Inverse variance weighted
MR Egger
Simple mode
Weighted median
Weighted mode
GCST90032364_GCST90277347-PCOS
SNP effect on GCST90277347.tsv
0.1
0.0
−0.1
0.05
0.10
0.15
SNP effect on exposure

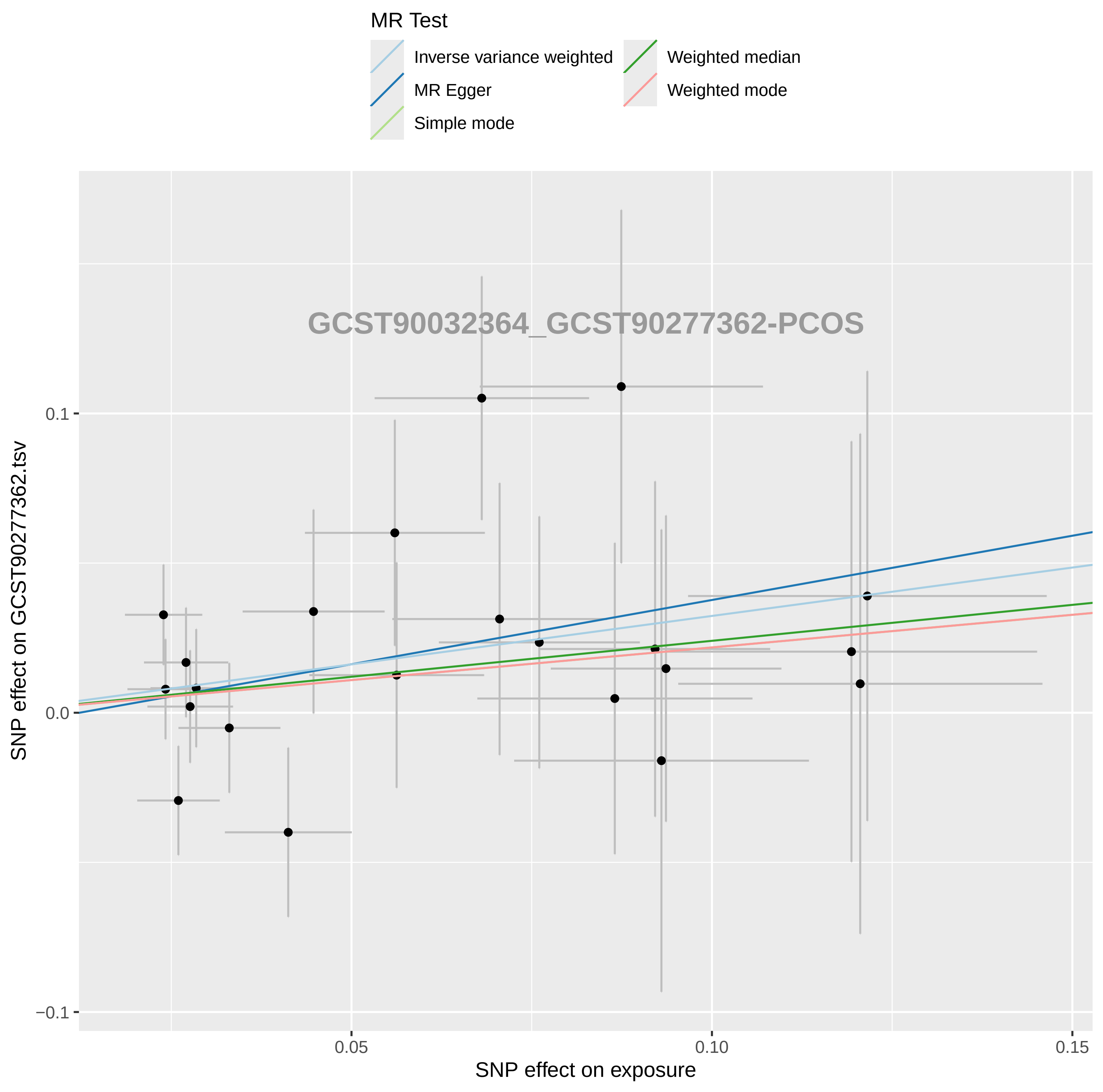

MR Test
Inverse variance weighted
MR Egger
Simple mode
Weighted median
Weighted mode
GCST90032364_GCST90277362-PCOS
SNP effect on GCST90277362.tsv
SNP effect on exposure
0.1
0.0
−0.1
0.05
0.10
0.15

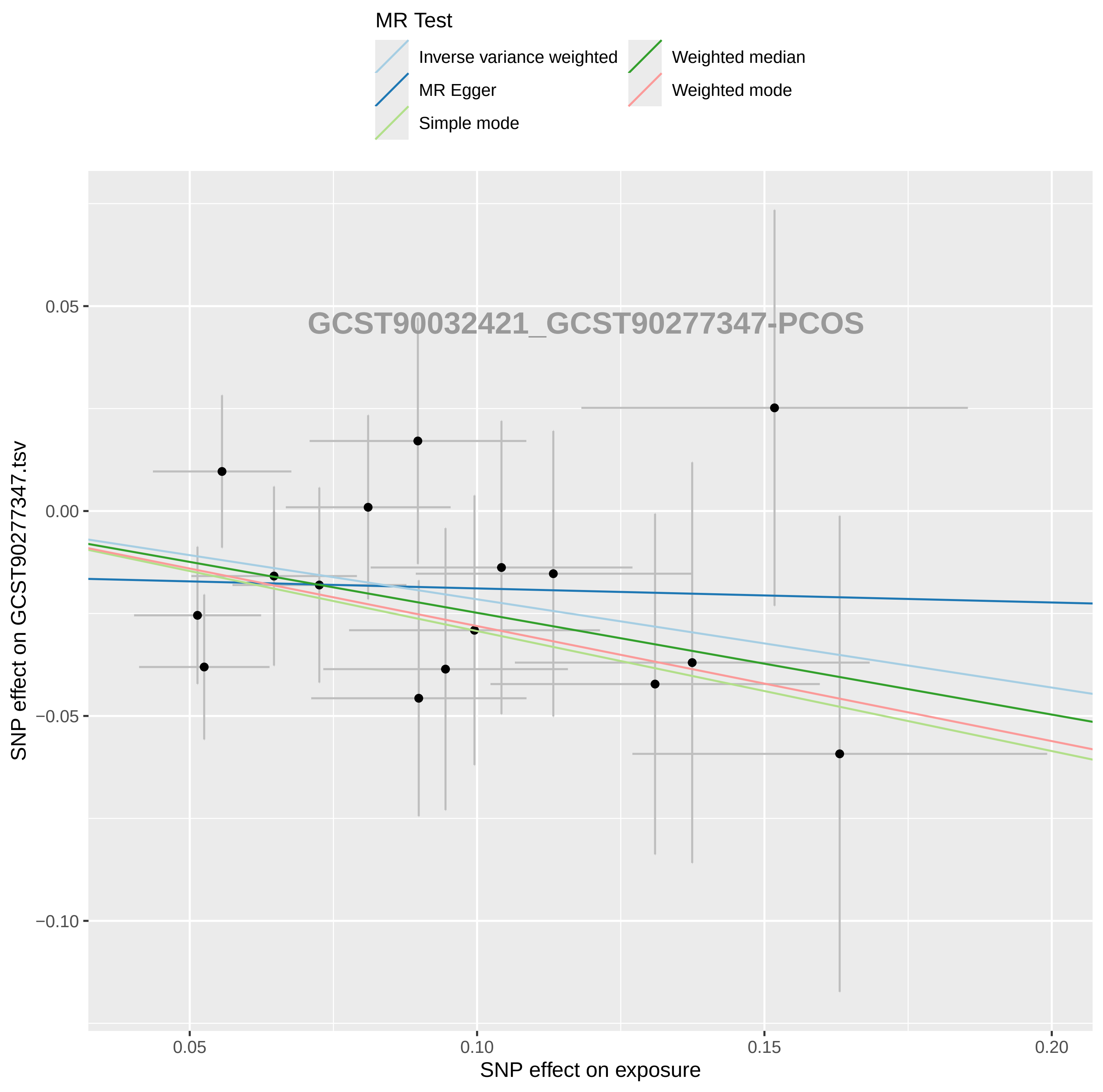
MR Test
Inverse variance weighted
MR Egger
Simple mode
Weighted median
Weighted mode
GCST90032421_GCST90277347-PCOS
SNP effect on GCST90277347.tsv
0.05
0.00
−0.05
−0.10
0.05
0.10
0.15
0.20
SNP effect on exposure

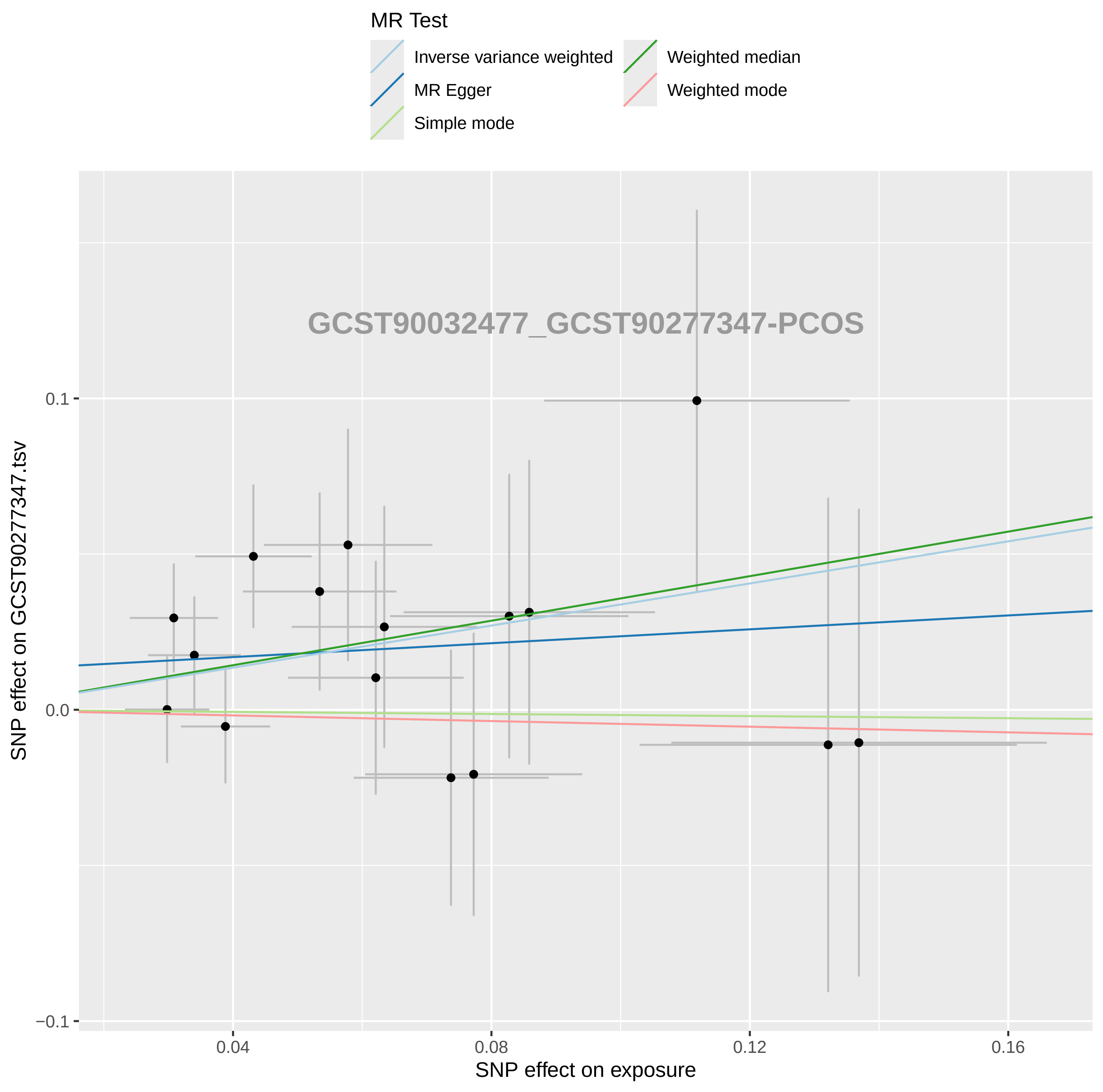
MR Test
Inverse variance weighted
MR Egger
Simple mode
Weighted median
Weighted mode
GCST90032477_GCST90277347-PCOS
0.1
0.0
−0.1
SNP effect on GCST90277347.tsv
0.04
0.08
0.12
0.16
SNP effect on exposure

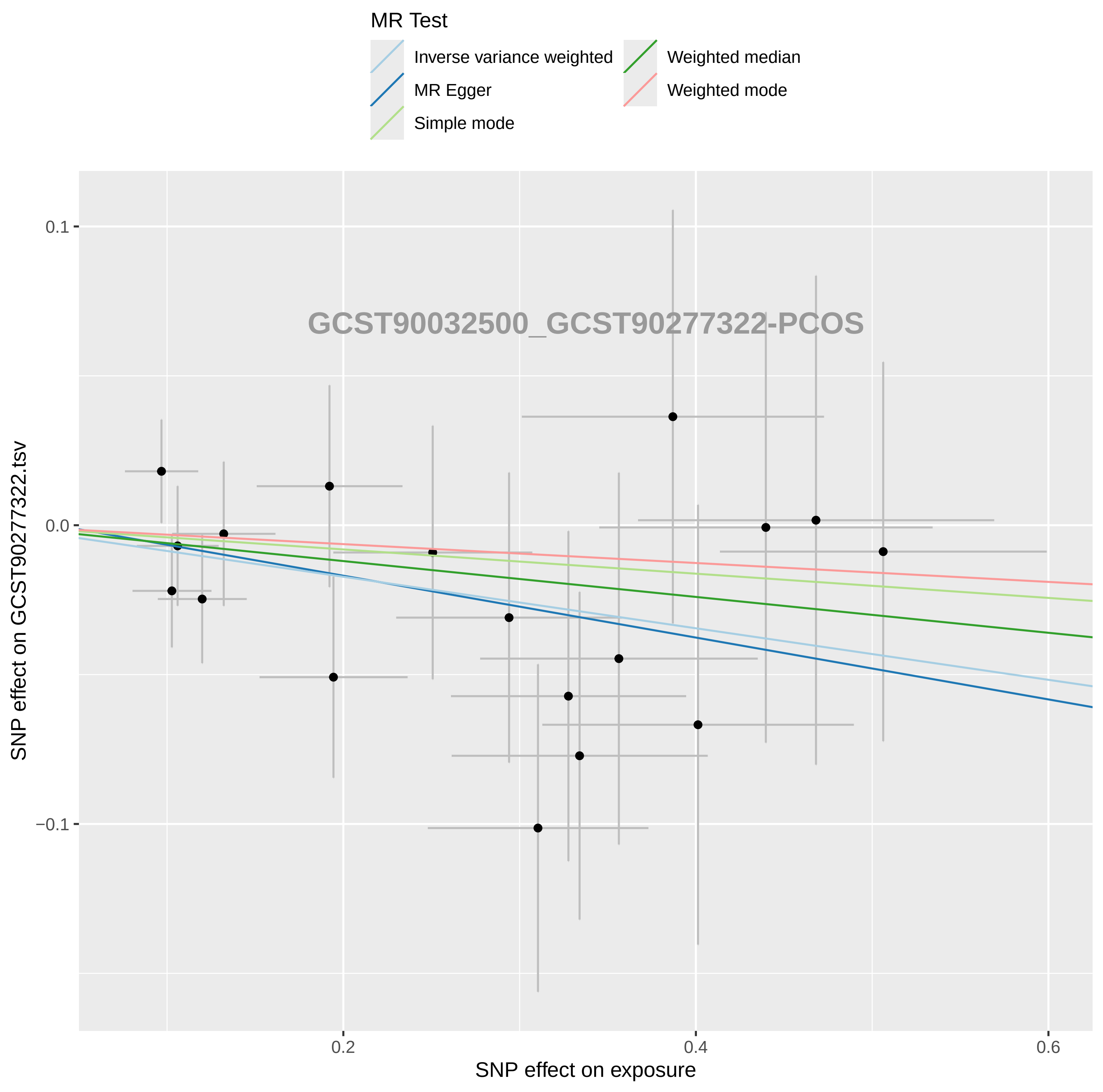
MR Test
Inverse variance weighted
MR Egger
Simple mode
Weighted median
Weighted mode
GCST90032500_GCST90277322-PCOS
0.1
0.0
−0.1
SNP effect on GCST90277322.tsv
0.2
0.4
0.6
SNP effect on exposure

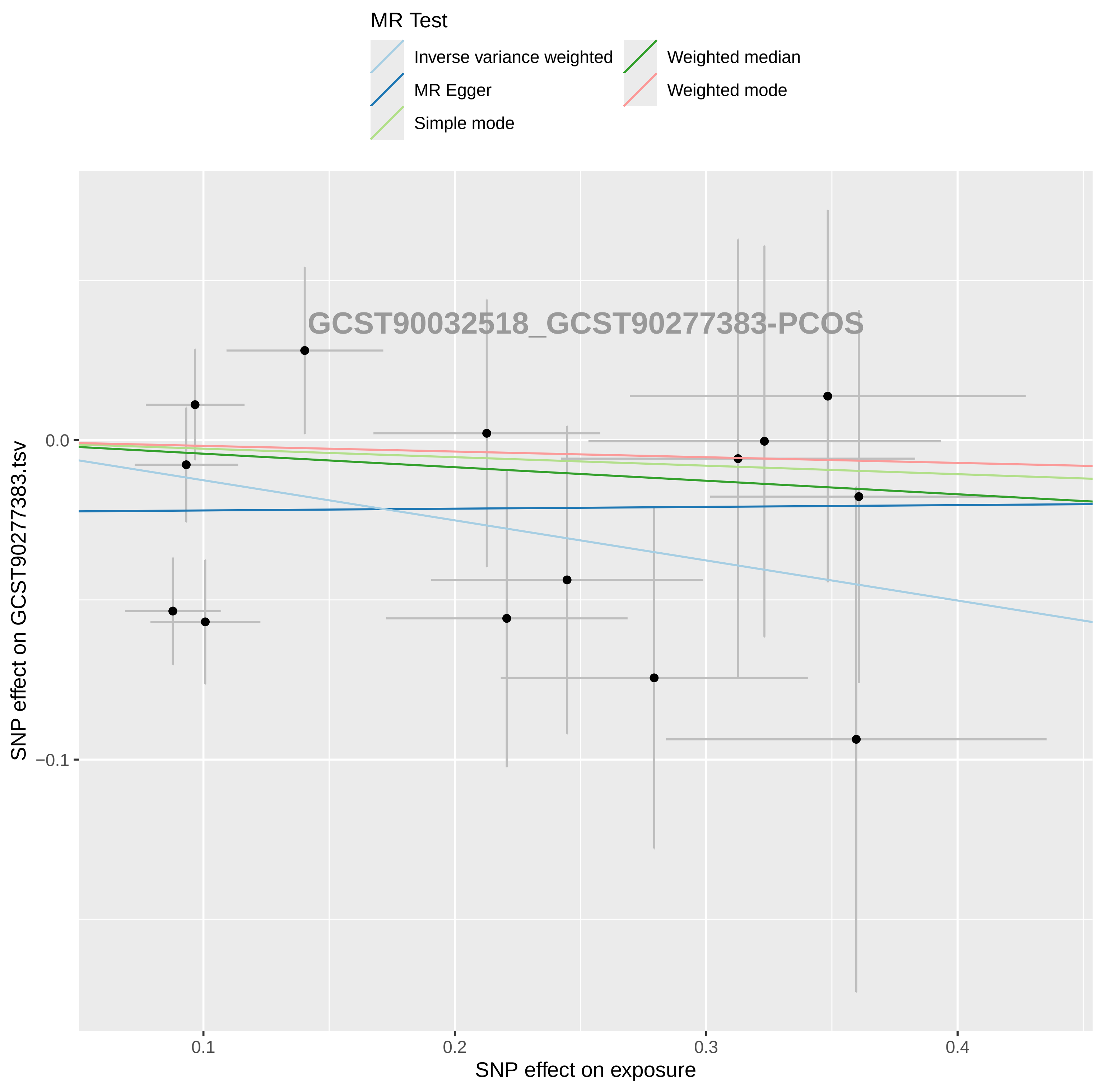

MR Test
Inverse variance weighted
MR Egger
Simple mode
Weighted median
Weighted mode
GCST90032518_GCST90277383-PCOS
SNP effect on GCST90277383.tsv
0.0
−0.1
0.1
0.2
0.3
0.4
SNP effect on exposure

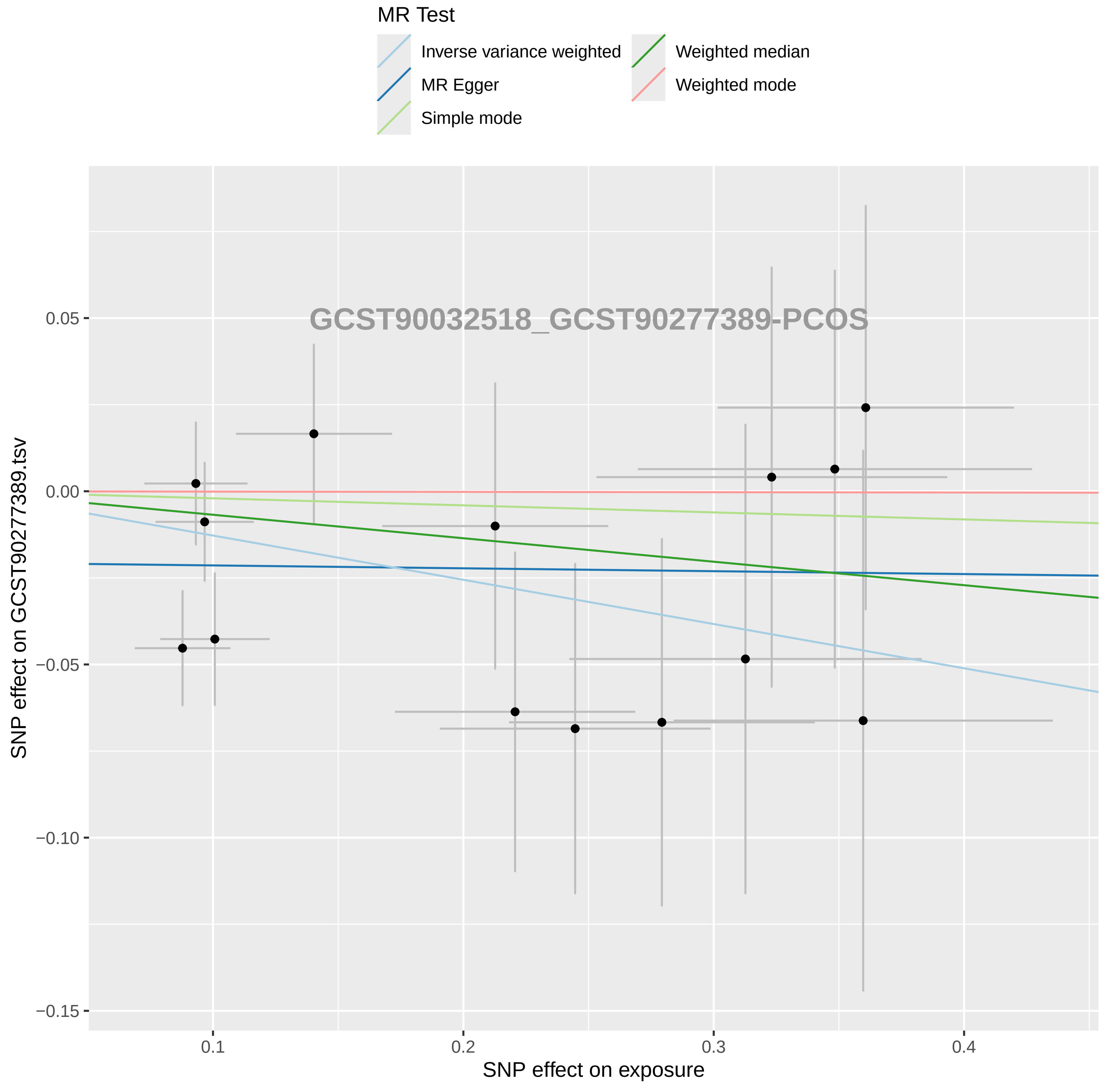
MR Test
Inverse variance weighted
MR Egger
Simple mode
Weighted median
Weighted mode
GCST90032518_GCST90277389-PCOS
0.05
0.00
−0.05
−0.10
−0.15
0.1
0.2
0.3
0.4
SNP effect on GCST90277389.tsv
SNP effect on exposure

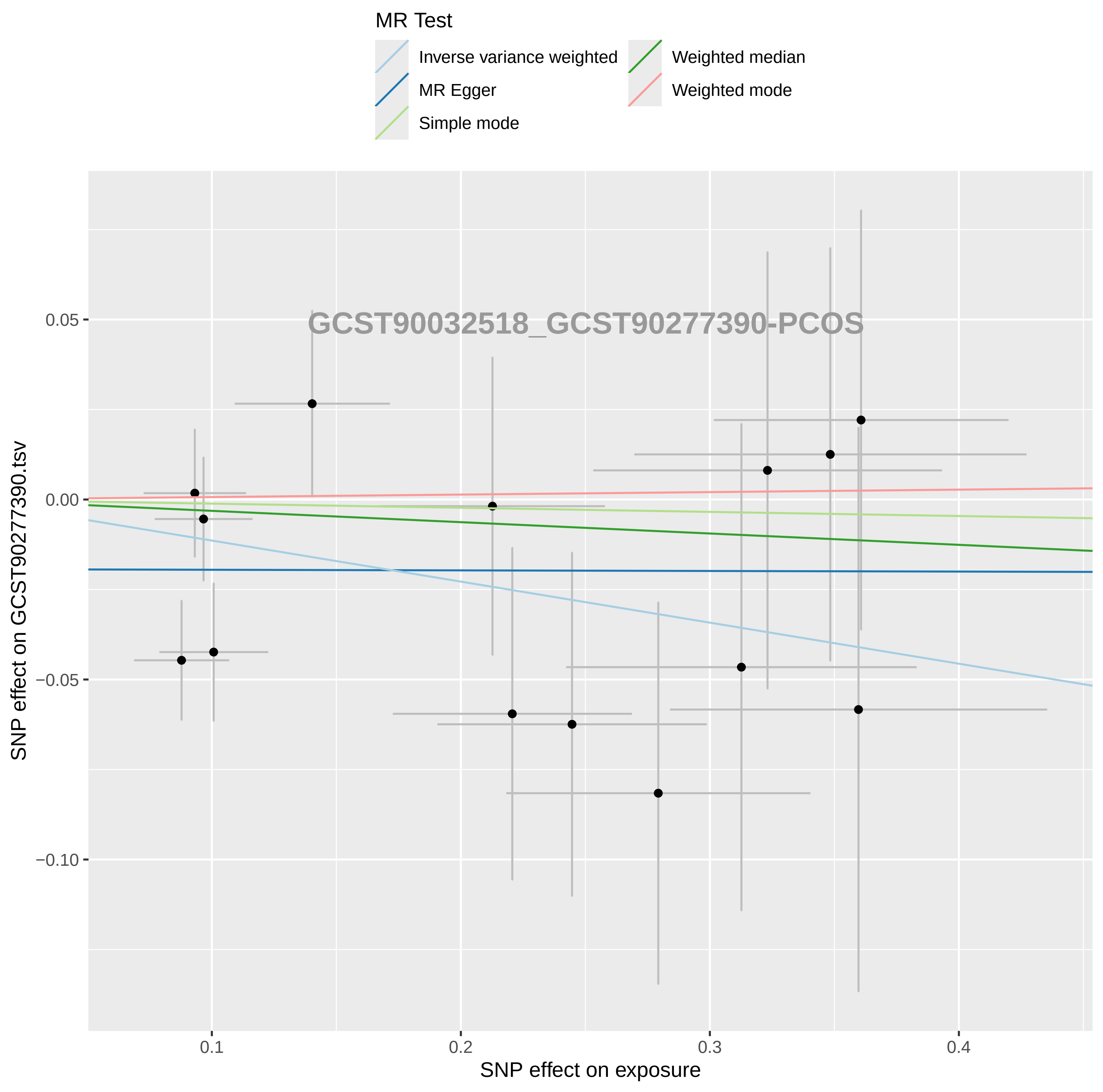

MR Test
Inverse variance weighted
MR Egger
Simple mode
Weighted median
Weighted mode
GCST90032518_GCST90277390-PCOS
0.05
0.00
−0.05
−0.10
0.1
0.2
0.3
0.4
SNP effect on exposure
SNP effect on GCST90277390.tsv

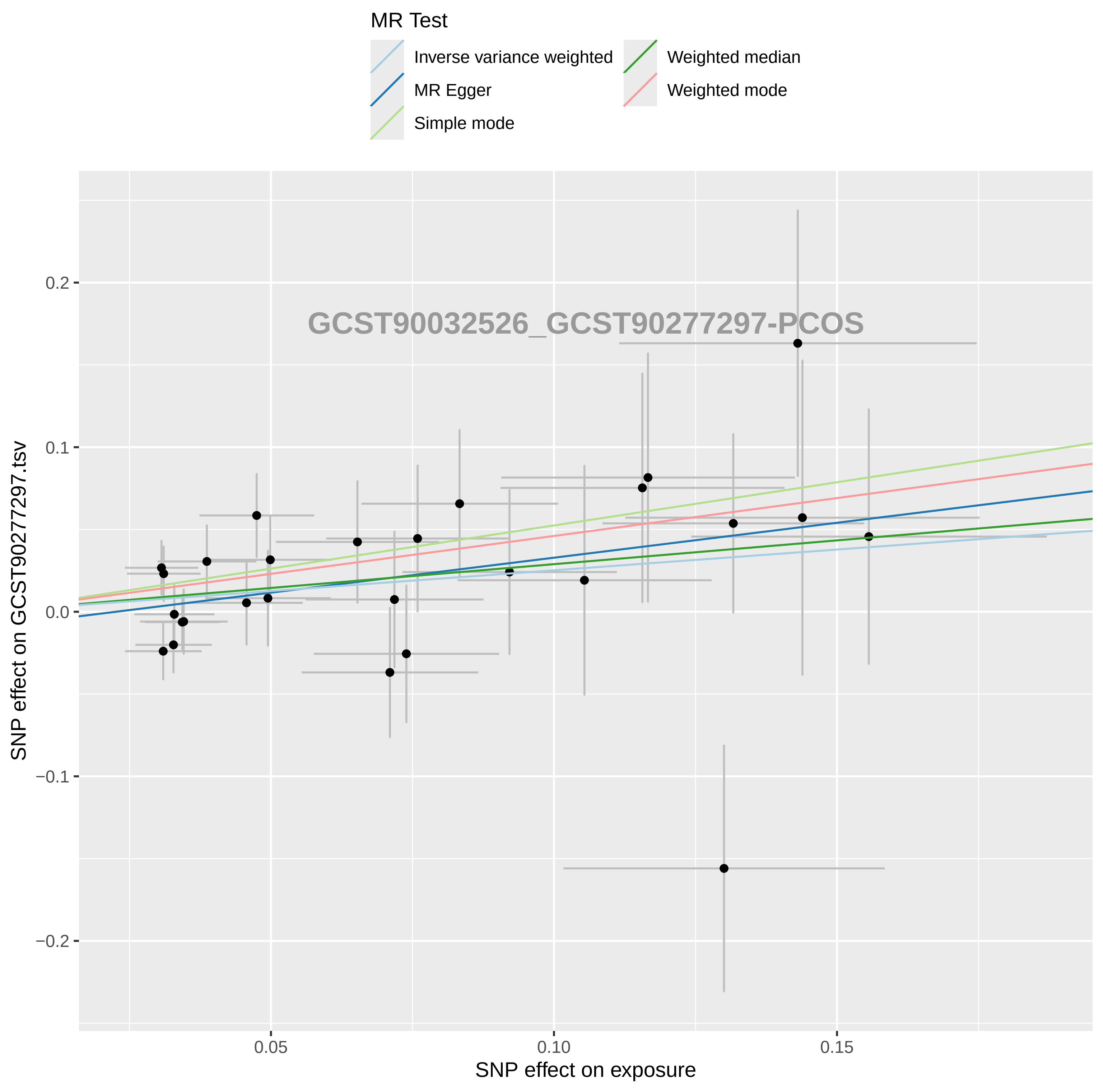

MR Test
Inverse variance weighted
MR Egger
Simple mode
Weighted median
Weighted mode
GCST90032526_GCST90277297-PCOS
SNP effect on GCST90277297.tsv
SNP effect on exposure
0.2
0.1
0.0
−0.1
−0.2
0.05
0.10
0.15

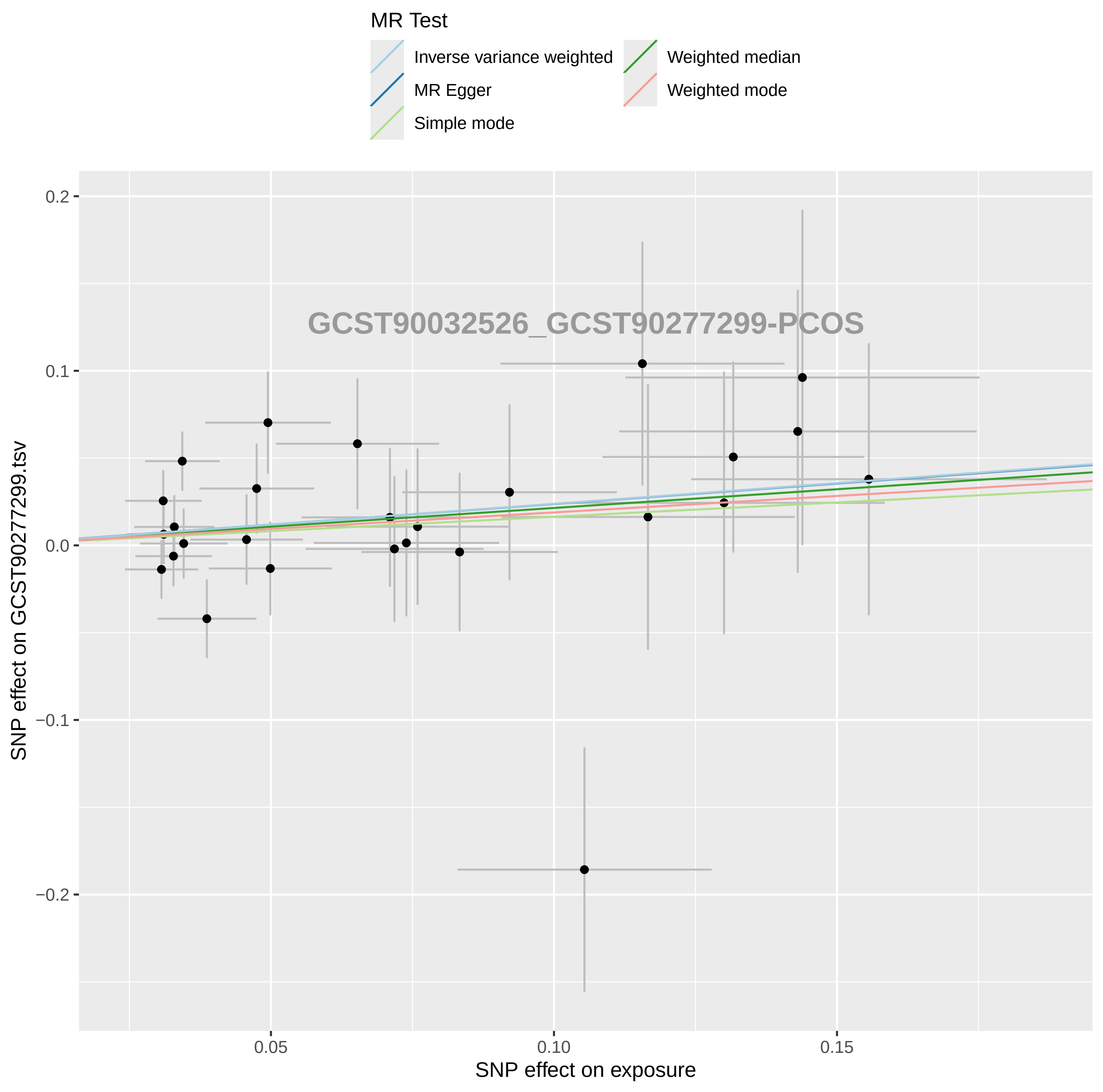

MR Test
Inverse variance weighted
MR Egger
Simple mode
Weighted median
Weighted mode
GCST90032526_GCST90277299-PCOS
SNP effect on GCST90277299.tsv
SNP effect on exposure
0.2
0.1
0.0
−0.1
−0.2
0.05
0.10
0.15

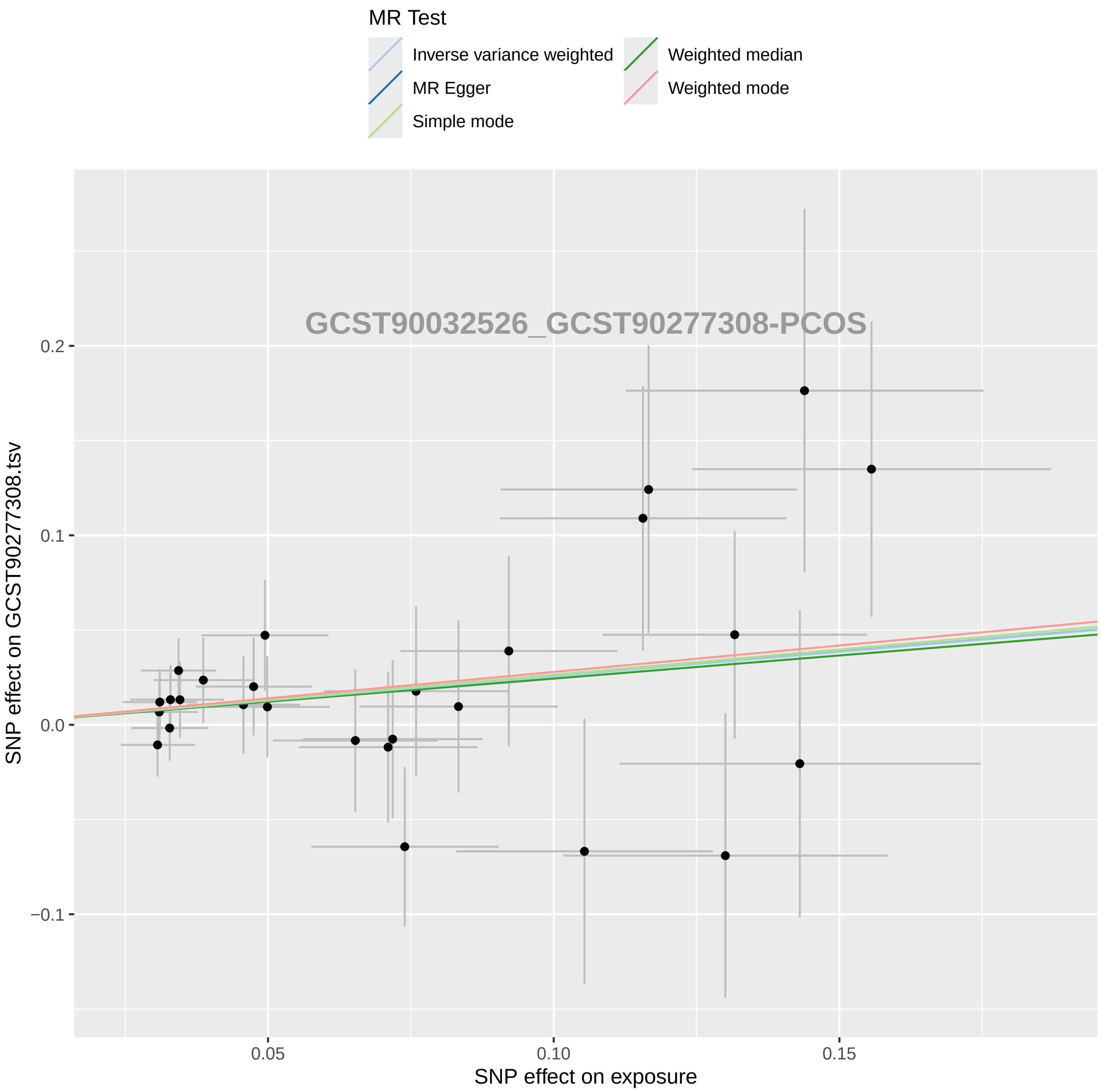

MR Test
Inverse variance weighted
MR Egger
Simple mode
Weighted median
Weighted mode
GCST90032526_GCST90277308-PCOS
SNP effect on GCST90277308.tsv
SNP effect on exposure
0.2
0.1
0.0
−0.1
0.05
0.10
0.15

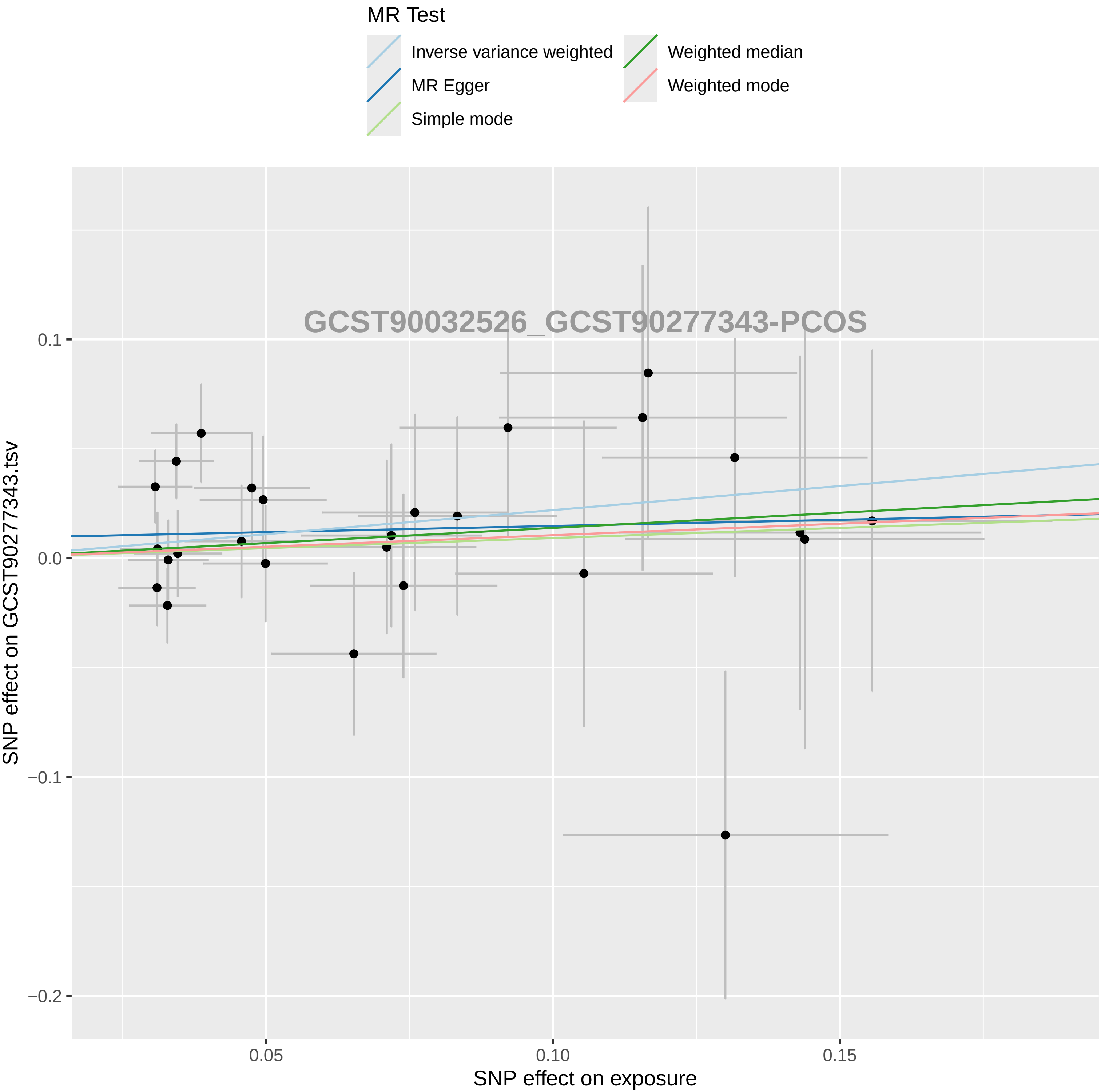
MR Test
Inverse variance weighted
MR Egger
Simple mode
Weighted median
Weighted mode
GCST90032526_GCST90277343-PCOS
0.1
0.0
−0.1
−0.2
0.05
0.10
0.15
SNP effect on GCST90277343.tsv
SNP effect on exposure

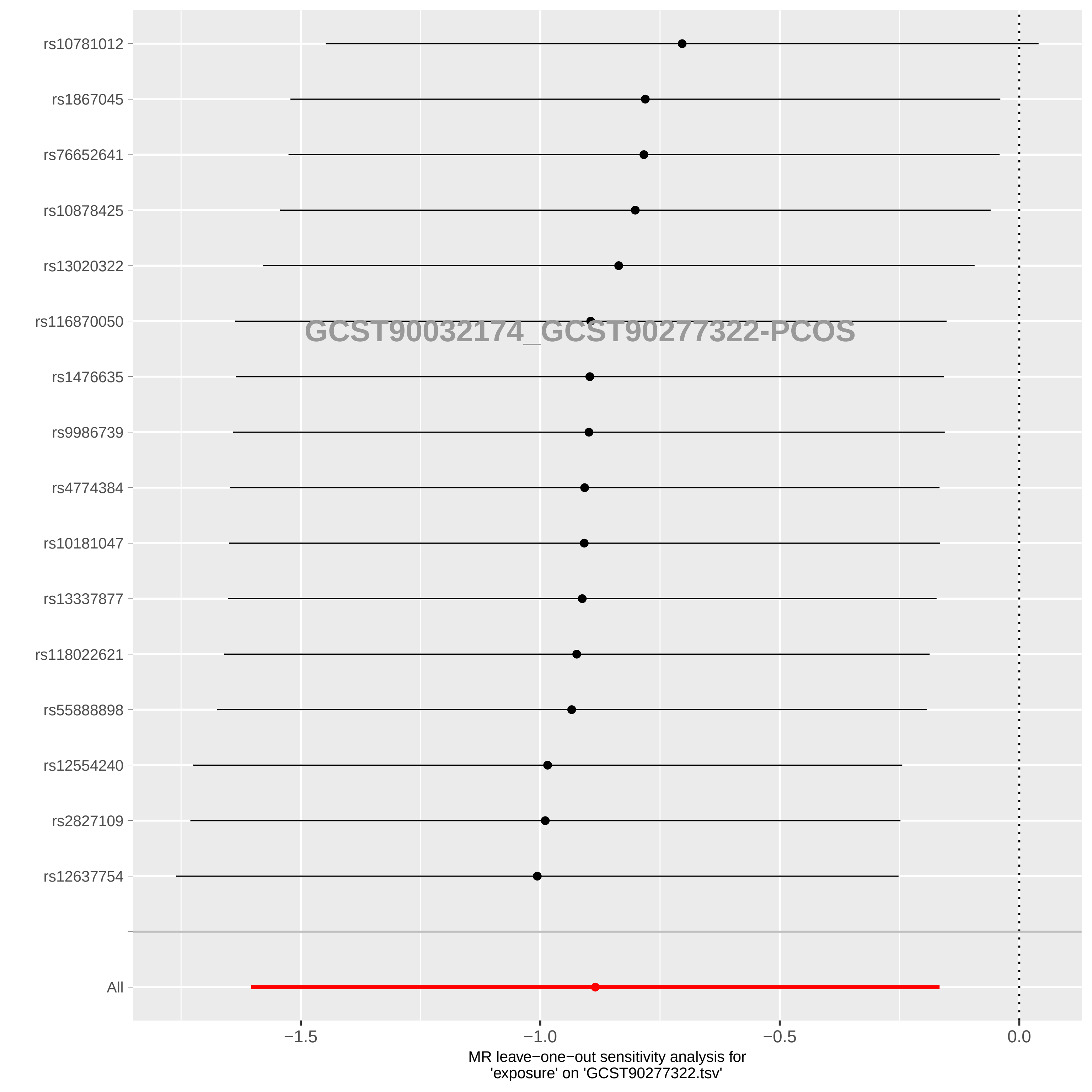

rs10781012
rs1867045
rs76652641
rs10878425
rs13020322
rs116870050
GCST90032174_GCST90277322-PCOS
rs1476635
rs9986739
rs4774384
rs10181047
rs13337877
rs118022621
rs55888898
rs12554240
rs2827109
rs12637754
All
−1.5
−1.0
−0.5
0.0
MR leave−one−out sensitivity analysis for
'exposure' on 'GCST90277322.tsv'

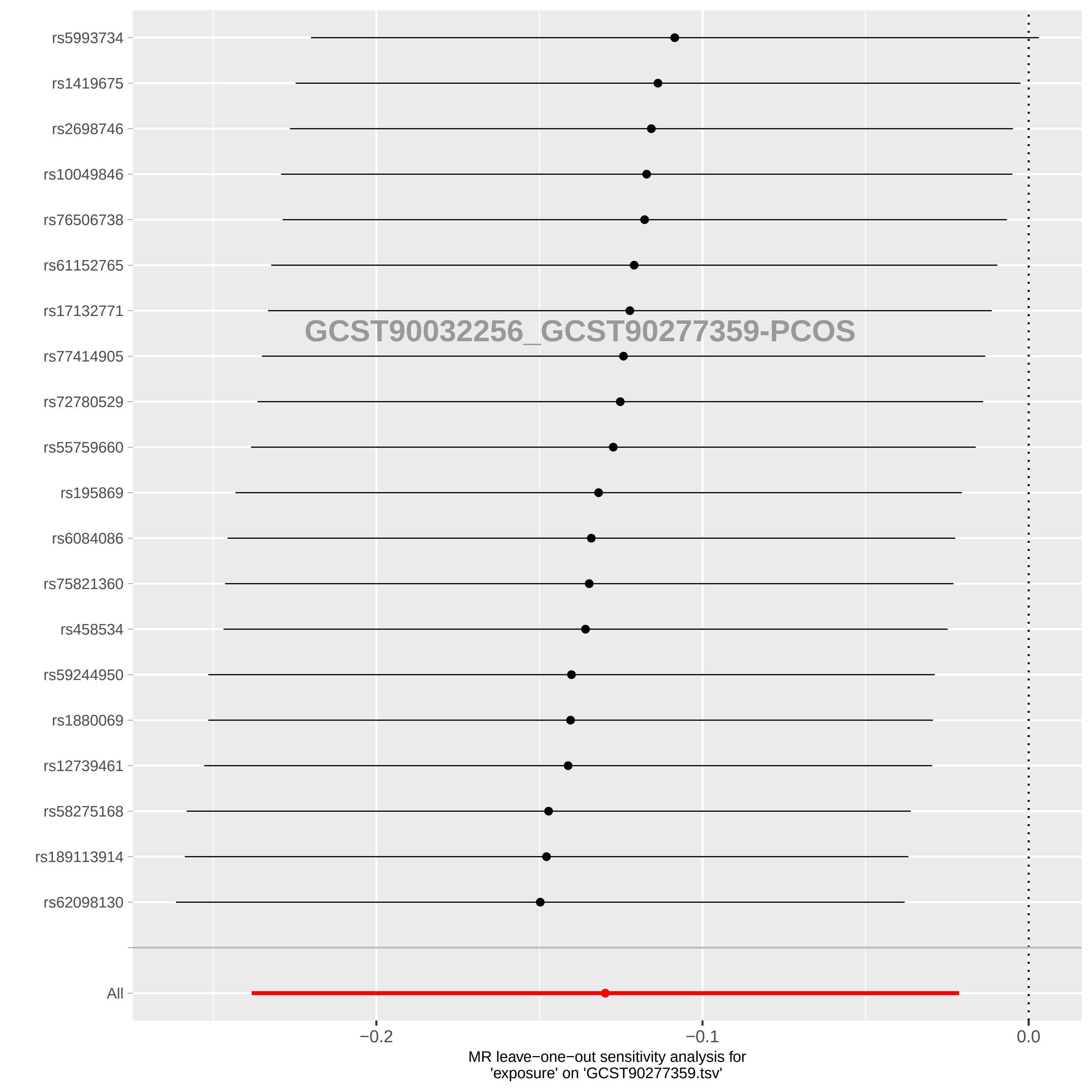

rs5993734
rs1419675
rs2698746
rs10049846
rs76506738
rs61152765
rs17132771
GCST90032256_GCST90277359-PCOS
rs77414905
rs72780529
rs55759660
rs195869
rs6084086
rs75821360
rs458534
rs59244950
rs1880069
rs12739461
rs58275168
rs189113914
rs62098130
All
−0.2
−0.1
0.0
MR leave−one−out sensitivity analysis for
'exposure' on 'GCST90277359.tsv'

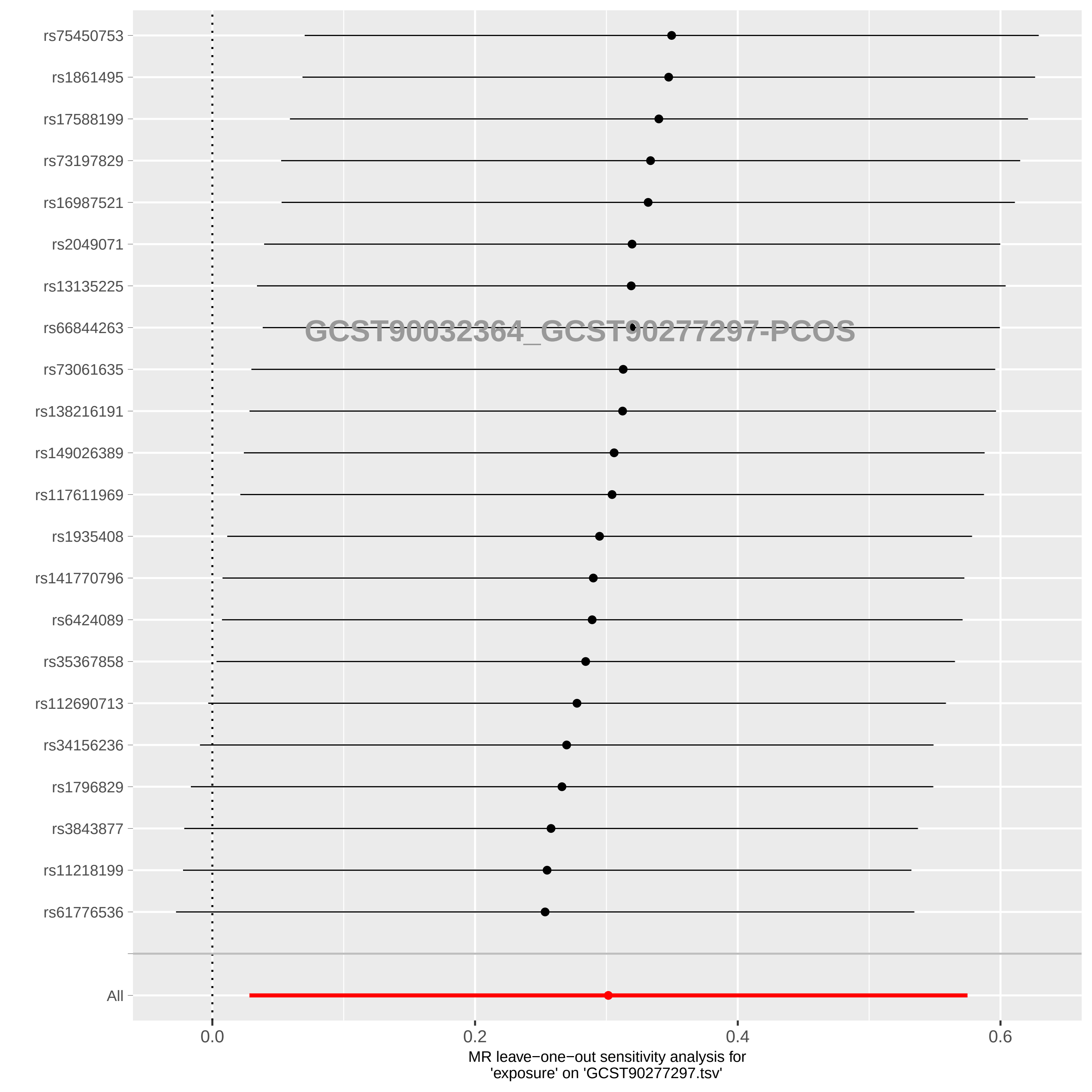

rs75450753
rs1861495
rs17588199
rs73197829
rs16987521
rs2049071
rs13135225
rs66844263
rs73061635
rs138216191
rs149026389
rs117611969
rs1935408
rs141770796
rs6424089
rs35367858
rs112690713
rs34156236
rs1796829
rs3843877
rs11218199
rs61776536
All
GCST90032364_GCST90277297-PCOS
0.0
0.2
0.4
0.6
MR leave−one−out sensitivity analysis for
'exposure' on 'GCST90277297.tsv'

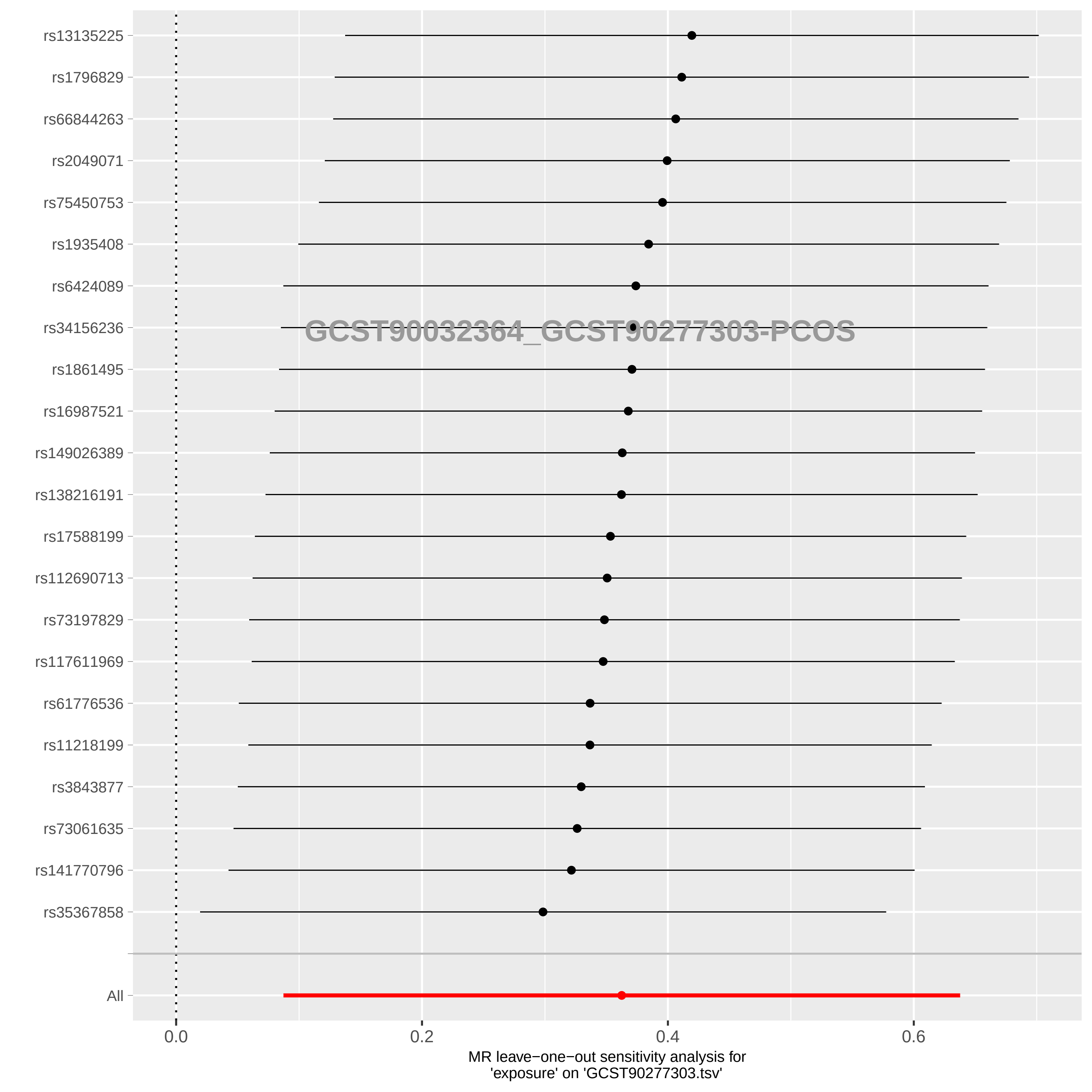

GCST90032364_GCST90277303-PCOS
rs13135225
rs1796829
rs66844263
rs2049071
rs75450753
rs1935408
rs6424089
rs34156236
rs1861495
rs16987521
rs149026389
rs138216191
rs17588199
rs112690713
rs73197829
rs117611969
rs61776536
rs11218199
rs3843877
rs73061635
rs141770796
rs35367858
All
0.0
0.2
0.4
0.6
MR leave−one−out sensitivity analysis for
'exposure' on 'GCST90277303.tsv'

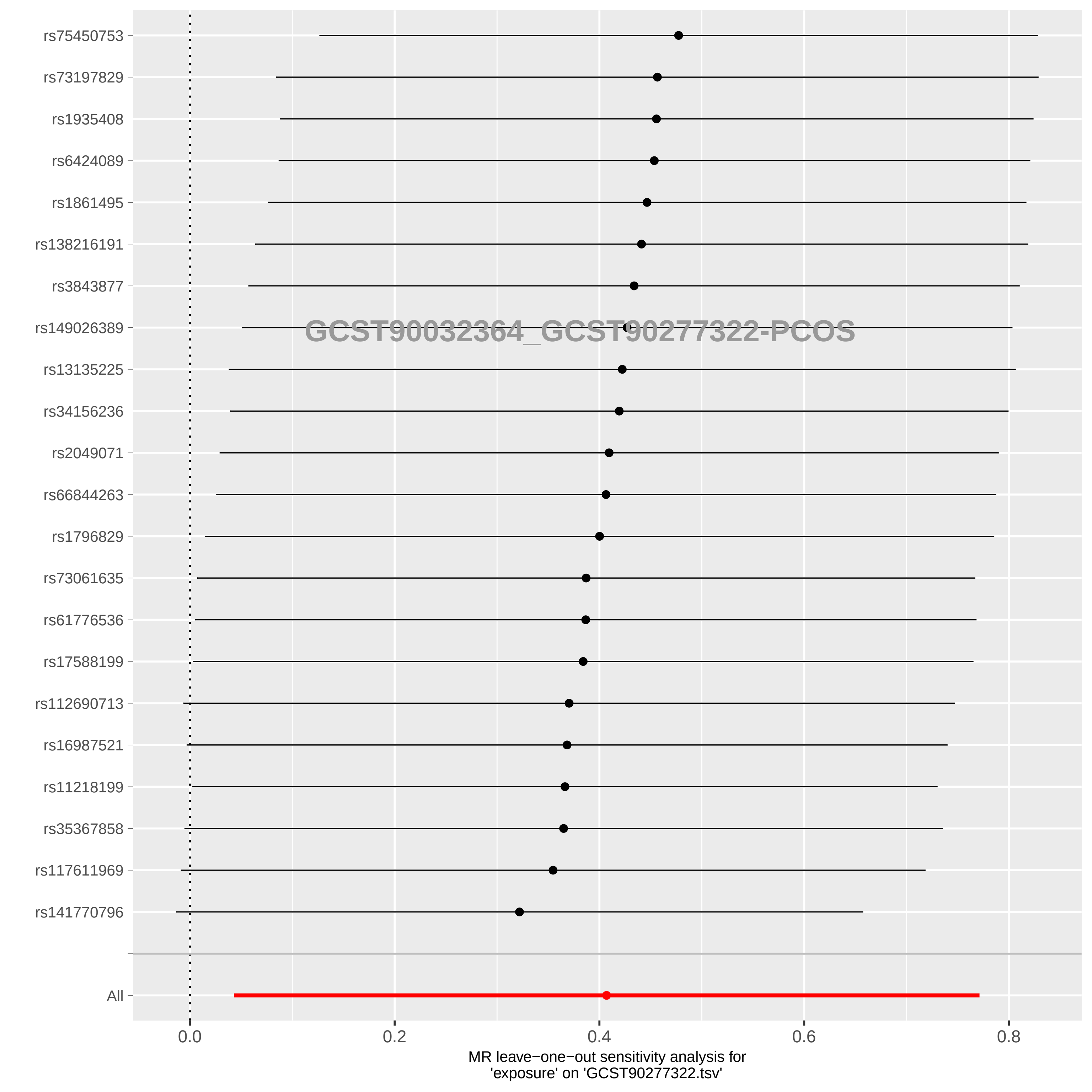

GCST90032364_GCST90277322-PCOS
rs75450753
rs73197829
rs1935408
rs6424089
rs1861495
rs138216191
rs3843877
rs149026389
rs13135225
rs34156236
rs2049071
rs66844263
rs1796829
rs73061635
rs61776536
rs17588199
rs112690713
rs16987521
rs11218199
rs35367858
rs117611969
rs141770796
All
0.0
0.2
0.4
0.6
0.8
MR leave−one−out sensitivity analysis for
'exposure' on 'GCST90277322.tsv'

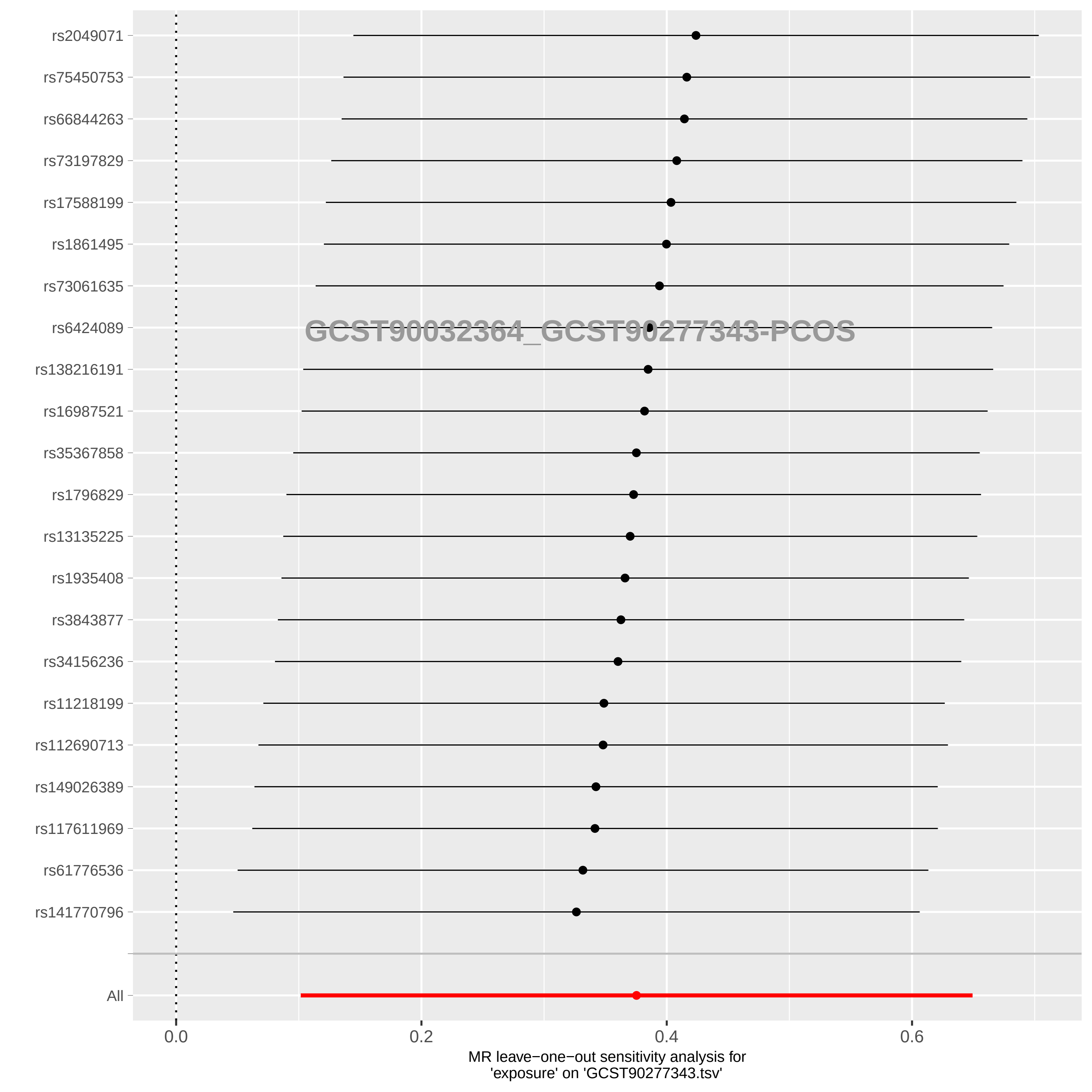
GCST90032364_GCST90277343-PCOS
rs2049071
rs75450753
rs66844263
rs73197829
rs17588199
rs1861495
rs73061635
rs6424089
rs138216191
rs16987521
rs35367858
rs1796829
rs13135225
rs1935408
rs3843877
rs34156236
rs11218199
rs112690713
rs149026389
rs117611969
rs61776536
rs141770796
All
0.0
0.2
0.4
0.6
MR leave−one−out sensitivity analysis for
'exposure' on 'GCST90277343.tsv'

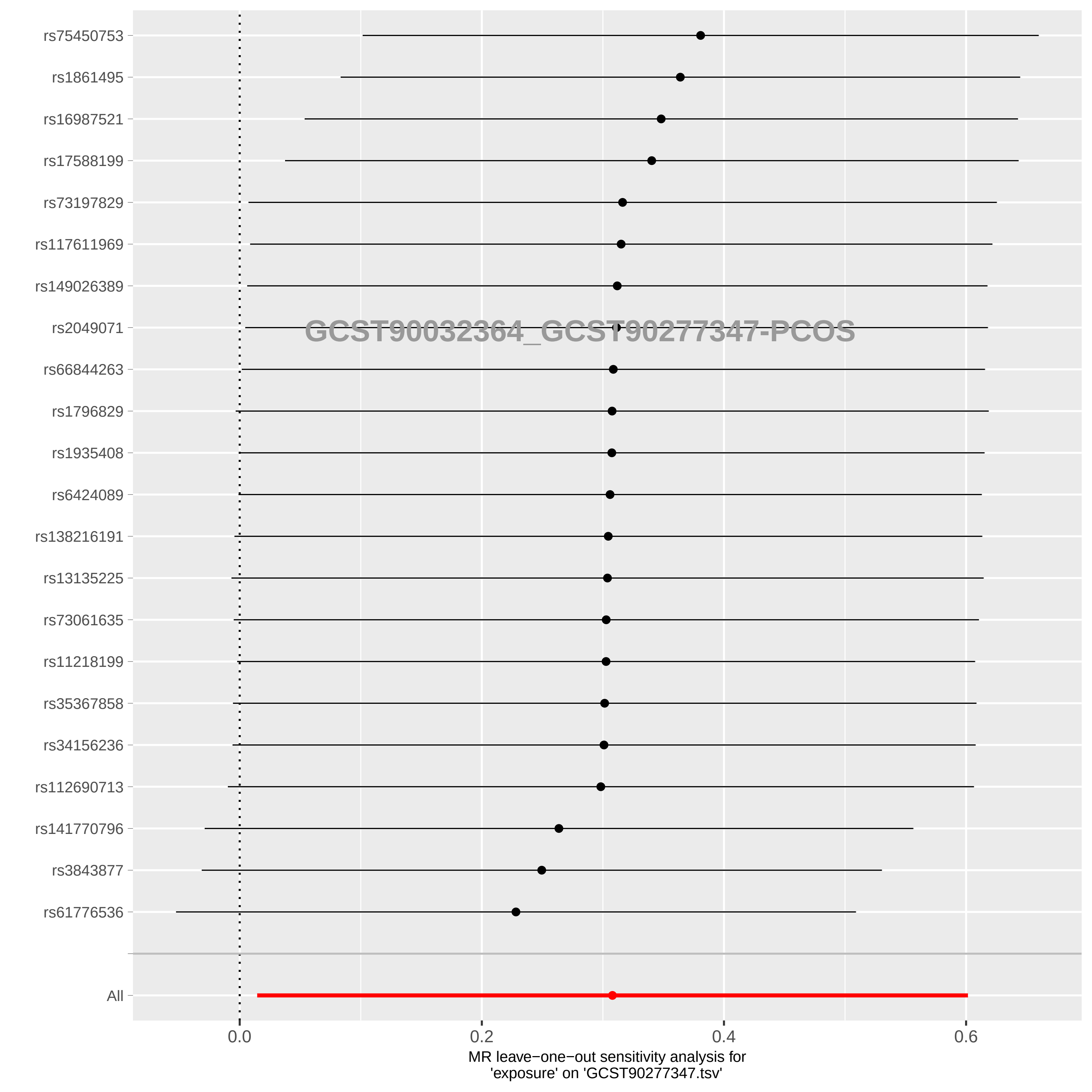

GCST90032364_GCST90277347-PCOS
rs75450753
rs1861495
rs16987521
rs17588199
rs73197829
rs117611969
rs149026389
rs2049071
rs66844263
rs1796829
rs1935408
rs6424089
rs138216191
rs13135225
rs73061635
rs11218199
rs35367858
rs34156236
rs112690713
rs141770796
rs3843877
rs61776536
All
0.0
0.2
0.4
0.6
MR leave−one−out sensitivity analysis for
'exposure' on 'GCST90277347.tsv'

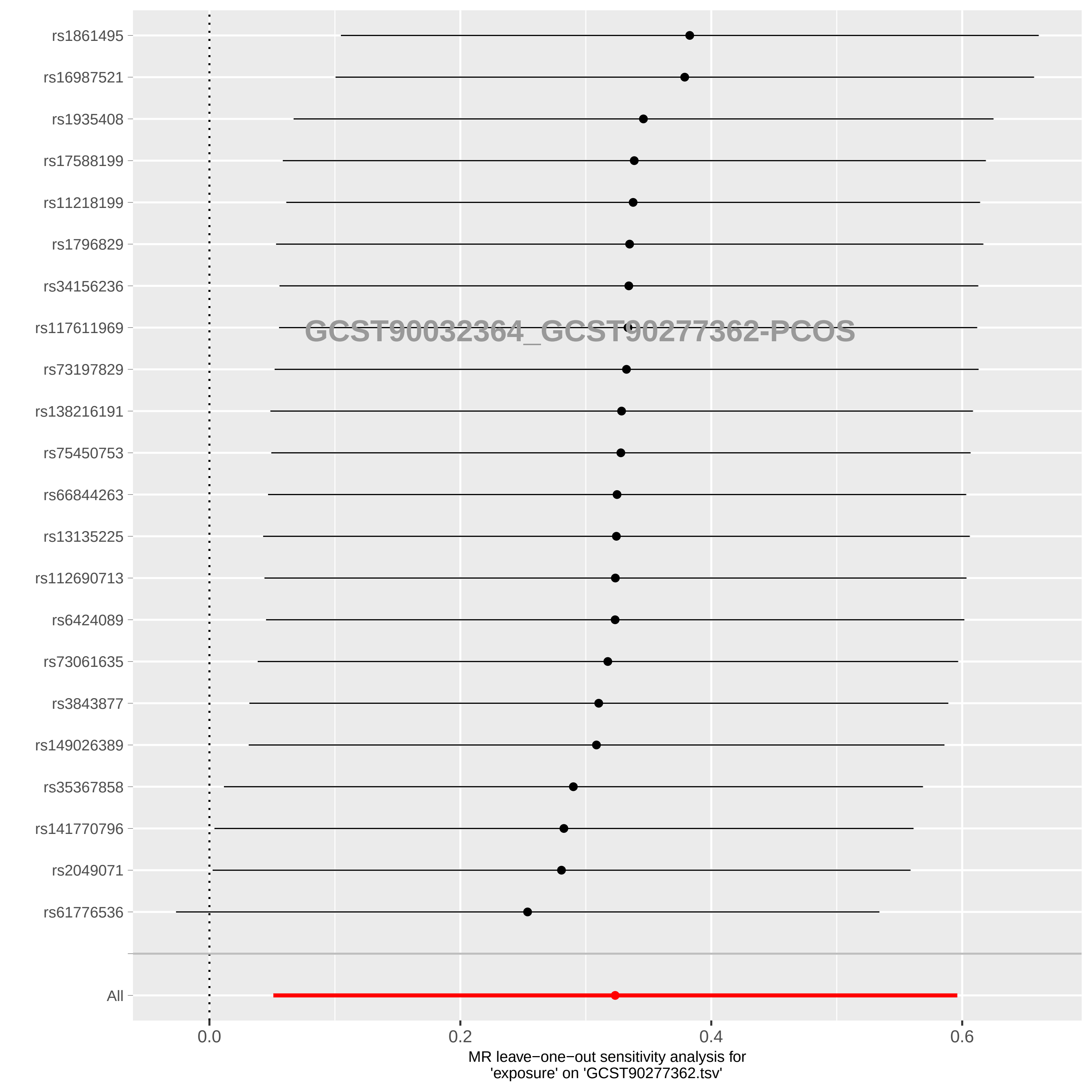
GCST90032364_GCST90277362-PCOS
rs1861495
rs16987521
rs1935408
rs17588199
rs11218199
rs1796829
rs34156236
rs117611969
rs73197829
rs138216191
rs75450753
rs66844263
rs13135225
rs112690713
rs6424089
rs73061635
rs3843877
rs149026389
rs35367858
rs141770796
rs2049071
rs61776536
All
0.0
0.2
0.4
0.6
MR leave−one−out sensitivity analysis for
'exposure' on 'GCST90277362.tsv'

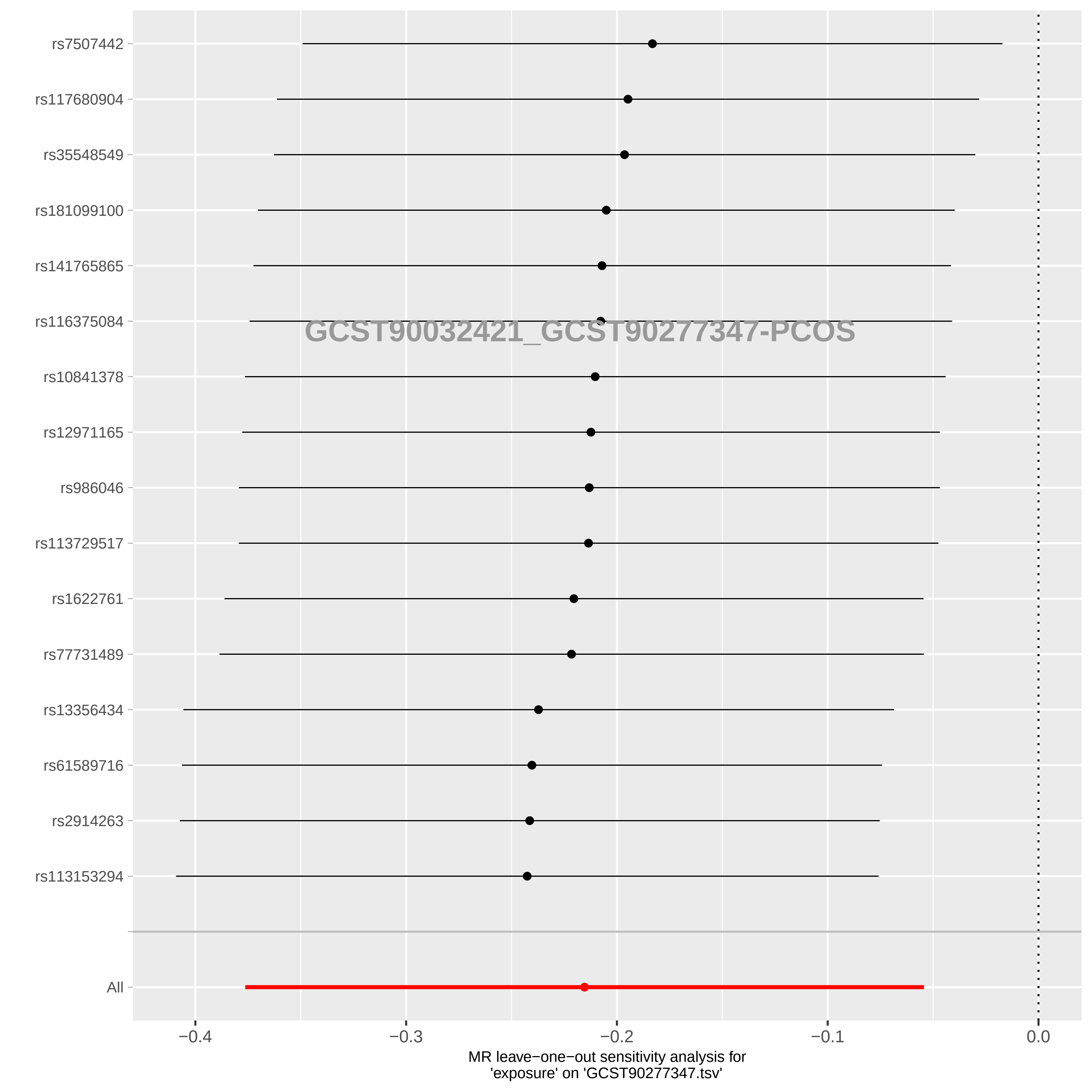

rs7507442
rs117680904
rs35548549
rs181099100
rs141765865
rs116375084
GCST90032421_GCST90277347-PCOS
rs10841378
rs12971165
rs986046
rs113729517
rs1622761
rs77731489
rs13356434
rs61589716
rs2914263
rs113153294
All
−0.4
−0.3
−0.2
−0.1
0.0
MR leave−one−out sensitivity analysis for
'exposure' on 'GCST90277347.tsv'

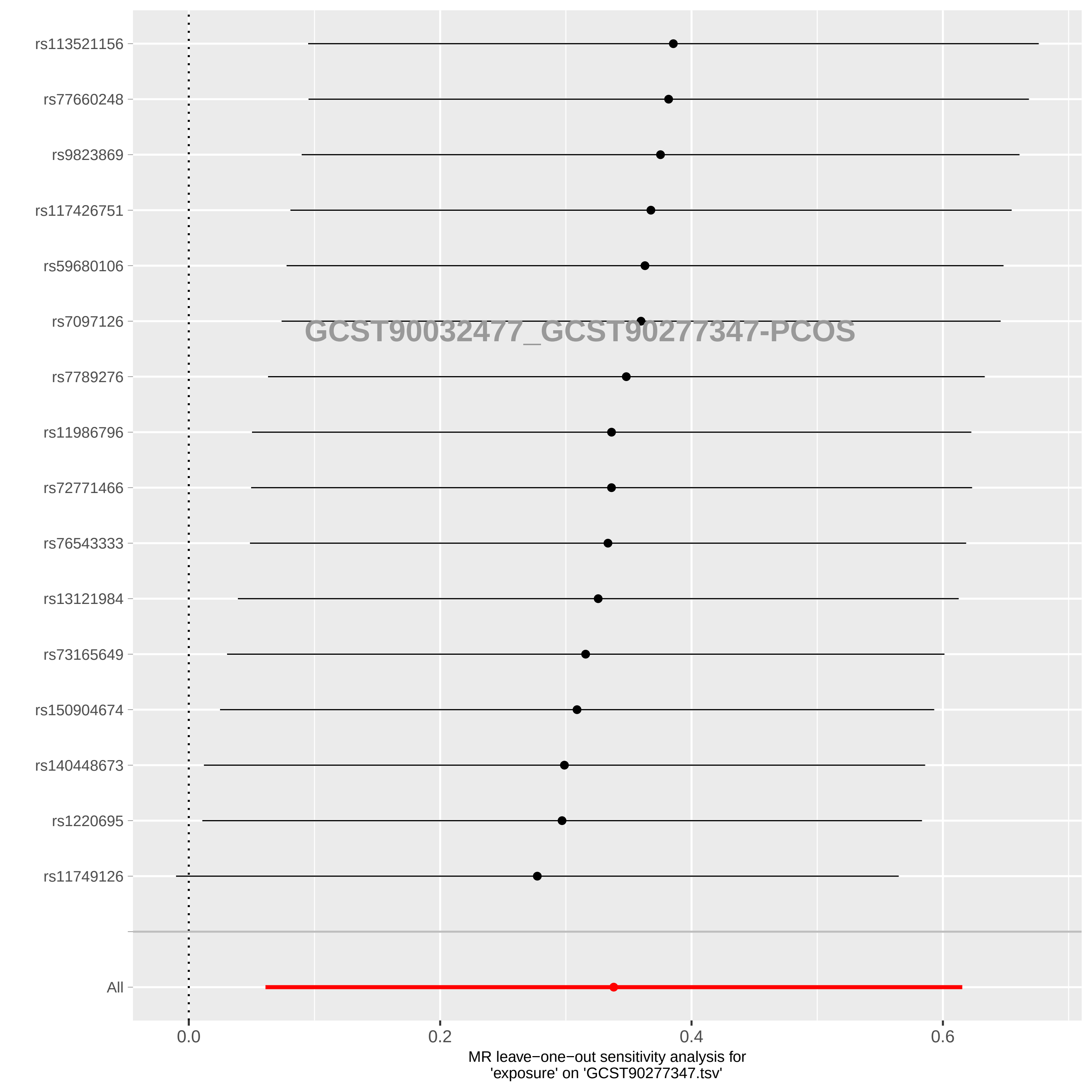

rs113521156
rs77660248
rs9823869
rs117426751
rs59680106
rs7097126
rs7789276
rs11986796
rs72771466
rs76543333
rs13121984
rs73165649
rs150904674
rs140448673
rs1220695
rs11749126
All
GCST90032477_GCST90277347-PCOS
0.0
0.2
0.4
0.6
MR leave−one−out sensitivity analysis for
'exposure' on 'GCST90277347.tsv'

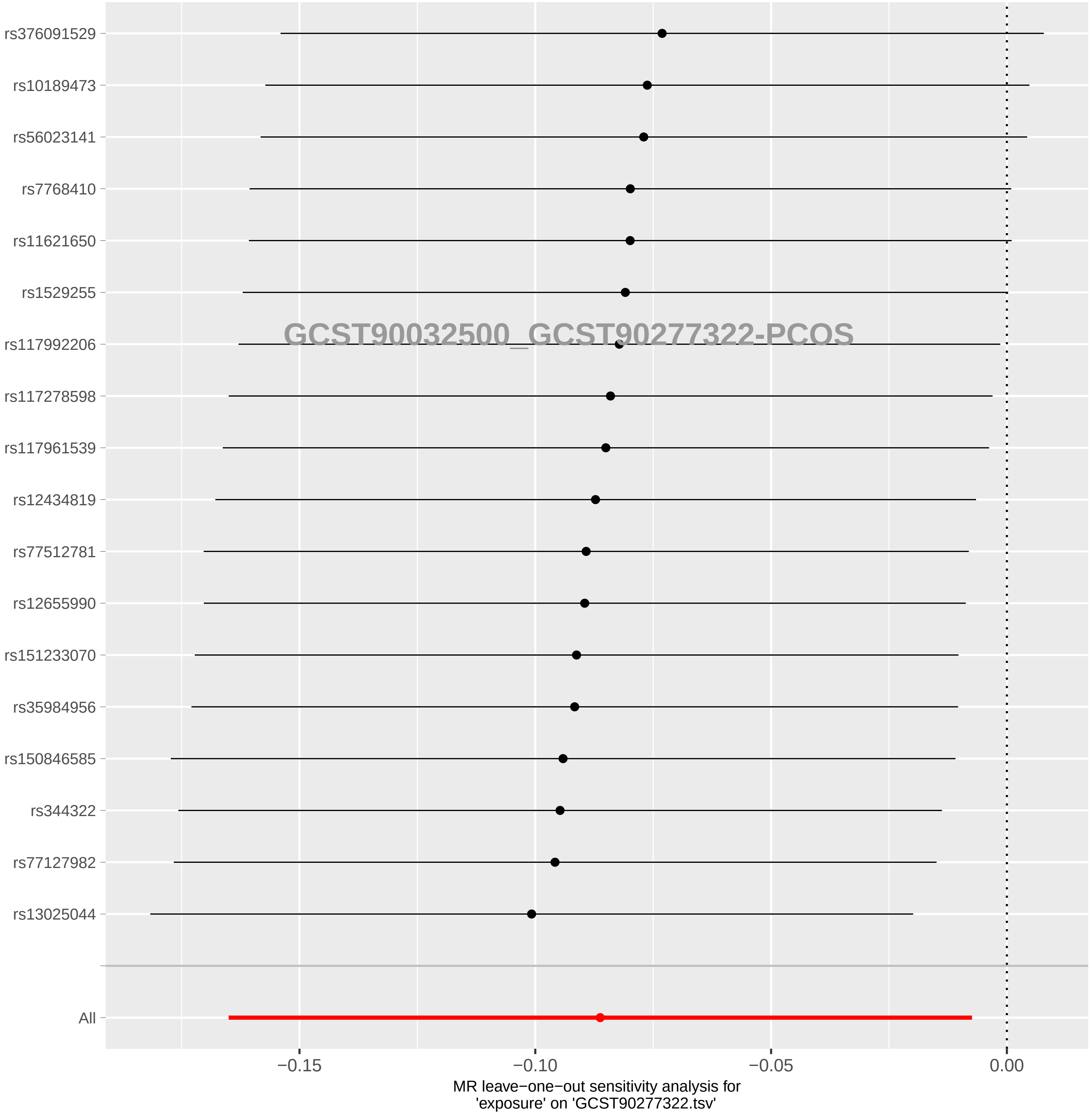

GCST90032500_GCST90277322-PCOS
rs376091529
rs10189473
rs56023141
rs7768410
rs11621650
rs1529255
rs117992206
rs117278598
rs117961539
rs12434819
rs77512781
rs12655990
rs151233070
rs35984956
rs150846585
rs344322
rs77127982
rs13025044
All
−0.15
−0.10
−0.05
0.00
MR leave−one−out sensitivity analysis for
'exposure' on 'GCST90277322.tsv'

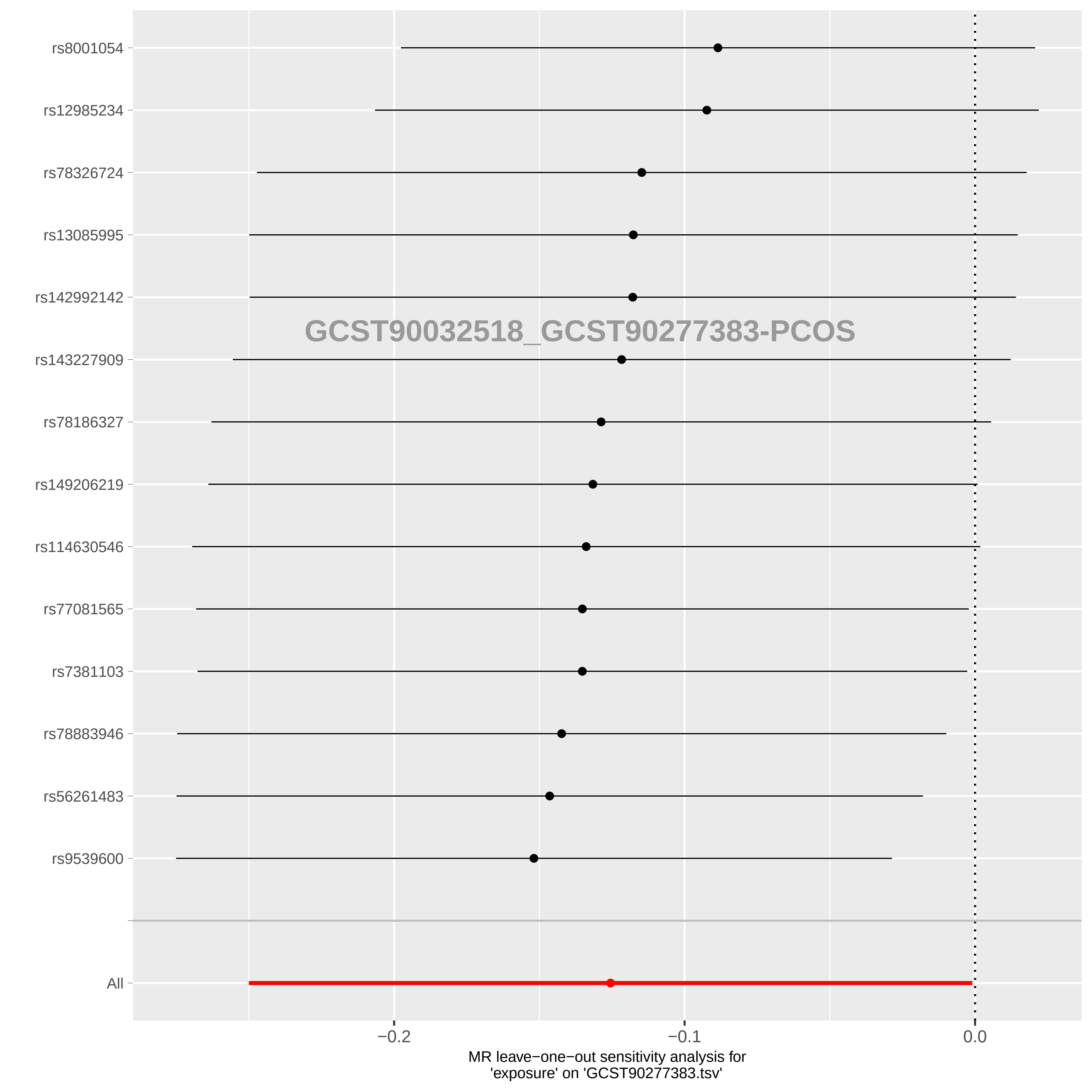

GCST90032518_GCST90277383-PCOS
rs8001054
rs12985234
rs78326724
rs13085995
rs142992142
rs143227909
rs78186327
rs149206219
rs114630546
rs77081565
rs7381103
rs78883946
rs56261483
rs9539600
All
−0.2
−0.1
0.0
MR leave−one−out sensitivity analysis for
'exposure' on 'GCST90277383.tsv'

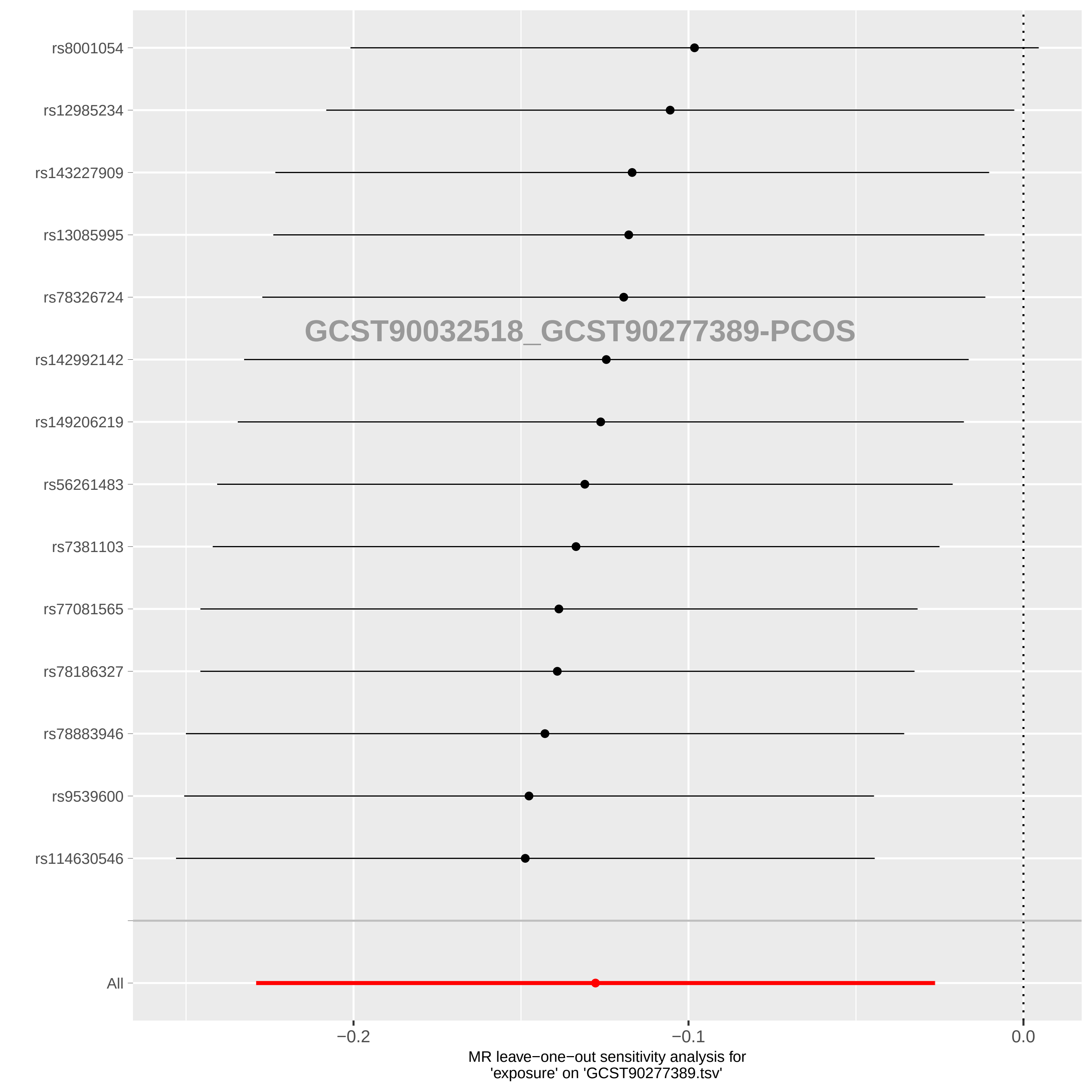
GCST90032518_GCST90277389-PCOS
rs8001054
rs12985234
rs143227909
rs13085995
rs78326724
rs142992142
rs149206219
rs56261483
rs7381103
rs77081565
rs78186327
rs78883946
rs9539600
rs114630546
All
−0.2
−0.1
0.0
MR leave−one−out sensitivity analysis for
'exposure' on 'GCST90277389.tsv'

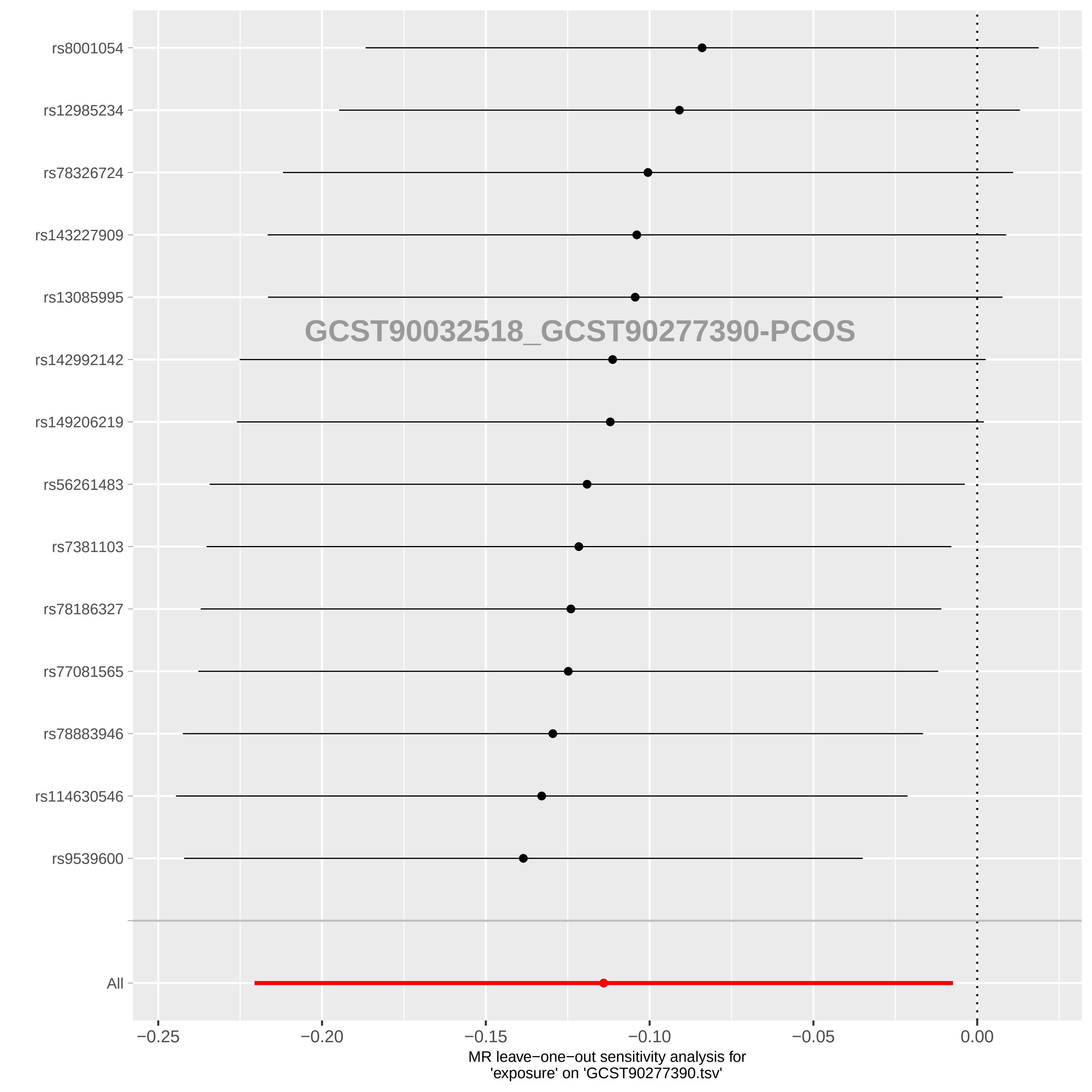
GCST90032518_GCST90277390-PCOS
rs8001054
rs12985234
rs78326724
rs143227909
rs13085995
rs142992142
rs149206219
rs56261483
rs7381103
rs78186327
rs77081565
rs78883946
rs114630546
rs9539600
All
−0.25
−0.20
−0.15
−0.10
−0.05
0.00
MR leave−one−out sensitivity analysis for
'exposure' on 'GCST90277390.tsv'

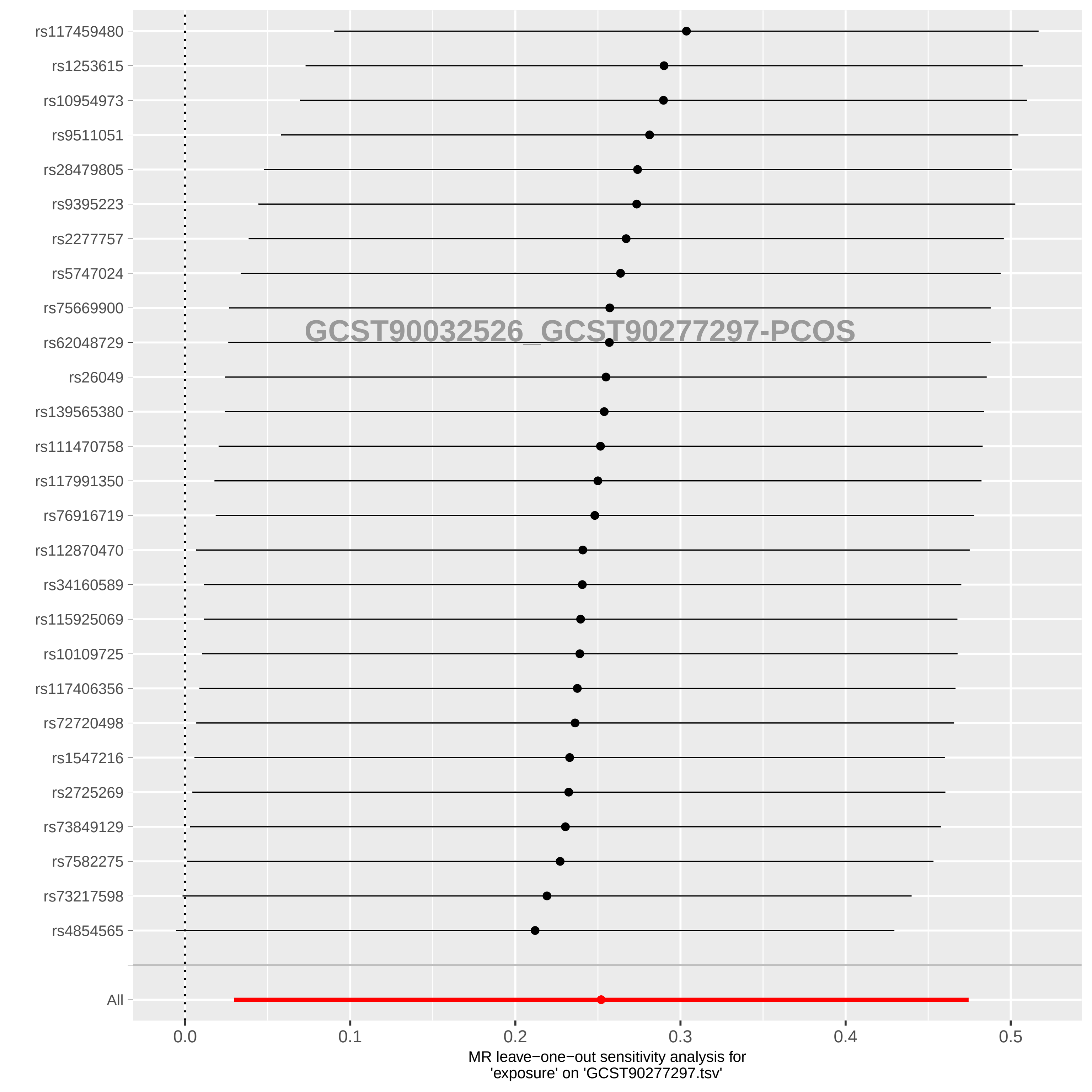
GCST90032526_GCST90277297-PCOS
rs117459480
rs1253615
rs10954973
rs9511051
rs28479805
rs9395223
rs2277757
rs5747024
rs75669900
rs62048729
rs26049
rs139565380
rs111470758
rs117991350
rs76916719
rs112870470
rs34160589
rs115925069
rs10109725
rs117406356
rs72720498
rs1547216
rs2725269
rs73849129
rs7582275
rs73217598
rs4854565
All
0.0
0.1
0.2
0.3
0.4
0.5
MR leave−one−out sensitivity analysis for
'exposure' on 'GCST90277297.tsv'

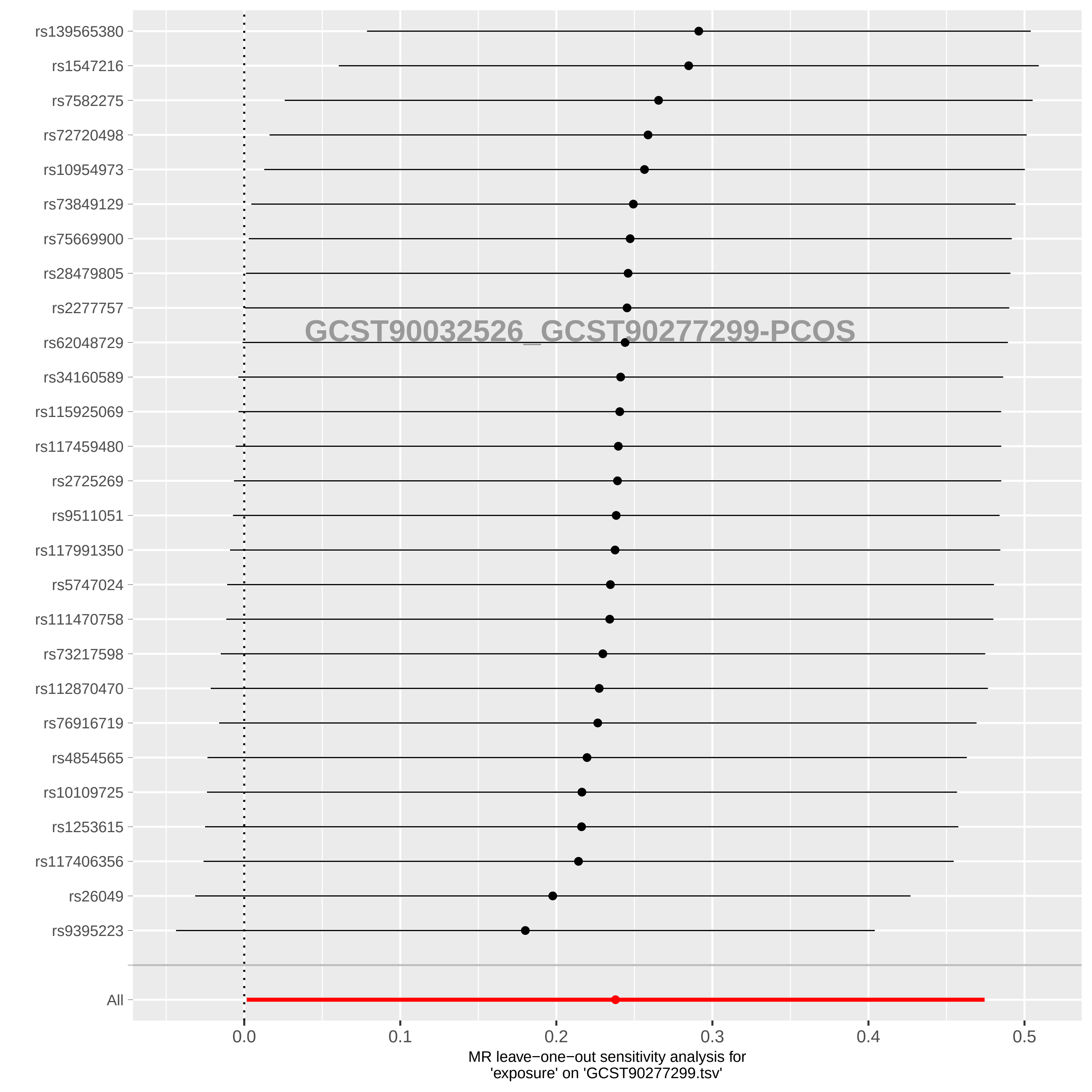
GCST90032526_GCST90277299-PCOS
rs139565380
rs1547216
rs7582275
rs72720498
rs10954973
rs73849129
rs75669900
rs28479805
rs2277757
rs62048729
rs34160589
rs115925069
rs117459480
rs2725269
rs9511051
rs117991350
rs5747024
rs111470758
rs73217598
rs112870470
rs76916719
rs4854565
rs10109725
rs1253615
rs117406356
rs26049
rs9395223
All
0.0
0.1
0.2
0.3
0.4
0.5
MR leave−one−out sensitivity analysis for
'exposure' on 'GCST90277299.tsv'

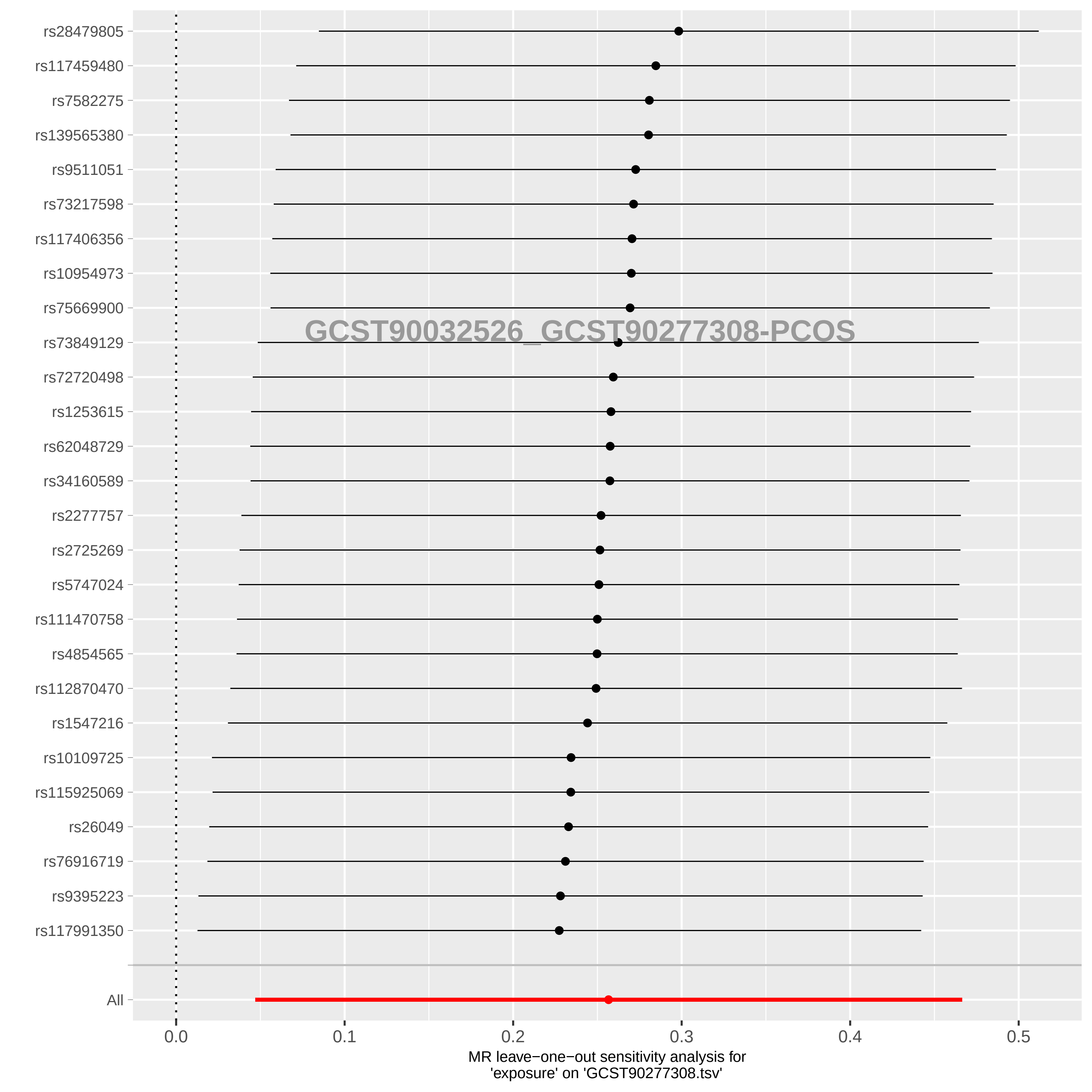

GCST90032526_GCST90277308-PCOS
rs28479805
rs117459480
rs7582275
rs139565380
rs9511051
rs73217598
rs117406356
rs10954973
rs75669900
rs73849129
rs72720498
rs1253615
rs62048729
rs34160589
rs2277757
rs2725269
rs5747024
rs111470758
rs4854565
rs112870470
rs1547216
rs10109725
rs115925069
rs26049
rs76916719
rs9395223
rs117991350
All
0.0
0.1
0.2
0.3
0.4
0.5
MR leave−one−out sensitivity analysis for
'exposure' on 'GCST90277308.tsv'

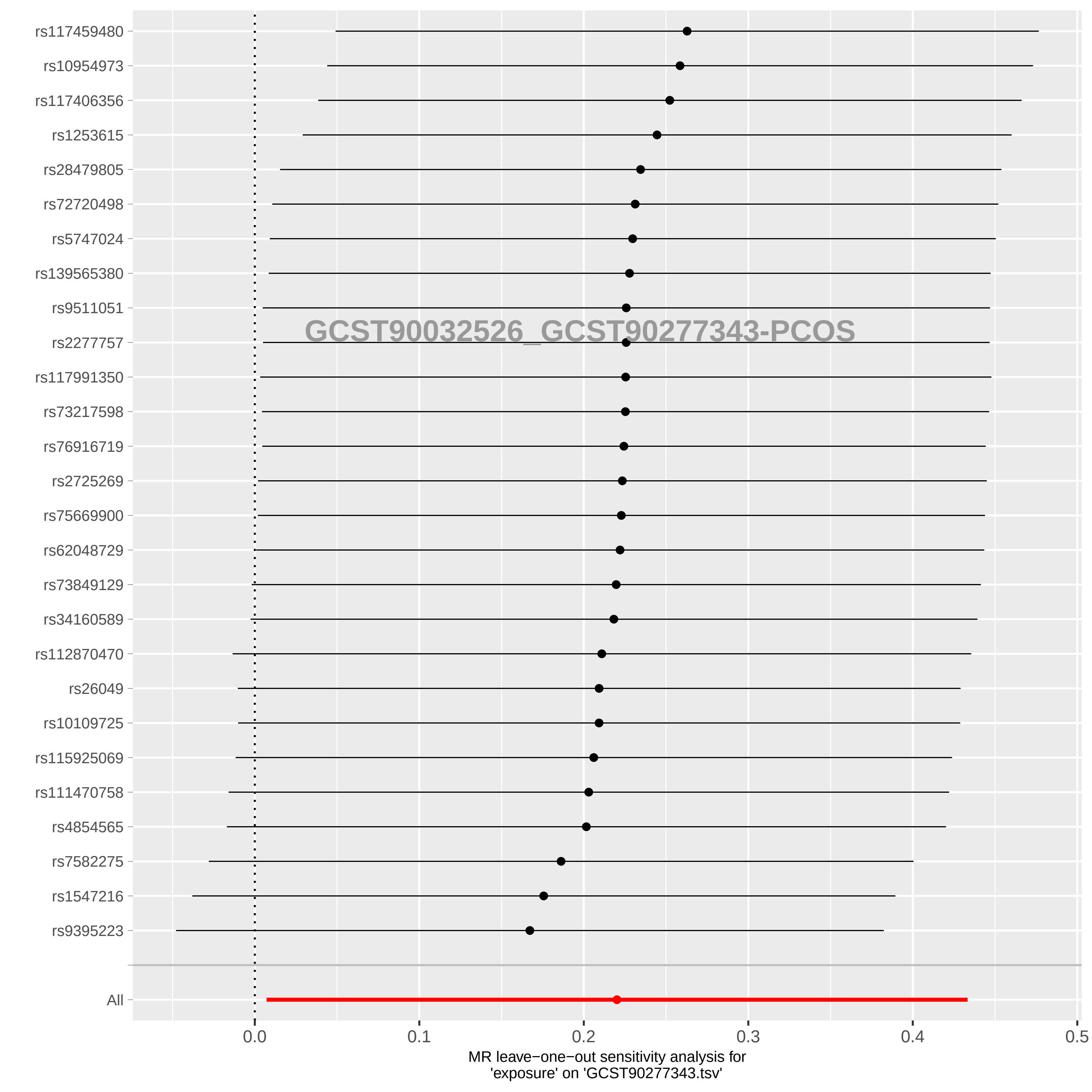

GCST90032526_GCST90277343-PCOS
rs117459480
rs10954973
rs117406356
rs1253615
rs28479805
rs72720498
rs5747024
rs139565380
rs9511051
rs2277757
rs117991350
rs73217598
rs76916719
rs2725269
rs75669900
rs62048729
rs73849129
rs34160589
rs112870470
rs26049
rs10109725
rs115925069
rs111470758
rs4854565
rs7582275
rs1547216
rs9395223
All
0.0
0.1
0.2
0.3
0.4
0.5
MR leave−one−out sensitivity analysis for
'exposure' on 'GCST90277343.tsv'

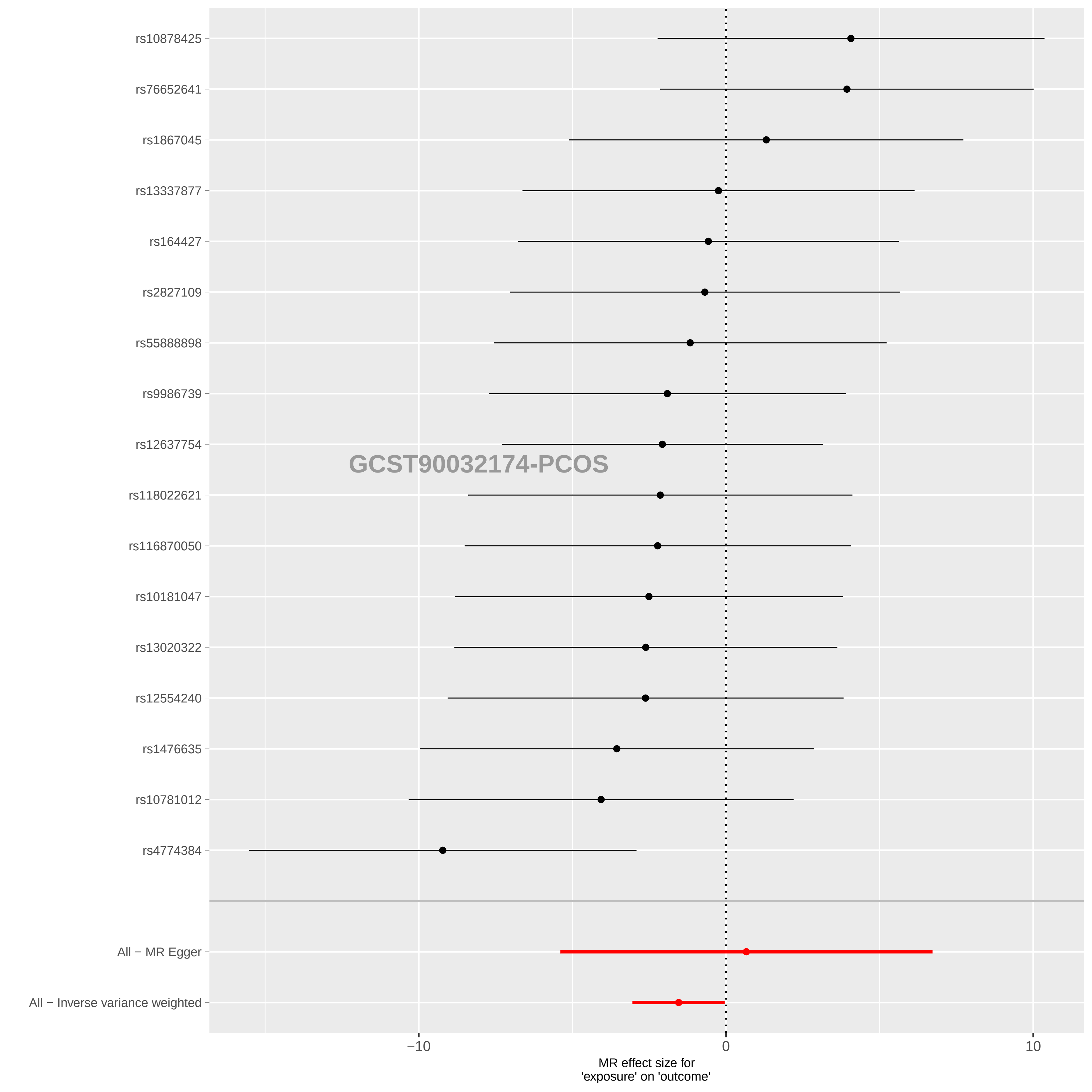
rs10878425
rs76652641
rs1867045
rs13337877
rs164427
rs2827109
rs55888898
rs9986739
rs12637754
GCST90032174-PCOS
rs118022621
rs116870050
rs10181047
rs13020322
rs12554240
rs1476635
rs10781012
rs4774384
All − MR Egger
All − Inverse variance weighted
−10
0
10
MR effect size for
'exposure' on 'outcome'

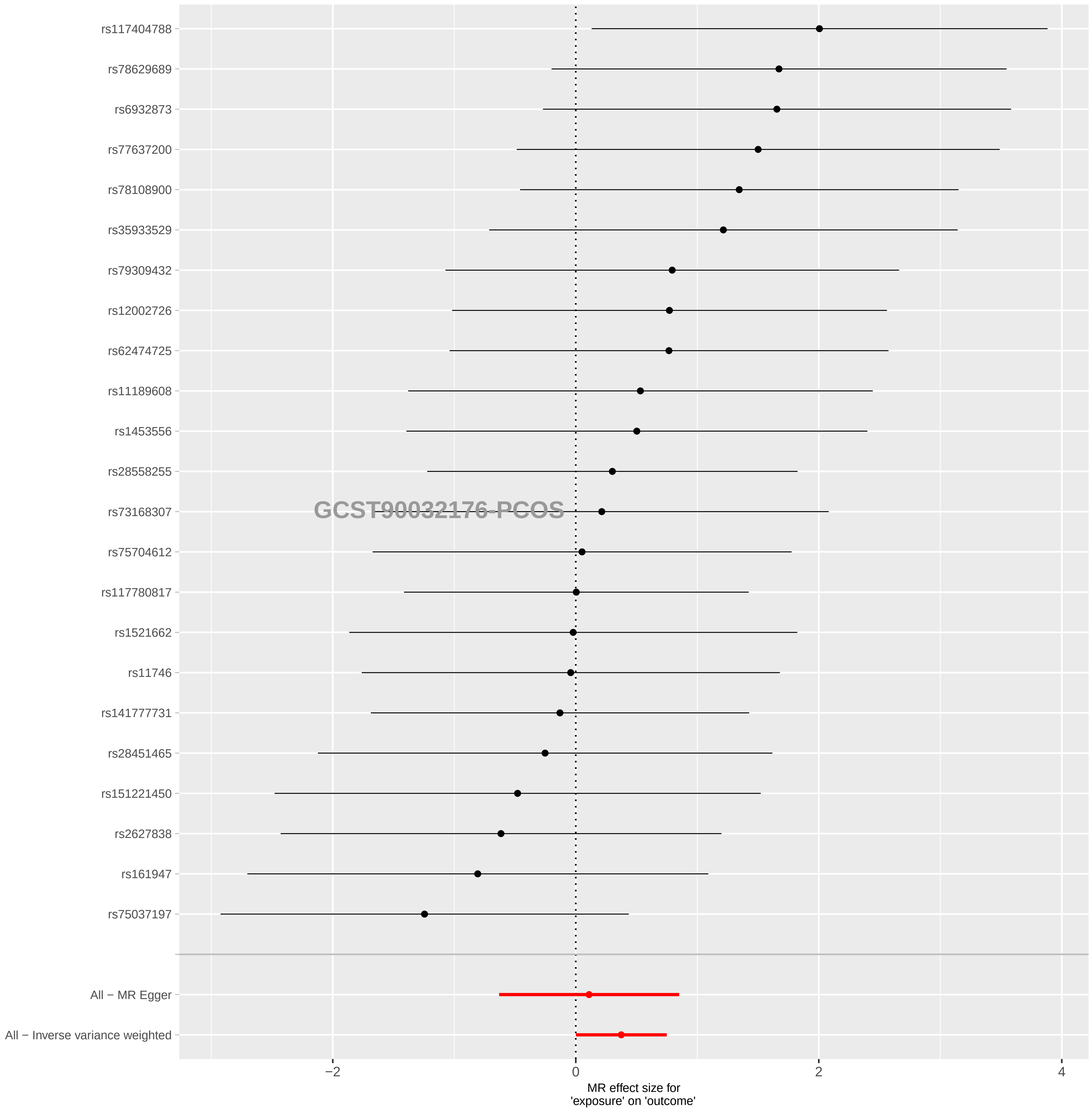
rs117404788
rs78629689
rs6932873
rs77637200
rs78108900
rs35933529
rs79309432
rs12002726
rs62474725
rs11189608
rs1453556
rs28558255
rs73168307
GCST90032176-PCOS
rs75704612
rs117780817
rs1521662
rs11746
rs141777731
rs28451465
rs151221450
rs2627838
rs161947
rs75037197
All − MR Egger
All − Inverse variance weighted
−2
0
2
4
MR effect size for
'exposure' on 'outcome'

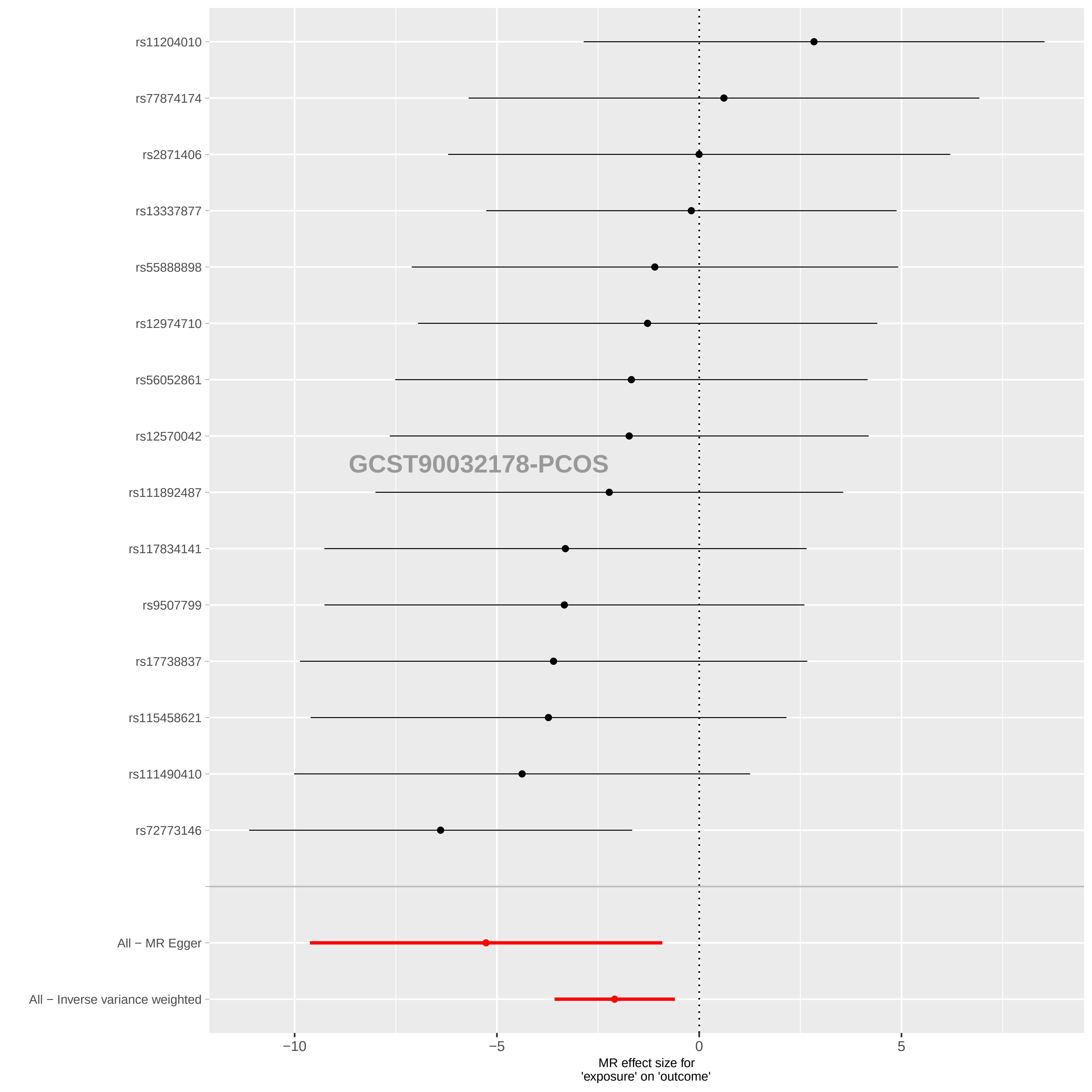

rs11204010
rs77874174
rs2871406
rs13337877
rs55888898
rs12974710
rs56052861
rs12570042
GCST90032178-PCOS
rs111892487
rs117834141
rs9507799
rs17738837
rs115458621
rs111490410
rs72773146
All − MR Egger
All − Inverse variance weighted
−10
−5
0
5
MR effect size for
'exposure' on 'outcome'

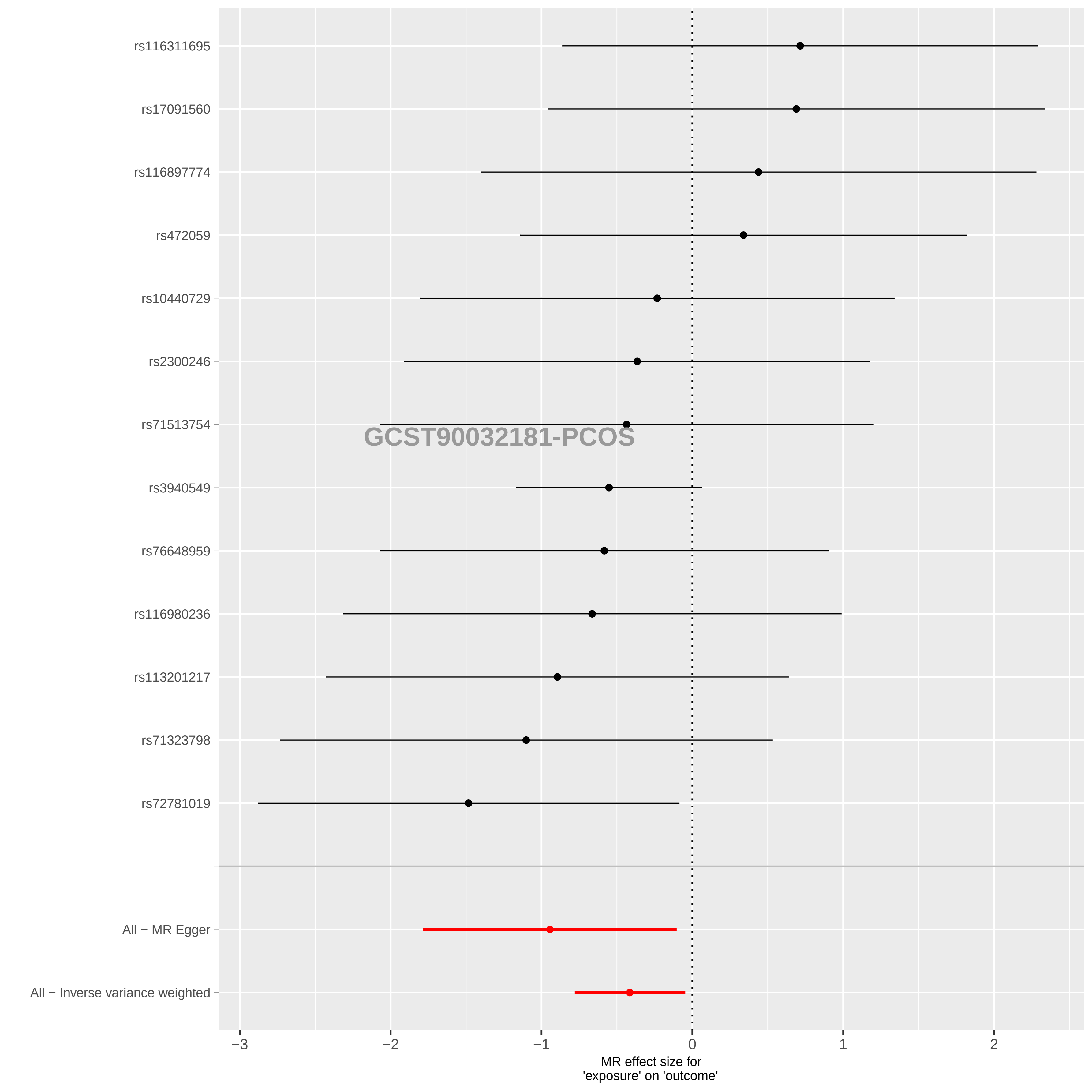
rs116311695
rs17091560
rs116897774
rs472059
rs10440729
rs2300246
rs71513754
GCST90032181-PCOS
rs3940549
rs76648959
rs116980236
rs113201217
rs71323798
rs72781019
All − MR Egger
All − Inverse variance weighted
−3
−2
−1
0
1
2
MR effect size for
'exposure' on 'outcome'

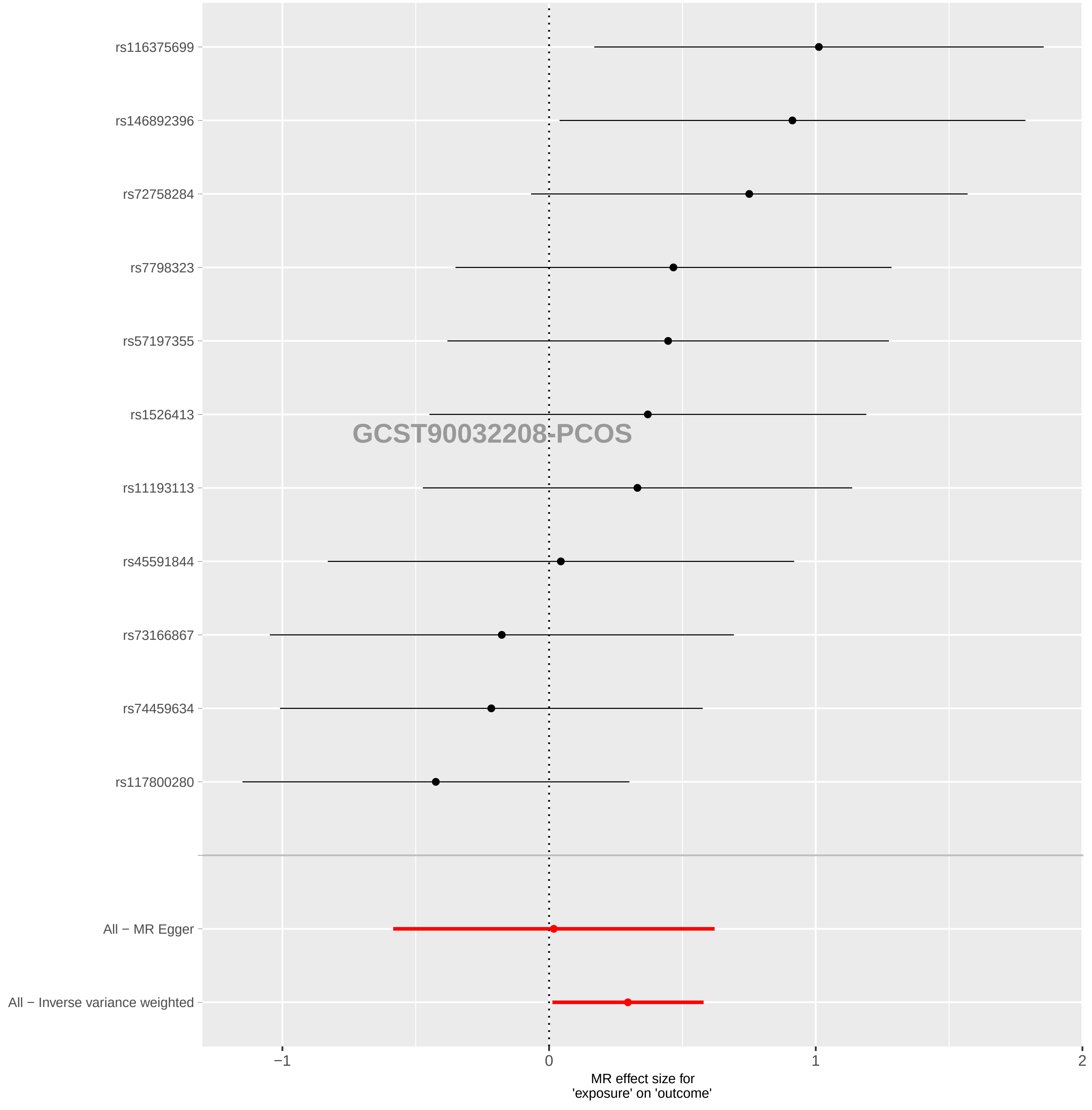
rs116375699
rs146892396
rs72758284
rs7798323
rs57197355
rs1526413
rs11193113
rs45591844
rs73166867
rs74459634
rs117800280
All − MR Egger
All − Inverse variance weighted
GCST90032208-PCOS
−1
0
1
2
MR effect size for
'exposure' on 'outcome'

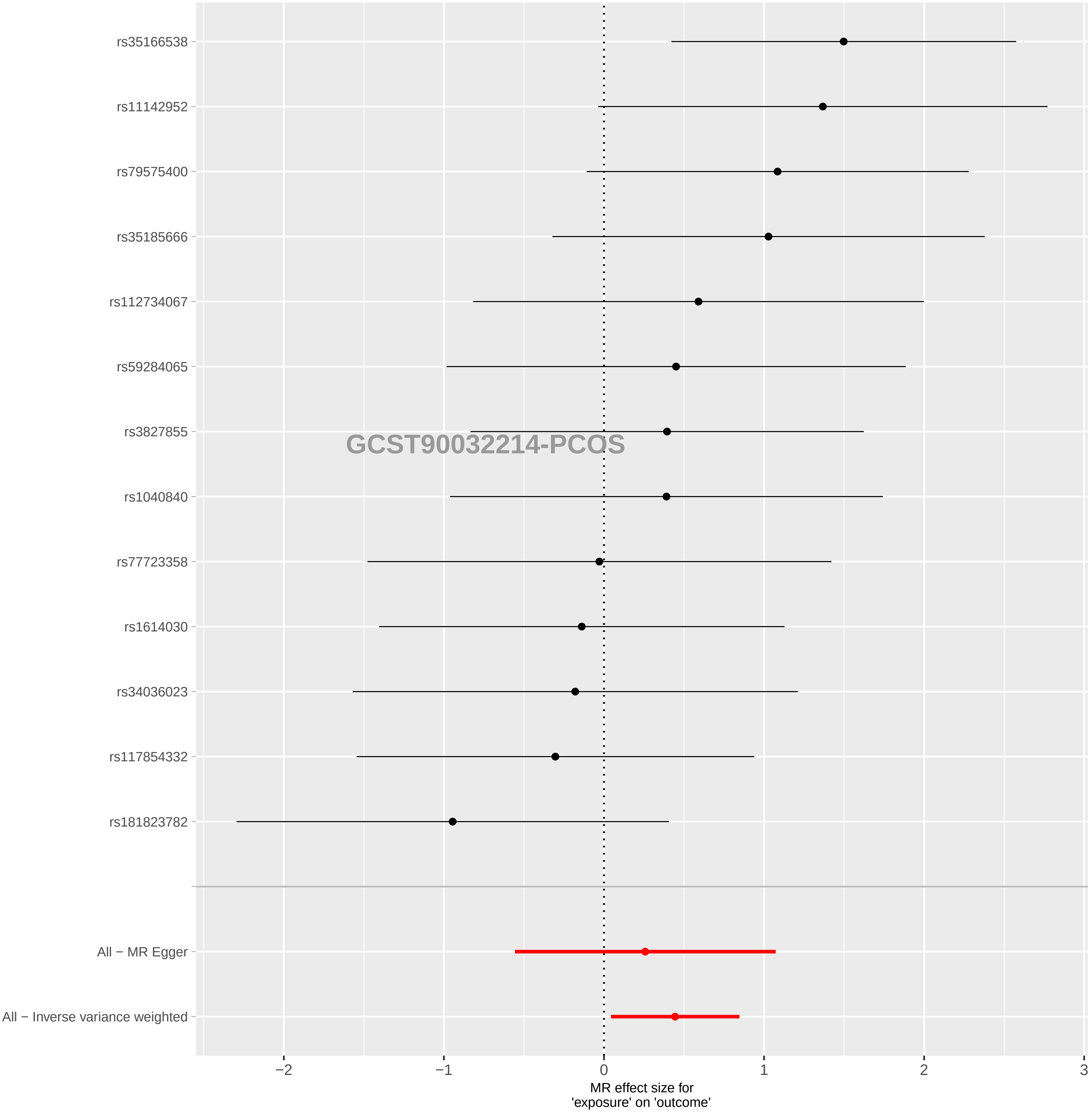

rs35166538
rs11142952
rs79575400
rs35185666
rs112734067
rs59284065
rs3827855
rs1040840
rs77723358
rs1614030
rs34036023
rs117854332
rs181823782
All − MR Egger
All − Inverse variance weighted
GCST90032214-PCOS
−2
−1
0
1
2
3
MR effect size for
'exposure' on 'outcome'

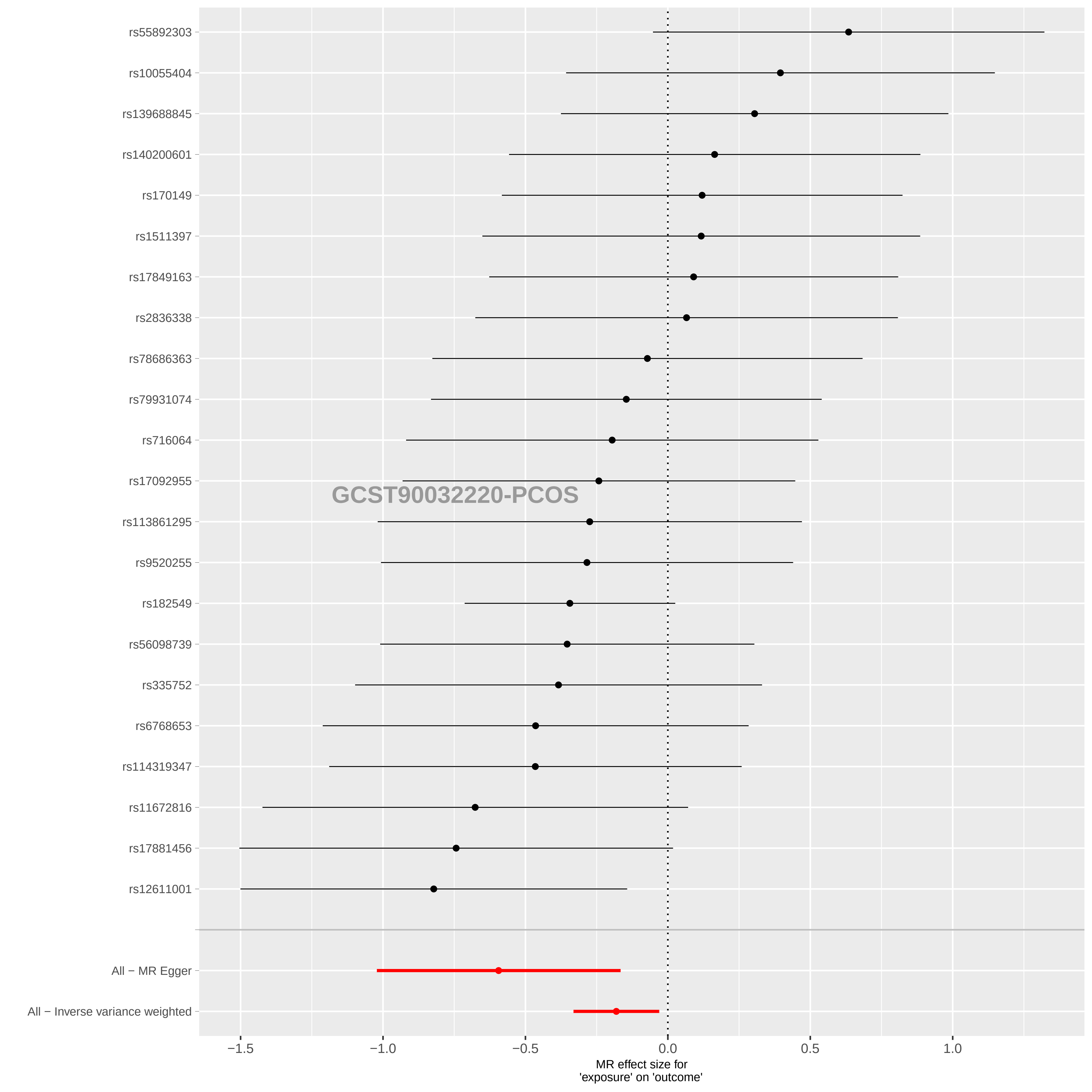

rs55892303
rs10055404
rs139688845
rs140200601
rs170149
rs1511397
rs17849163
rs2836338
rs78686363
rs79931074
rs716064
rs17092955
GCST90032220-PCOS
rs113861295
rs9520255
rs182549
rs56098739
rs335752
rs6768653
rs114319347
rs11672816
rs17881456
rs12611001
All − MR Egger
All − Inverse variance weighted
−1.5
−1.0
−0.5
0.0
0.5
1.0
MR effect size for
'exposure' on 'outcome'

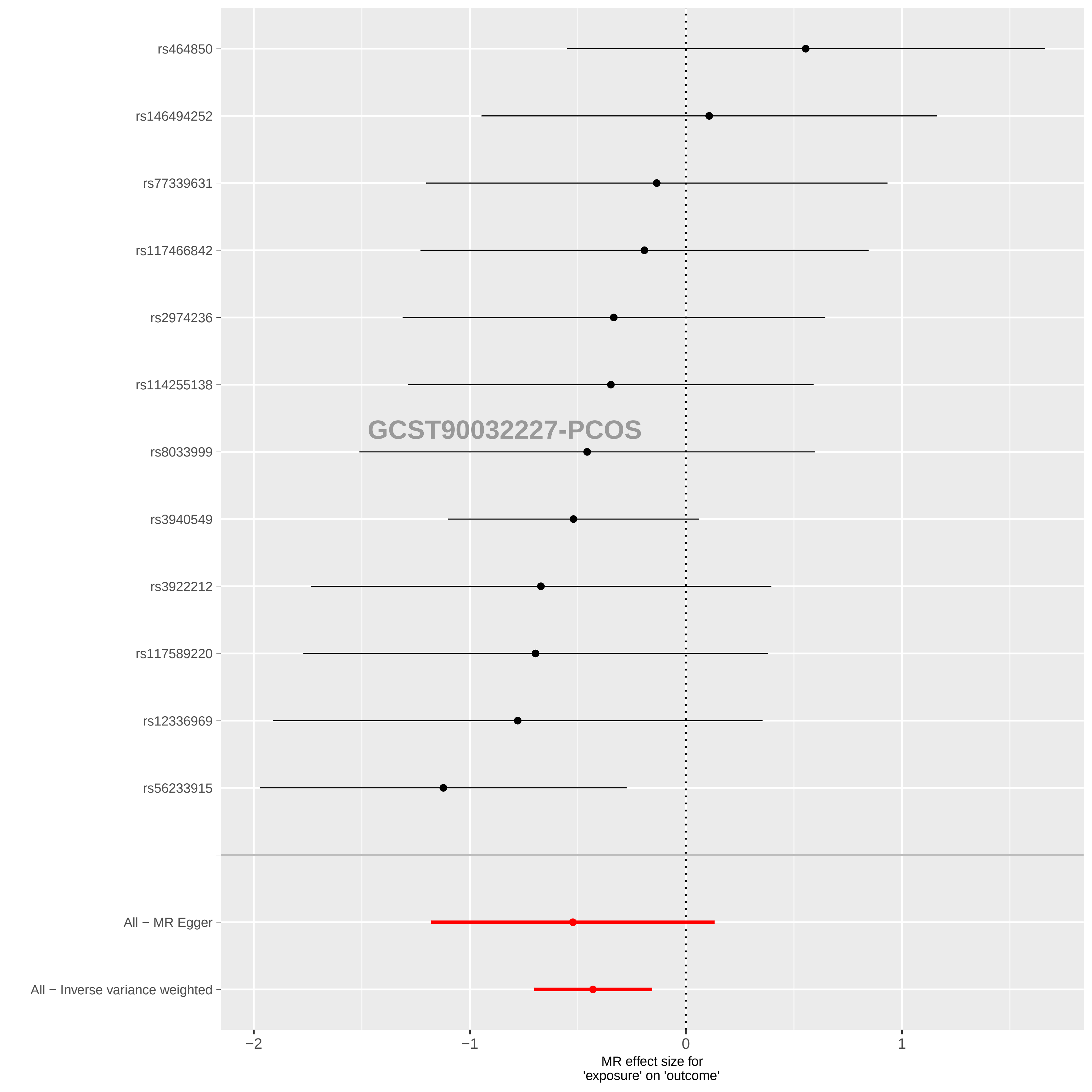

GCST90032227-PCOS
rs464850
rs146494252
rs77339631
rs117466842
rs2974236
rs114255138
rs8033999
rs3940549
rs3922212
rs117589220
rs12336969
rs56233915
All − MR Egger
All − Inverse variance weighted
−2
−1
0
1
MR effect size for
'exposure' on 'outcome'

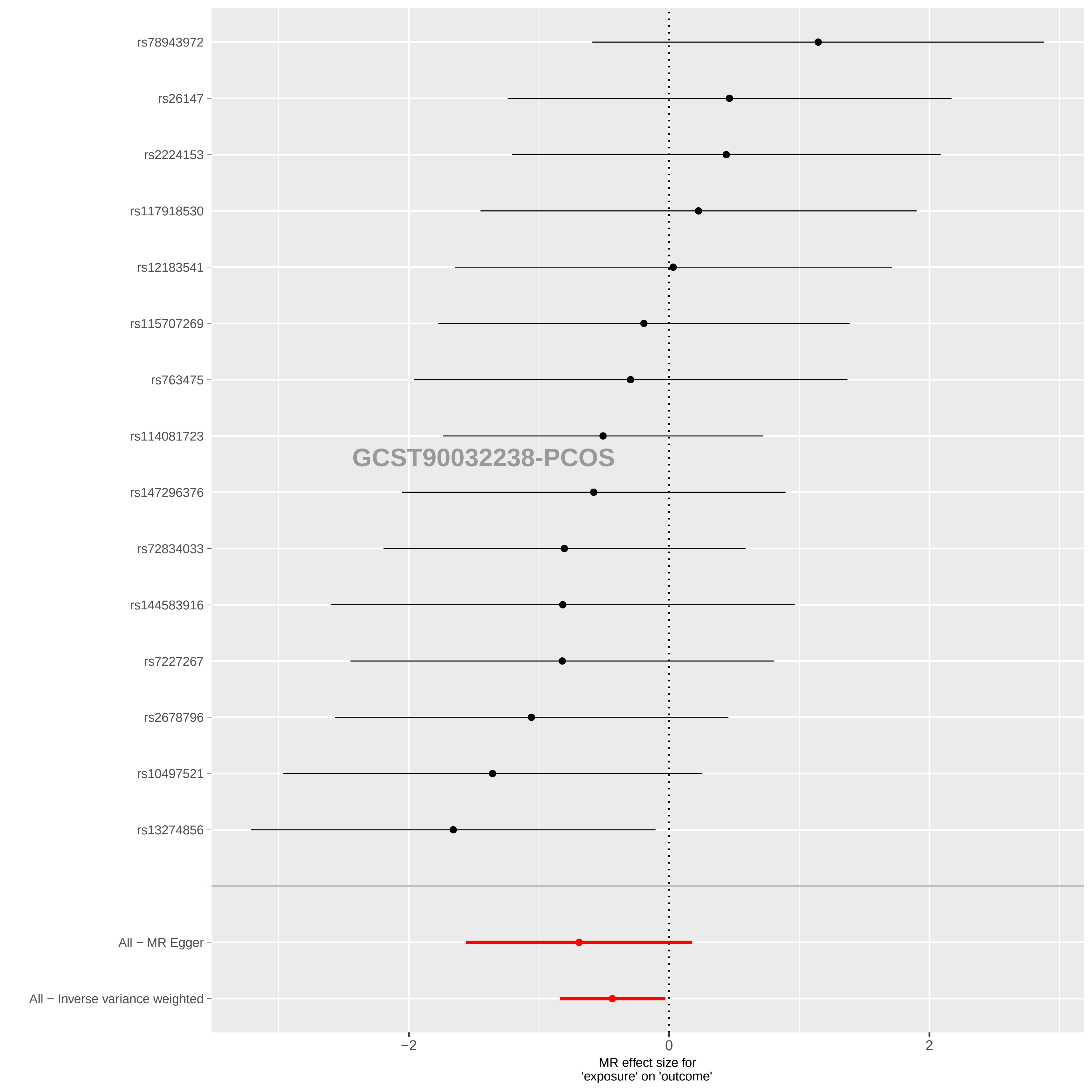

GCST90032238-PCOS
rs78943972
rs26147
rs2224153
rs117918530
rs12183541
rs115707269
rs763475
rs114081723
rs147296376
rs72834033
rs144583916
rs7227267
rs2678796
rs10497521
rs13274856
All − MR Egger
All − Inverse variance weighted
−2
0
2
MR effect size for
'exposure' on 'outcome'

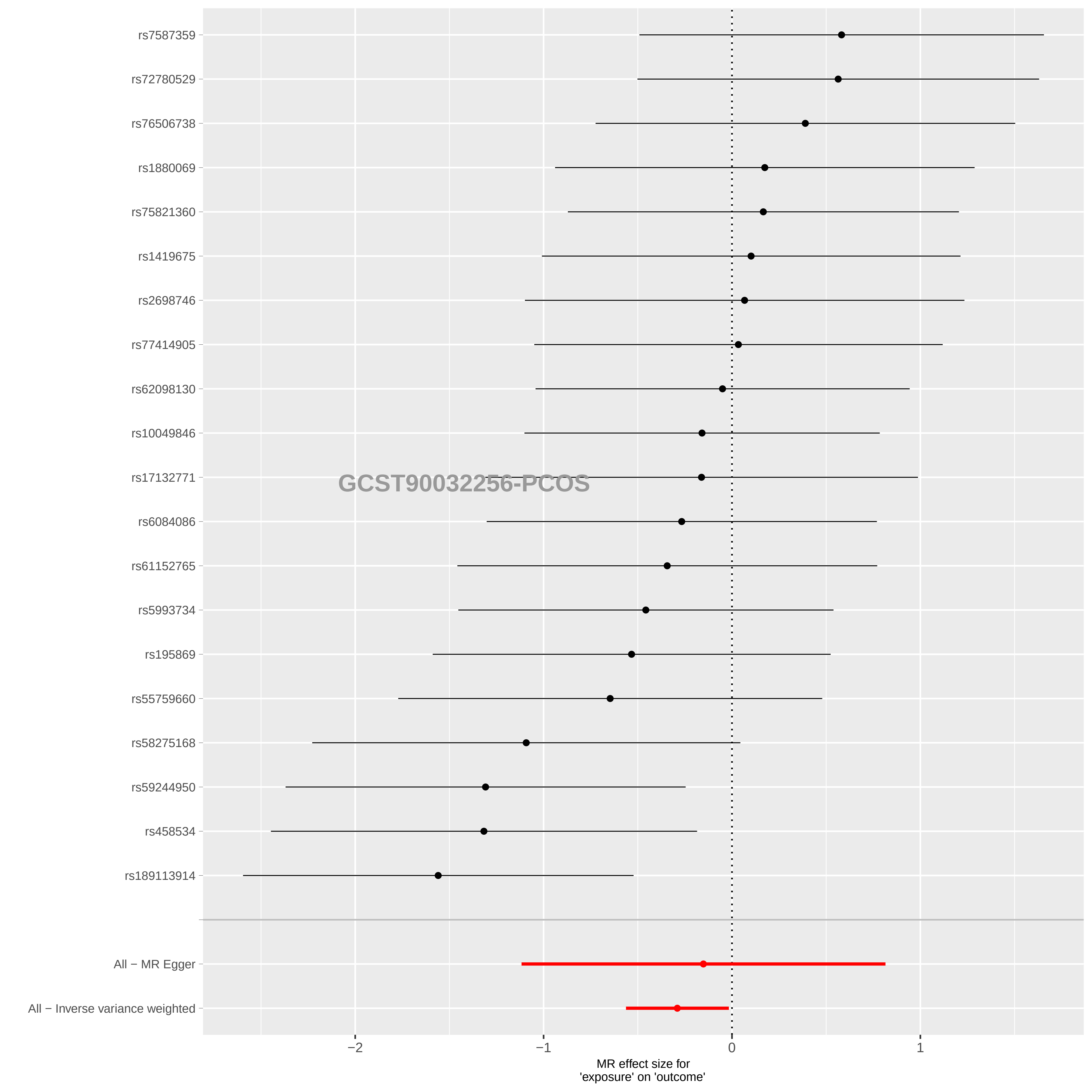

GCST90032256-PCOS
rs7587359
rs72780529
rs76506738
rs1880069
rs75821360
rs1419675
rs2698746
rs77414905
rs62098130
rs10049846
rs17132771
rs6084086
rs61152765
rs5993734
rs195869
rs55759660
rs58275168
rs59244950
rs458534
rs189113914
All − MR Egger
All − Inverse variance weighted
−2
−1
0
1
MR effect size for
'exposure' on 'outcome'

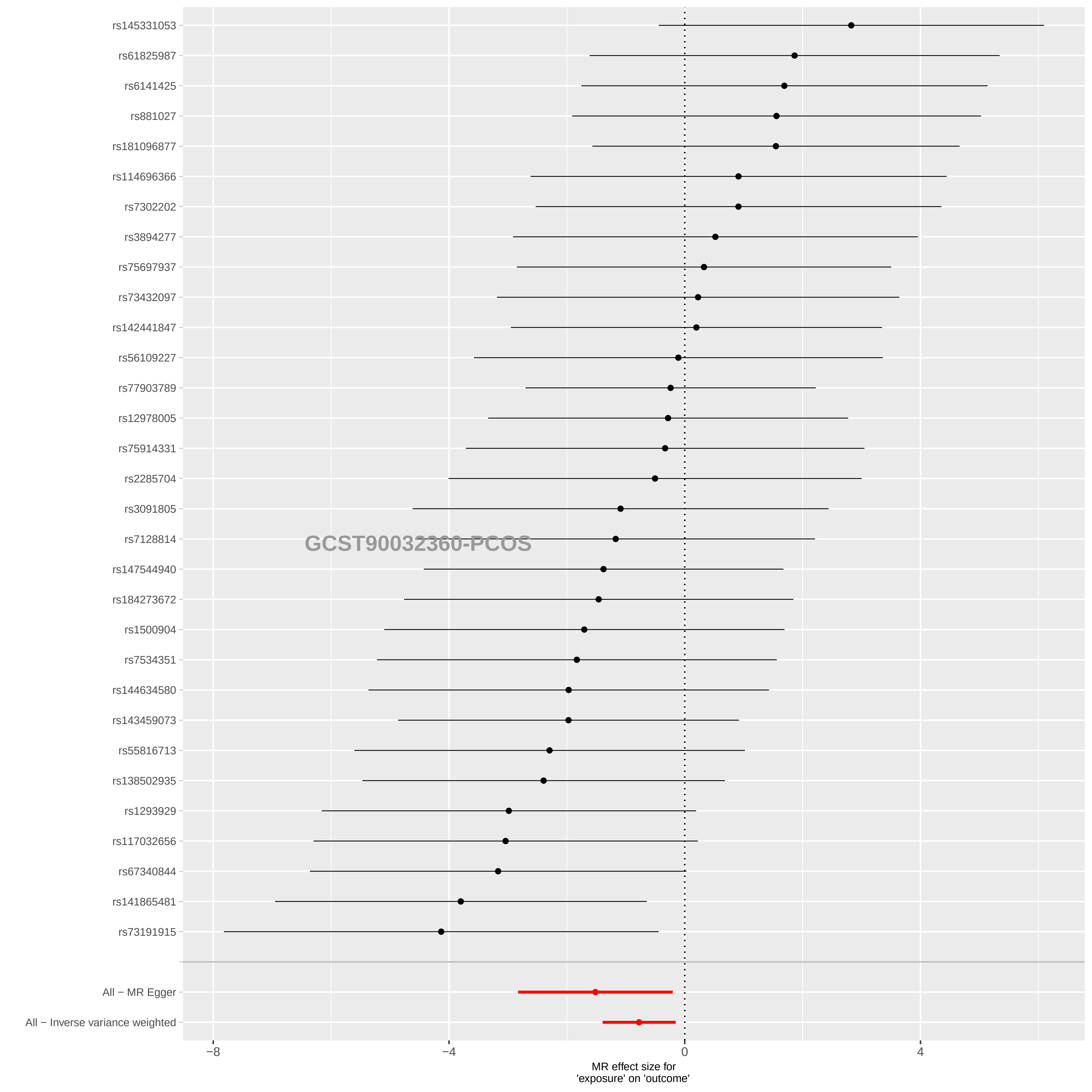
rs145331053
rs61825987
rs6141425
rs881027
rs181096877
rs114696366
rs7302202
rs3894277
rs75697937
rs73432097
rs142441847
rs56109227
rs77903789
rs12978005
rs75914331
rs2285704
rs3091805
rs7128814
GCST90032360-PCOS
rs147544940
rs184273672
rs1500904
rs7534351
rs144634580
rs143459073
rs55816713
rs138502935
rs1293929
rs117032656
rs67340844
rs141865481
rs73191915
All − MR Egger
All − Inverse variance weighted
−8
−4
0
4
MR effect size for
'exposure' on 'outcome'

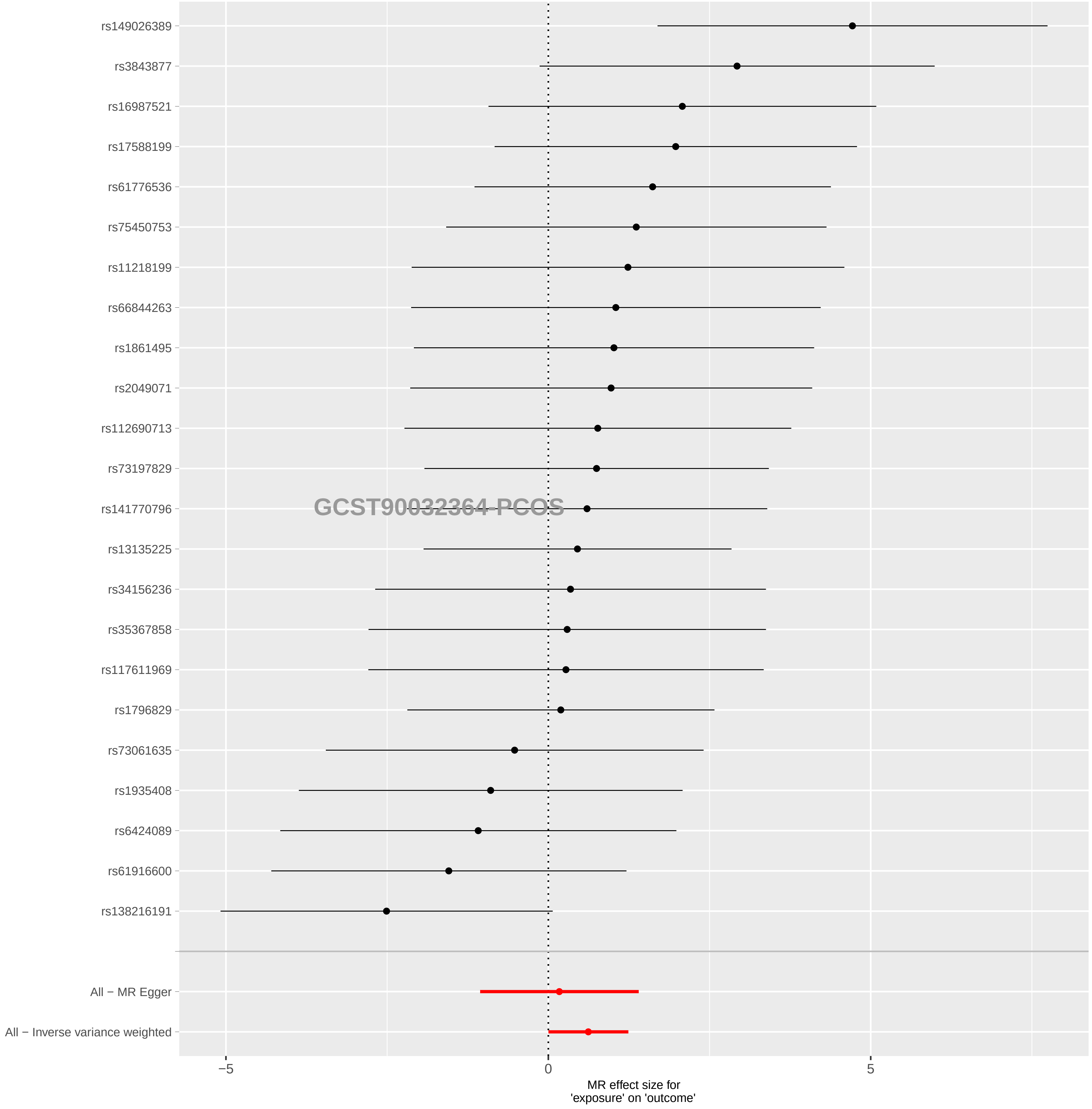
rs149026389
rs3843877
rs16987521
rs17588199
rs61776536
rs75450753
rs11218199
rs66844263
rs1861495
rs2049071
rs112690713
rs73197829
rs141770796
GCST90032364-PCOS
rs13135225
rs34156236
rs35367858
rs117611969
rs1796829
rs73061635
rs1935408
rs6424089
rs61916600
rs138216191
All − MR Egger
All − Inverse variance weighted
−5
0
5
MR effect size for
'exposure' on 'outcome'

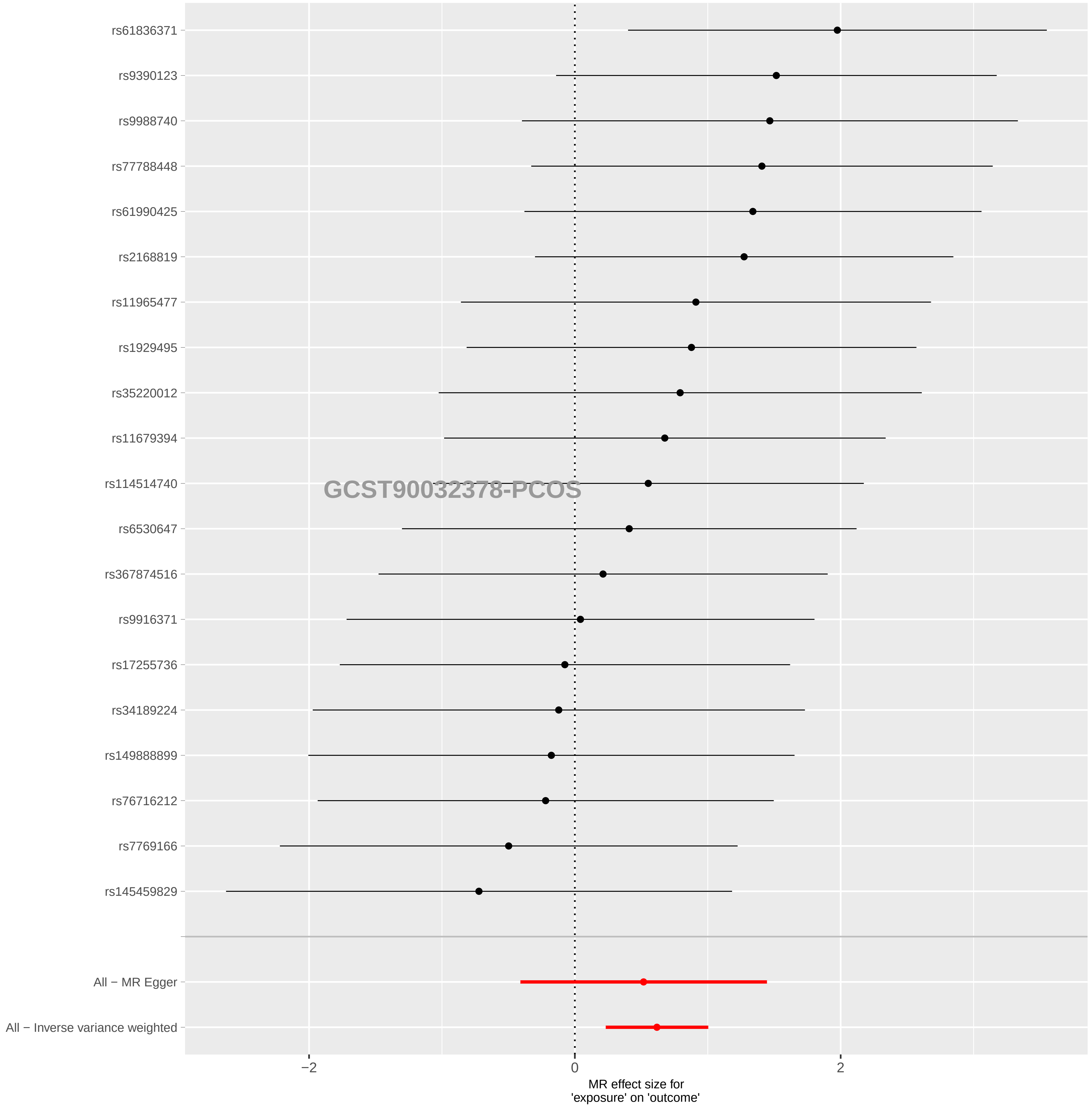

rs61836371
rs9390123
rs9988740
rs77788448
rs61990425
rs2168819
rs11965477
rs1929495
rs35220012
rs11679394
rs114514740
rs6530647
rs367874516
rs9916371
rs17255736
rs34189224
rs149888899
rs76716212
rs7769166
rs145459829
All − MR Egger
All − Inverse variance weighted
GCST90032378-PCOS
−2
0
2
MR effect size for
'exposure' on 'outcome'

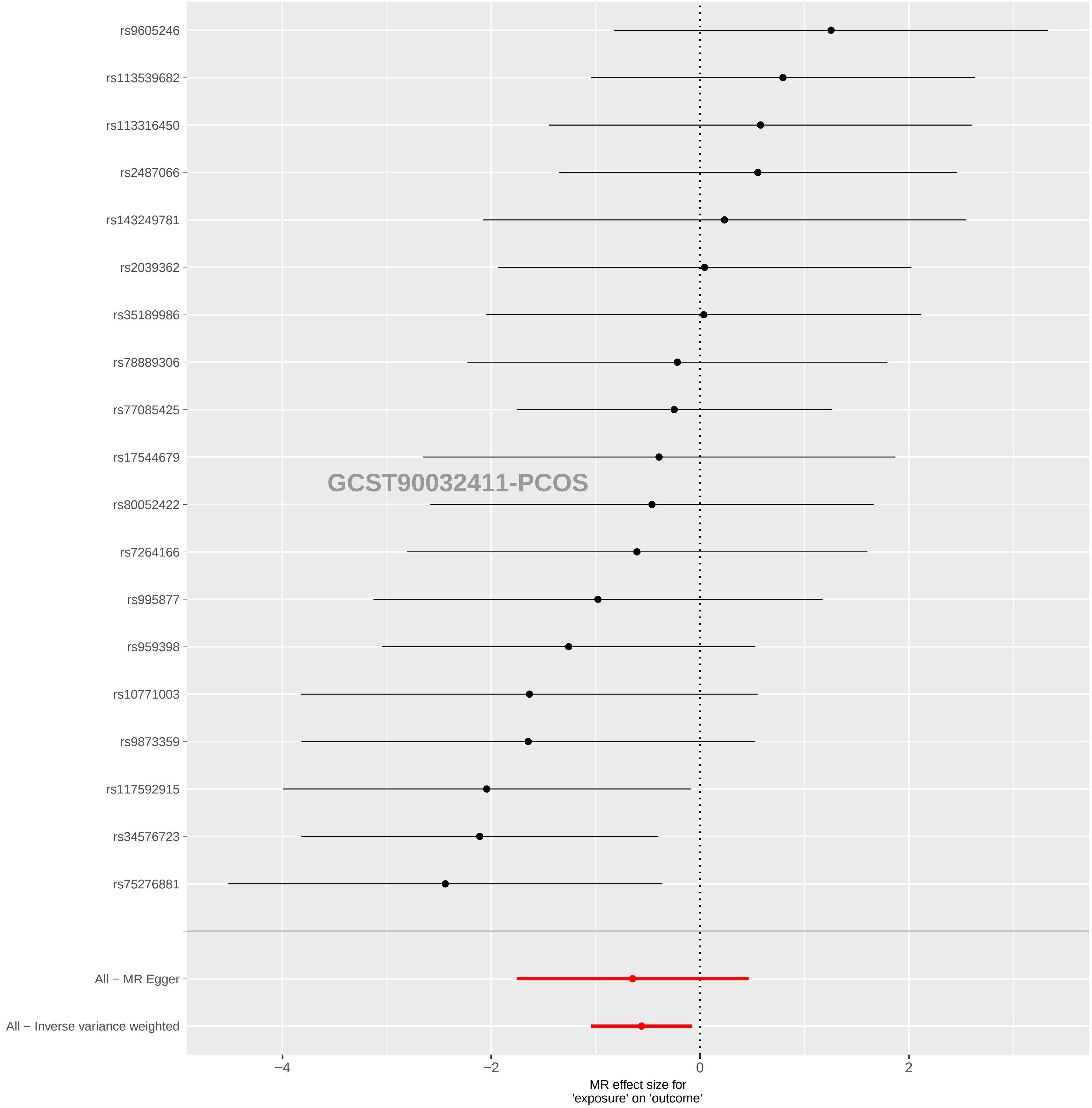

GCST90032411-PCOS
rs9605246
rs113539682
rs113316450
rs2487066
rs143249781
rs2039362
rs35189986
rs78889306
rs77085425
rs17544679
rs80052422
rs7264166
rs995877
rs959398
rs10771003
rs9873359
rs117592915
rs34576723
rs75276881
All − MR Egger
All − Inverse variance weighted
−4
−2
0
2
MR effect size for
'exposure' on 'outcome'

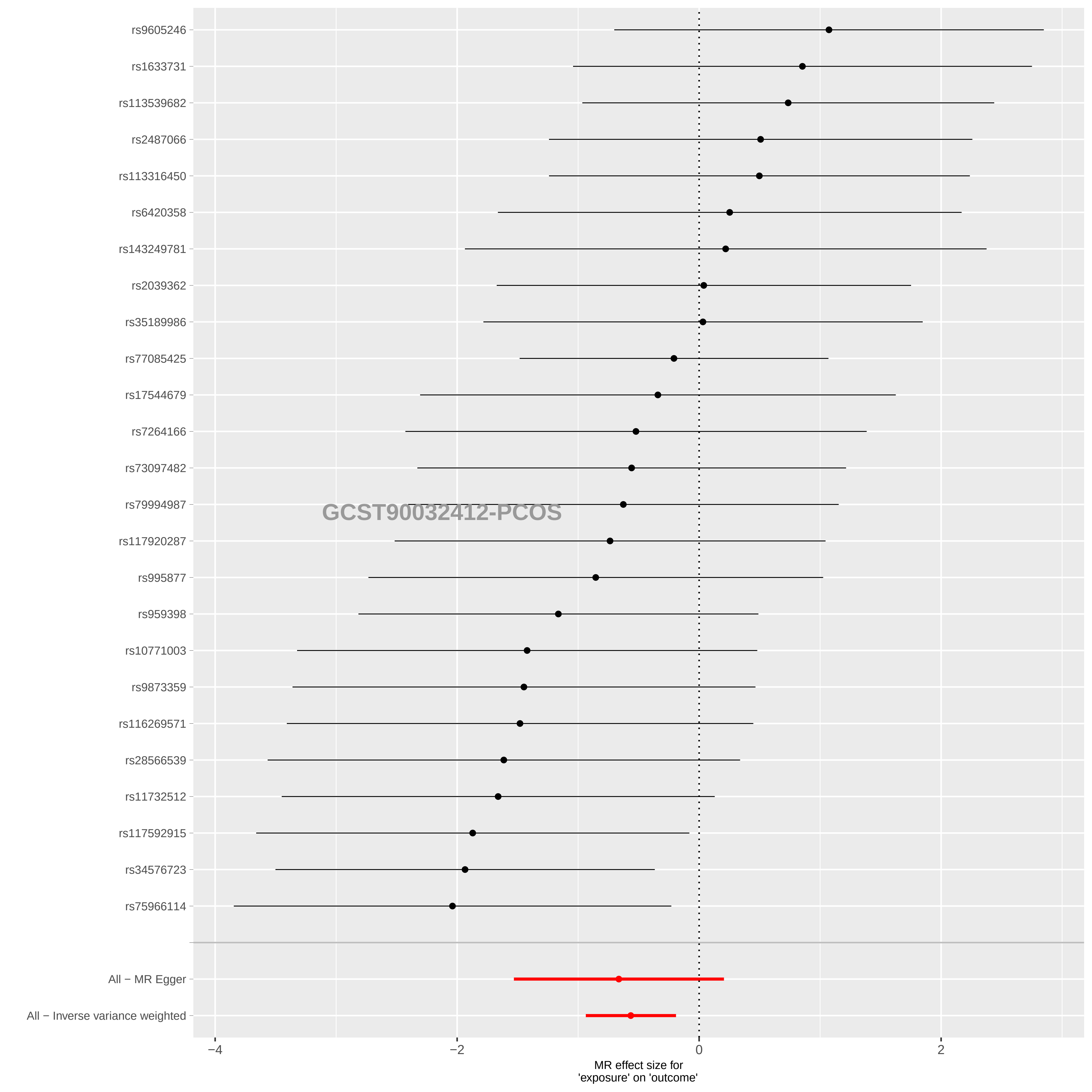

rs9605246
rs1633731
rs113539682
rs2487066
rs113316450
rs6420358
rs143249781
rs2039362
rs35189986
rs77085425
rs17544679
rs7264166
rs73097482
rs79994987
GCST90032412-PCOS
rs117920287
rs995877
rs959398
rs10771003
rs9873359
rs116269571
rs28566539
rs11732512
rs117592915
rs34576723
rs75966114
All − MR Egger
All − Inverse variance weighted
−4
−2
0
2
MR effect size for
'exposure' on 'outcome'

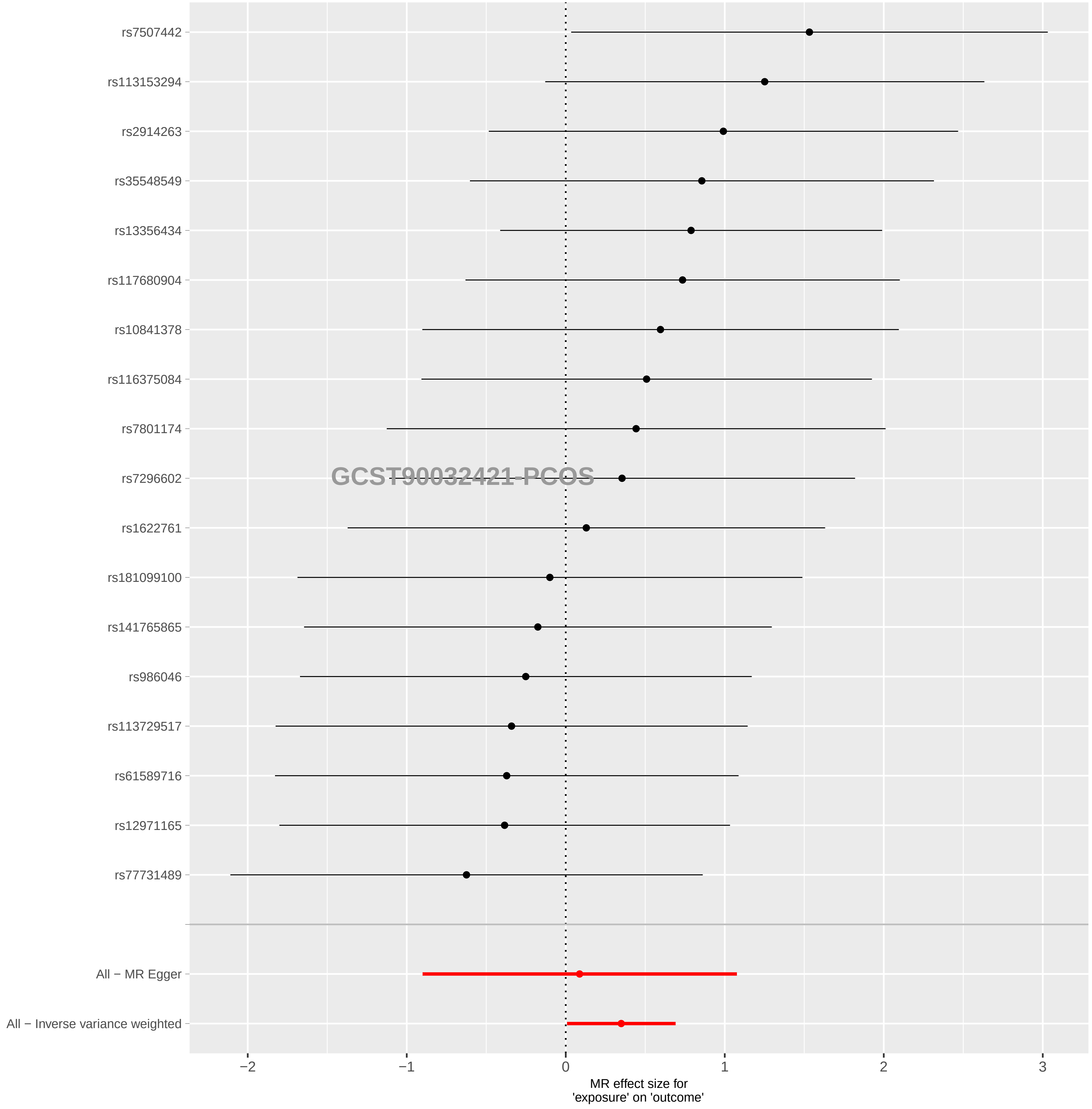

rs7507442
rs113153294
rs2914263
rs35548549
rs13356434
rs117680904
rs10841378
rs116375084
rs7801174
rs7296602
rs1622761
rs181099100
rs141765865
rs986046
rs113729517
rs61589716
rs12971165
rs77731489
All − MR Egger
All − Inverse variance weighted
GCST90032421-PCOS
−2
−1
0
1
2
3
MR effect size for
'exposure' on 'outcome'

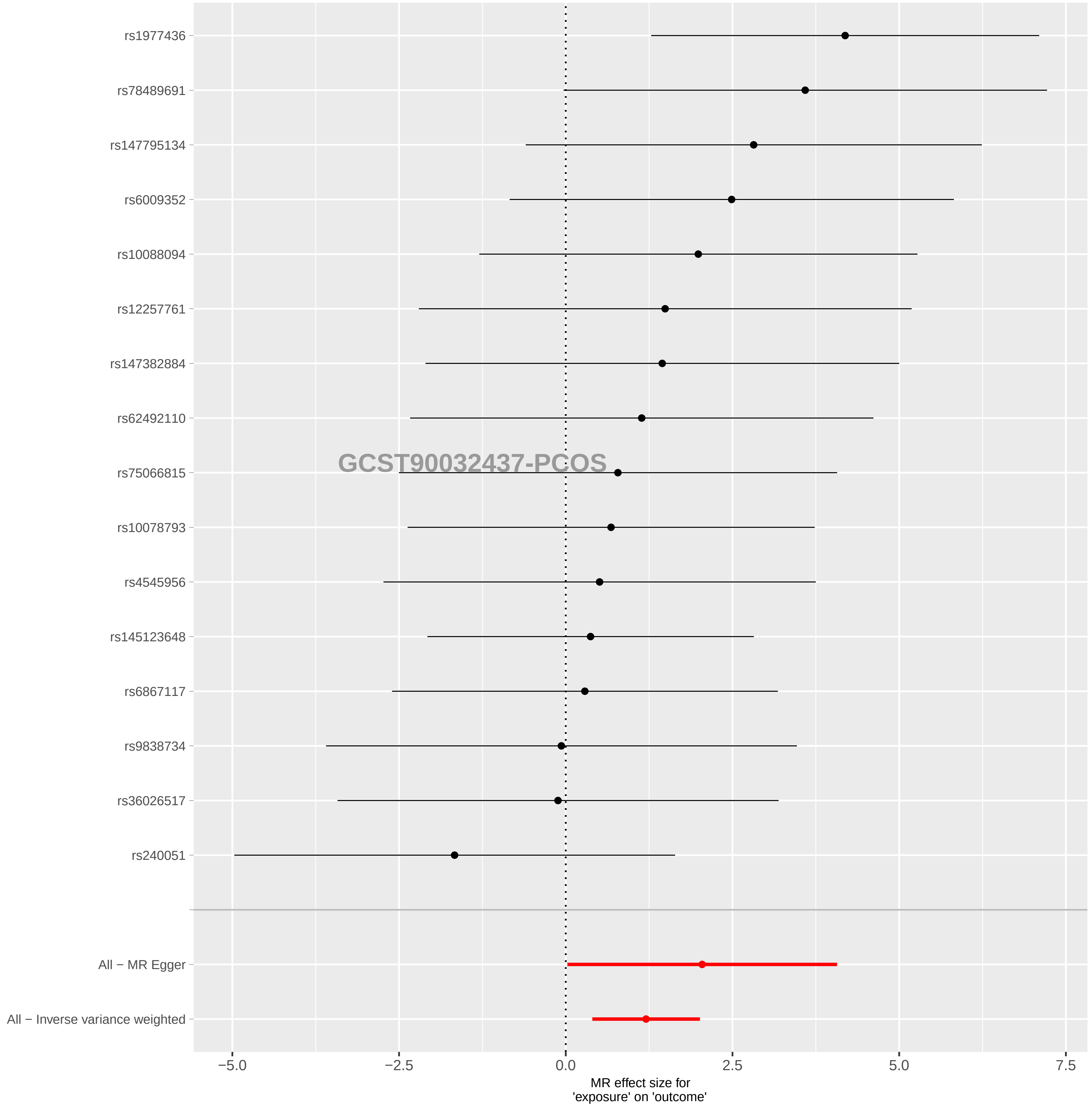

GCST90032437-PCOS
rs1977436
rs78489691
rs147795134
rs6009352
rs10088094
rs12257761
rs147382884
rs62492110
rs75066815
rs10078793
rs4545956
rs145123648
rs6867117
rs9838734
rs36026517
rs240051
All − MR Egger
All − Inverse variance weighted
−5.0
−2.5
0.0
2.5
5.0
7.5
MR effect size for
'exposure' on 'outcome'

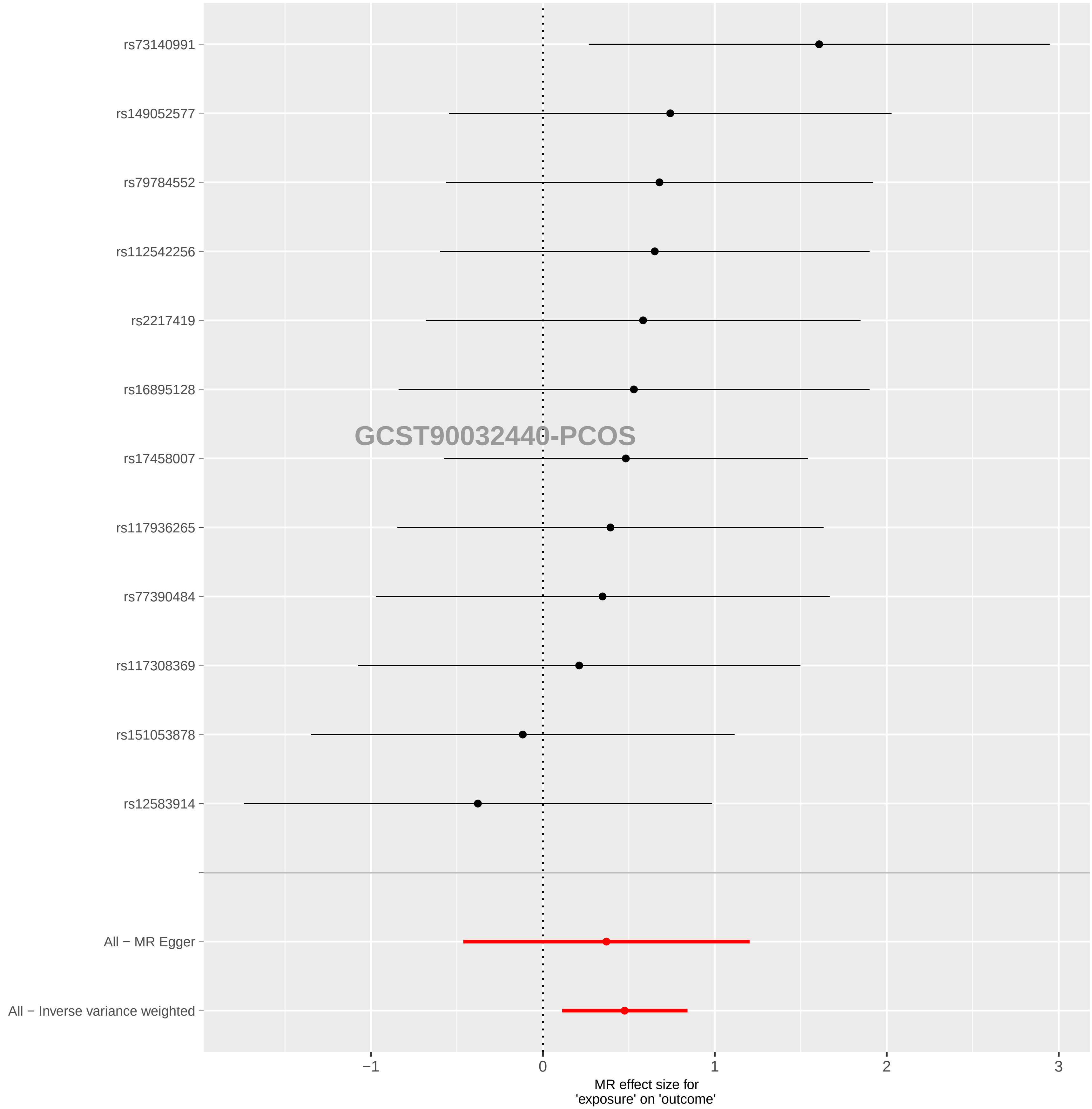

rs73140991
rs149052577
rs79784552
rs112542256
rs2217419
rs16895128
GCST90032440-PCOS
rs17458007
rs117936265
rs77390484
rs117308369
rs151053878
rs12583914
All − MR Egger
All − Inverse variance weighted
−1
0
1
2
3
MR effect size for
'exposure' on 'outcome'

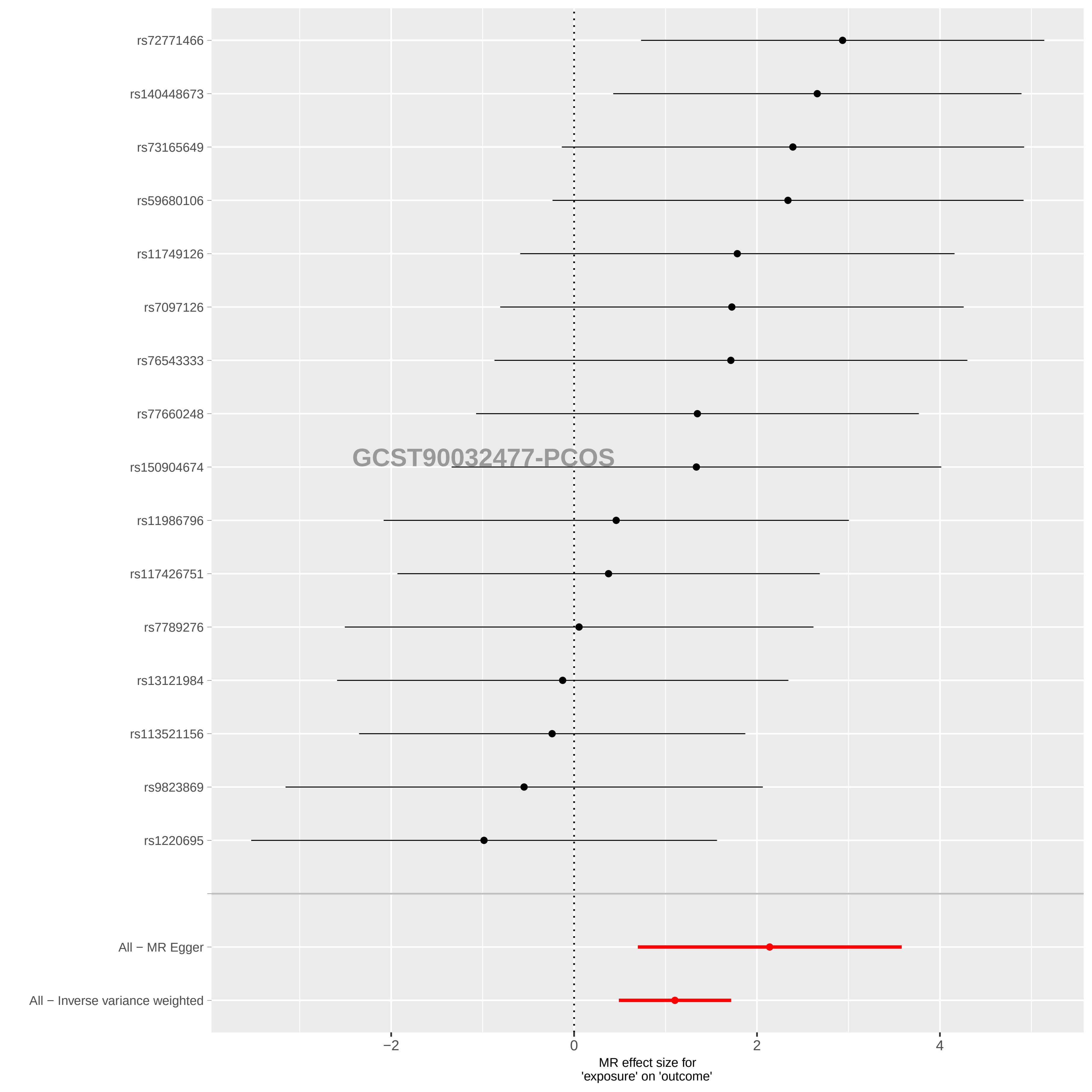

rs72771466
rs140448673
rs73165649
rs59680106
rs11749126
rs7097126
rs76543333
rs77660248
GCST90032477-PCOS
rs150904674
rs11986796
rs117426751
rs7789276
rs13121984
rs113521156
rs9823869
rs1220695
All − MR Egger
All − Inverse variance weighted
−2
0
2
4
MR effect size for
'exposure' on 'outcome'

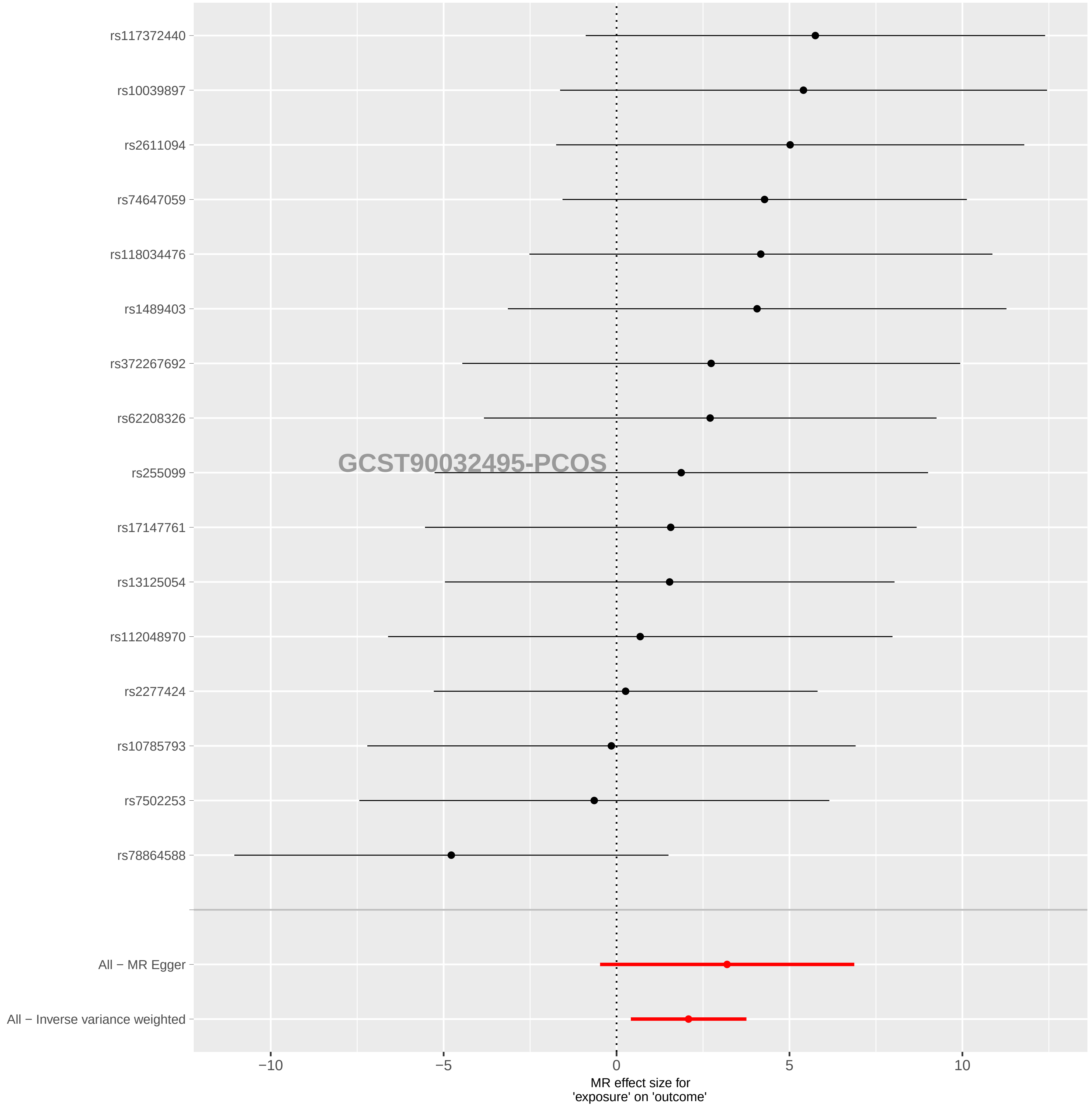

GCST90032495-PCOS
rs117372440
rs10039897
rs2611094
rs74647059
rs118034476
rs1489403
rs372267692
rs62208326
rs255099
rs17147761
rs13125054
rs112048970
rs2277424
rs10785793
rs7502253
rs78864588
All − MR Egger
All − Inverse variance weighted
−10
−5
0
5
10
MR effect size for
'exposure' on 'outcome'

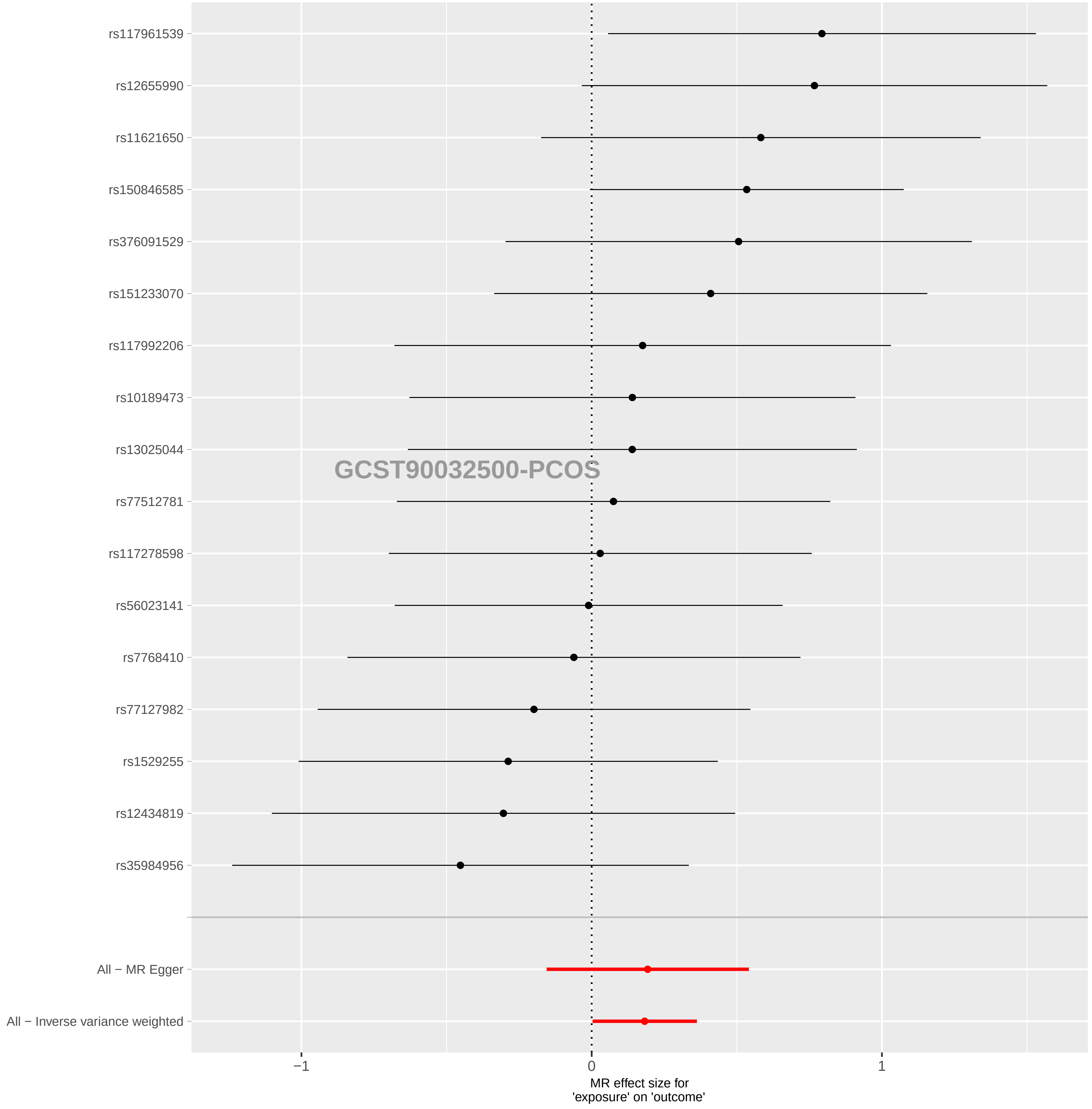

rs117961539
rs12655990
rs11621650
rs150846585
rs376091529
rs151233070
rs117992206
rs10189473
rs13025044
GCST90032500-PCOS
rs77512781
rs117278598
rs56023141
rs7768410
rs77127982
rs1529255
rs12434819
rs35984956
All − MR Egger
All − Inverse variance weighted
−1
0
1
MR effect size for
'exposure' on 'outcome'

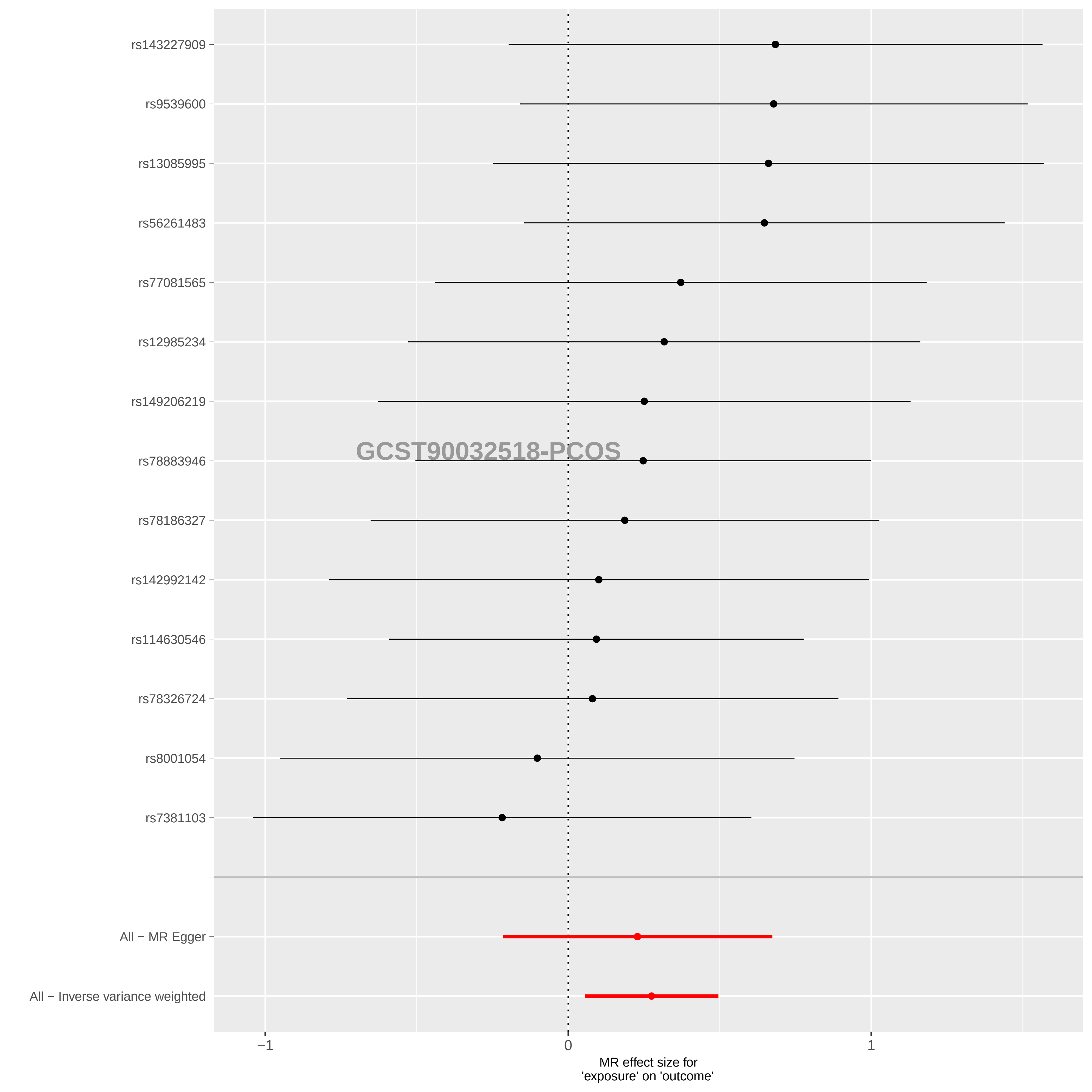
rs143227909
rs9539600
rs13085995
rs56261483
rs77081565
rs12985234
rs149206219
GCST90032518-PCOS
rs78883946
rs78186327
rs142992142
rs114630546
rs78326724
rs8001054
rs7381103
All − MR Egger
All − Inverse variance weighted
−1
0
1
MR effect size for
'exposure' on 'outcome'

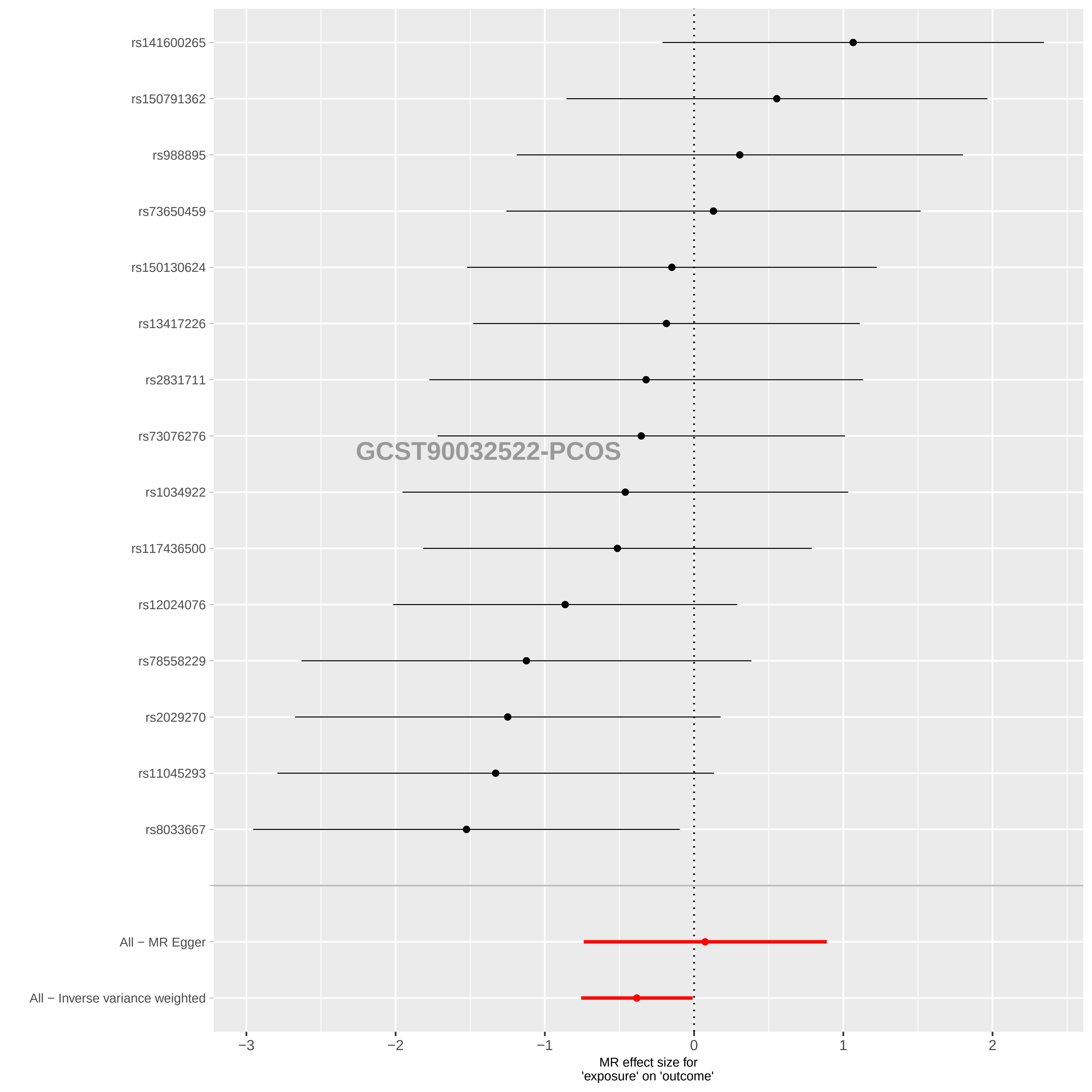

rs141600265
rs150791362
rs988895
rs73650459
rs150130624
rs13417226
rs2831711
rs73076276
GCST90032522-PCOS
rs1034922
rs117436500
rs12024076
rs78558229
rs2029270
rs11045293
rs8033667
All − MR Egger
All − Inverse variance weighted
−3
−2
−1
0
1
2
MR effect size for
'exposure' on 'outcome'

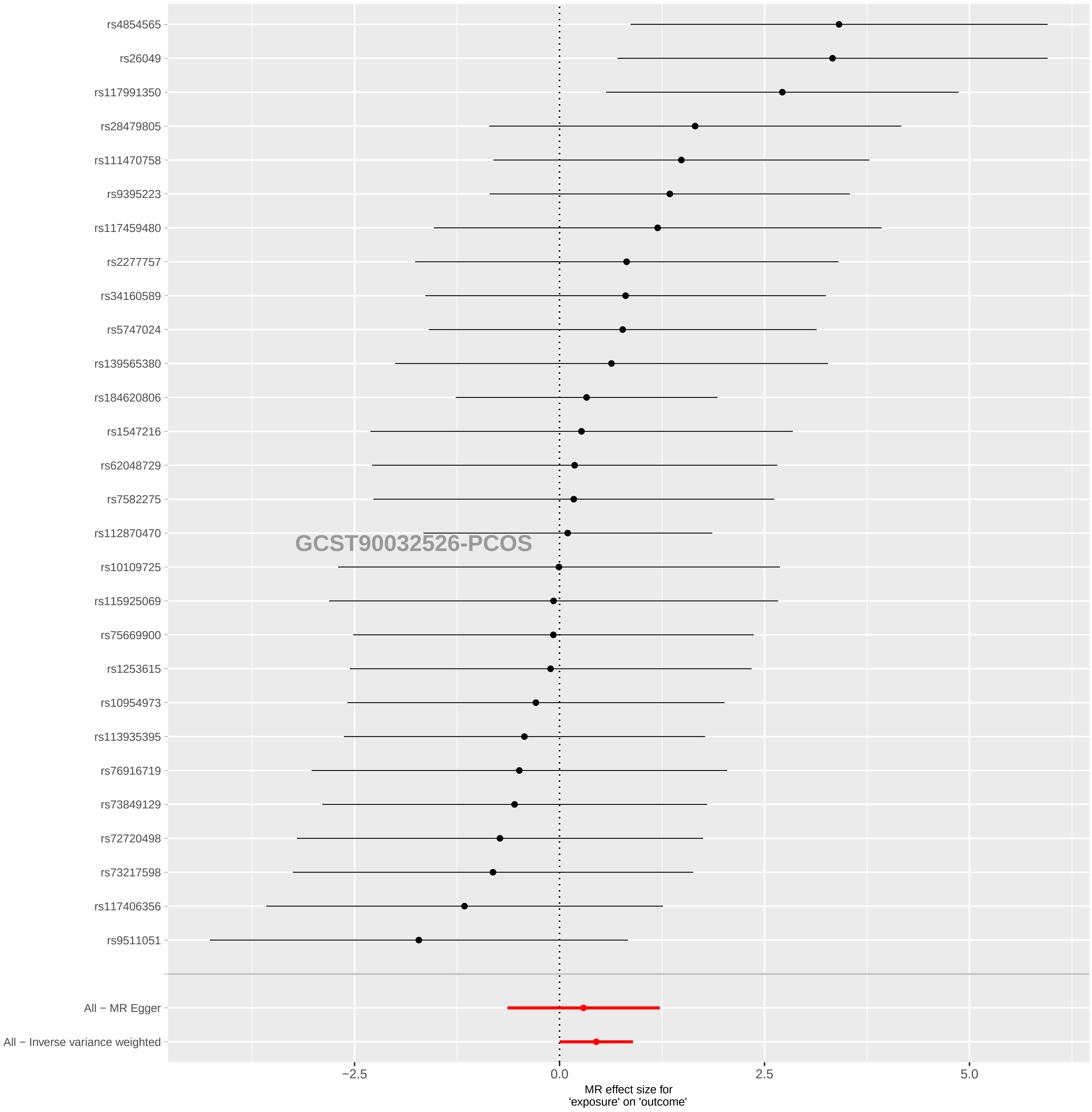
GCST90032526-PCOS
rs4854565
rs26049
rs117991350
rs28479805
rs111470758
rs9395223
rs117459480
rs2277757
rs34160589
rs5747024
rs139565380
rs184620806
rs1547216
rs62048729
rs7582275
rs112870470
rs10109725
rs115925069
rs75669900
rs1253615
rs10954973
rs113935395
rs76916719
rs73849129
rs72720498
rs73217598
rs117406356
rs9511051
All − MR Egger
All − Inverse variance weighted
−2.5
0.0
2.5
5.0
MR effect size for
'exposure' on 'outcome'

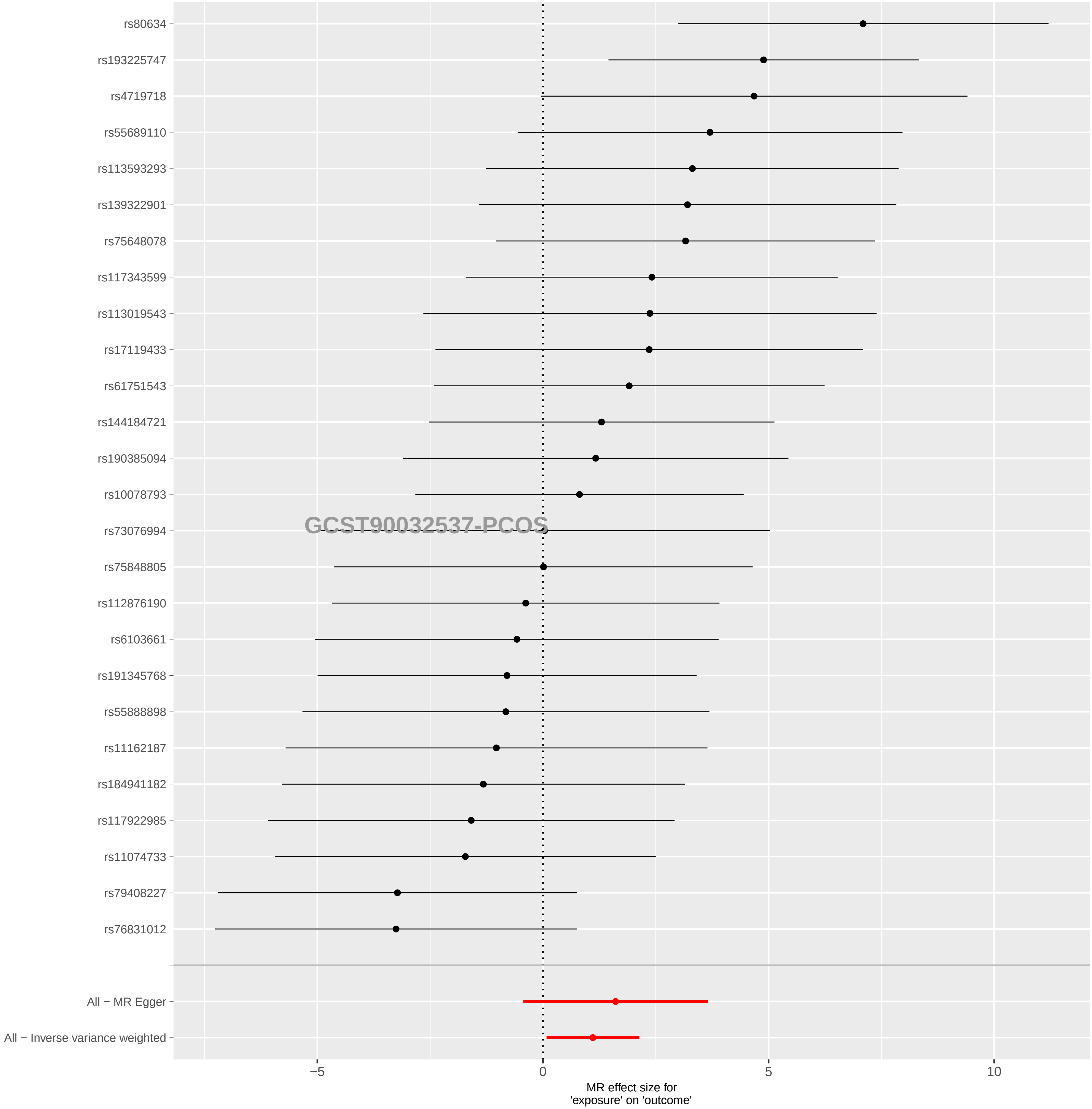

rs80634
rs193225747
rs4719718
rs55689110
rs113593293
rs139322901
rs75648078
rs117343599
rs113019543
rs17119433
rs61751543
rs144184721
rs190385094
rs10078793
rs73076994
rs75848805
rs112876190
rs6103661
rs191345768
rs55888898
rs11162187
rs184941182
rs117922985
rs11074733
rs79408227
rs76831012
All − MR Egger
All − Inverse variance weighted
GCST90032537-PCOS
−5
0
5
10
MR effect size for
'exposure' on 'outcome'

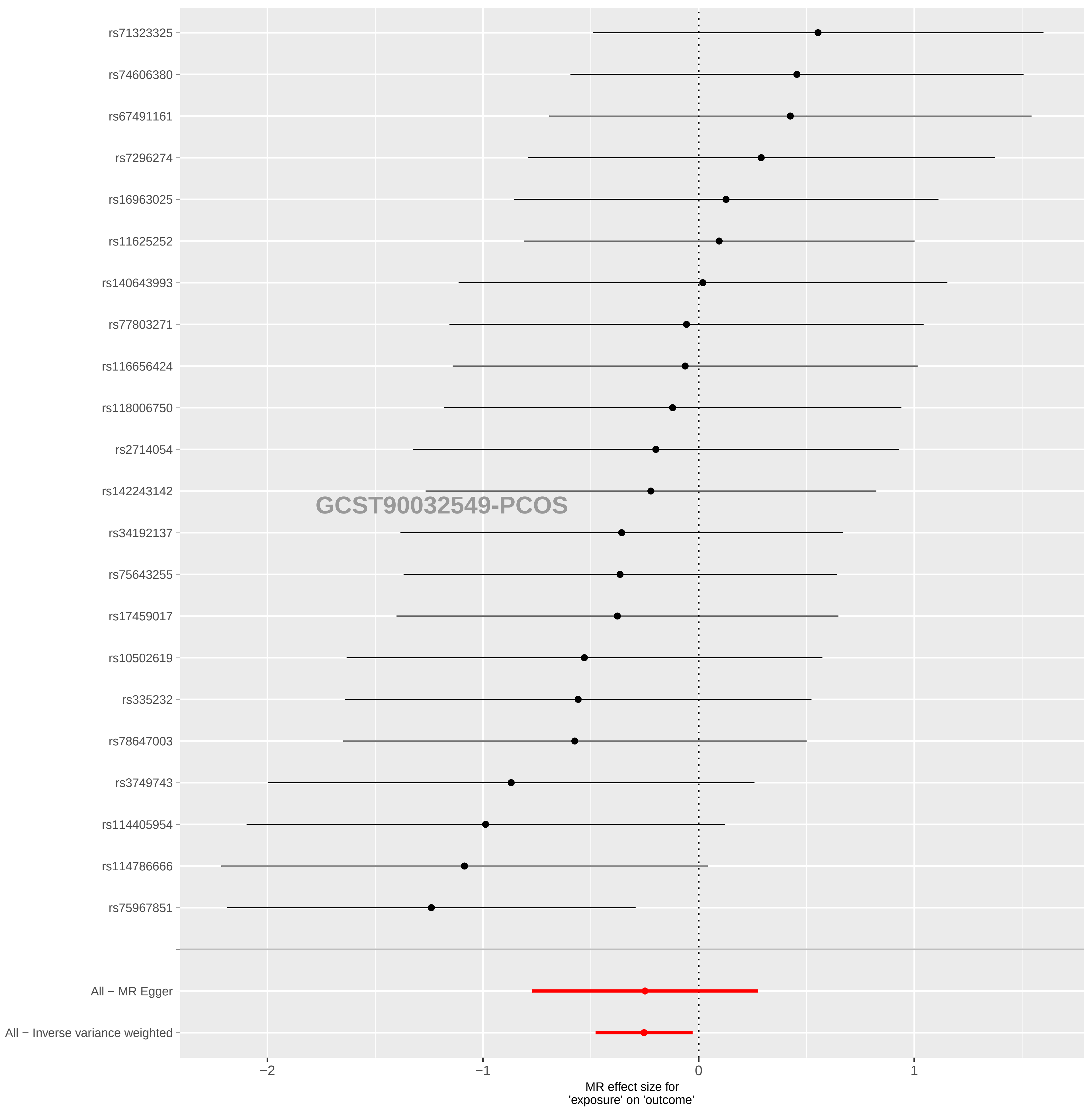

rs71323325
rs74606380
rs67491161
rs7296274
rs16963025
rs11625252
rs140643993
rs77803271
rs116656424
rs118006750
rs2714054
rs142243142
GCST90032549-PCOS
rs34192137
rs75643255
rs17459017
rs10502619
rs335232
rs78647003
rs3749743
rs114405954
rs114786666
rs75967851
All − MR Egger
All − Inverse variance weighted
−2
−1
0
1
MR effect size for
'exposure' on 'outcome'

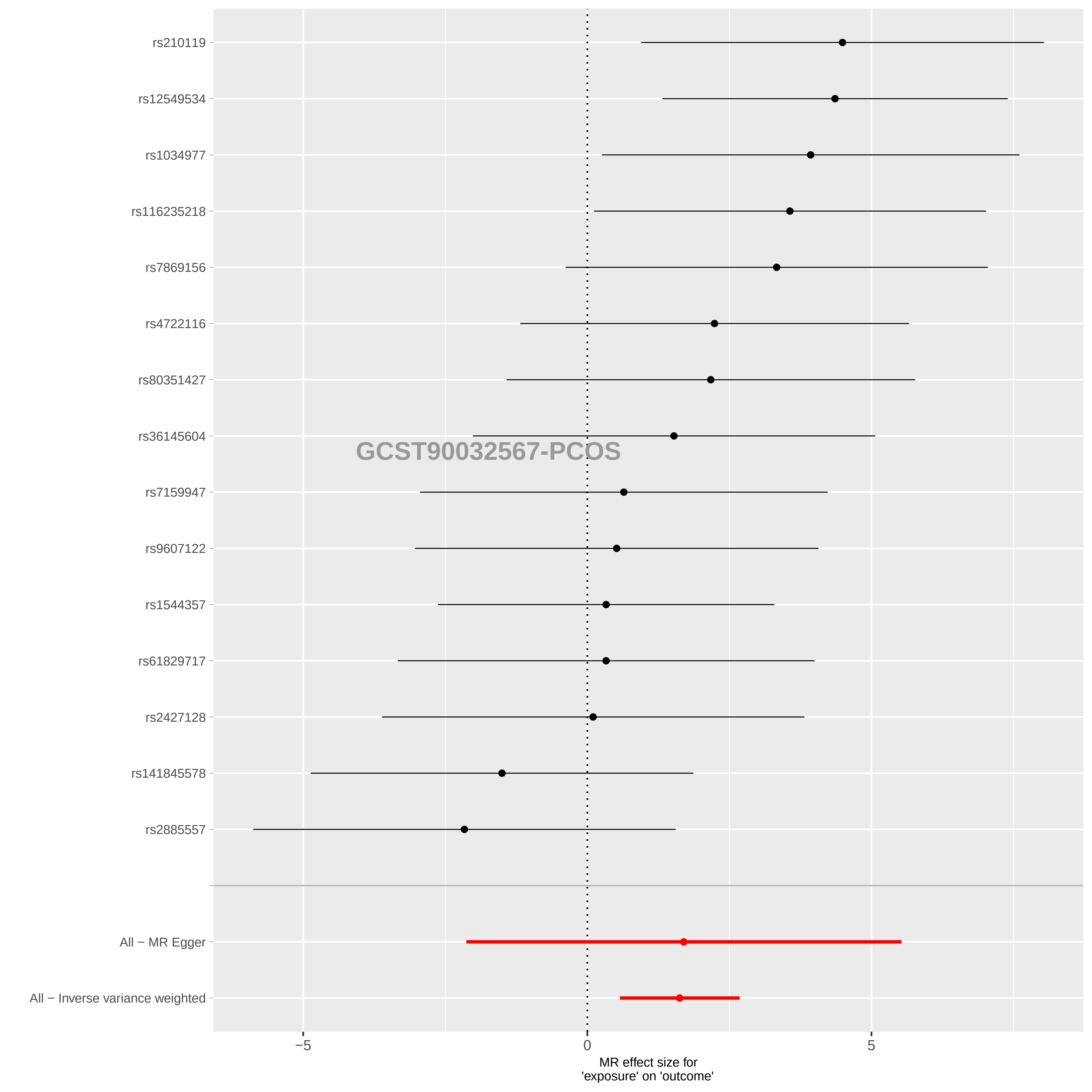

rs210119
rs12549534
rs1034977
rs116235218
rs7869156
rs4722116
rs80351427
rs36145604
GCST90032567-PCOS
rs7159947
rs9607122
rs1544357
rs61829717
rs2427128
rs141845578
rs2885557
All − MR Egger
All − Inverse variance weighted
−5
0
5
MR effect size for
'exposure' on 'outcome'

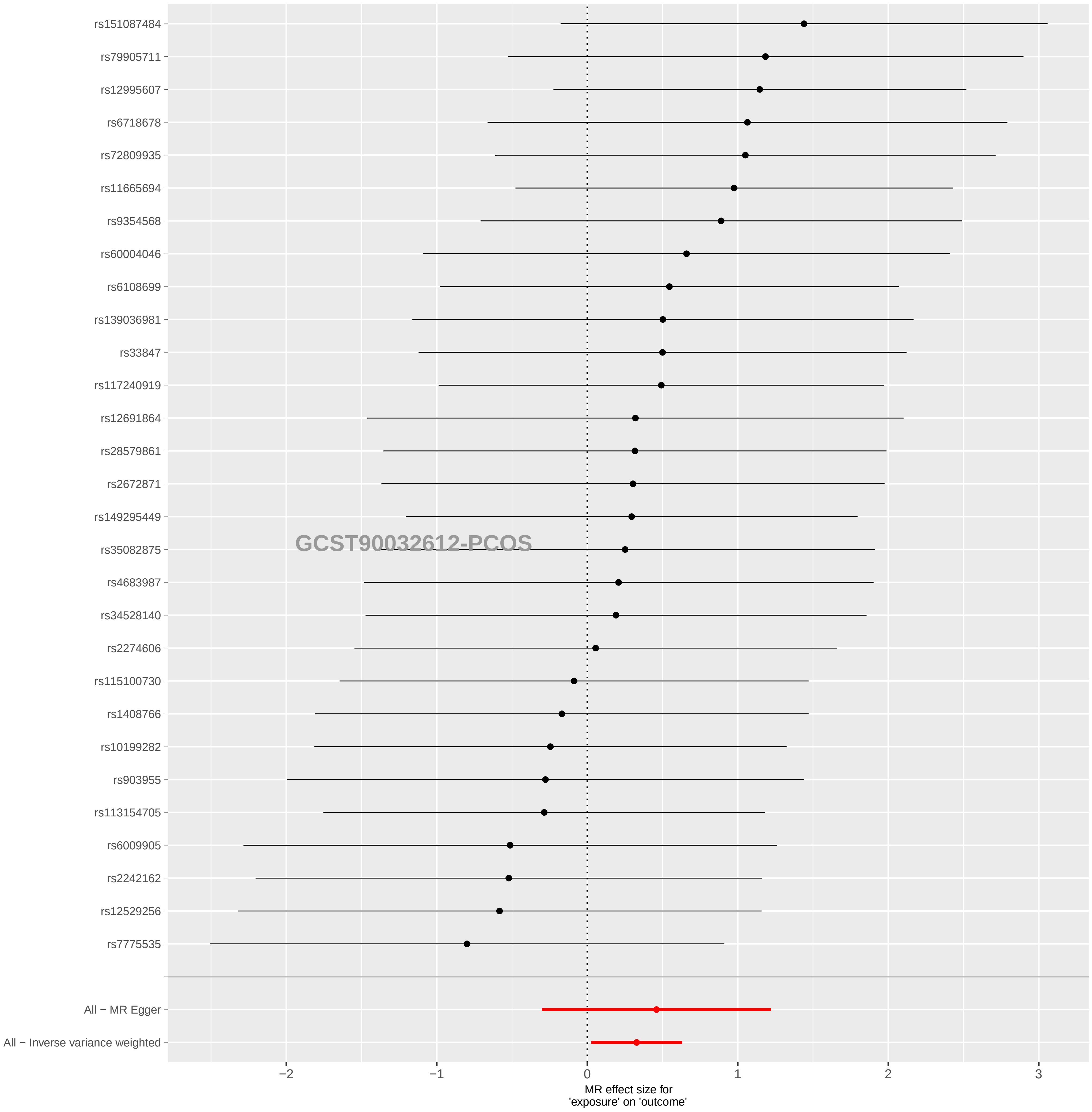
rs151087484
rs79905711
rs12995607
rs6718678
rs72809935
rs11665694
rs9354568
rs60004046
rs6108699
rs139036981
rs33847
rs117240919
rs12691864
rs28579861
rs2672871
rs149295449
GCST90032612-PCOS
rs35082875
rs4683987
rs34528140
rs2274606
rs115100730
rs1408766
rs10199282
rs903955
rs113154705
rs6009905
rs2242162
rs12529256
rs7775535
All − MR Egger
All − Inverse variance weighted
−2
−1
0
1
2
3
MR effect size for
'exposure' on 'outcome'

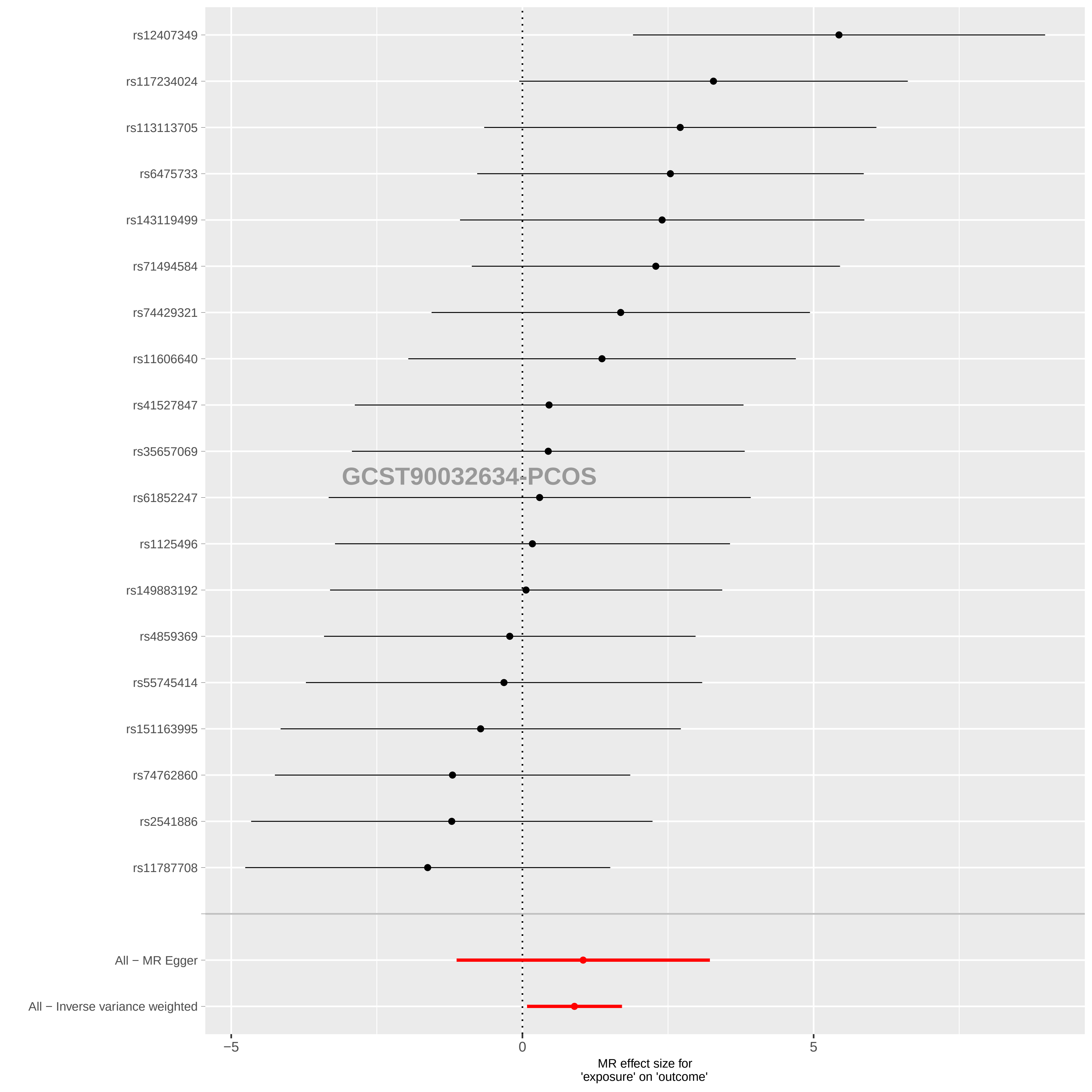

GCST90032634-PCOS
rs12407349
rs117234024
rs113113705
rs6475733
rs143119499
rs71494584
rs74429321
rs11606640
rs41527847
rs35657069
rs61852247
rs1125496
rs149883192
rs4859369
rs55745414
rs151163995
rs74762860
rs2541886
rs11787708
All − MR Egger
All − Inverse variance weighted
−5
0
5
MR effect size for
'exposure' on 'outcome'

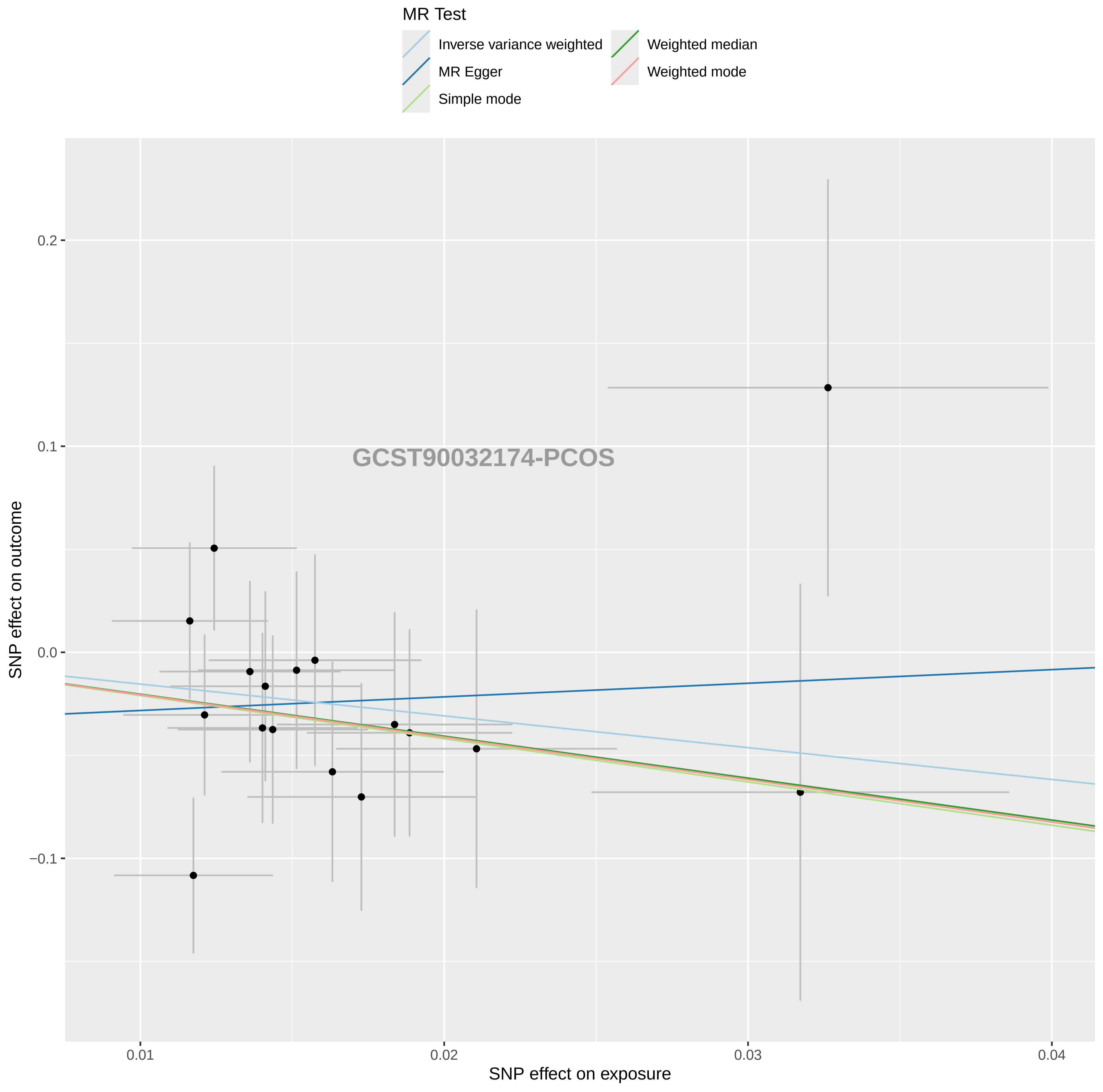

MR Test
Inverse variance weighted
MR Egger
Simple mode
Weighted median
Weighted mode
GCST90032174-PCOS
SNP effect on outcome
SNP effect on exposure
0.2
0.1
0.0
−0.1
0.01
0.02
0.03
0.04

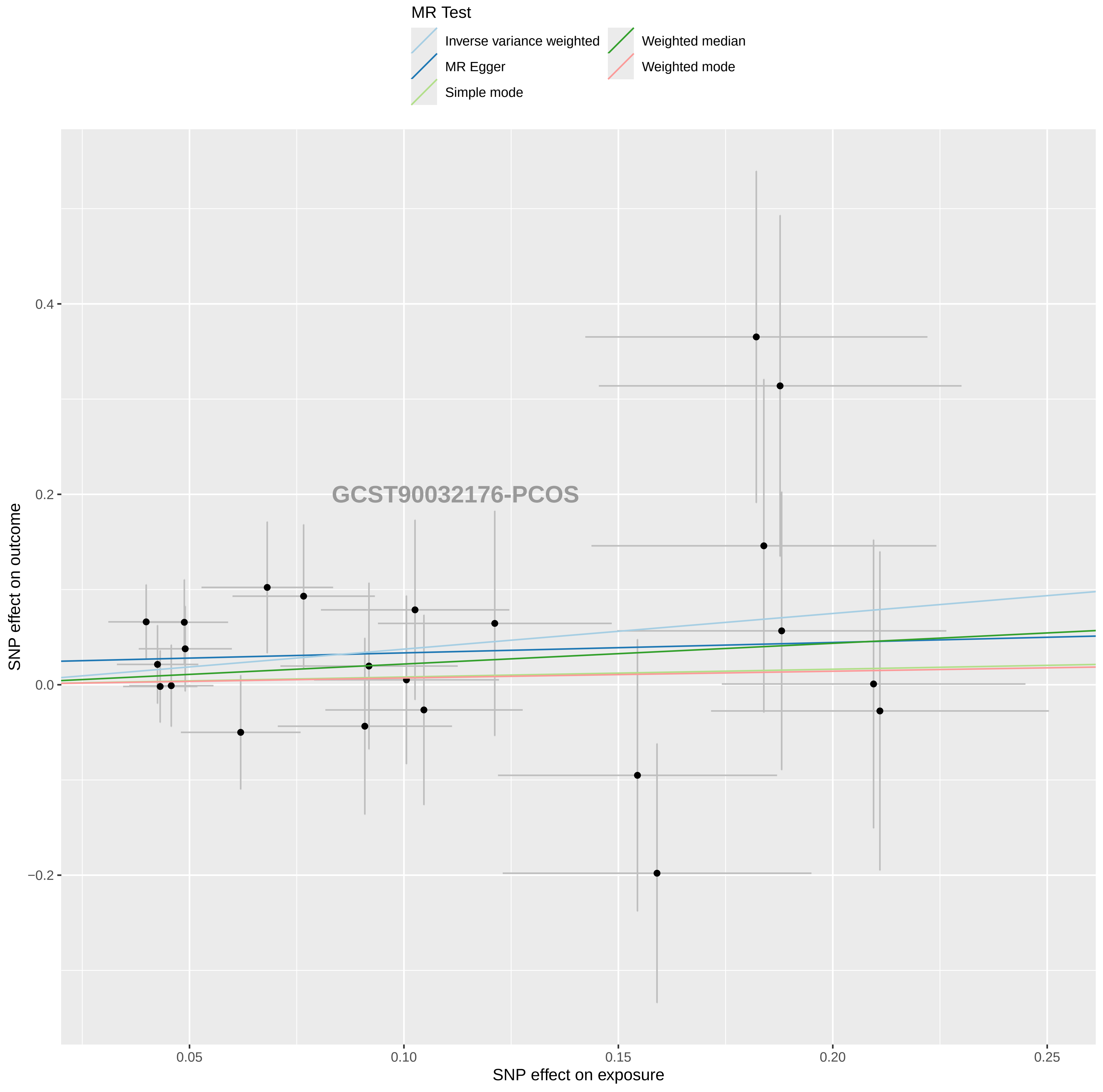

MR Test
Inverse variance weighted
MR Egger
Simple mode
Weighted median
Weighted mode
GCST90032176-PCOS
SNP effect on outcome
SNP effect on exposure
0.4
0.2
0.0
−0.2
0.05
0.10
0.15
0.20
0.25

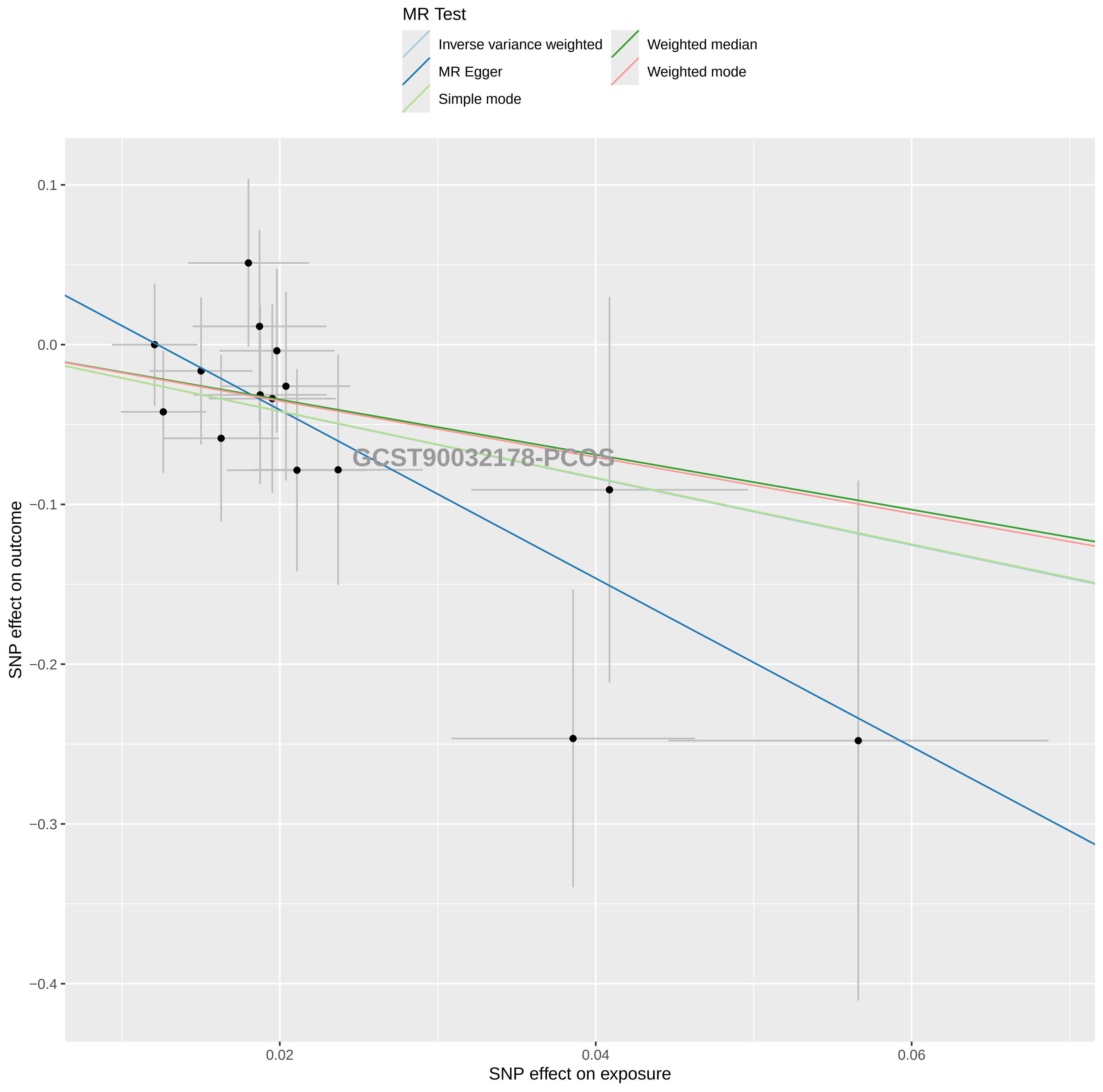

MR Test
Inverse variance weighted
MR Egger
Simple mode
Weighted median
Weighted mode
GCST90032178-PCOS
SNP effect on outcome
SNP effect on exposure
0.1
0.0
−0.1
−0.2
−0.3
−0.4
0.02
0.04
0.06

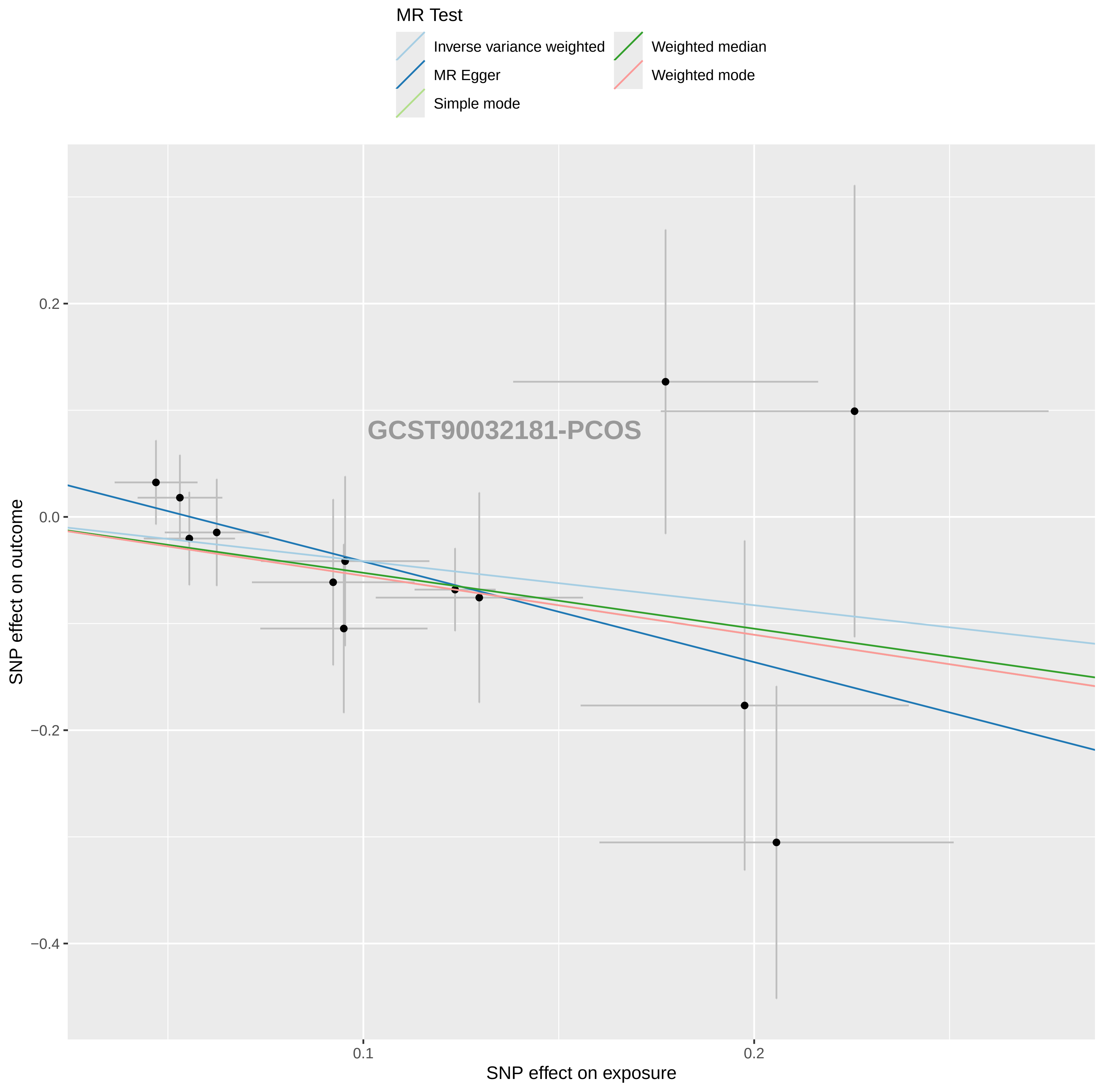

MR Test
Inverse variance weighted
MR Egger
Simple mode
Weighted median
Weighted mode
GCST90032181-PCOS
0.2
0.0
−0.2
−0.4
0.1
0.2
SNP effect on outcome
SNP effect on exposure

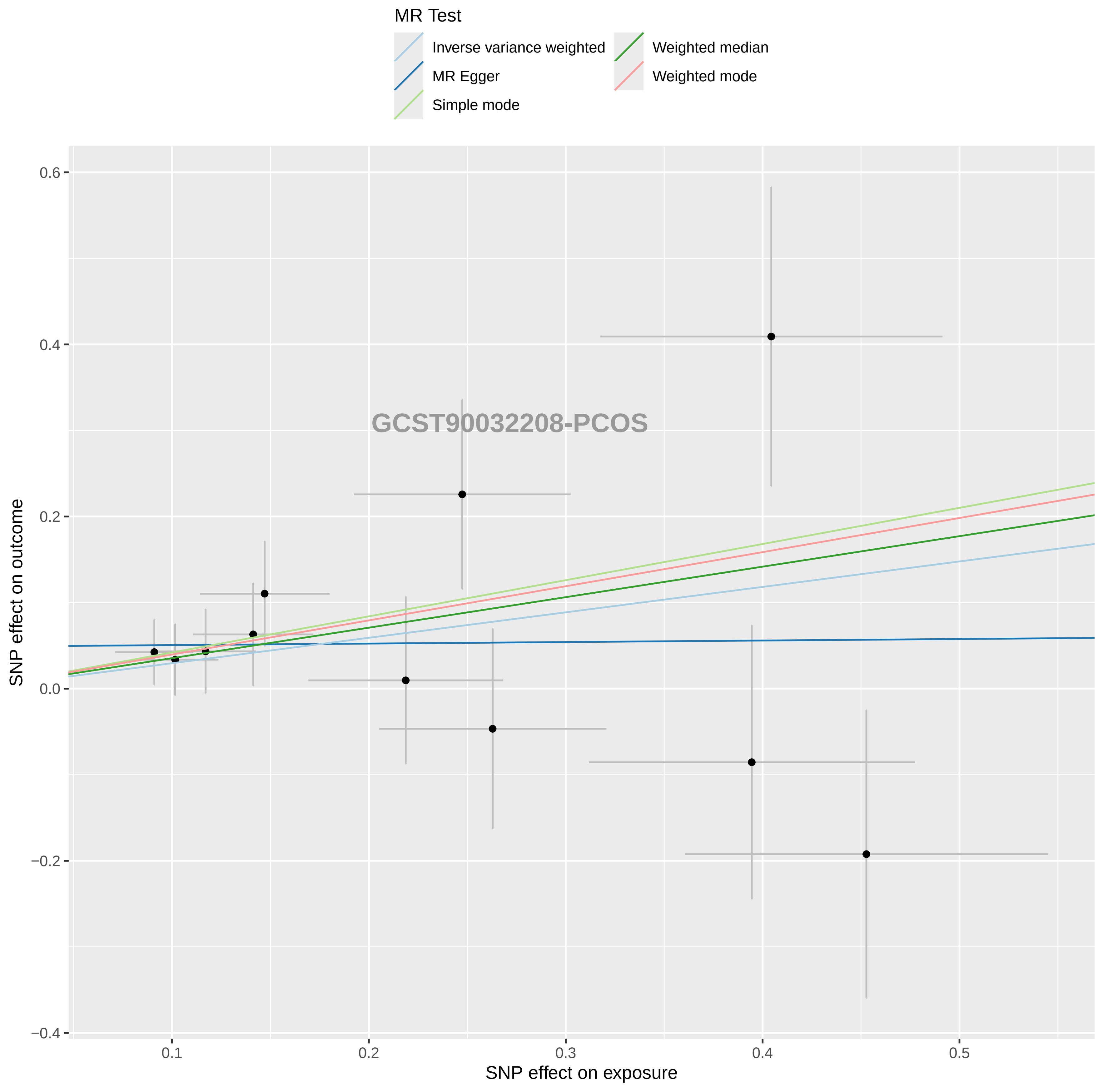
MR Test
Inverse variance weighted
MR Egger
Simple mode
Weighted median
Weighted mode
GCST90032208-PCOS
SNP effect on outcome
SNP effect on exposure
0.6
0.4
0.2
0.0
−0.2
−0.4
0.1
0.2
0.3
0.4
0.5

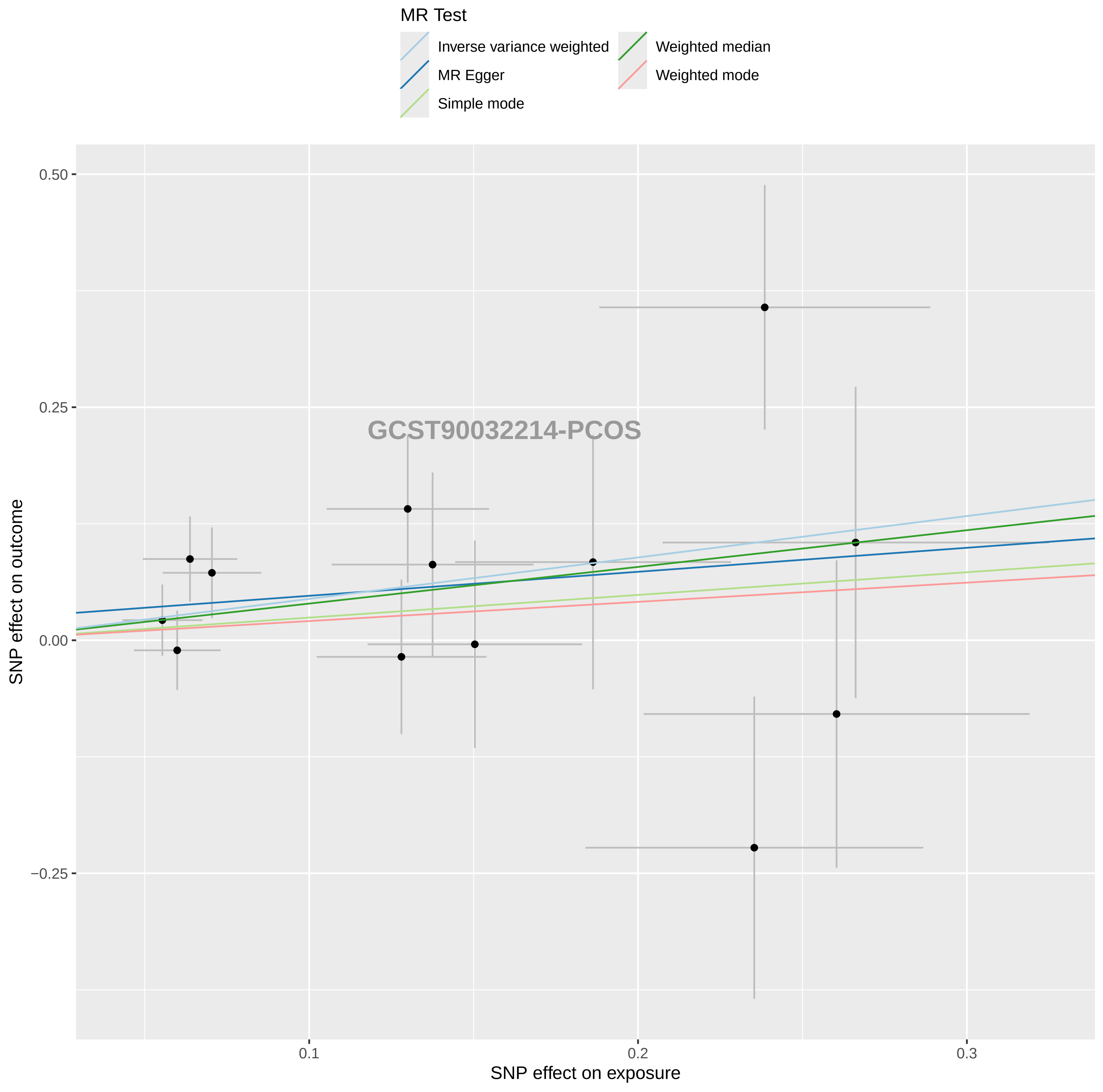
MR Test
Inverse variance weighted
MR Egger
Simple mode
Weighted median
Weighted mode
GCST90032214-PCOS
0.50
0.25
0.00
−0.25
0.1
0.2
0.3
SNP effect on outcome
SNP effect on exposure

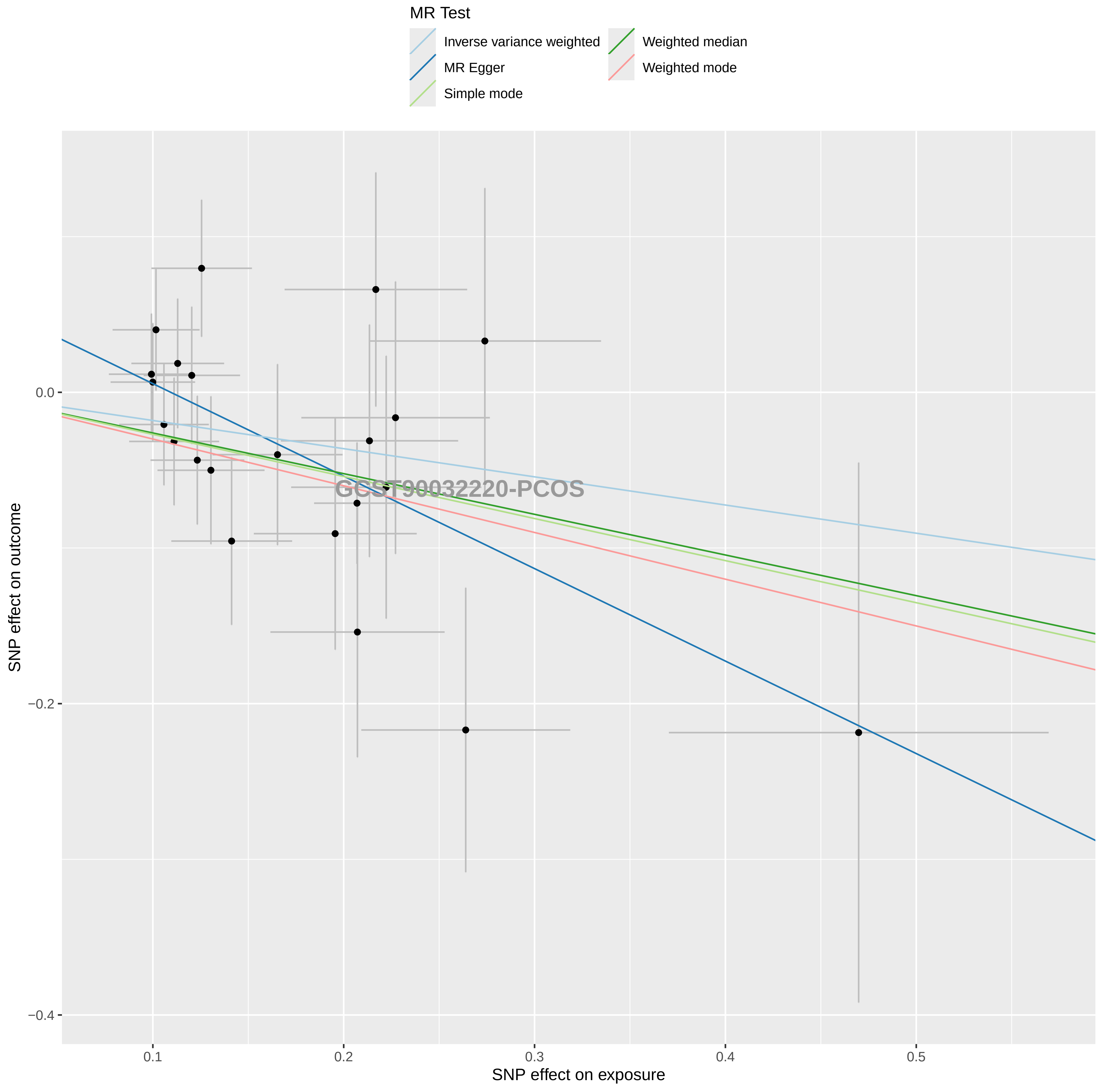
MR Test
Inverse variance weighted
MR Egger
Simple mode
Weighted median
Weighted mode
GCST90032220-PCOS
SNP effect on outcome
0.0
−0.2
−0.4
SNP effect on exposure
0.1
0.2
0.3
0.4
0.5

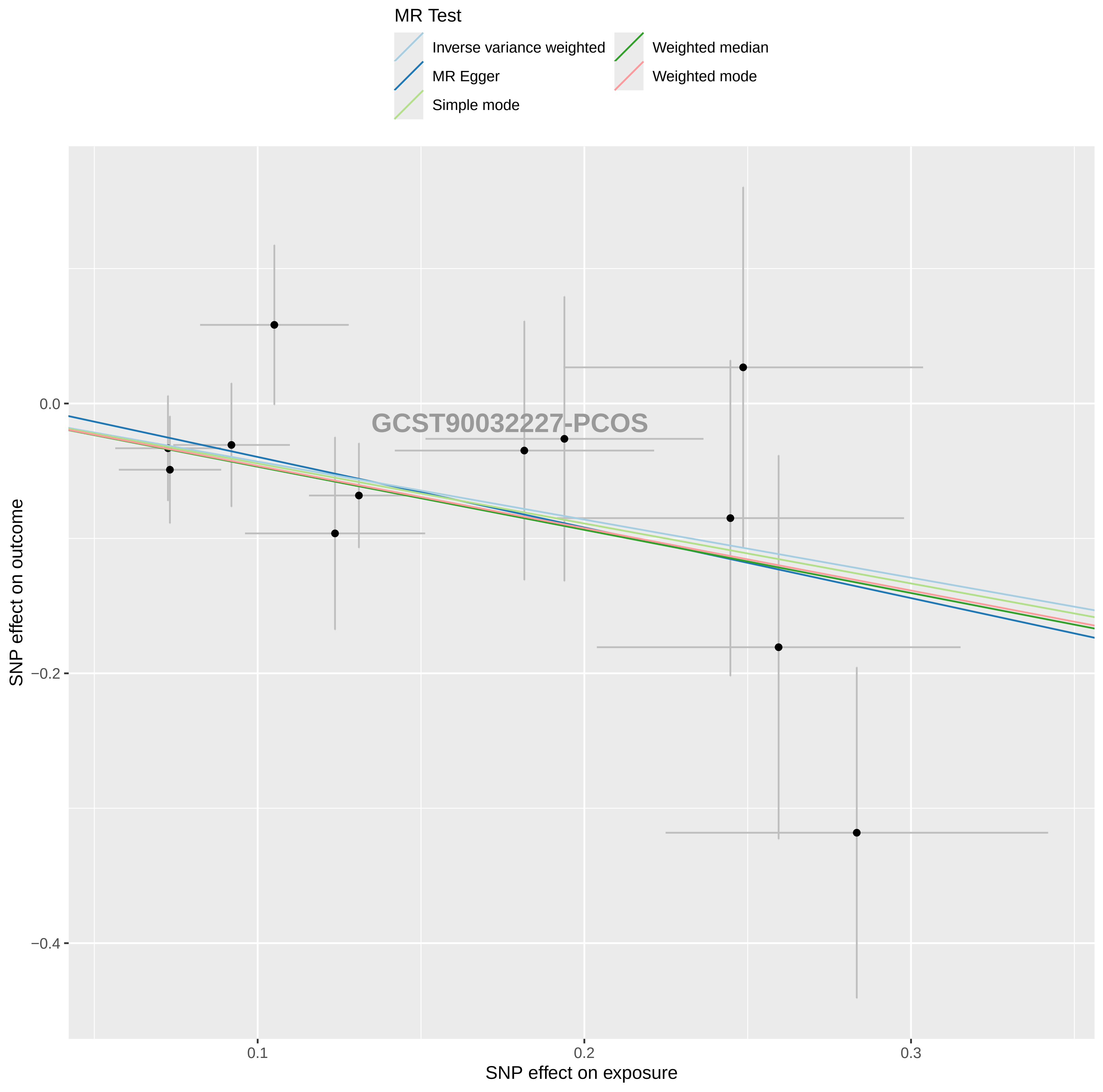
MR Test
Inverse variance weighted
MR Egger
Simple mode
Weighted median
Weighted mode
GCST90032227-PCOS
SNP effect on outcome
0.0
−0.2
−0.4
SNP effect on exposure
0.1
0.2
0.3

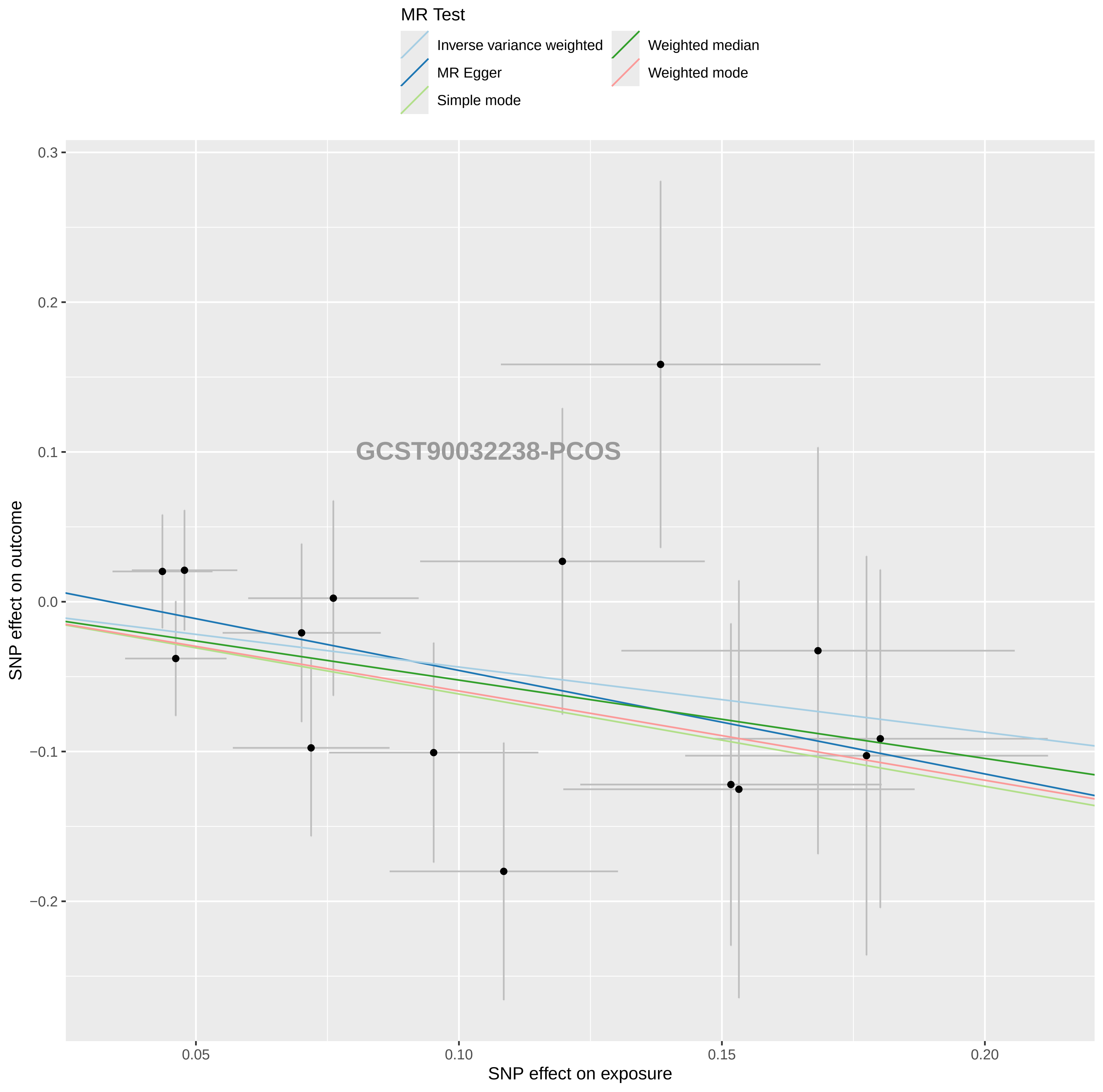

MR Test
Inverse variance weighted
MR Egger
Simple mode
Weighted median
Weighted mode
GCST90032238-PCOS
SNP effect on outcome
SNP effect on exposure
0.3
0.2
0.1
0.0
−0.1
−0.2
0.05
0.10
0.15
0.20

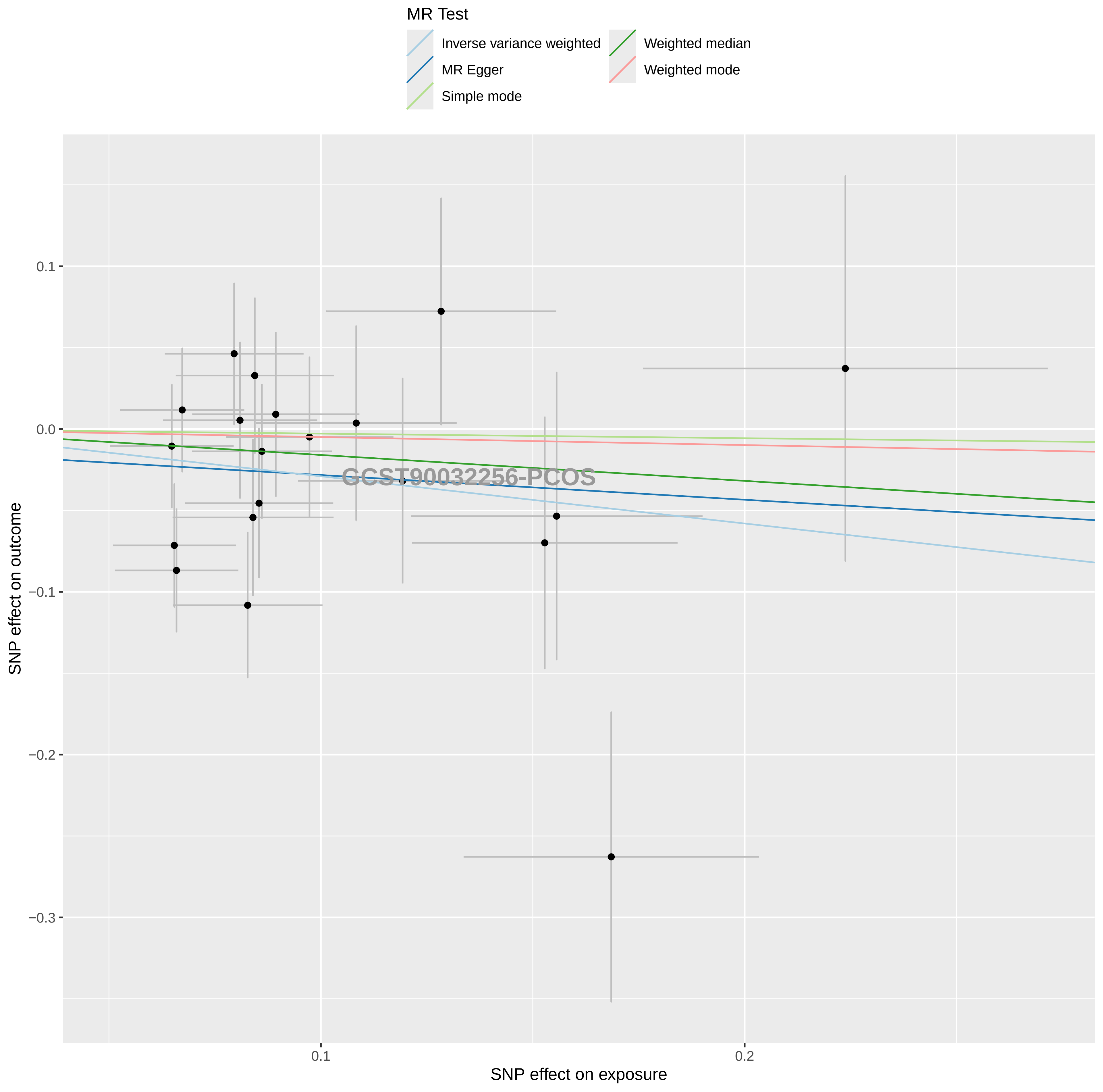
MR Test
Inverse variance weighted
MR Egger
Simple mode
Weighted median
Weighted mode
GCST90032256-PCOS
SNP effect on outcome
SNP effect on exposure
0.1
0.0
−0.1
−0.2
−0.3
0.1
0.2

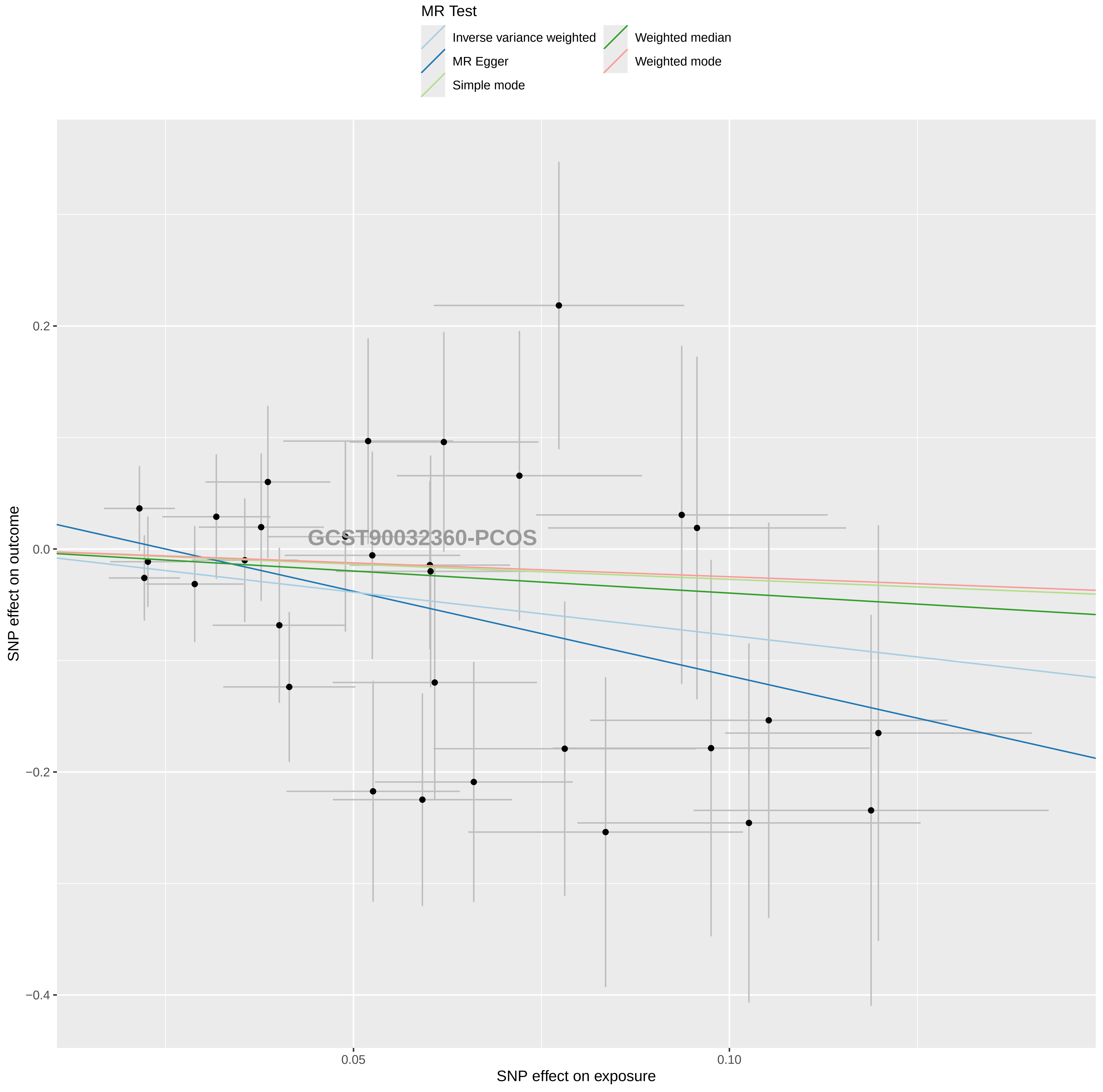
MR Test
Inverse variance weighted
MR Egger
Simple mode
Weighted median
Weighted mode
GCST90032360-PCOS
SNP effect on outcome
SNP effect on exposure
0.2
0.0
−0.2
−0.4
0.05
0.10

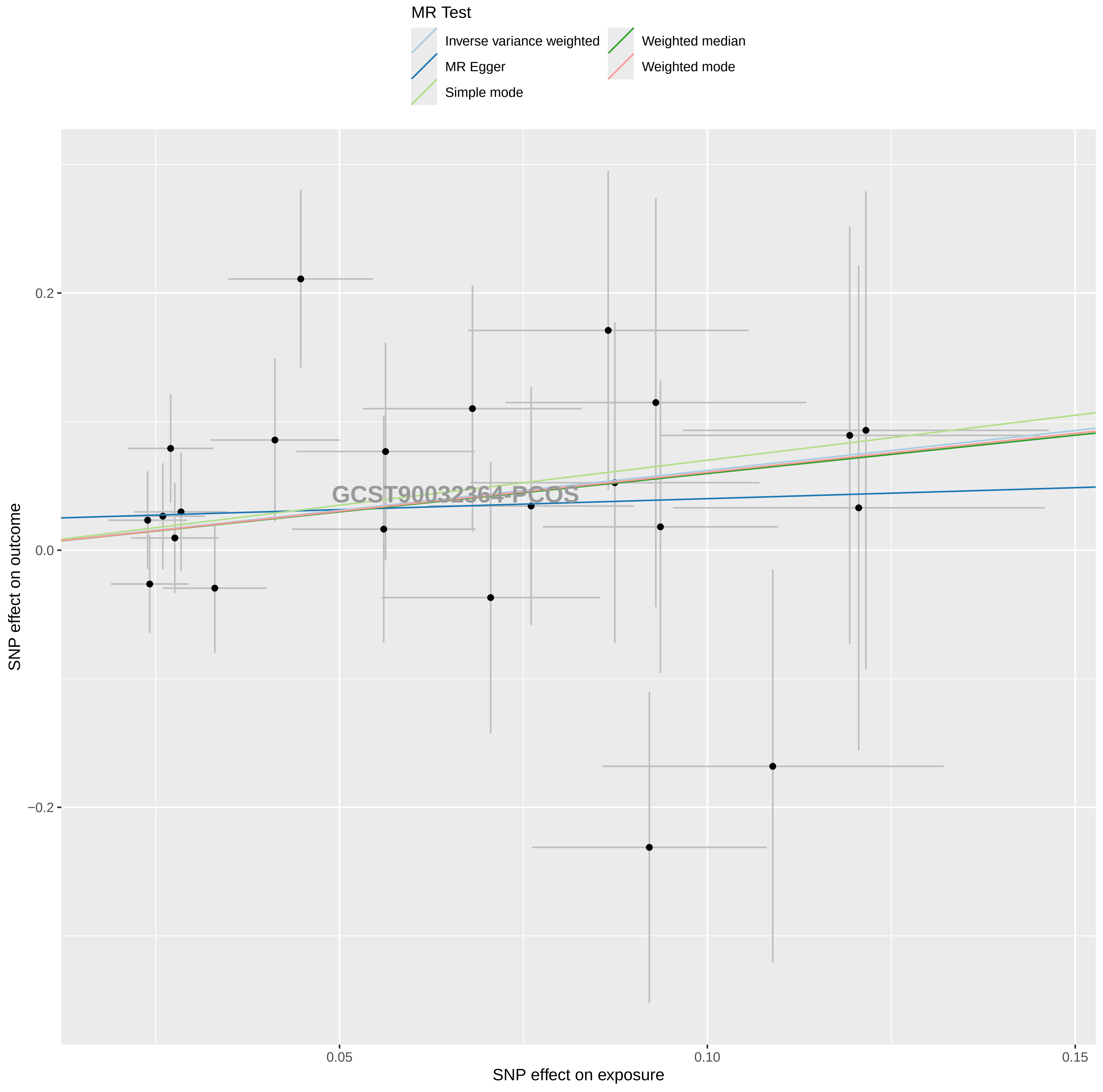
MR Test
Inverse variance weighted
MR Egger
Simple mode
Weighted median
Weighted mode
GCST90032364-PCOS
SNP effect on outcome
SNP effect on exposure
0.2
0.0
−0.2
0.05
0.10
0.15

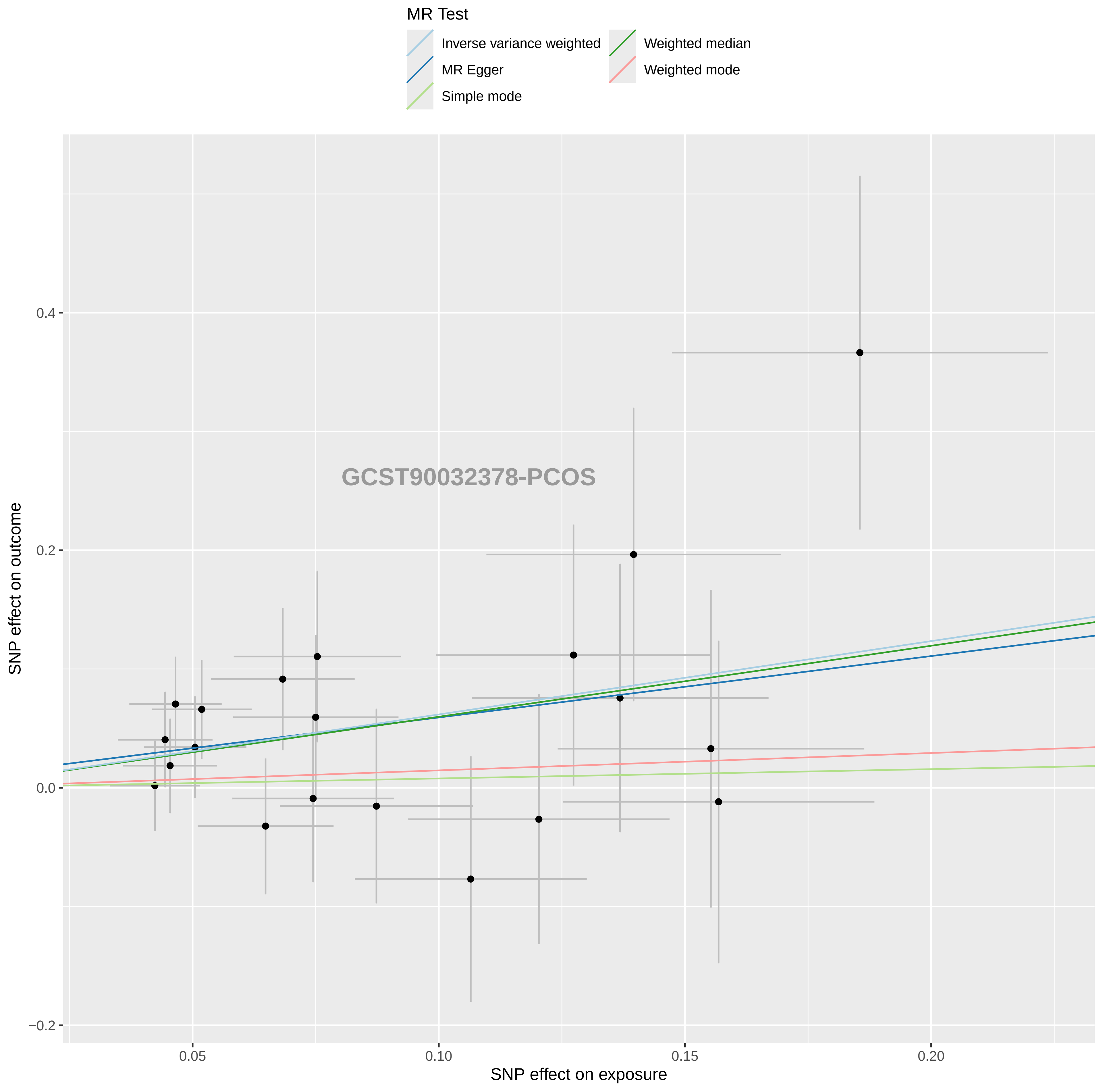

MR Test
Inverse variance weighted
MR Egger
Simple mode
Weighted median
Weighted mode
GCST90032378-PCOS
SNP effect on outcome
SNP effect on exposure
0.4
0.2
0.0
−0.2
0.05
0.10
0.15
0.20

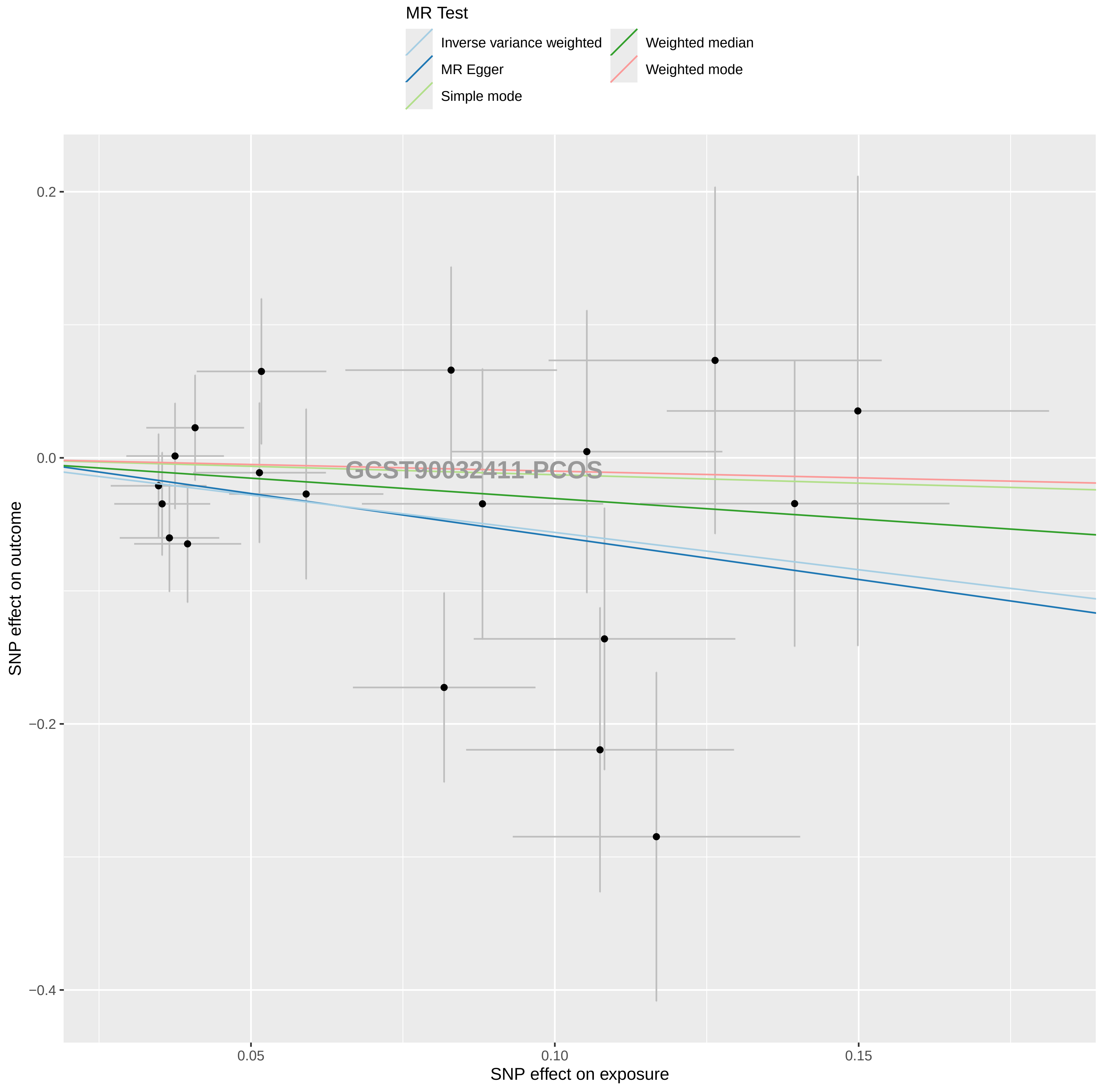

MR Test
Inverse variance weighted
MR Egger
Simple mode
Weighted median
Weighted mode
GCST90032411-PCOS
SNP effect on outcome
SNP effect on exposure
0.2
0.0
−0.2
−0.4
0.05
0.10
0.15

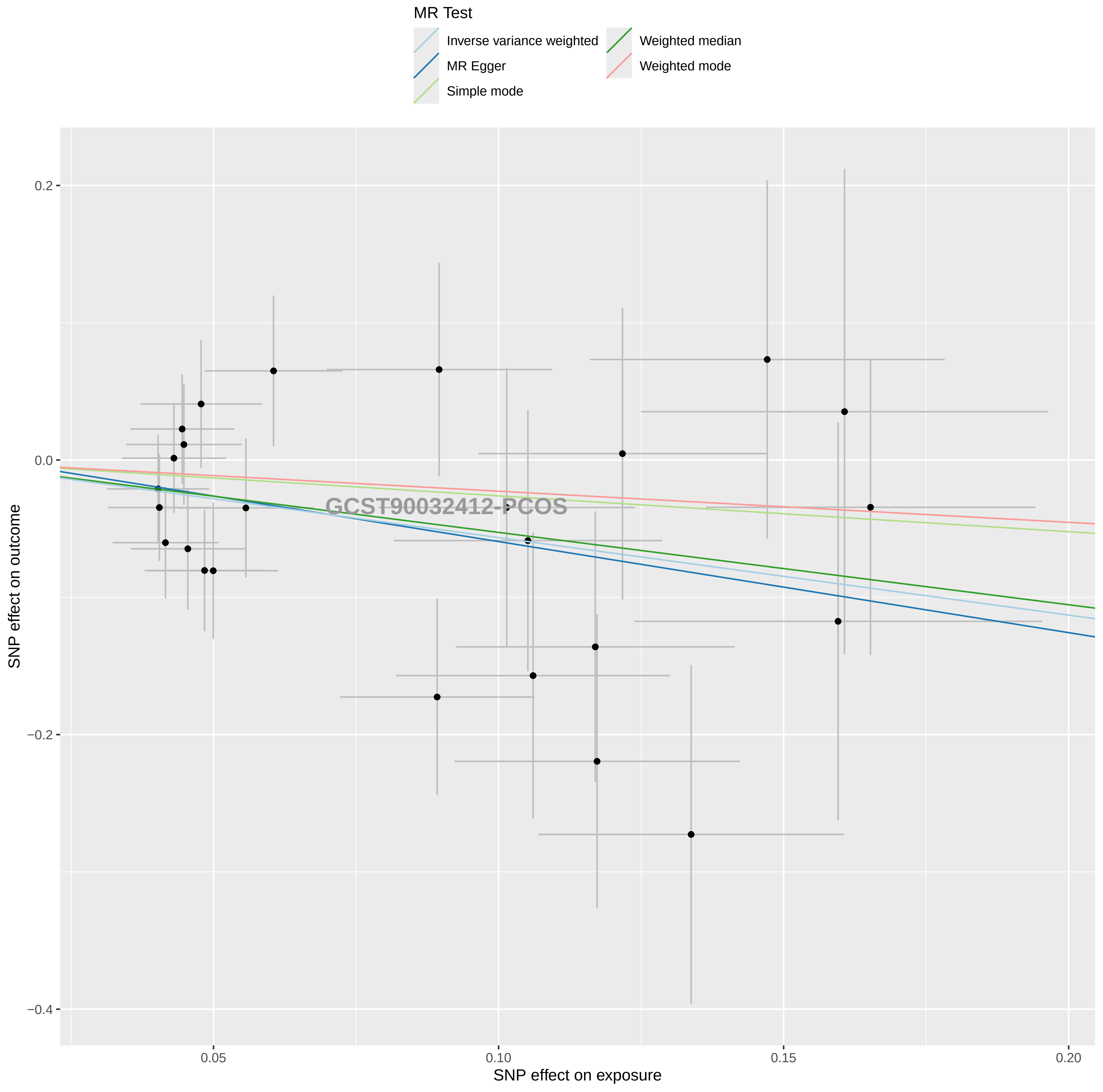

MR Test
Inverse variance weighted
MR Egger
Simple mode
Weighted median
Weighted mode
GCST90032412-PCOS
SNP effect on outcome
SNP effect on exposure
0.2
0.0
−0.2
−0.4
0.05
0.10
0.15
0.20

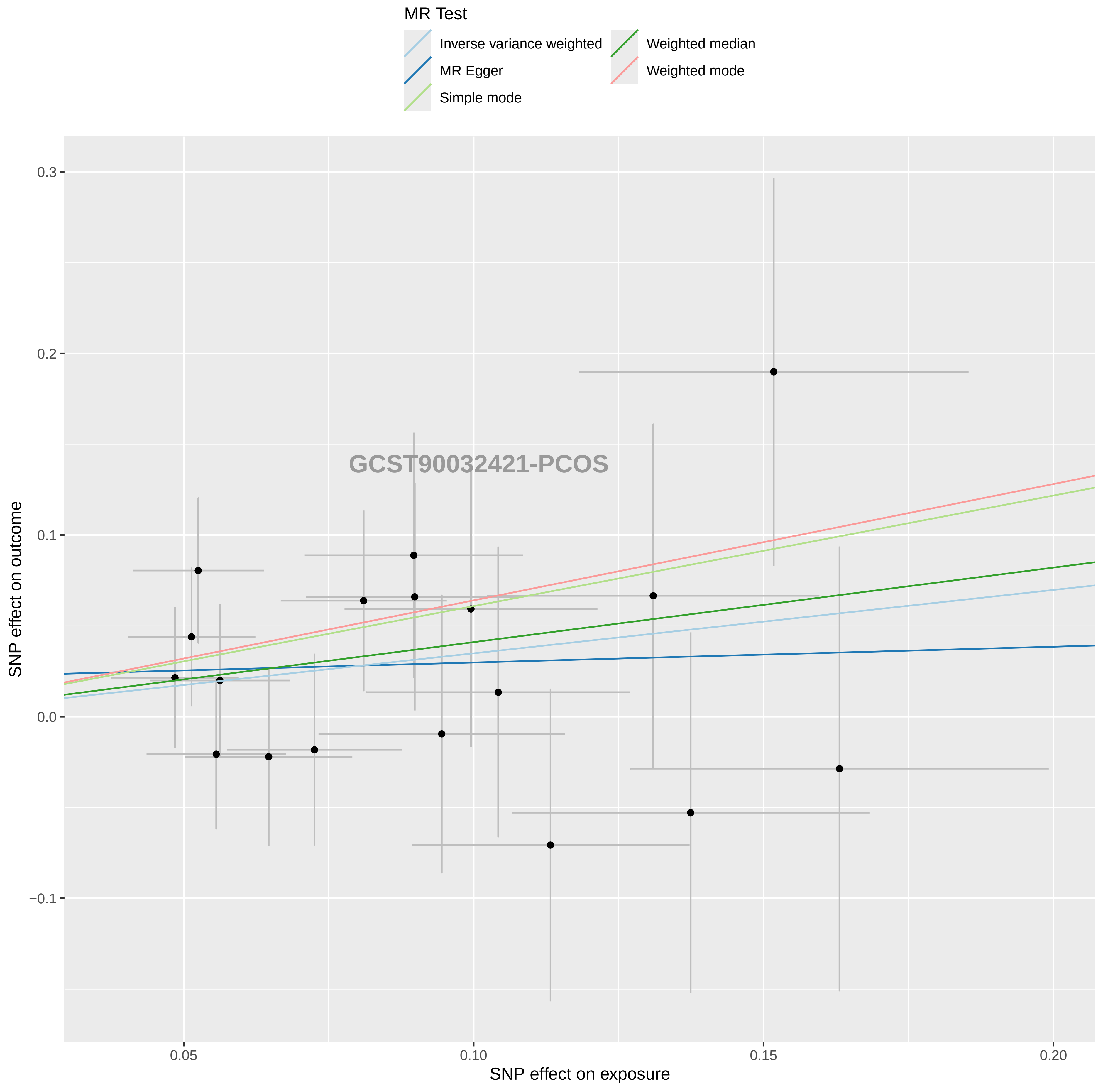
MR Test
Inverse variance weighted
MR Egger
Simple mode
Weighted median
Weighted mode
GCST90032421-PCOS
SNP effect on outcome
SNP effect on exposure
0.3
0.2
0.1
0.0
−0.1
0.05
0.10
0.15
0.20

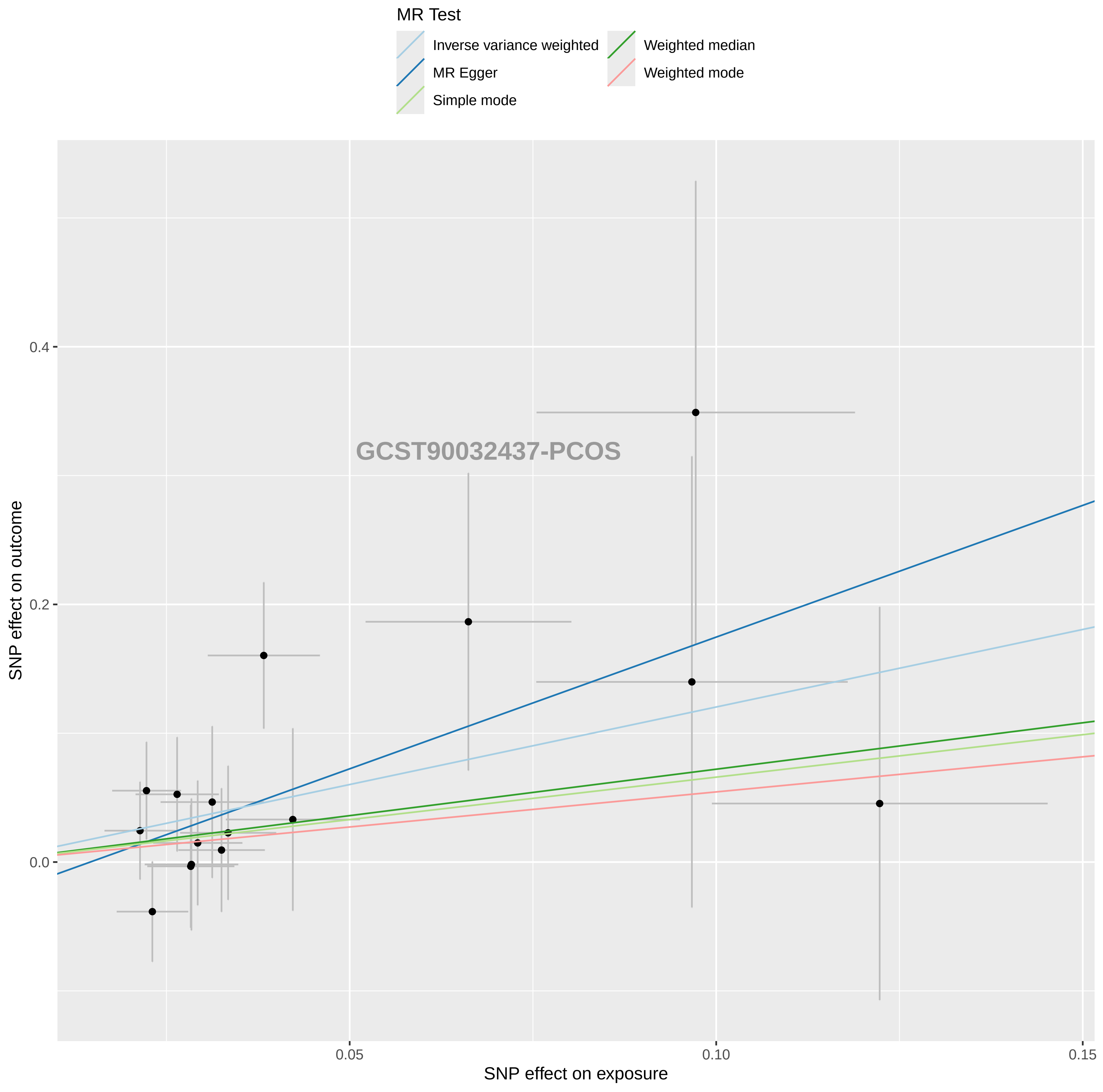
MR Test
Inverse variance weighted
MR Egger
Simple mode
Weighted median
Weighted mode
GCST90032437-PCOS
SNP effect on outcome
0.4
0.2
0.0
SNP effect on exposure
0.05
0.10
0.15

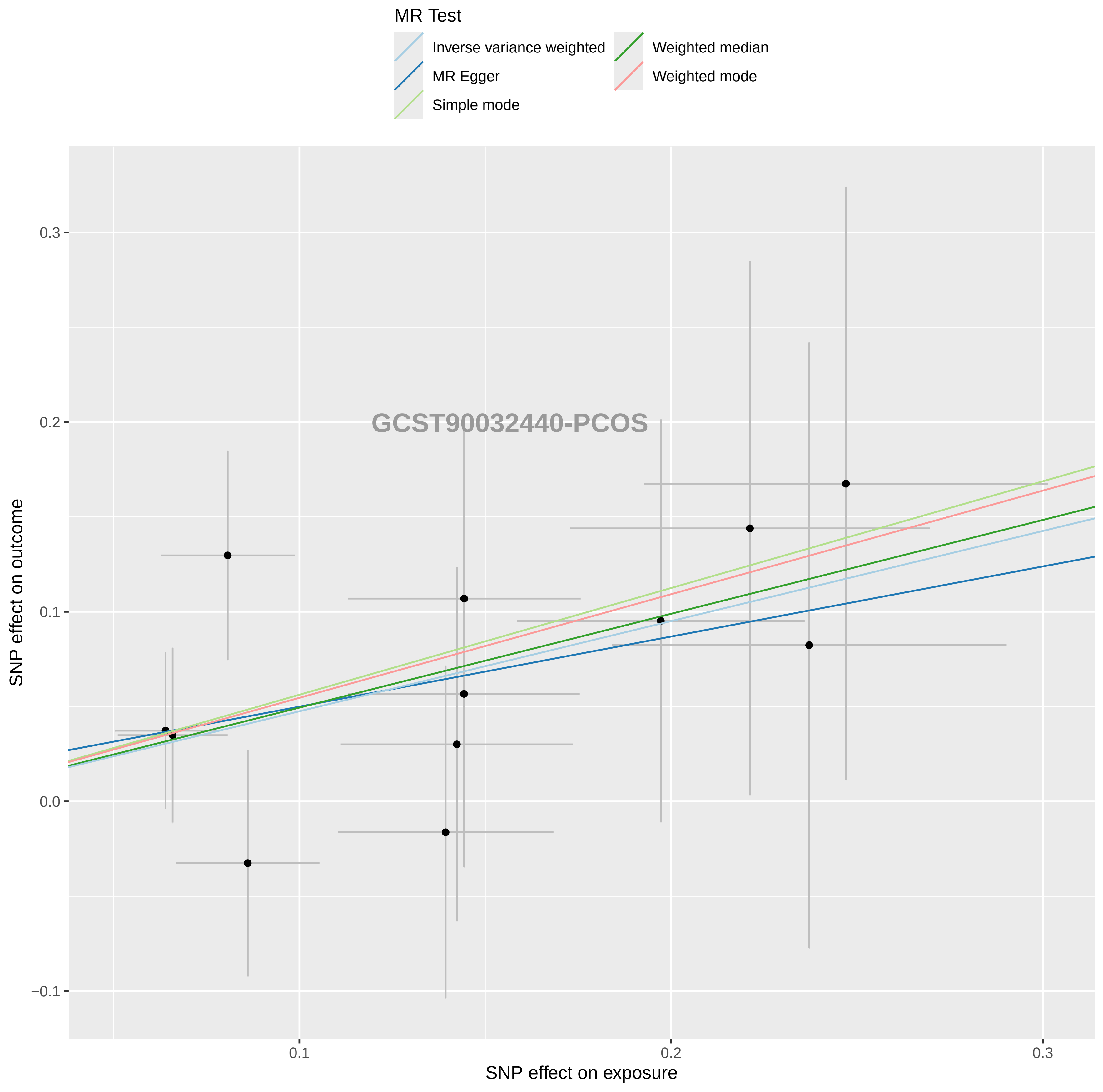
MR Test
Inverse variance weighted
MR Egger
Simple mode
Weighted median
Weighted mode
GCST90032440-PCOS
SNP effect on outcome
SNP effect on exposure
0.3
0.2
0.1
0.0
−0.1
0.1
0.2
0.3

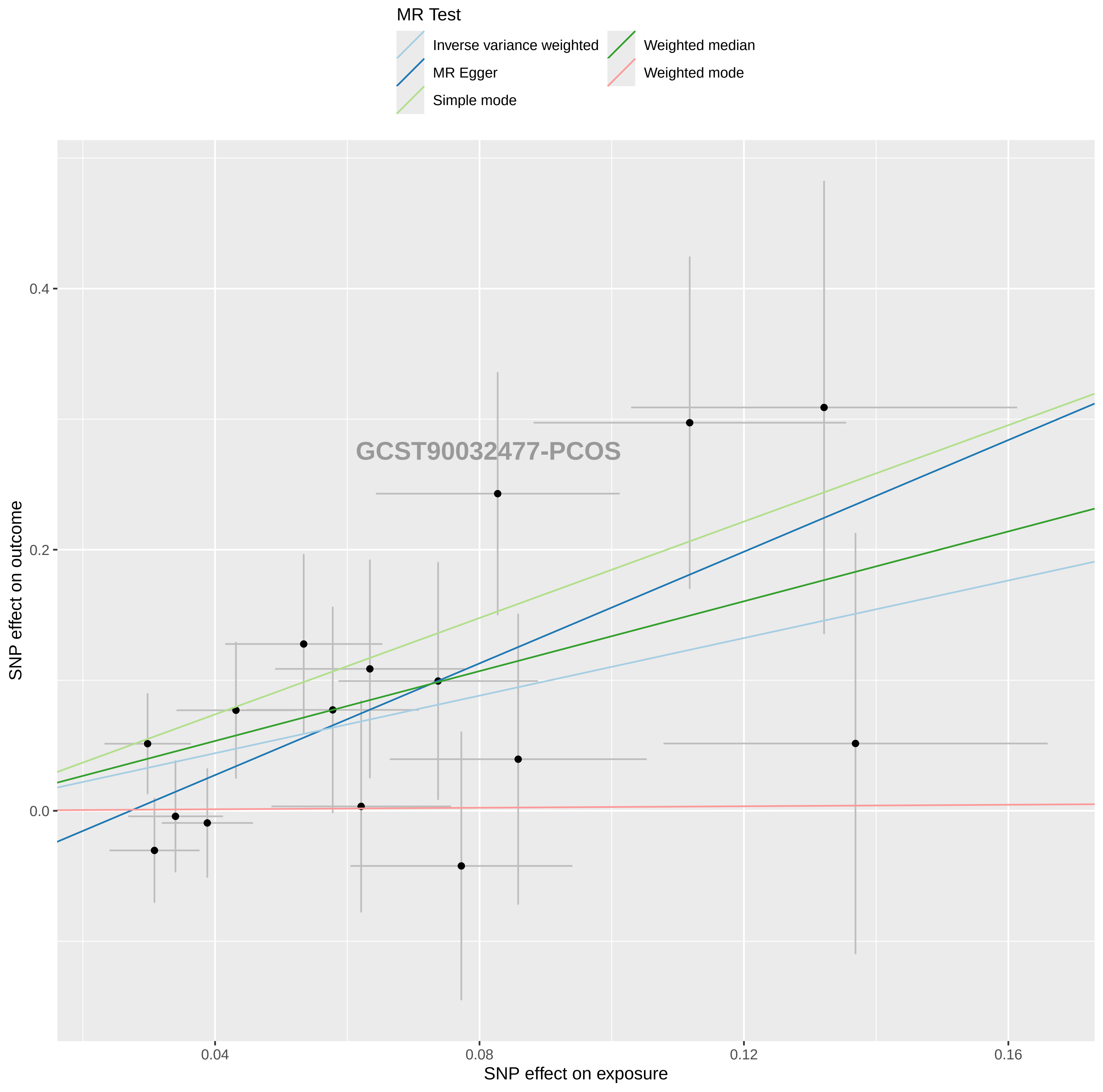

MR Test
Inverse variance weighted
MR Egger
Simple mode
Weighted median
Weighted mode
GCST90032477-PCOS
SNP effect on outcome
SNP effect on exposure
0.4
0.2
0.0
0.04
0.08
0.12
0.16

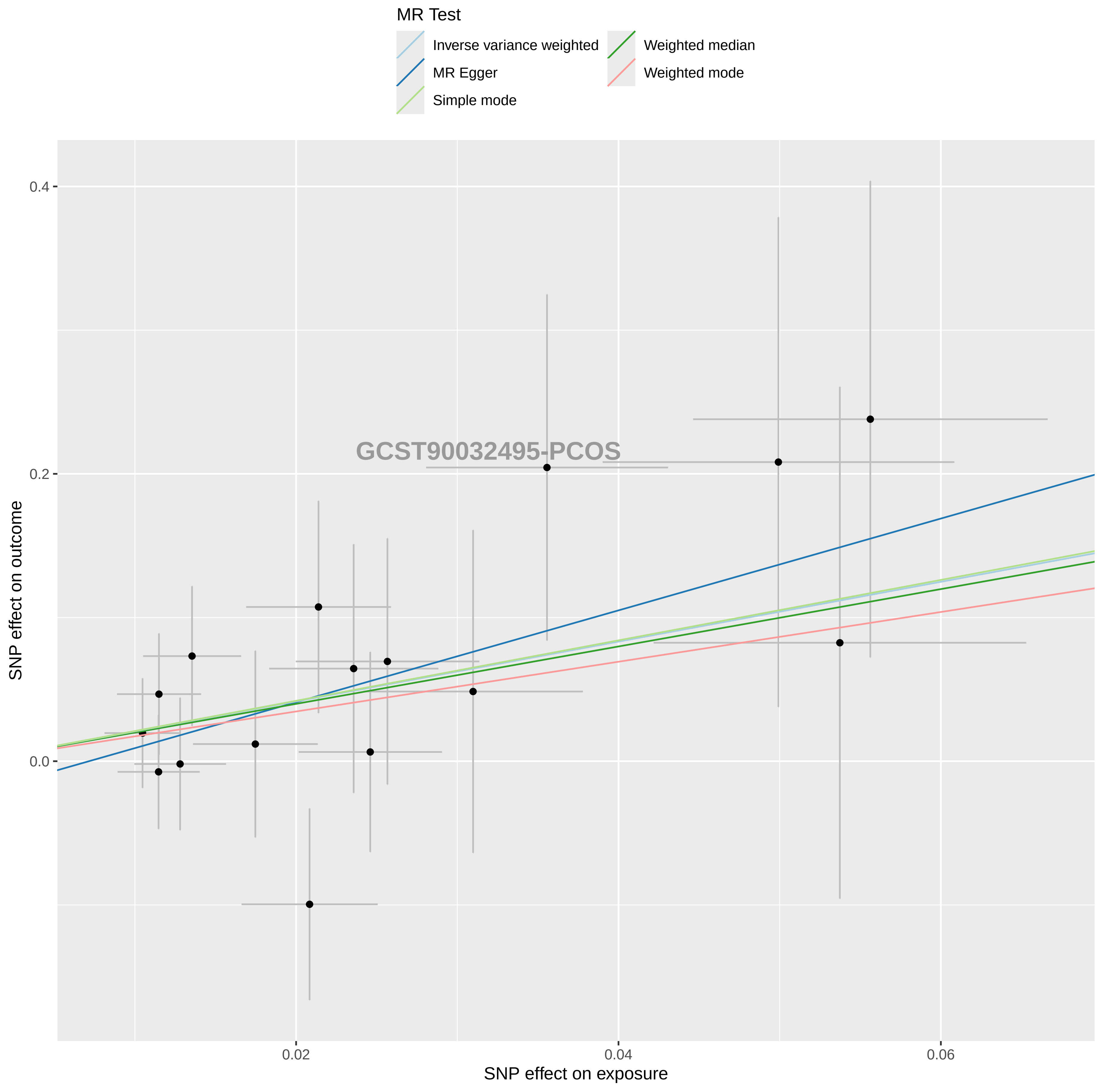
MR Test
Inverse variance weighted
MR Egger
Simple mode
Weighted median
Weighted mode
GCST90032495-PCOS
0.4
0.2
0.0
0.02
0.04
0.06
SNP effect on outcome
SNP effect on exposure

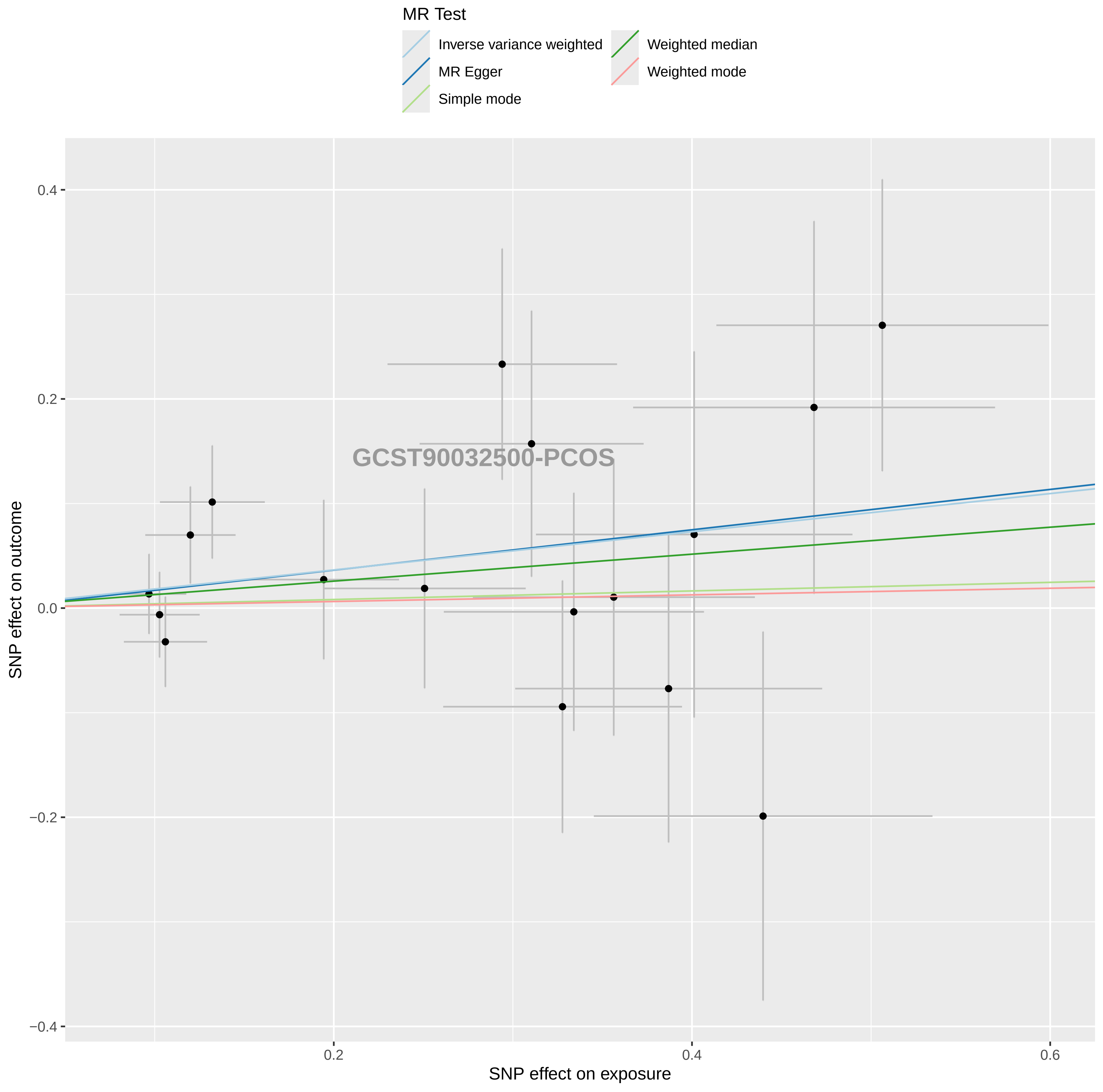
MR Test
Inverse variance weighted
MR Egger
Simple mode
Weighted median
Weighted mode
GCST90032500-PCOS
SNP effect on outcome
SNP effect on exposure
0.4
0.2
0.0
−0.2
−0.4
0.2
0.4
0.6

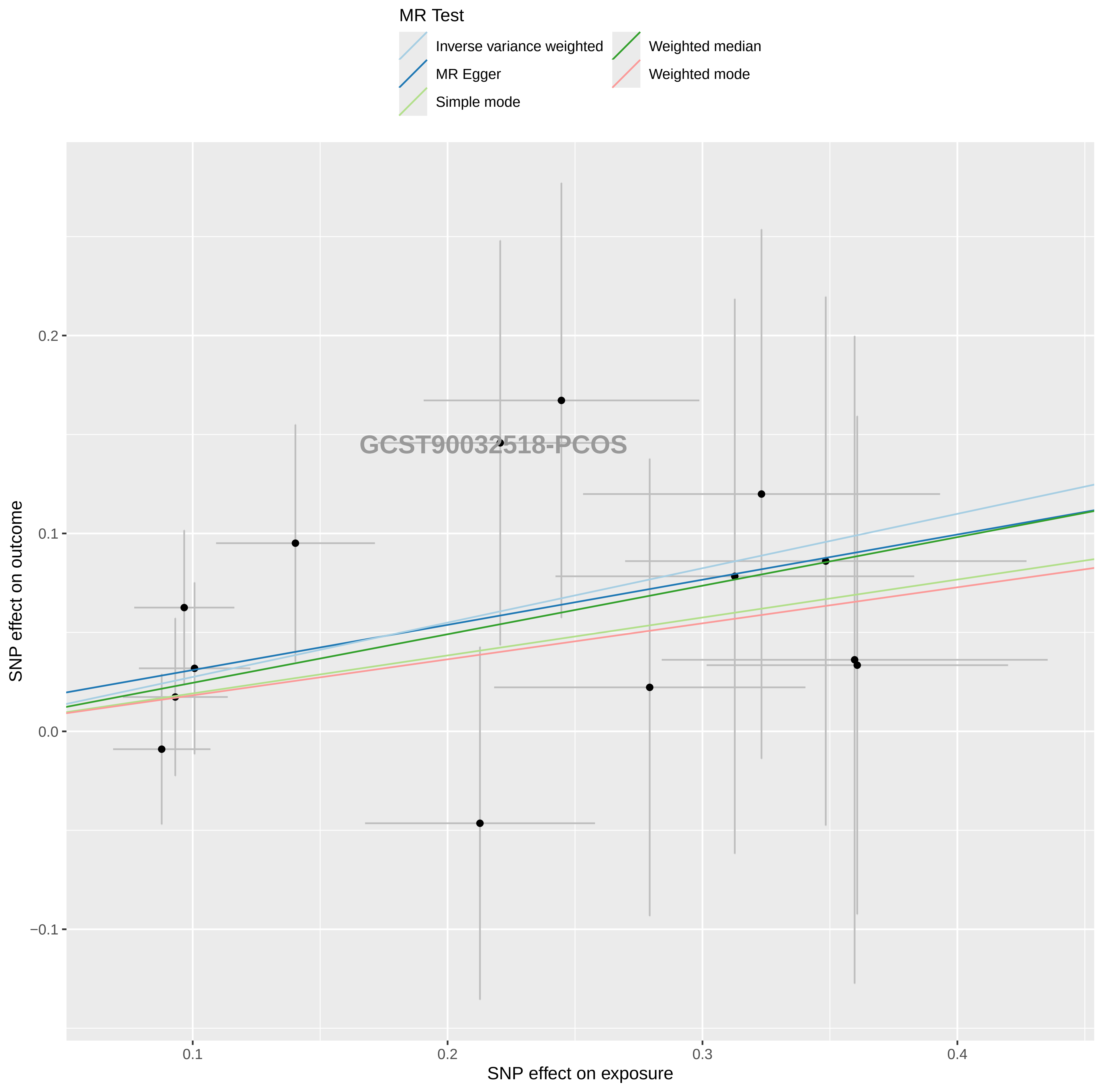
MR Test
Inverse variance weighted
MR Egger
Simple mode
Weighted median
Weighted mode
GCST90032518-PCOS
SNP effect on outcome
SNP effect on exposure
0.2
0.1
0.0
−0.1
0.1
0.2
0.3
0.4

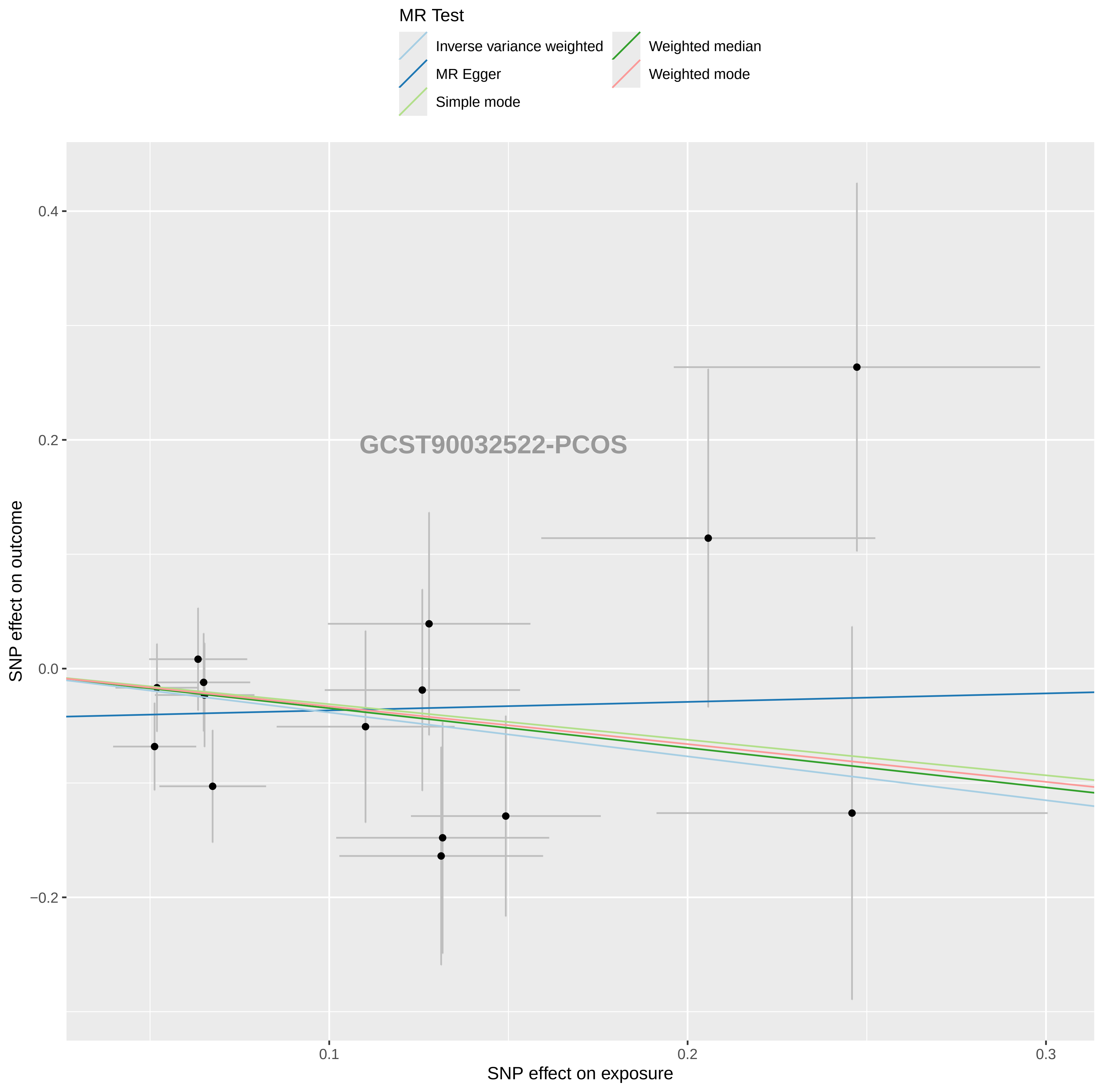

MR Test
Inverse variance weighted
MR Egger
Simple mode
Weighted median
Weighted mode
GCST90032522-PCOS
SNP effect on outcome
0.4
0.2
0.0
−0.2
SNP effect on exposure
0.1
0.2
0.3

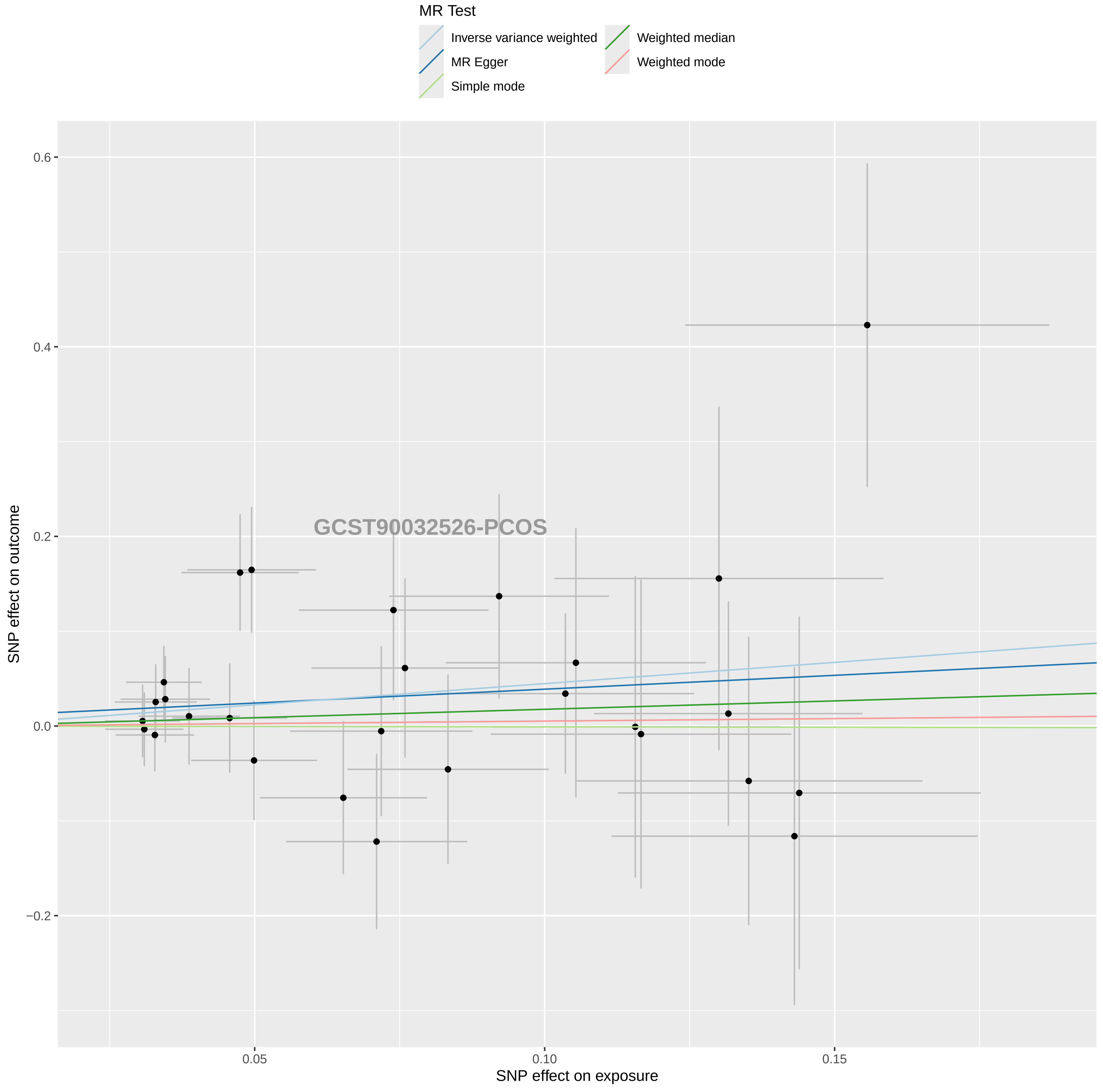
MR Test
Inverse variance weighted
MR Egger
Simple mode
Weighted median
Weighted mode
GCST90032526-PCOS
SNP effect on outcome
SNP effect on exposure
0.6
0.4
0.2
0.0
−0.2
0.05
0.10
0.15

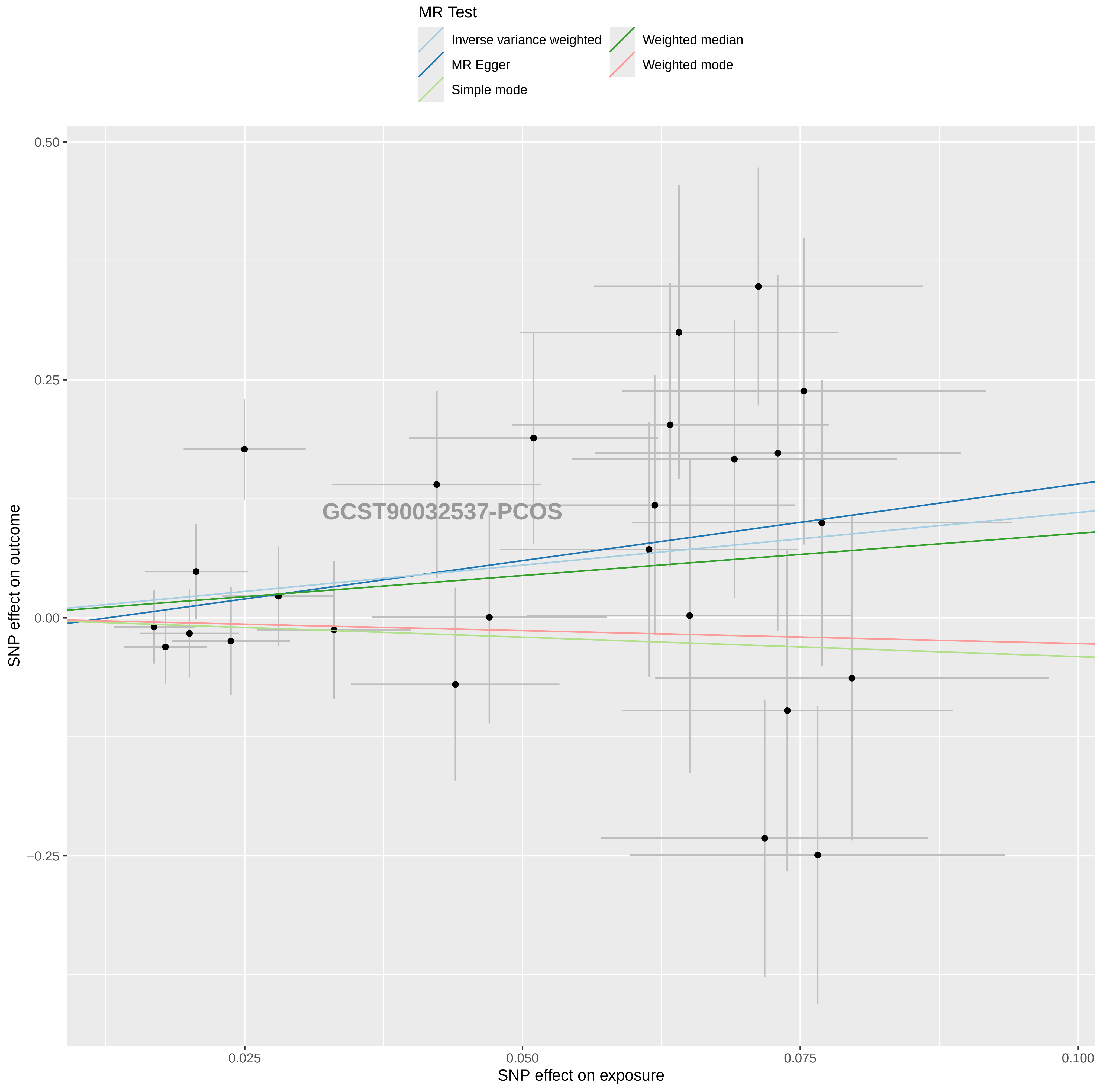
MR Test
Inverse variance weighted
MR Egger
Simple mode
Weighted median
Weighted mode
GCST90032537-PCOS
0.50
0.25
0.00
−0.25
0.025
0.050
0.075
0.100
SNP effect on outcome
SNP effect on exposure

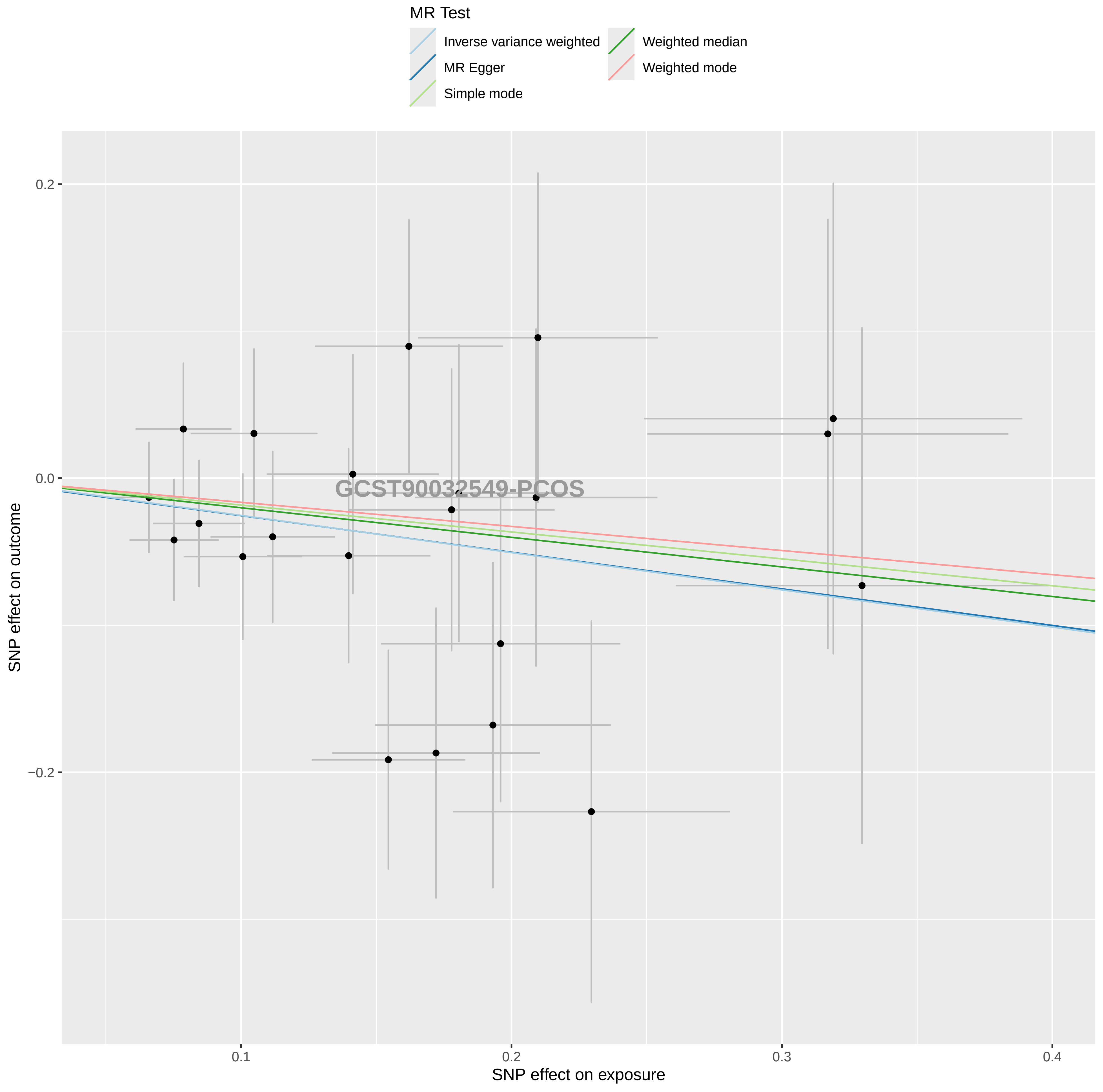
MR Test
Inverse variance weighted
MR Egger
Simple mode
Weighted median
Weighted mode
GCST90032549-PCOS
0.2
0.0
−0.2
0.1
0.2
0.3
0.4
SNP effect on outcome
SNP effect on exposure

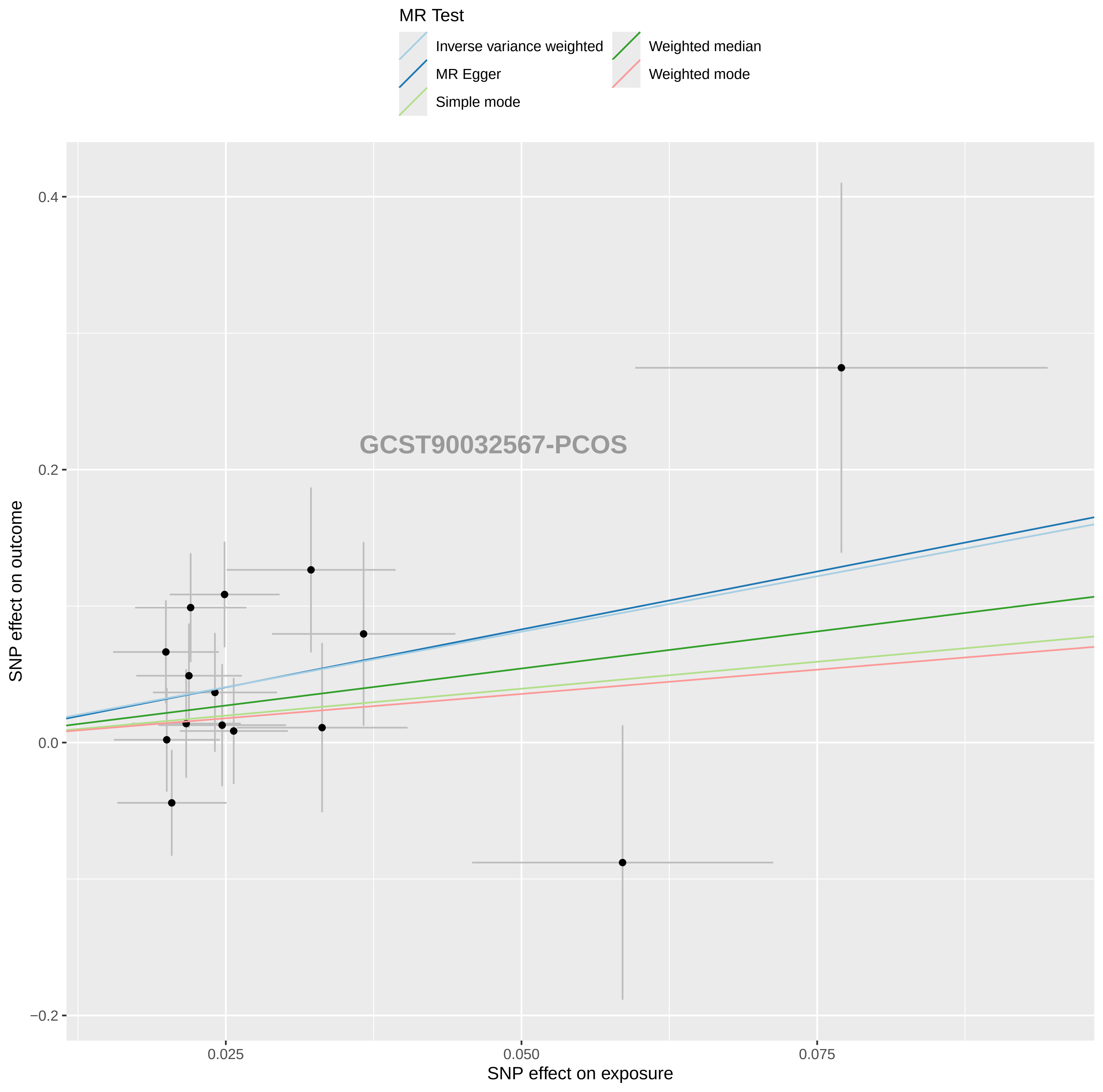

MR Test
Inverse variance weighted
MR Egger
Simple mode
Weighted median
Weighted mode
GCST90032567-PCOS
0.4
0.2
0.0
−0.2
SNP effect on outcome
0.025
0.050
0.075
SNP effect on exposure

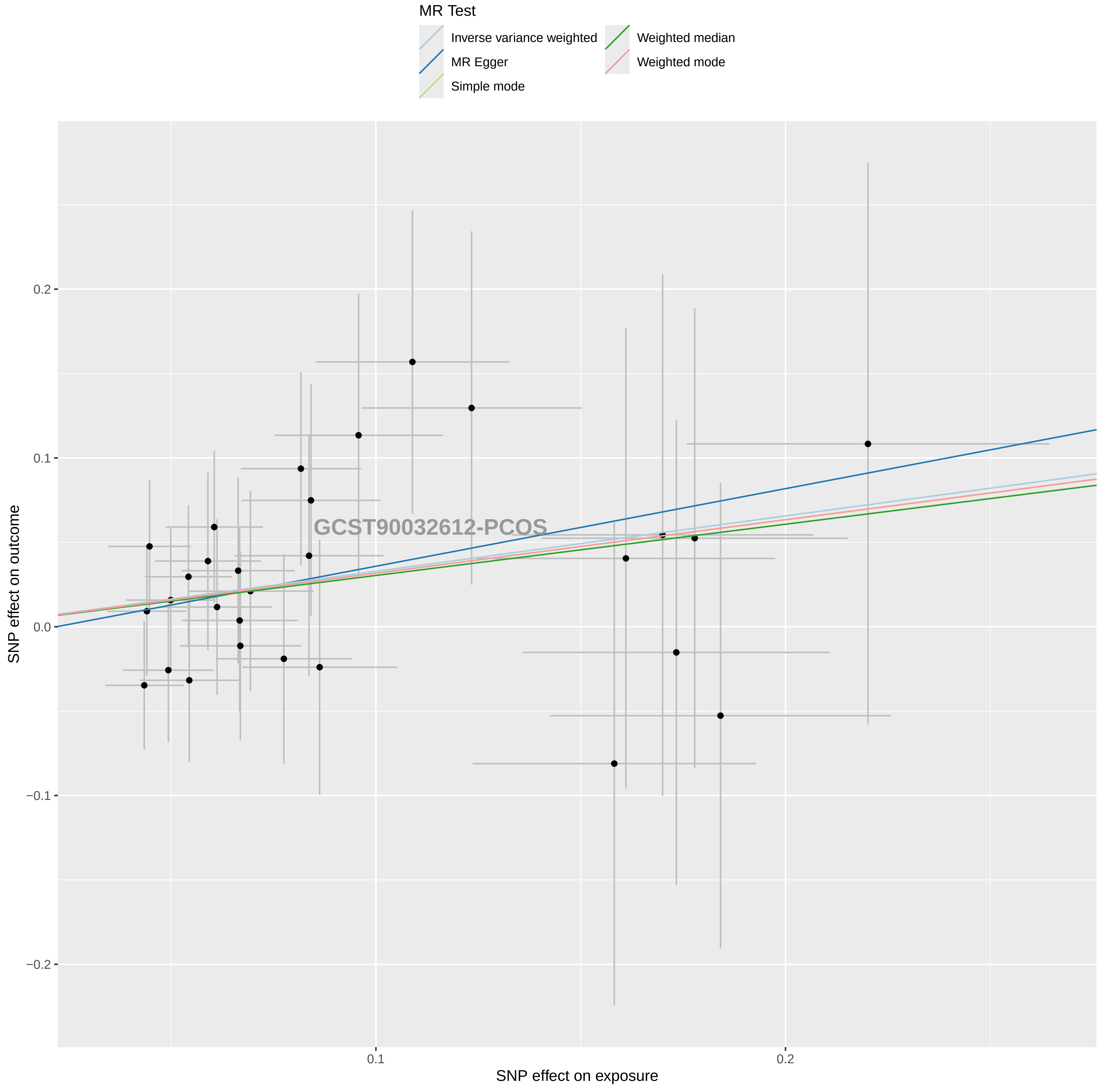
MR Test
Inverse variance weighted
MR Egger
Simple mode
Weighted median
Weighted mode
GCST90032612-PCOS
SNP effect on outcome
SNP effect on exposure
0.2
0.1
0.0
−0.1
−0.2
0.1
0.2

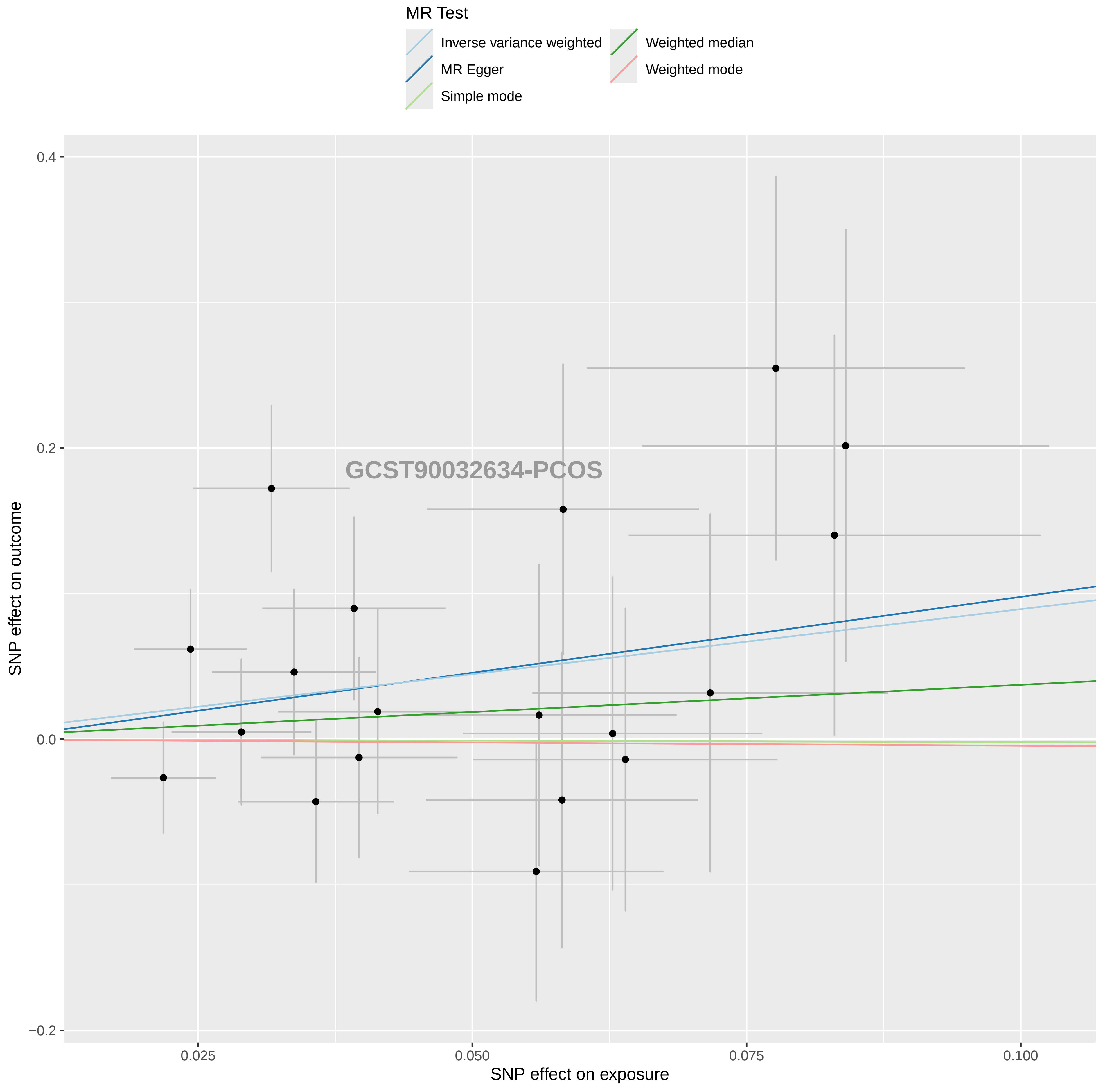
MR Test
Inverse variance weighted
MR Egger
Simple mode
Weighted median
Weighted mode
GCST90032634-PCOS
SNP effect on outcome
SNP effect on exposure
0.4
0.2
0.0
−0.2
0.025
0.050
0.075
0.100

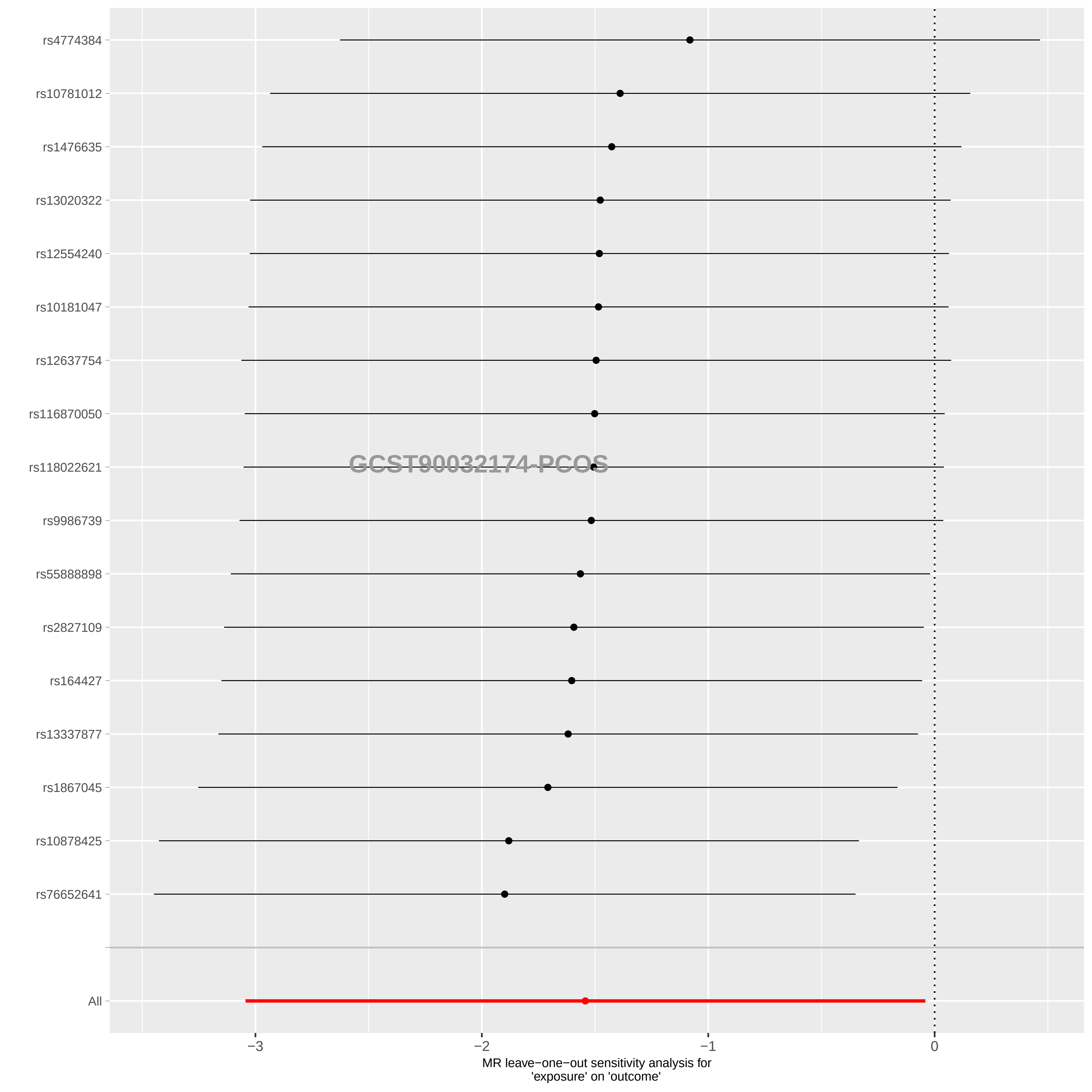
GCST90032174-PCOS
rs4774384
rs10781012
rs1476635
rs13020322
rs12554240
rs10181047
rs12637754
rs116870050
rs118022621
rs9986739
rs55888898
rs2827109
rs164427
rs13337877
rs1867045
rs10878425
rs76652641
All
−3
−2
−1
0
MR leave−one−out sensitivity analysis for
'exposure' on 'outcome'

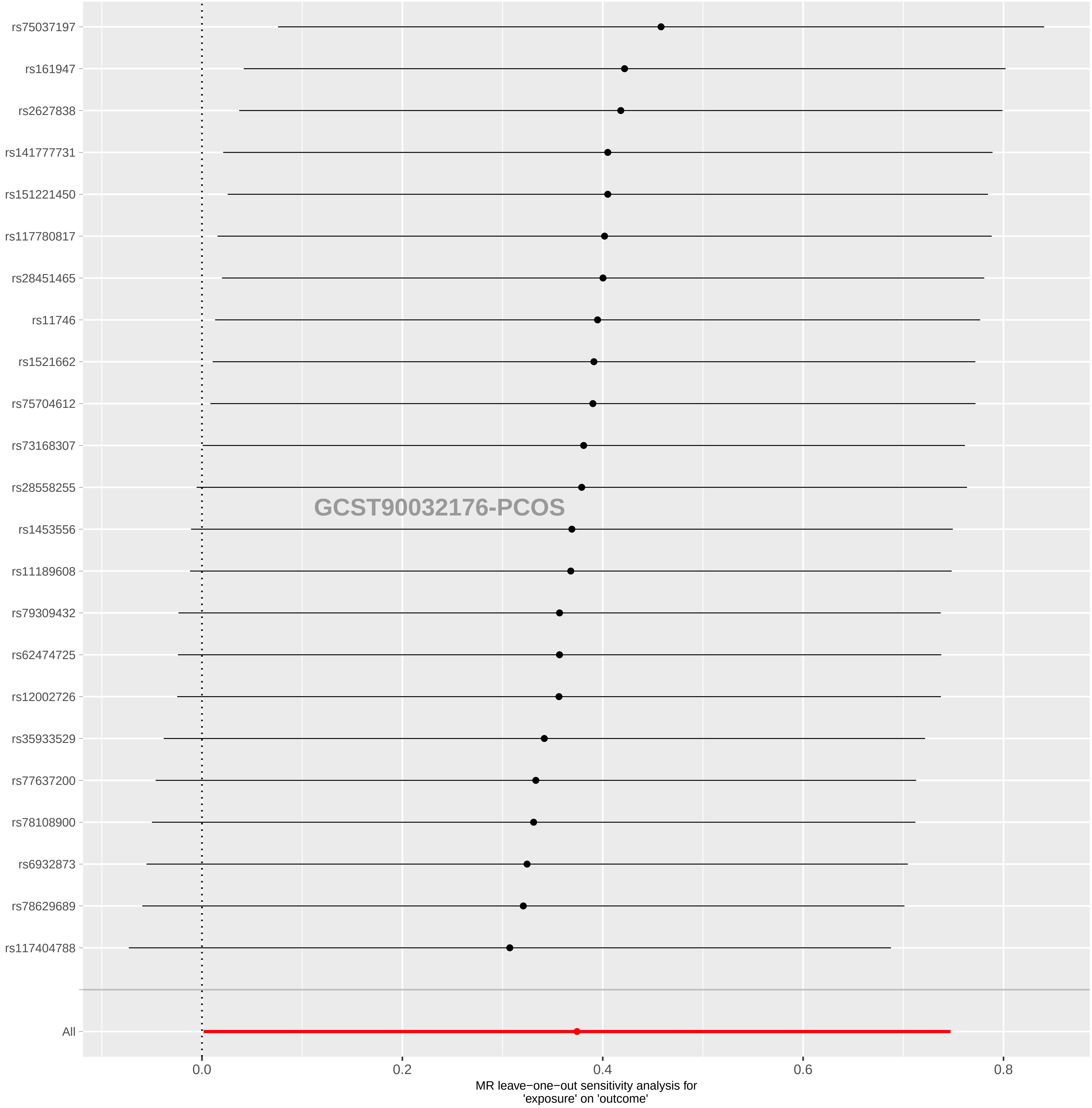
rs75037197
rs161947
rs2627838
rs141777731
rs151221450
rs117780817
rs28451465
rs11746
rs1521662
rs75704612
rs73168307
rs28558255
GCST90032176-PCOS
rs1453556
rs11189608
rs79309432
rs62474725
rs12002726
rs35933529
rs77637200
rs78108900
rs6932873
rs78629689
rs117404788
All
0.0
0.2
0.4
0.6
0.8
MR leave−one−out sensitivity analysis for
'exposure' on 'outcome'

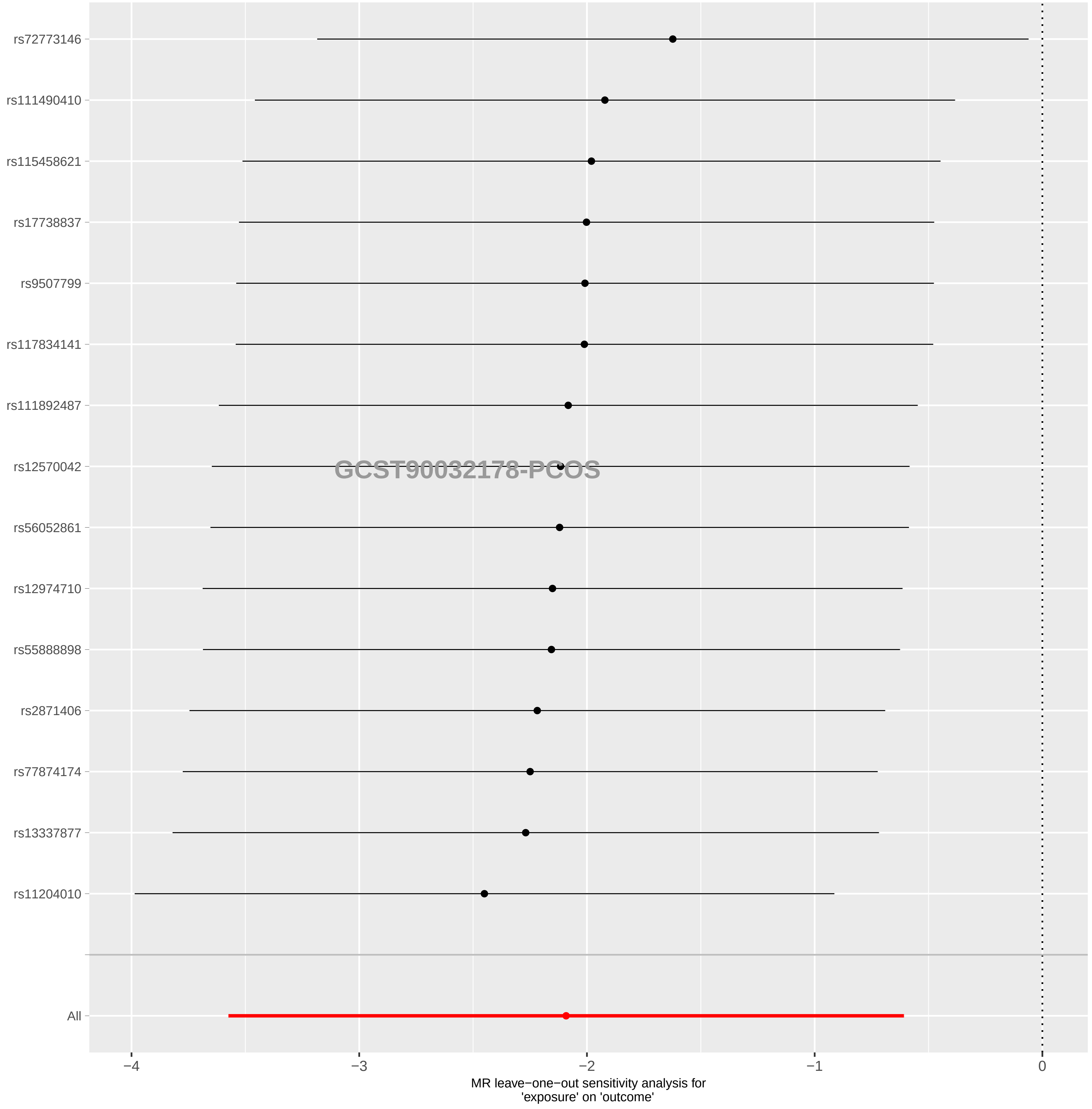

rs72773146
rs111490410
rs115458621
rs17738837
rs9507799
rs117834141
rs111892487
rs12570042
rs56052861
rs12974710
rs55888898
rs2871406
rs77874174
rs13337877
rs11204010
All
GCST90032178-PCOS
−4
−3
−2
−1
0
MR leave−one−out sensitivity analysis for
'exposure' on 'outcome'

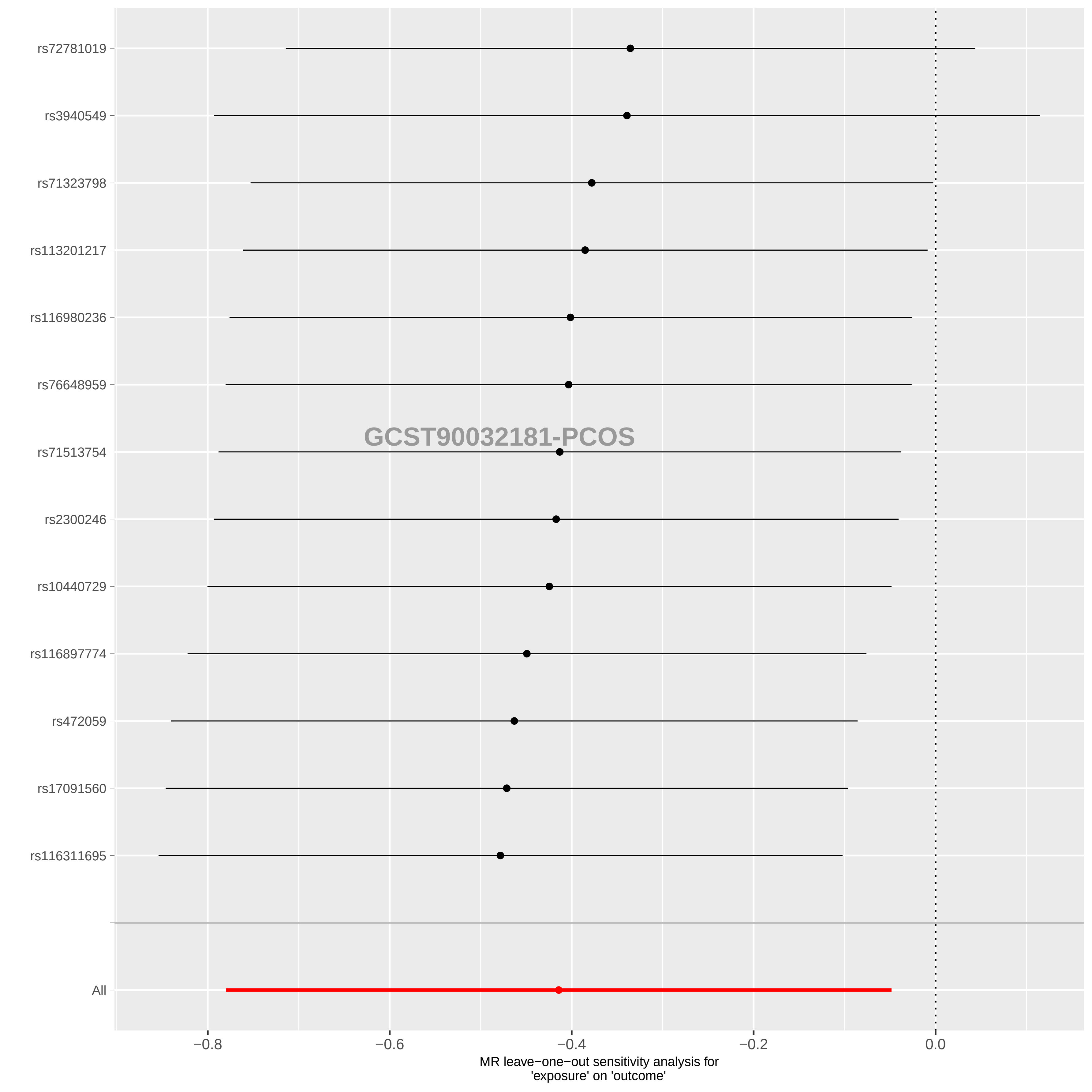
GCST90032181-PCOS
rs72781019
rs3940549
rs71323798
rs113201217
rs116980236
rs76648959
rs71513754
rs2300246
rs10440729
rs116897774
rs472059
rs17091560
rs116311695
All
−0.8
−0.6
−0.4
−0.2
0.0
MR leave−one−out sensitivity analysis for
'exposure' on 'outcome'

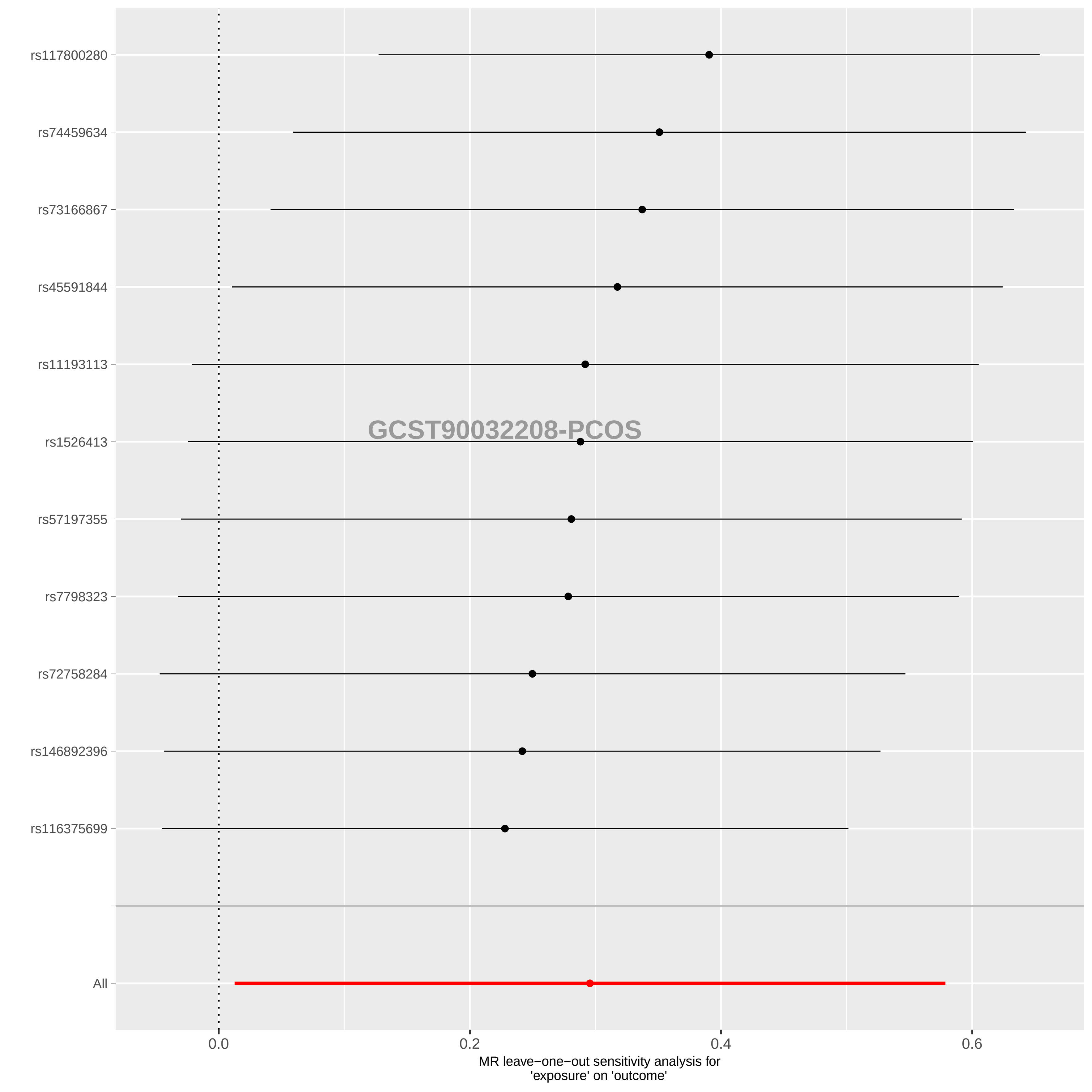

rs117800280
rs74459634
rs73166867
rs45591844
rs11193113
rs1526413
rs57197355
rs7798323
rs72758284
rs146892396
rs116375699
All
GCST90032208-PCOS
0.0
0.2
0.4
0.6
MR leave−one−out sensitivity analysis for
'exposure' on 'outcome'

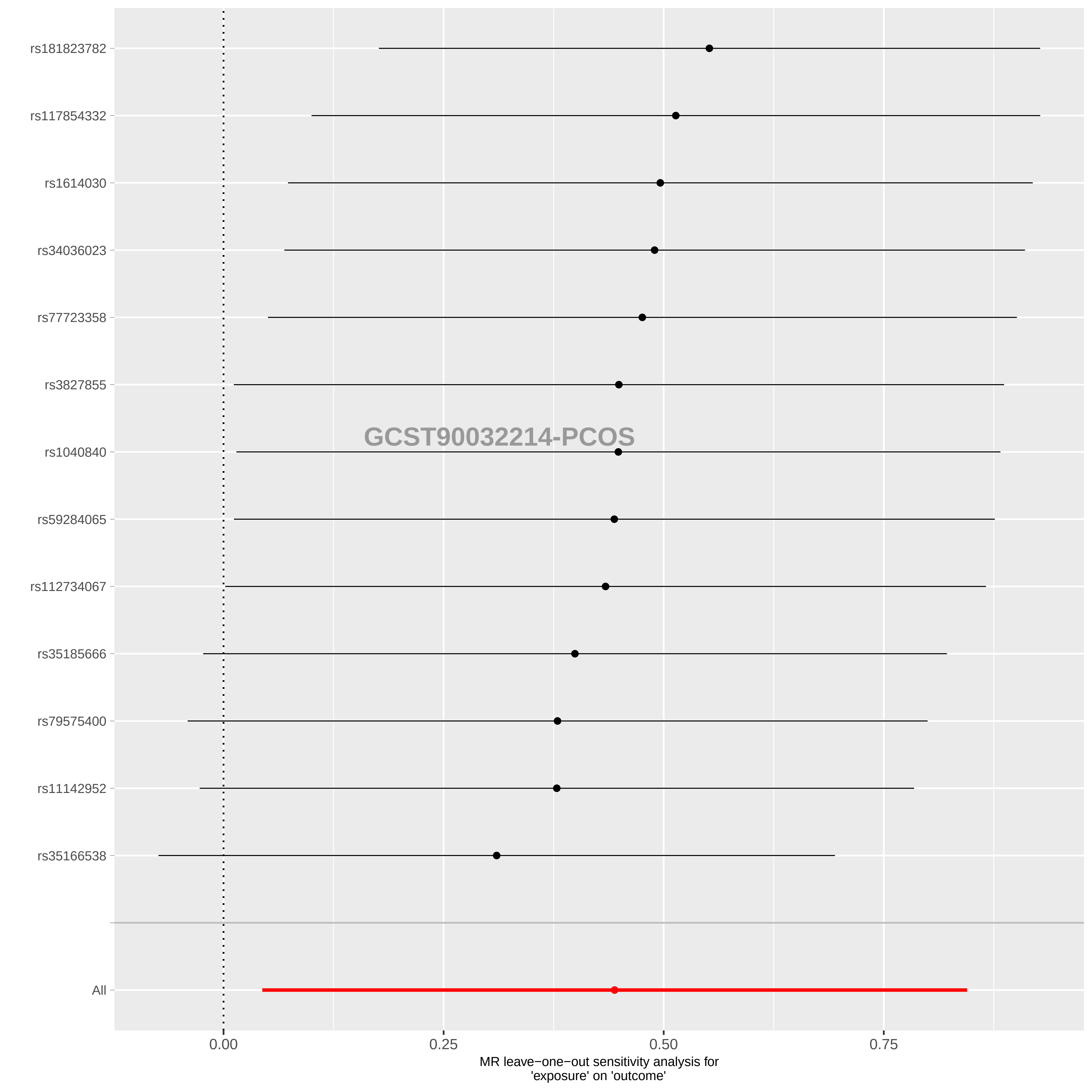

GCST90032214-PCOS
rs181823782
rs117854332
rs1614030
rs34036023
rs77723358
rs3827855
rs1040840
rs59284065
rs112734067
rs35185666
rs79575400
rs11142952
rs35166538
All
0.00
0.25
0.50
0.75
MR leave−one−out sensitivity analysis for
'exposure' on 'outcome'

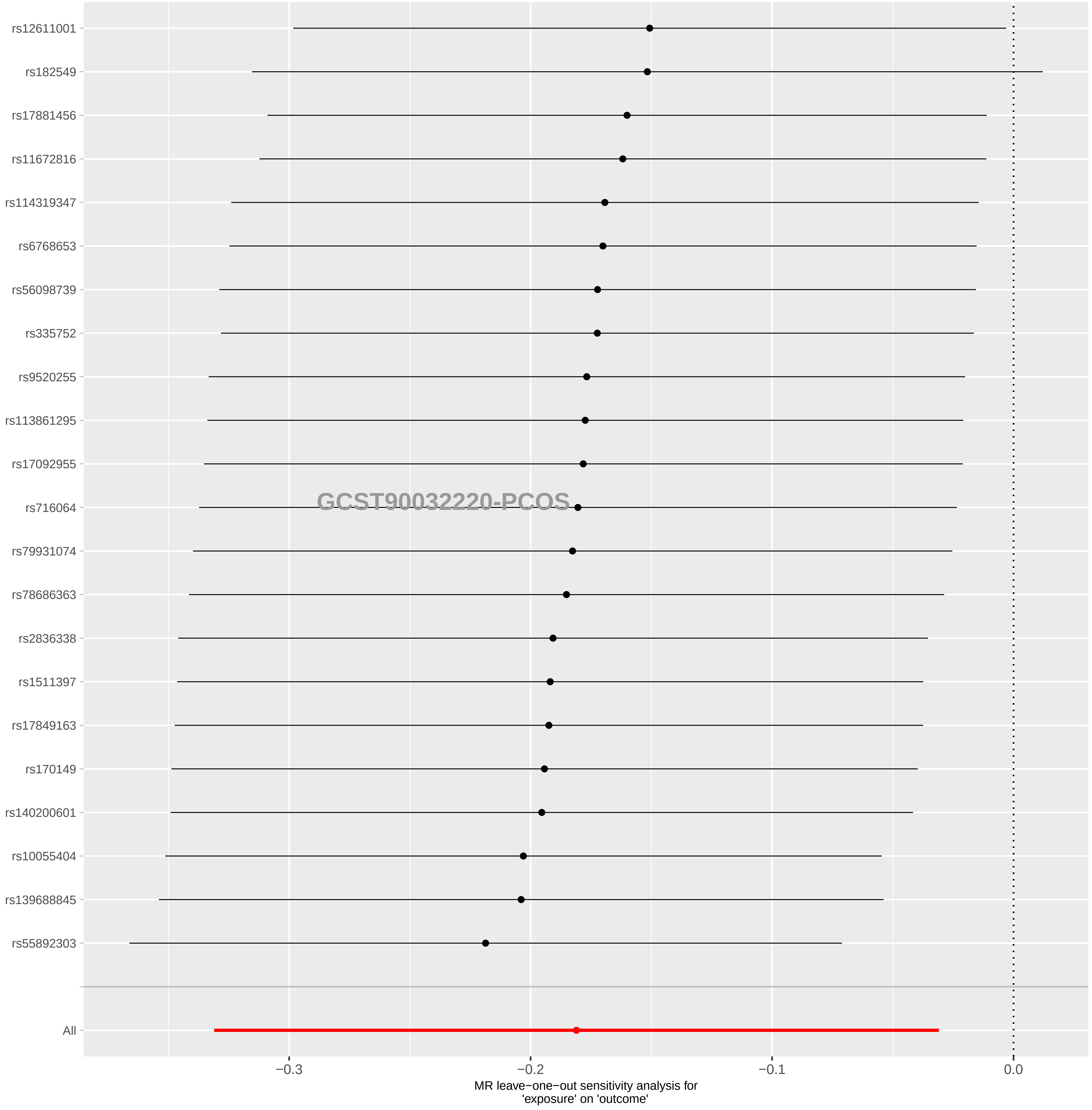

rs12611001
rs182549
rs17881456
rs11672816
rs114319347
rs6768653
rs56098739
rs335752
rs9520255
rs113861295
rs17092955
rs716064
rs79931074
rs78686363
rs2836338
rs1511397
rs17849163
rs170149
rs140200601
rs10055404
rs139688845
rs55892303
All
GCST90032220-PCOS
−0.3
−0.2
−0.1
0.0
MR leave−one−out sensitivity analysis for
'exposure' on 'outcome'

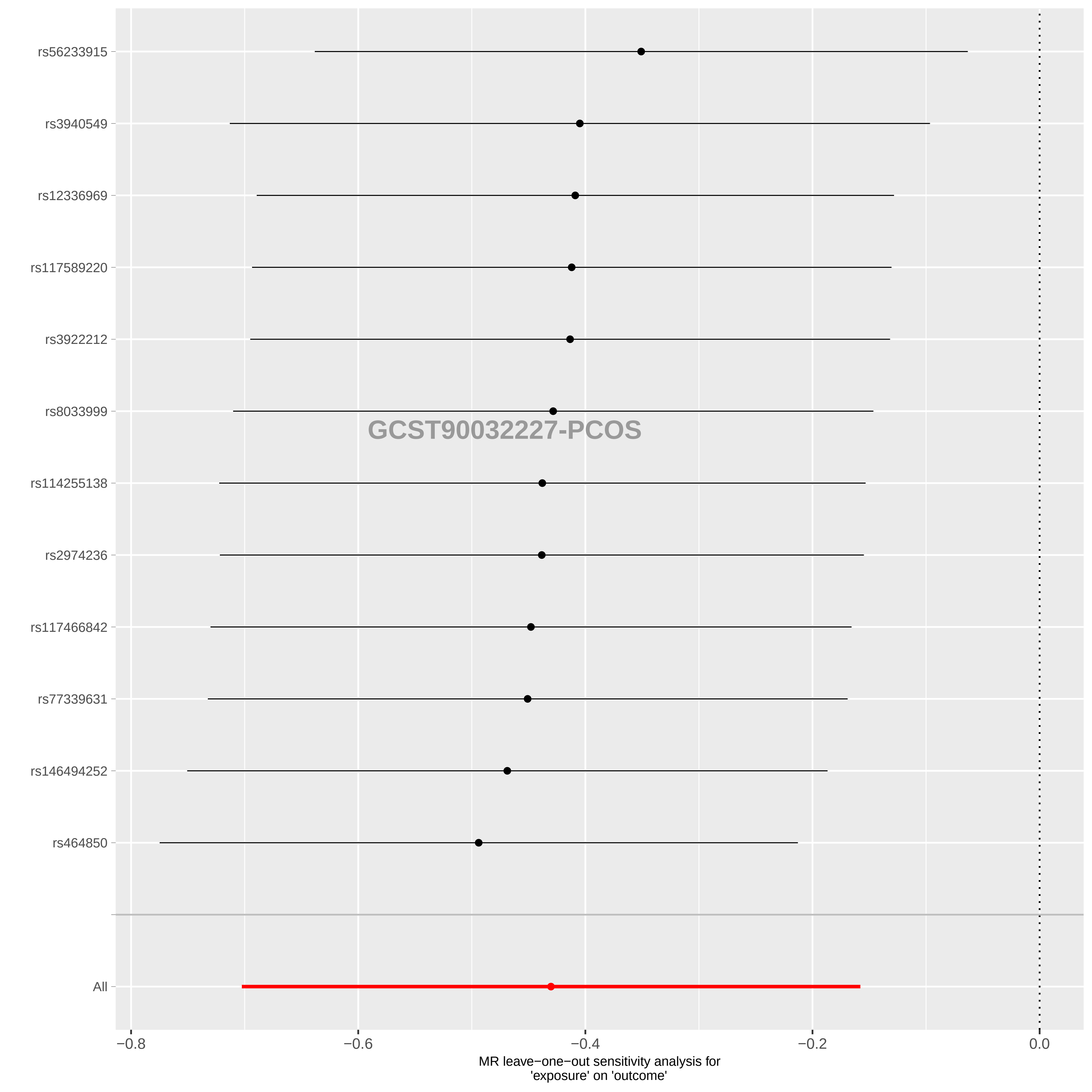
rs56233915
rs3940549
rs12336969
rs117589220
rs3922212
rs8033999
GCST90032227-PCOS
rs114255138
rs2974236
rs117466842
rs77339631
rs146494252
rs464850
All
−0.8
−0.6
−0.4
−0.2
0.0
MR leave−one−out sensitivity analysis for
'exposure' on 'outcome'

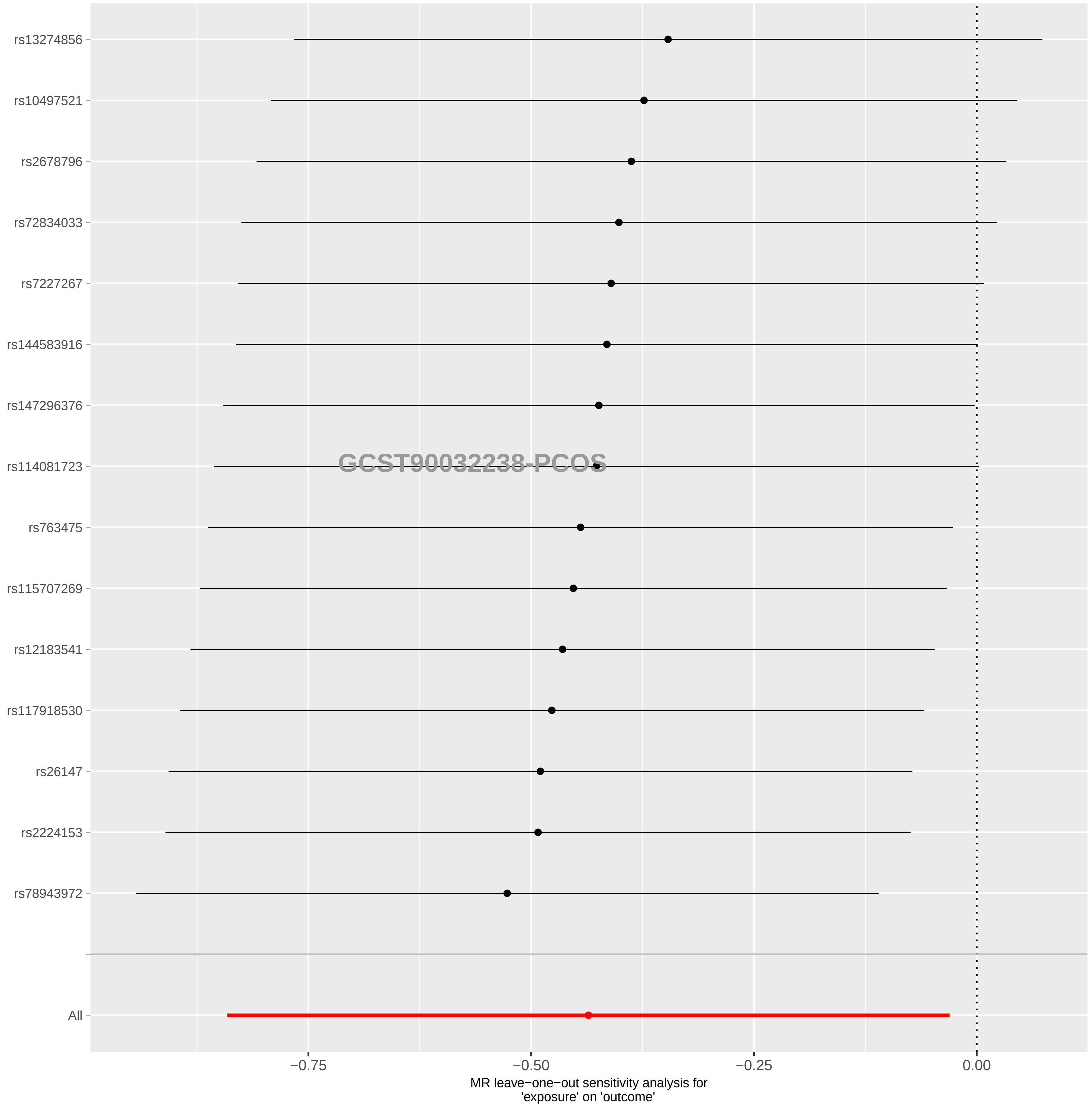
rs13274856
rs10497521
rs2678796
rs72834033
rs7227267
rs144583916
rs147296376
rs114081723
rs763475
rs115707269
rs12183541
rs117918530
rs26147
rs2224153
rs78943972
All
GCST90032238-PCOS
−0.75
−0.50
−0.25
0.00
MR leave−one−out sensitivity analysis for
'exposure' on 'outcome'

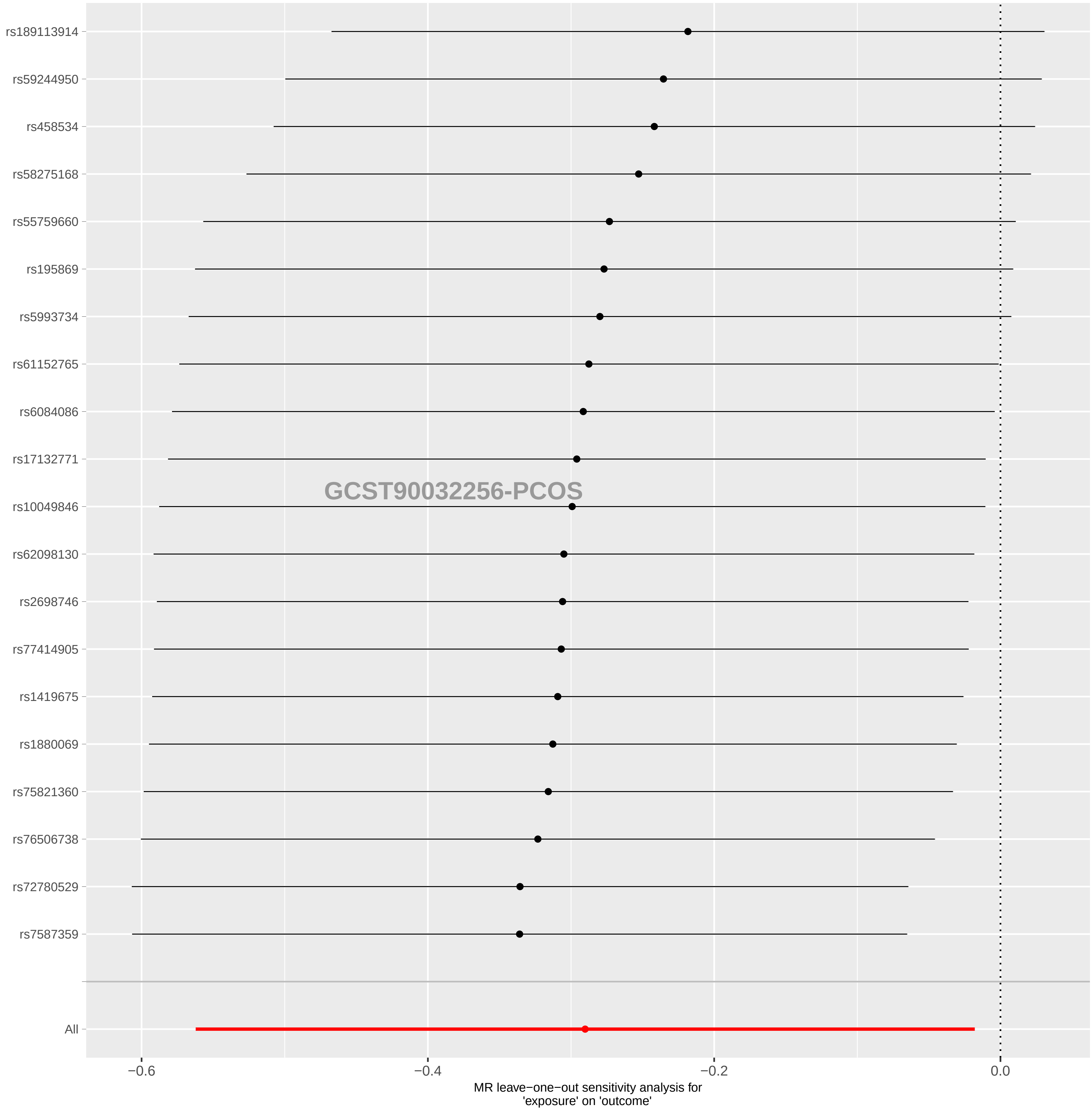
rs189113914
rs59244950
rs458534
rs58275168
rs55759660
rs195869
rs5993734
rs61152765
rs6084086
rs17132771
GCST90032256-PCOS
rs10049846
rs62098130
rs2698746
rs77414905
rs1419675
rs1880069
rs75821360
rs76506738
rs72780529
rs7587359
All
−0.6
−0.4
−0.2
0.0
MR leave−one−out sensitivity analysis for
'exposure' on 'outcome'

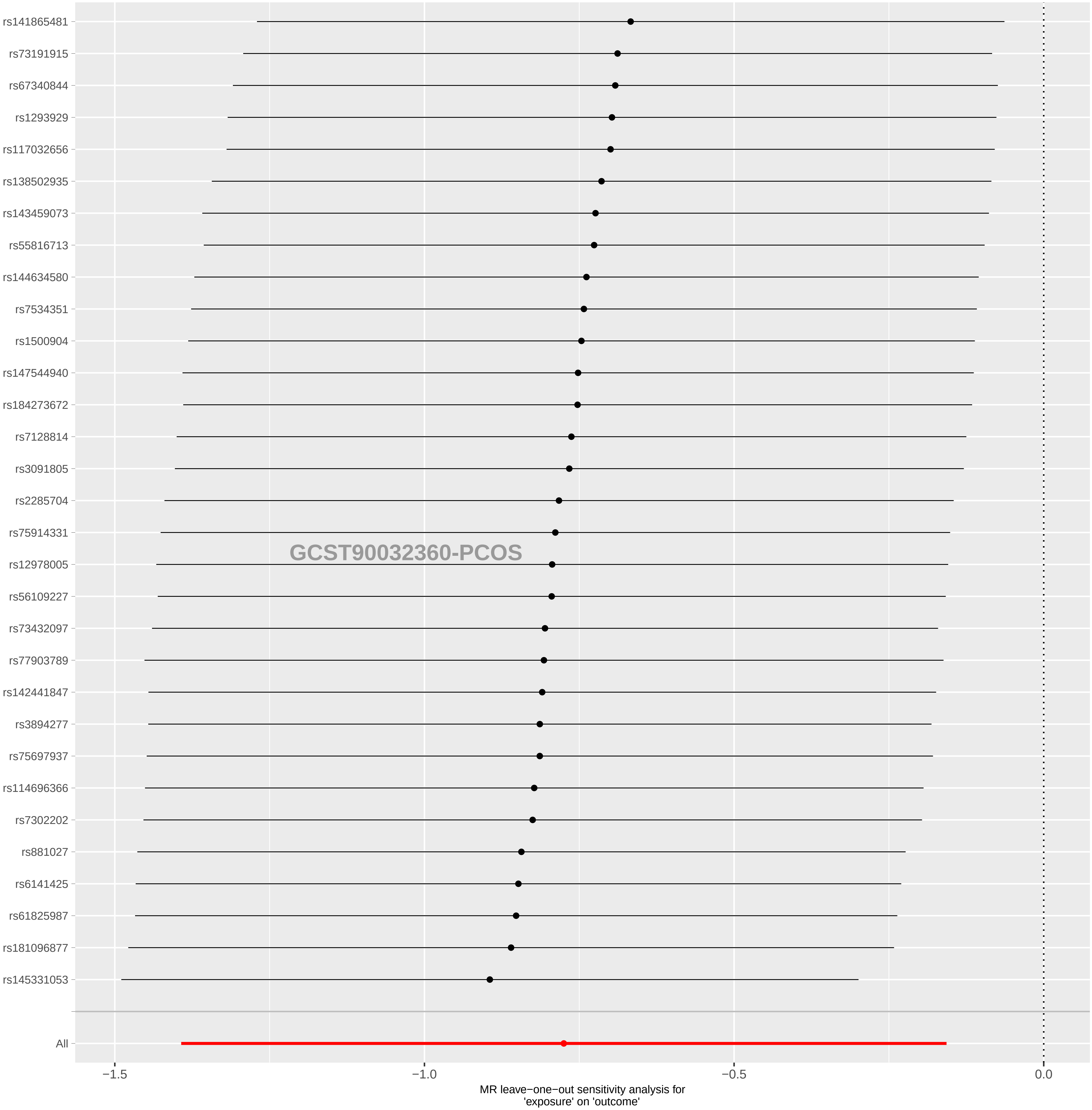
rs141865481
rs73191915
rs67340844
rs1293929
rs117032656
rs138502935
rs143459073
rs55816713
rs144634580
rs7534351
rs1500904
rs147544940
rs184273672
rs7128814
rs3091805
rs2285704
rs75914331
GCST90032360-PCOS
rs12978005
rs56109227
rs73432097
rs77903789
rs142441847
rs3894277
rs75697937
rs114696366
rs7302202
rs881027
rs6141425
rs61825987
rs181096877
rs145331053
All
−1.5
−1.0
−0.5
0.0
MR leave−one−out sensitivity analysis for
'exposure' on 'outcome'

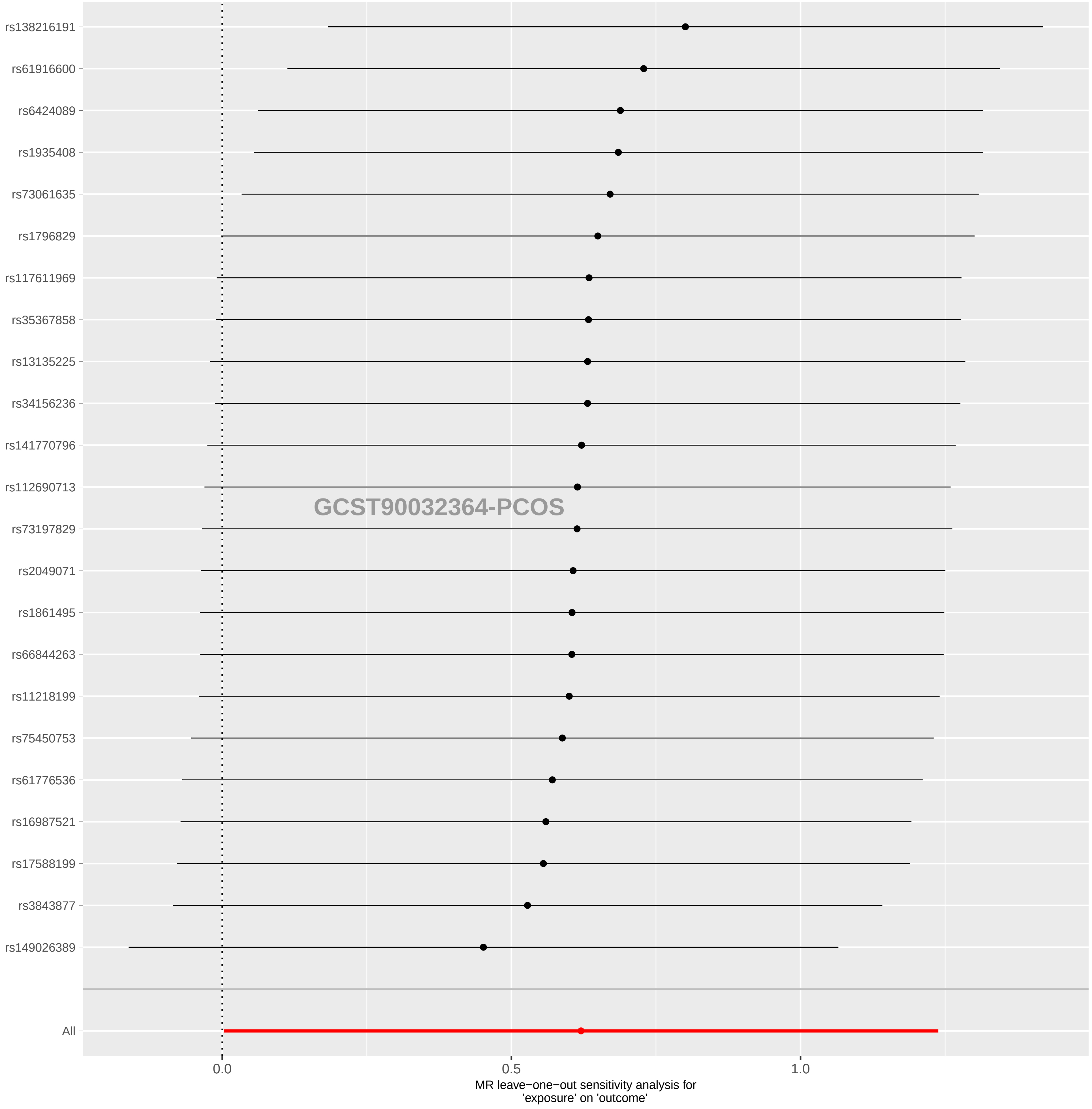

rs138216191
rs61916600
rs6424089
rs1935408
rs73061635
rs1796829
rs117611969
rs35367858
rs13135225
rs34156236
rs141770796
rs112690713
GCST90032364-PCOS
rs73197829
rs2049071
rs1861495
rs66844263
rs11218199
rs75450753
rs61776536
rs16987521
rs17588199
rs3843877
rs149026389
All
0.0
0.5
1.0
MR leave−one−out sensitivity analysis for
'exposure' on 'outcome'

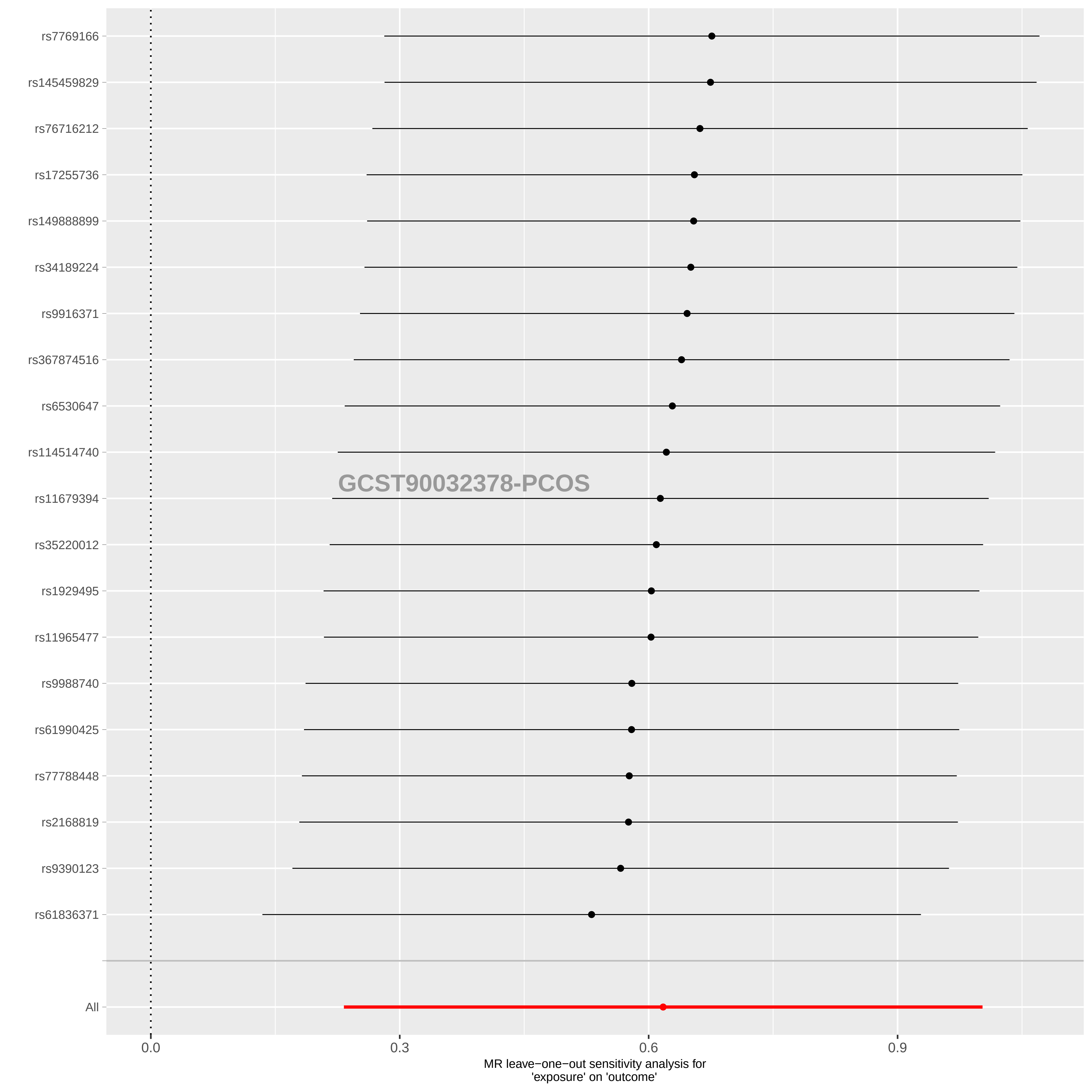
rs7769166
rs145459829
rs76716212
rs17255736
rs149888899
rs34189224
rs9916371
rs367874516
rs6530647
rs114514740
GCST90032378-PCOS
rs11679394
rs35220012
rs1929495
rs11965477
rs9988740
rs61990425
rs77788448
rs2168819
rs9390123
rs61836371
All
0.0
0.3
0.6
0.9
MR leave−one−out sensitivity analysis for
'exposure' on 'outcome'

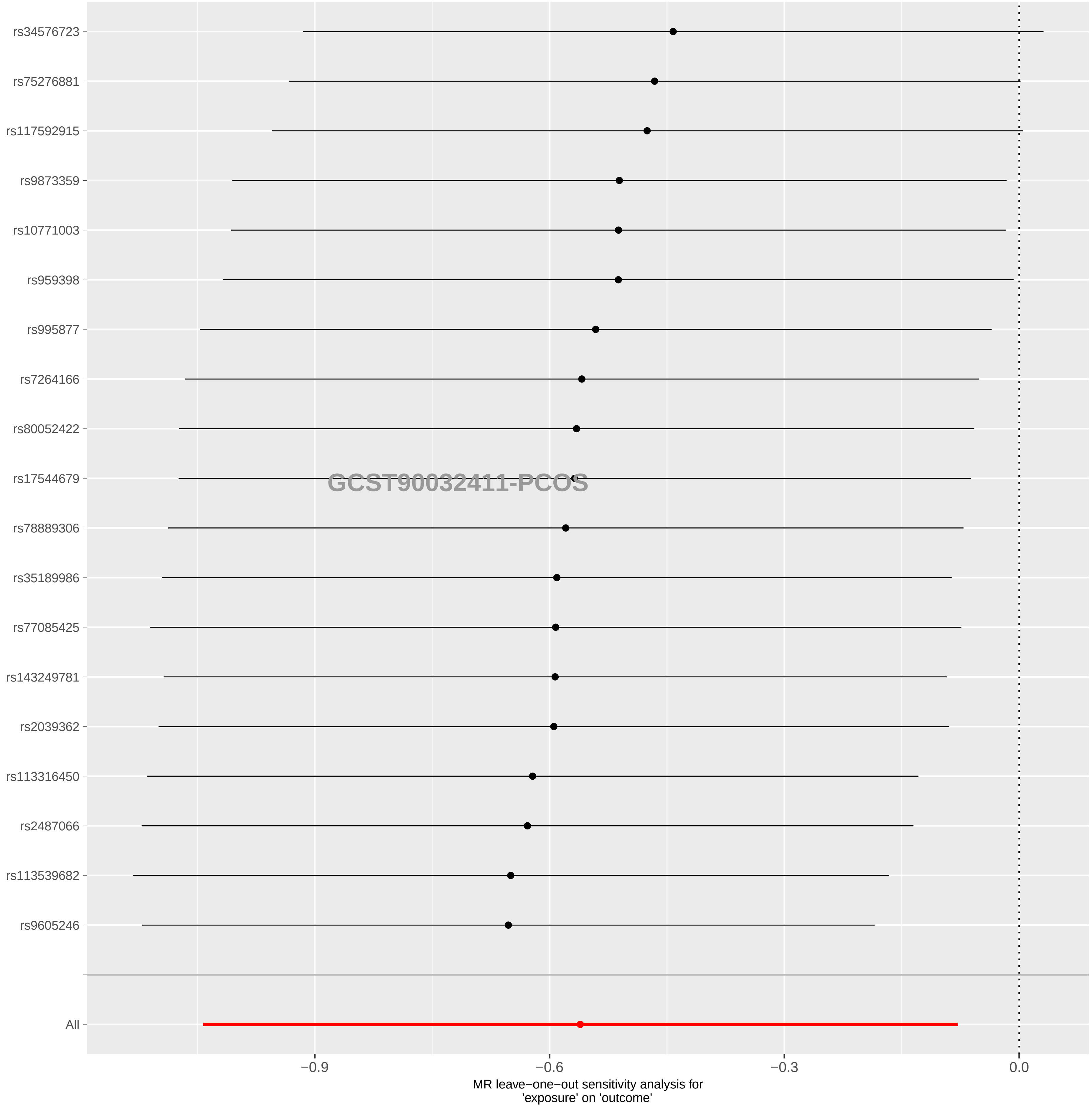
rs34576723
rs75276881
rs117592915
rs9873359
rs10771003
rs959398
rs995877
rs7264166
rs80052422
rs17544679
rs78889306
rs35189986
rs77085425
rs143249781
rs2039362
rs113316450
rs2487066
rs113539682
rs9605246
All
GCST90032411-PCOS
−0.9
−0.6
−0.3
0.0
MR leave−one−out sensitivity analysis for
'exposure' on 'outcome'

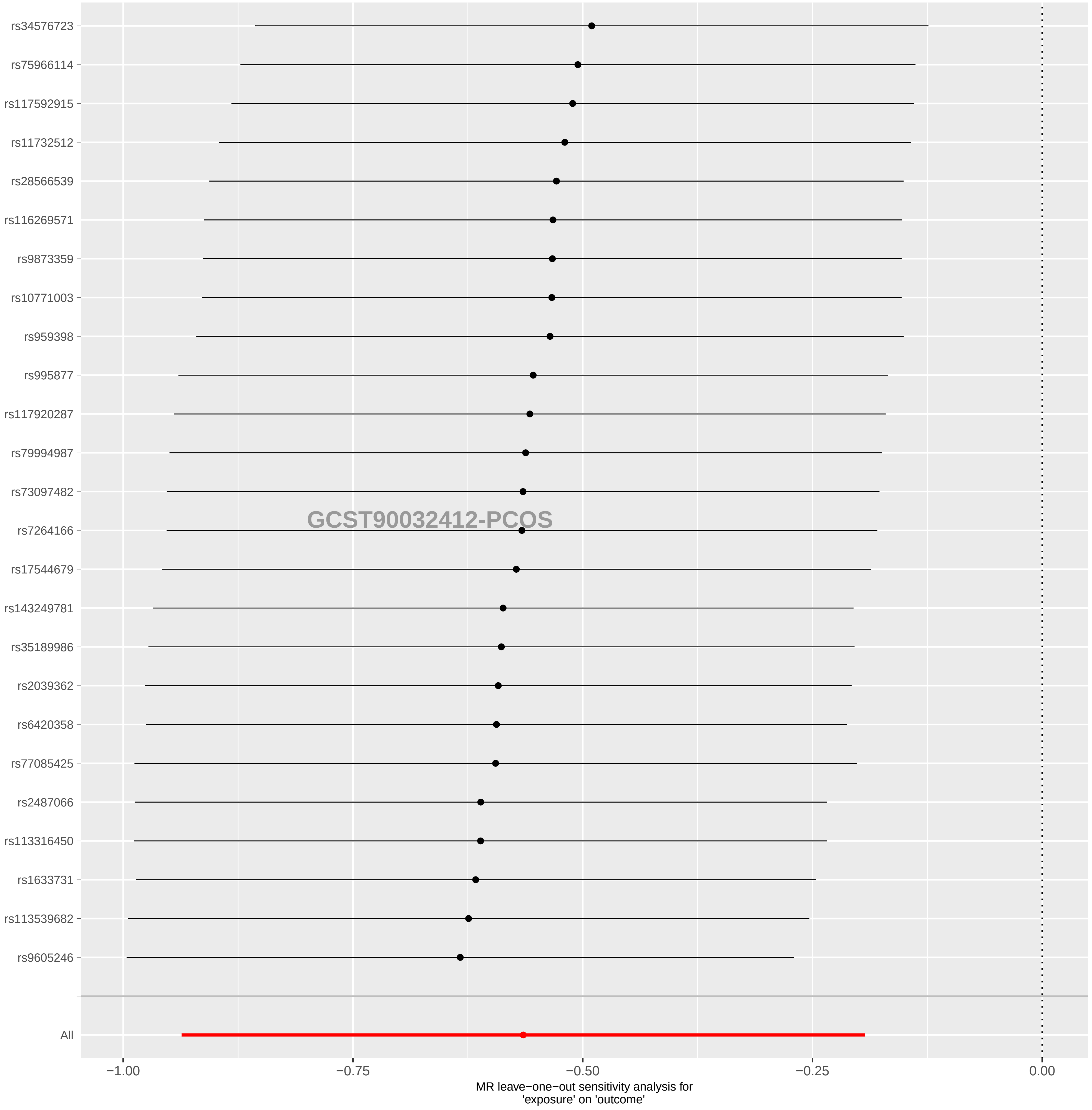

rs34576723
rs75966114
rs117592915
rs11732512
rs28566539
rs116269571
rs9873359
rs10771003
rs959398
rs995877
rs117920287
rs79994987
rs73097482
rs7264166
rs17544679
rs143249781
rs35189986
rs2039362
rs6420358
rs77085425
rs2487066
rs113316450
rs1633731
rs113539682
rs9605246
All
GCST90032412-PCOS
−1.00
−0.75
−0.50
−0.25
0.00
MR leave−one−out sensitivity analysis for
'exposure' on 'outcome'

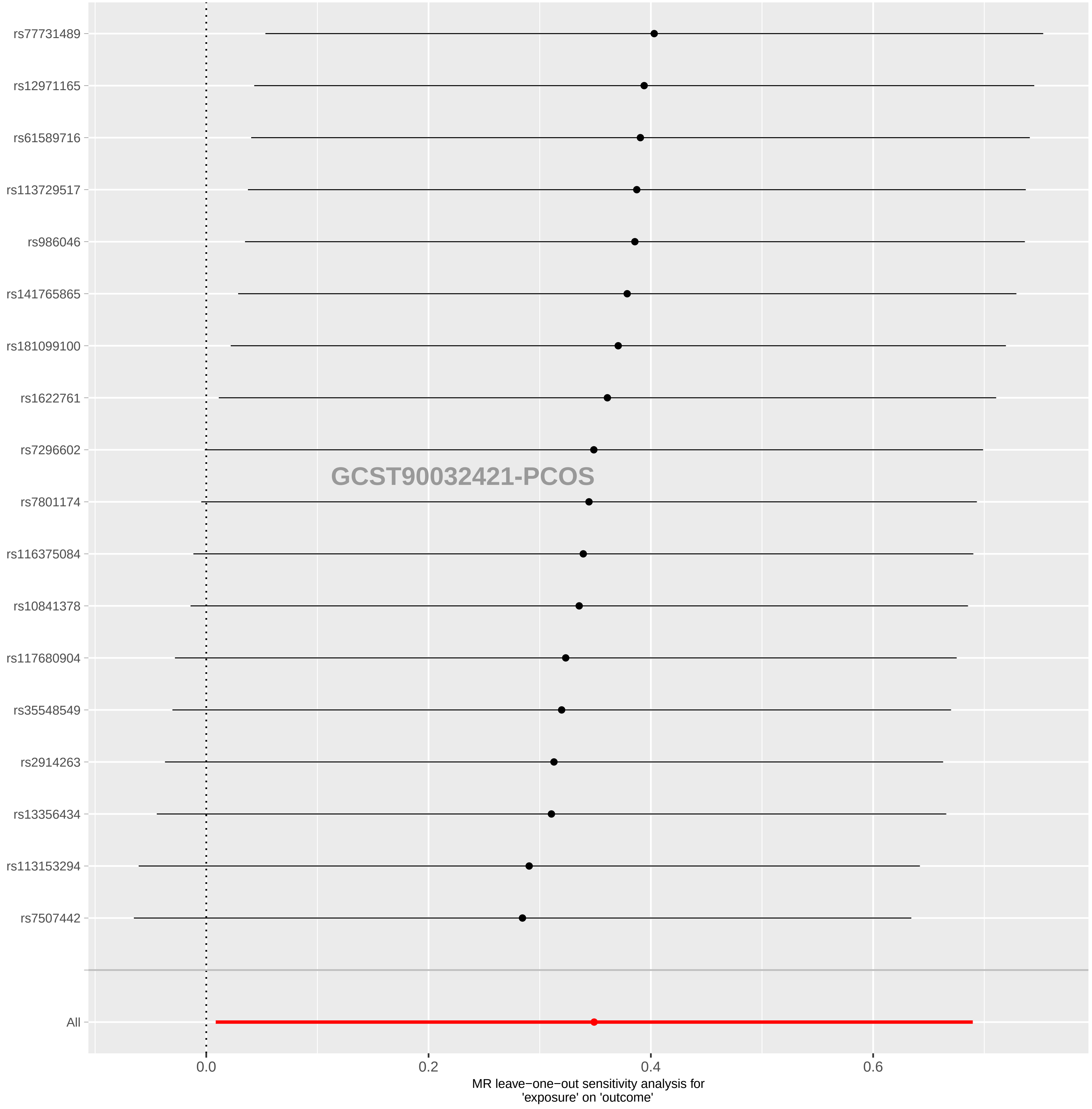
rs77731489
rs12971165
rs61589716
rs113729517
rs986046
rs141765865
rs181099100
rs1622761
rs7296602
GCST90032421-PCOS
rs7801174
rs116375084
rs10841378
rs117680904
rs35548549
rs2914263
rs13356434
rs113153294
rs7507442
All
0.0
0.2
0.4
0.6
MR leave−one−out sensitivity analysis for
'exposure' on 'outcome'

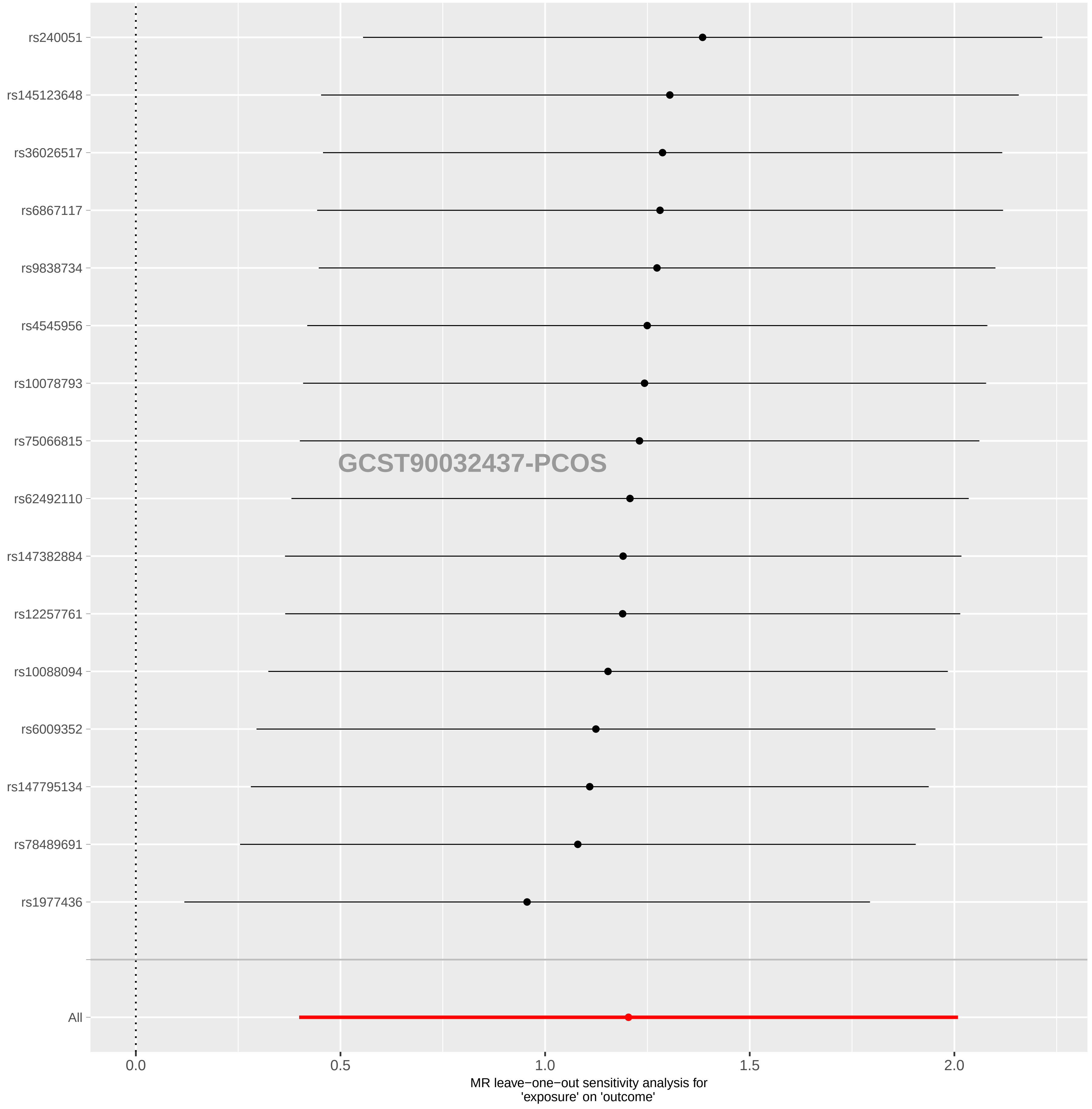
rs240051
rs145123648
rs36026517
rs6867117
rs9838734
rs4545956
rs10078793
rs75066815
GCST90032437-PCOS
rs62492110
rs147382884
rs12257761
rs10088094
rs6009352
rs147795134
rs78489691
rs1977436
All
0.0
0.5
1.0
1.5
2.0
MR leave−one−out sensitivity analysis for
'exposure' on 'outcome'

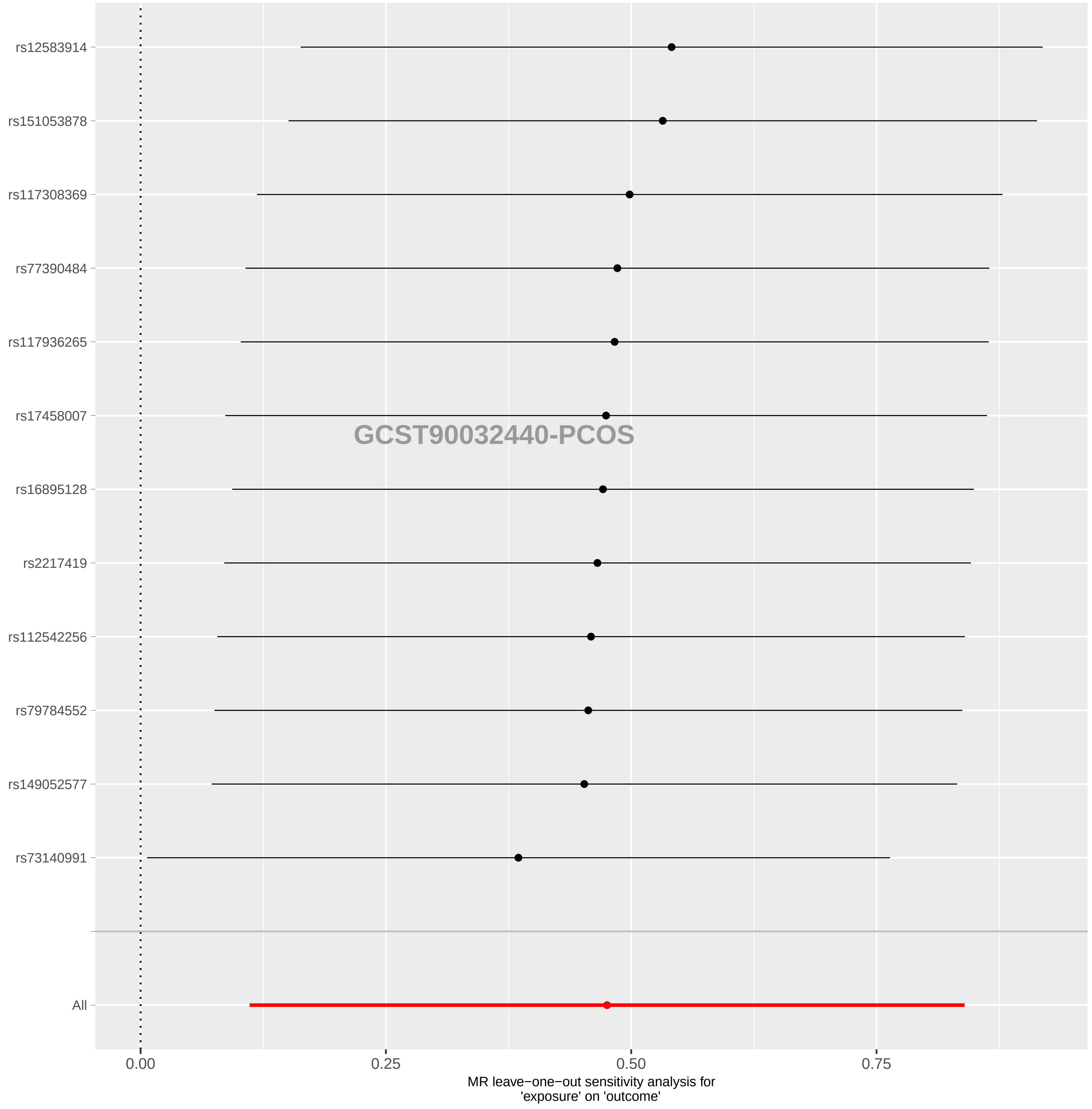

GCST90032440-PCOS
rs12583914
rs151053878
rs117308369
rs77390484
rs117936265
rs17458007
rs16895128
rs2217419
rs112542256
rs79784552
rs149052577
rs73140991
All
0.00
0.25
0.50
0.75
MR leave−one−out sensitivity analysis for
'exposure' on 'outcome'

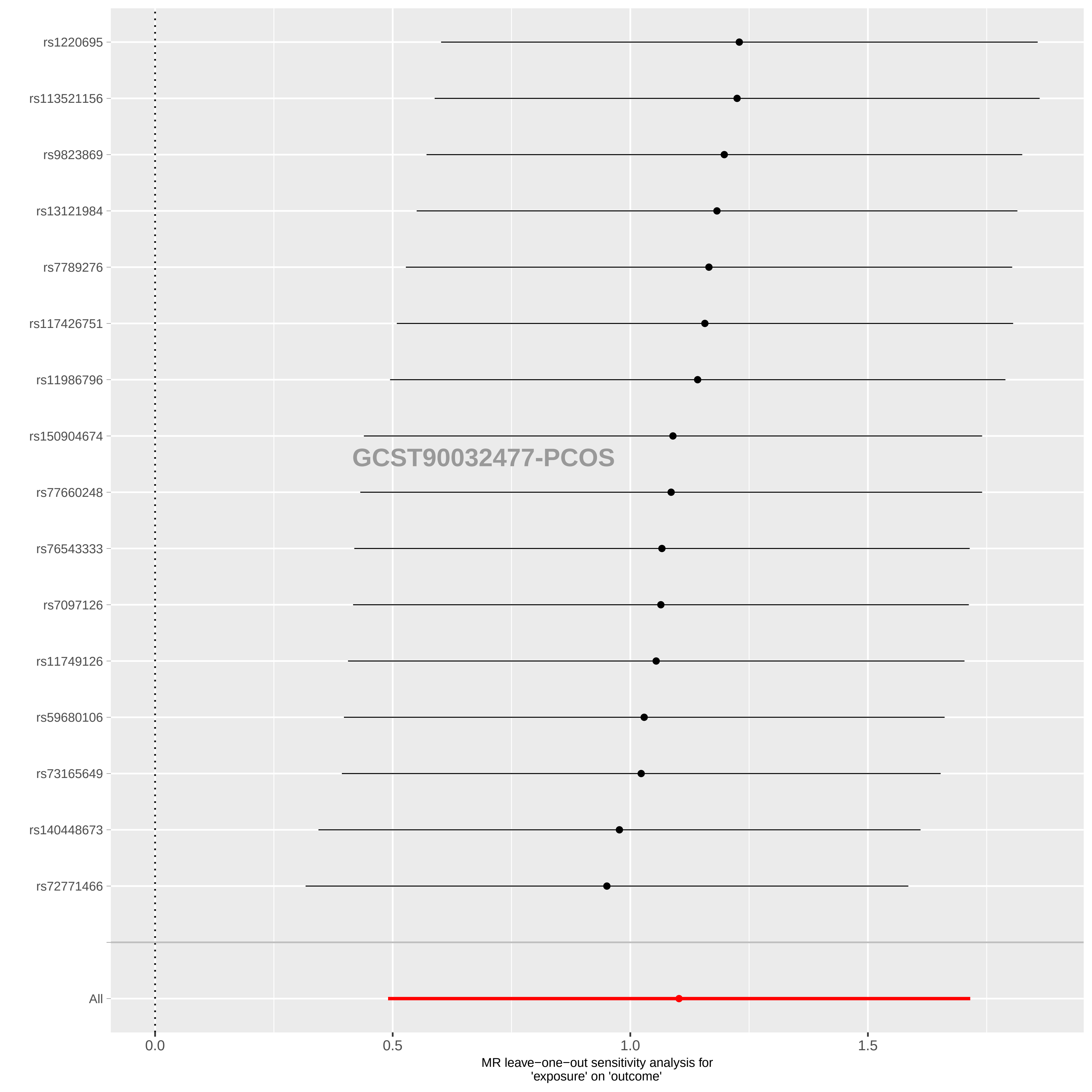
GCST90032477-PCOS
rs1220695
rs113521156
rs9823869
rs13121984
rs7789276
rs117426751
rs11986796
rs150904674
rs77660248
rs76543333
rs7097126
rs11749126
rs59680106
rs73165649
rs140448673
rs72771466
All
0.0
0.5
1.0
1.5
MR leave−one−out sensitivity analysis for
'exposure' on 'outcome'

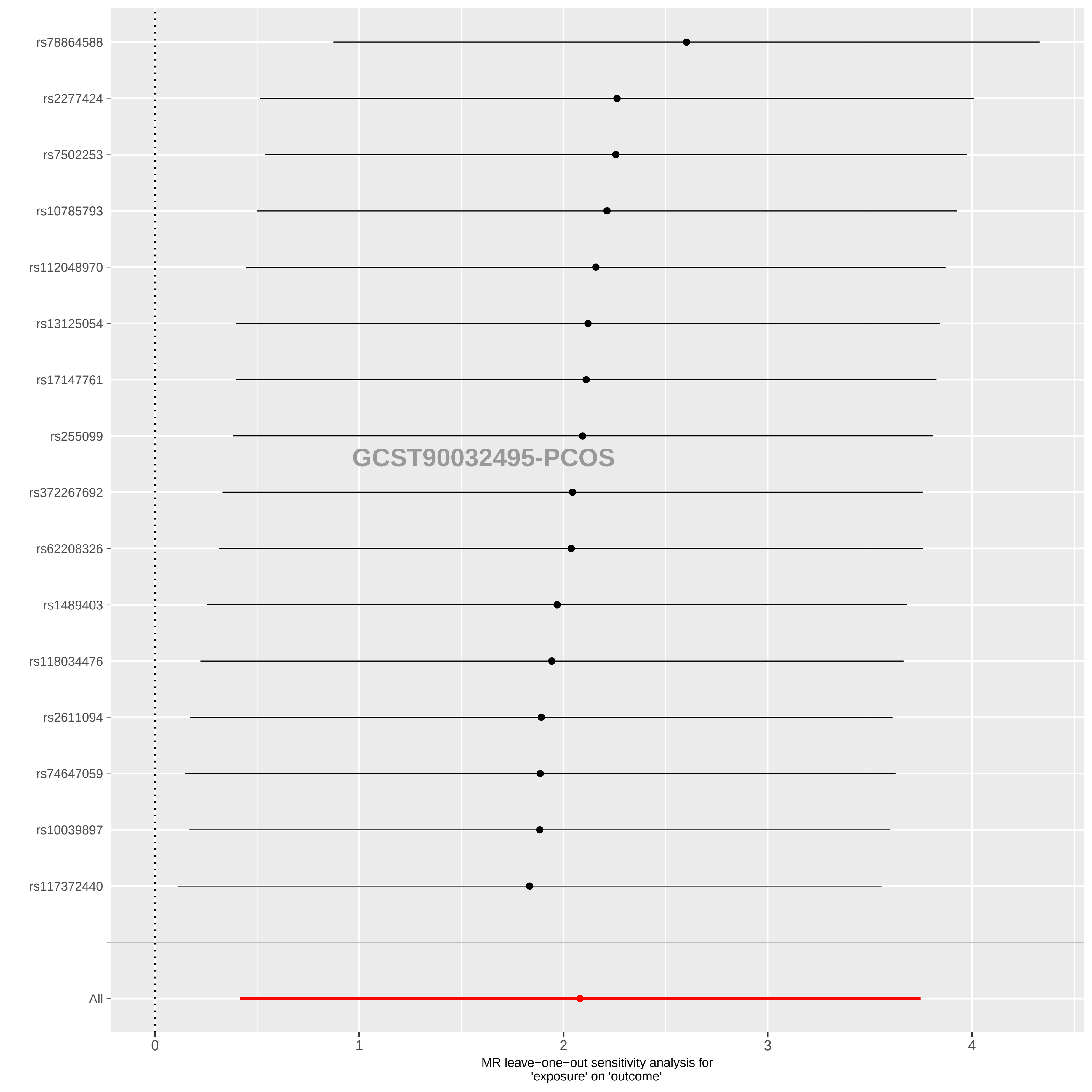
GCST90032495-PCOS
rs78864588
rs2277424
rs7502253
rs10785793
rs112048970
rs13125054
rs17147761
rs255099
rs372267692
rs62208326
rs1489403
rs118034476
rs2611094
rs74647059
rs10039897
rs117372440
All
0
1
2
3
4
MR leave−one−out sensitivity analysis for
'exposure' on 'outcome'

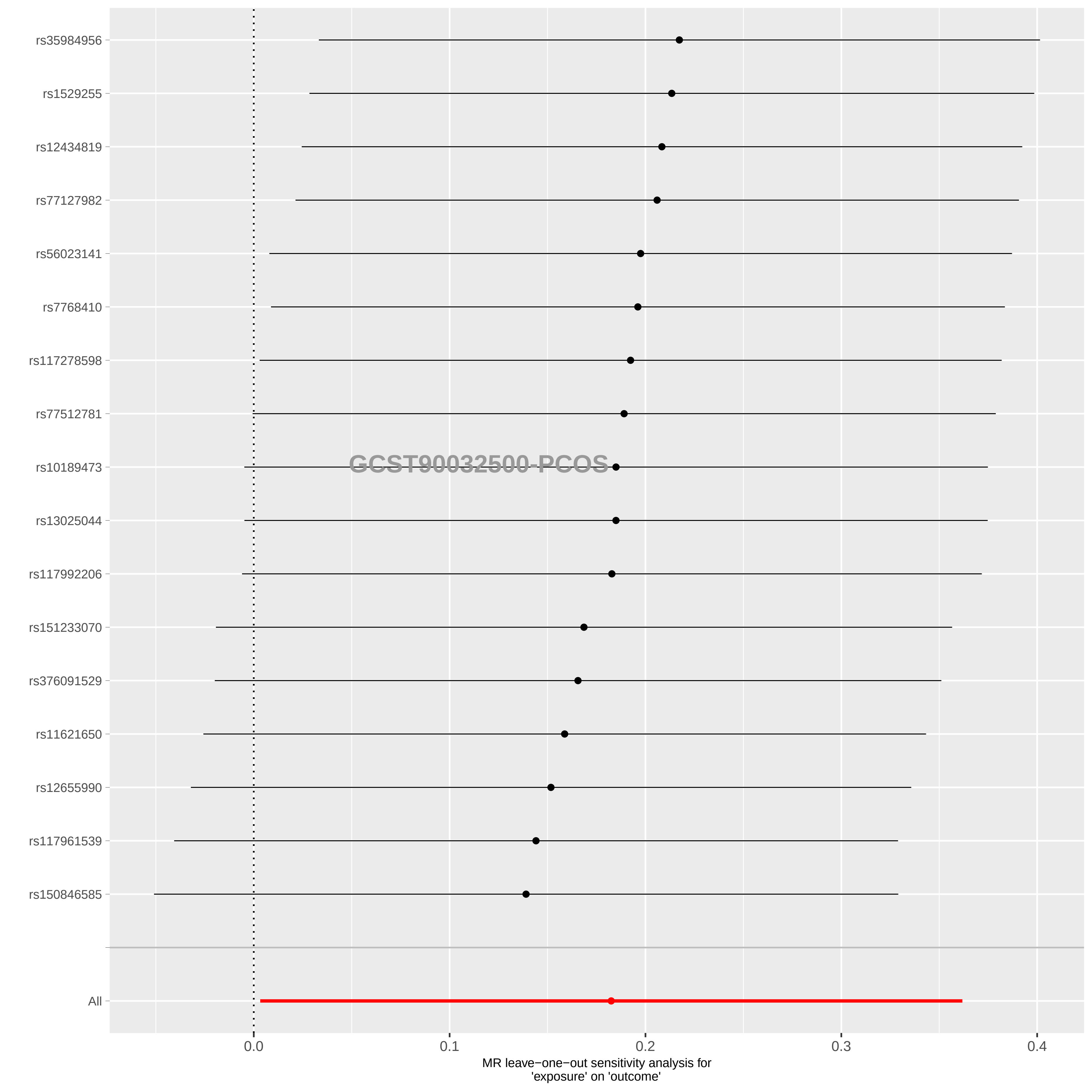
rs35984956
rs1529255
rs12434819
rs77127982
rs56023141
rs7768410
rs117278598
rs77512781
rs10189473
rs13025044
rs117992206
rs151233070
rs376091529
rs11621650
rs12655990
rs117961539
rs150846585
All
GCST90032500-PCOS
0.0
0.1
0.2
0.3
0.4
MR leave−one−out sensitivity analysis for
'exposure' on 'outcome'

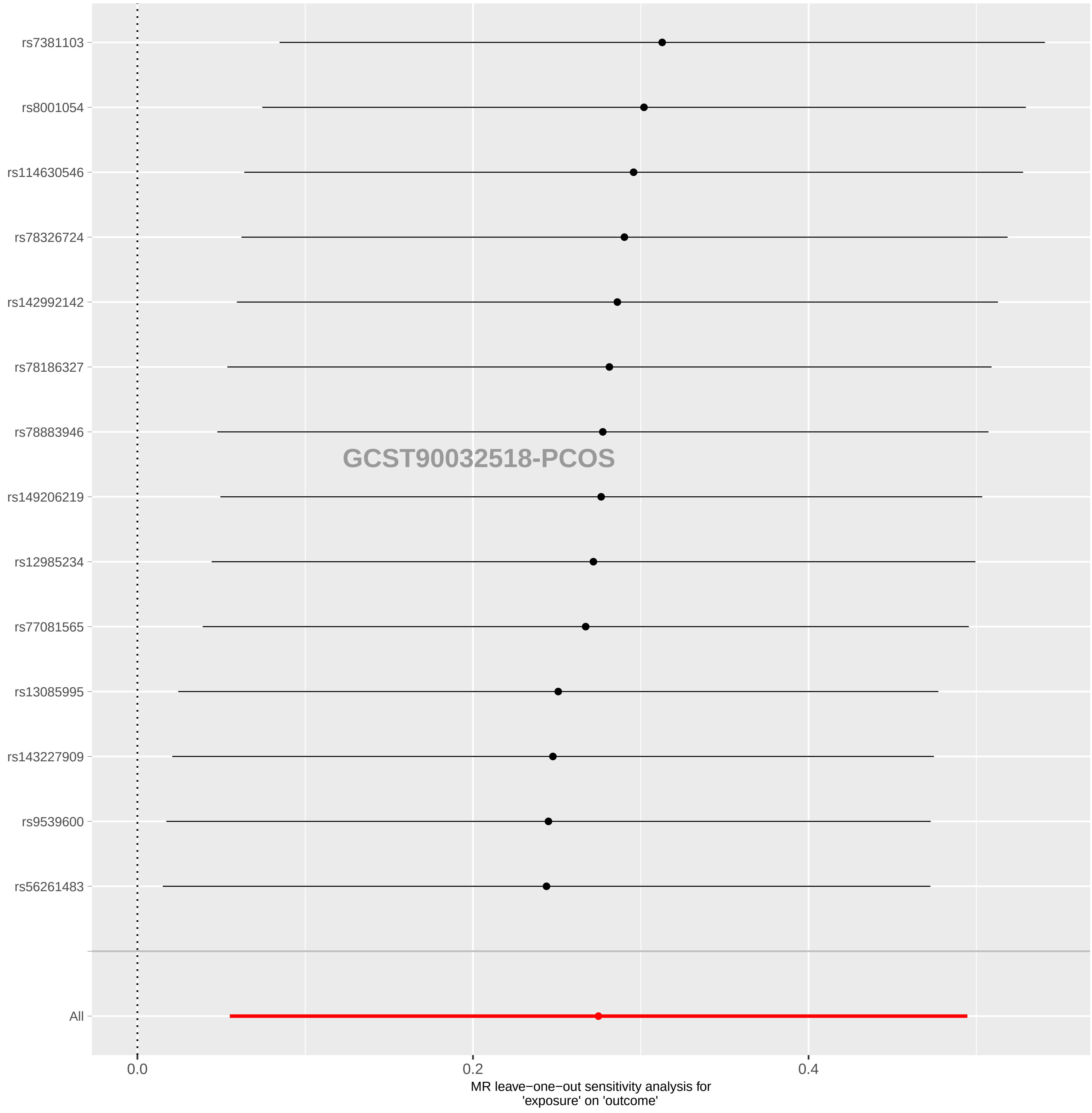

rs7381103
rs8001054
rs114630546
rs78326724
rs142992142
rs78186327
rs78883946
GCST90032518-PCOS
rs149206219
rs12985234
rs77081565
rs13085995
rs143227909
rs9539600
rs56261483
All
0.0
0.2
0.4
MR leave−one−out sensitivity analysis for
'exposure' on 'outcome'

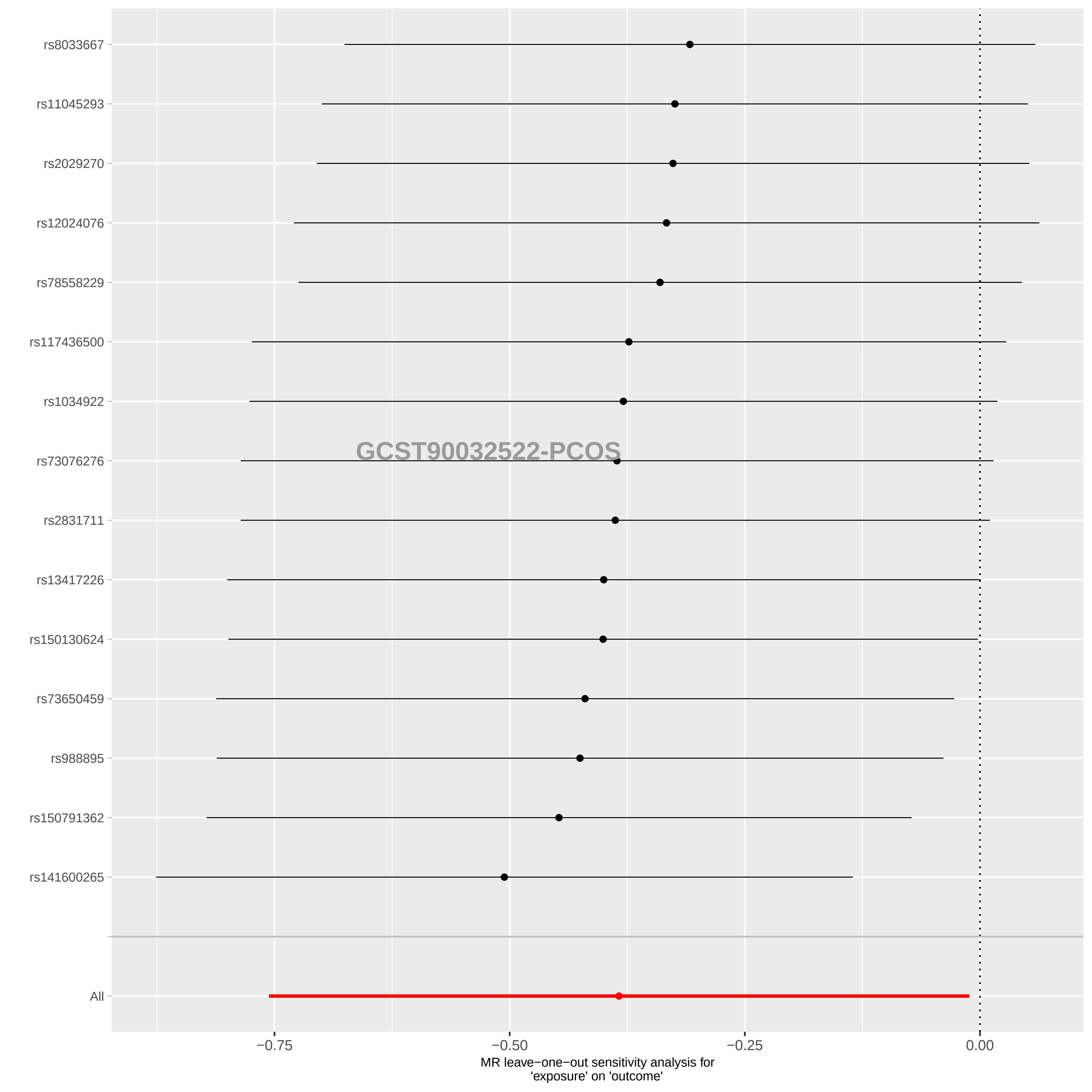

rs8033667
rs11045293
rs2029270
rs12024076
rs78558229
rs117436500
rs1034922
rs73076276
rs2831711
rs13417226
rs150130624
rs73650459
rs988895
rs150791362
rs141600265
All
GCST90032522-PCOS
−0.75
−0.50
−0.25
0.00
MR leave−one−out sensitivity analysis for
'exposure' on 'outcome'

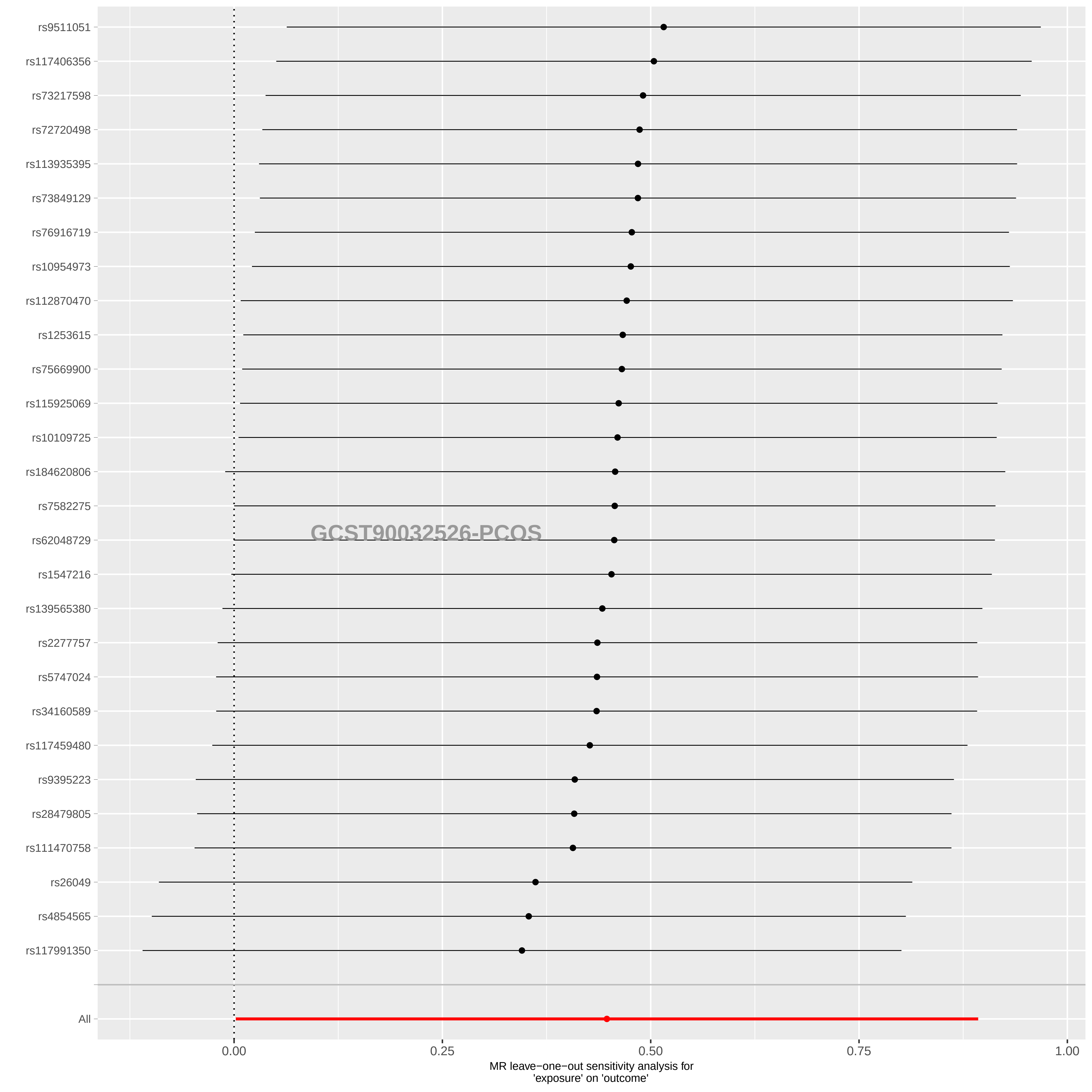

GCST90032526-PCOS
rs9511051
rs117406356
rs73217598
rs72720498
rs113935395
rs73849129
rs76916719
rs10954973
rs112870470
rs1253615
rs75669900
rs115925069
rs10109725
rs184620806
rs7582275
rs62048729
rs1547216
rs139565380
rs2277757
rs5747024
rs34160589
rs117459480
rs9395223
rs28479805
rs111470758
rs26049
rs4854565
rs117991350
All
0.00
0.25
0.50
0.75
1.00
MR leave−one−out sensitivity analysis for
'exposure' on 'outcome'

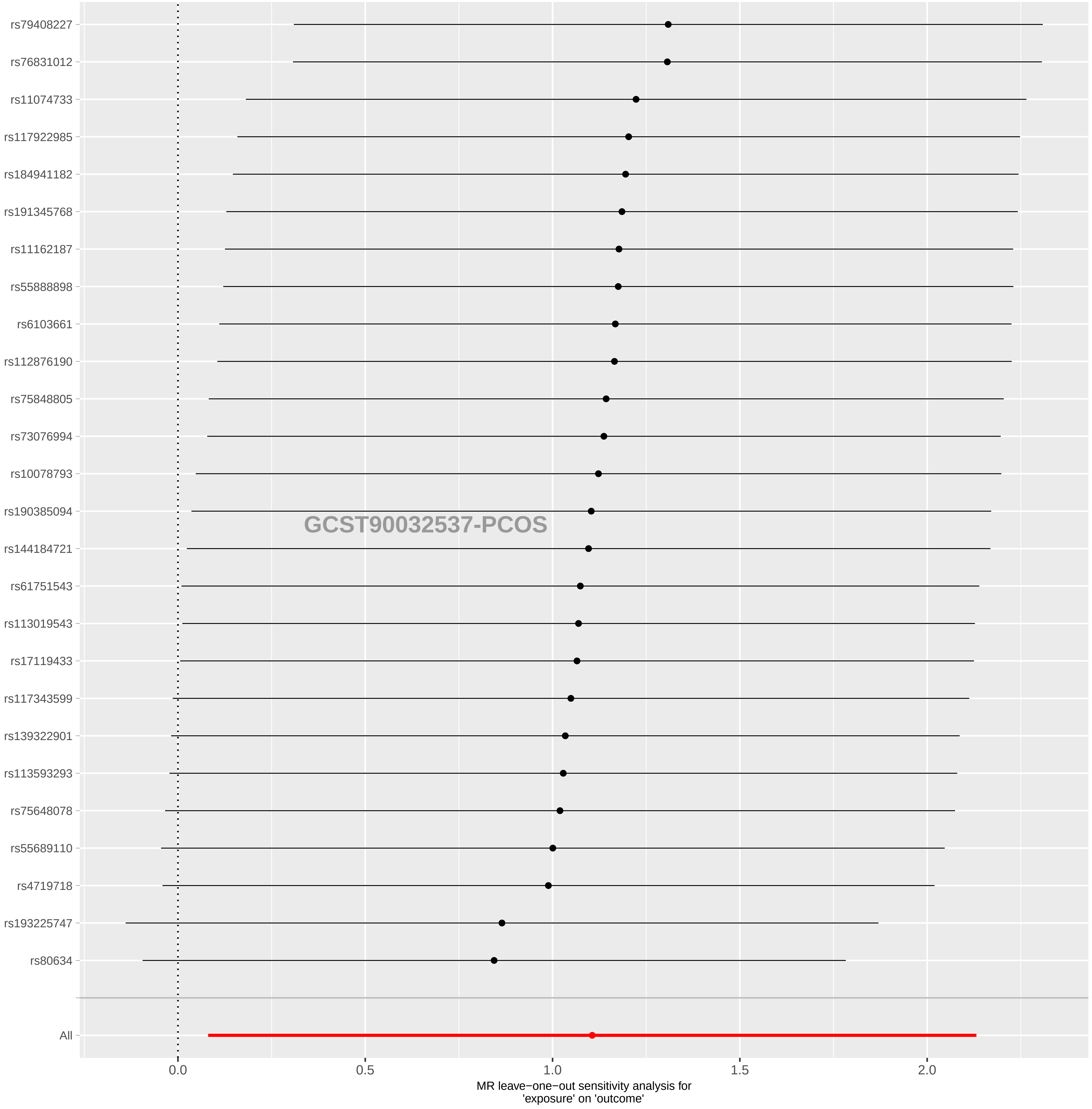

rs79408227
rs76831012
rs11074733
rs117922985
rs184941182
rs191345768
rs11162187
rs55888898
rs6103661
rs112876190
rs75848805
rs73076994
rs10078793
rs190385094
rs144184721
rs61751543
rs113019543
rs17119433
rs117343599
rs139322901
rs113593293
rs75648078
rs55689110
rs4719718
rs193225747
rs80634
All
GCST90032537-PCOS
0.0
0.5
1.0
1.5
2.0
MR leave−one−out sensitivity analysis for
'exposure' on 'outcome'

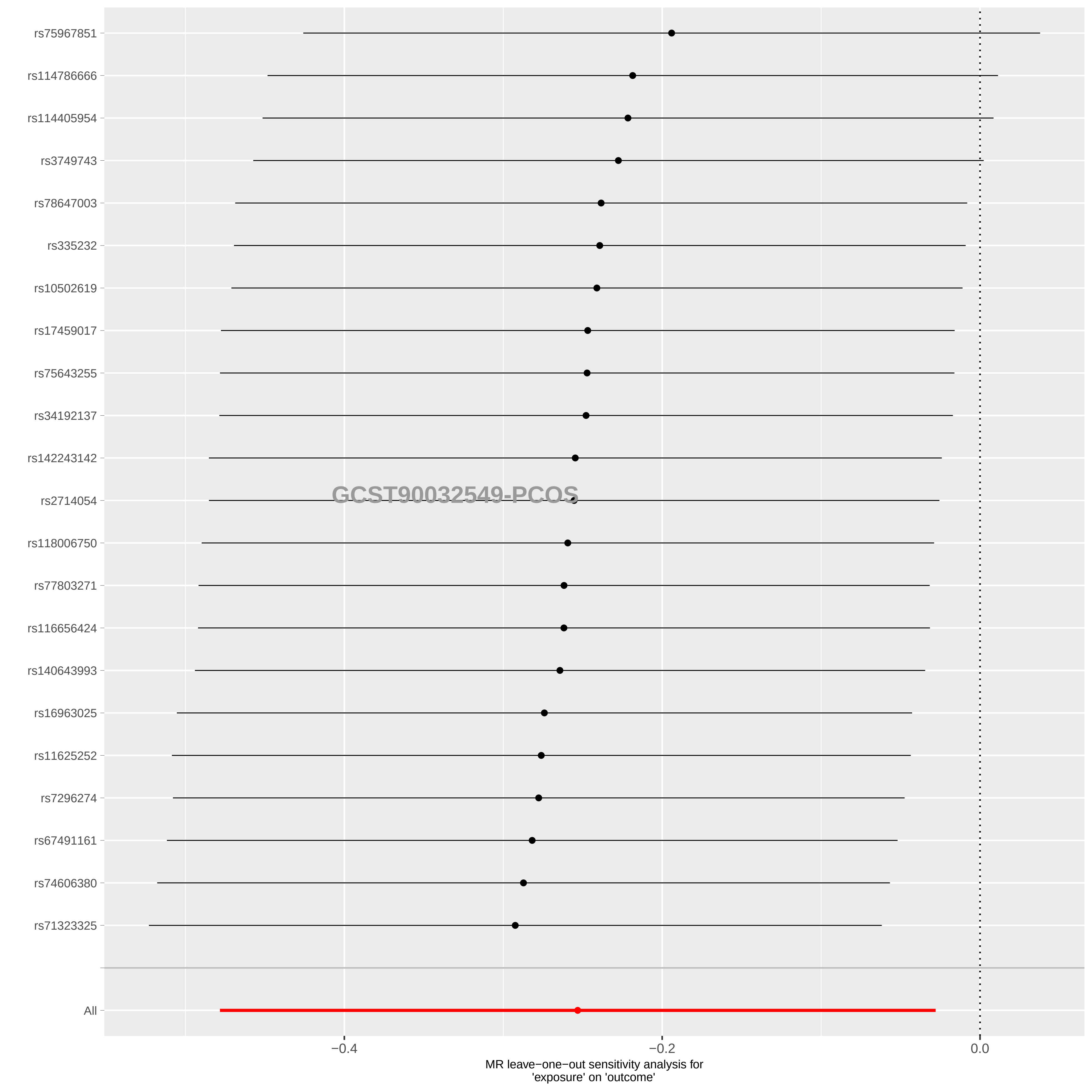

GCST90032549-PCOS
rs75967851
rs114786666
rs114405954
rs3749743
rs78647003
rs335232
rs10502619
rs17459017
rs75643255
rs34192137
rs142243142
rs2714054
rs118006750
rs77803271
rs116656424
rs140643993
rs16963025
rs11625252
rs7296274
rs67491161
rs74606380
rs71323325
All
−0.4
−0.2
0.0
MR leave−one−out sensitivity analysis for
'exposure' on 'outcome'

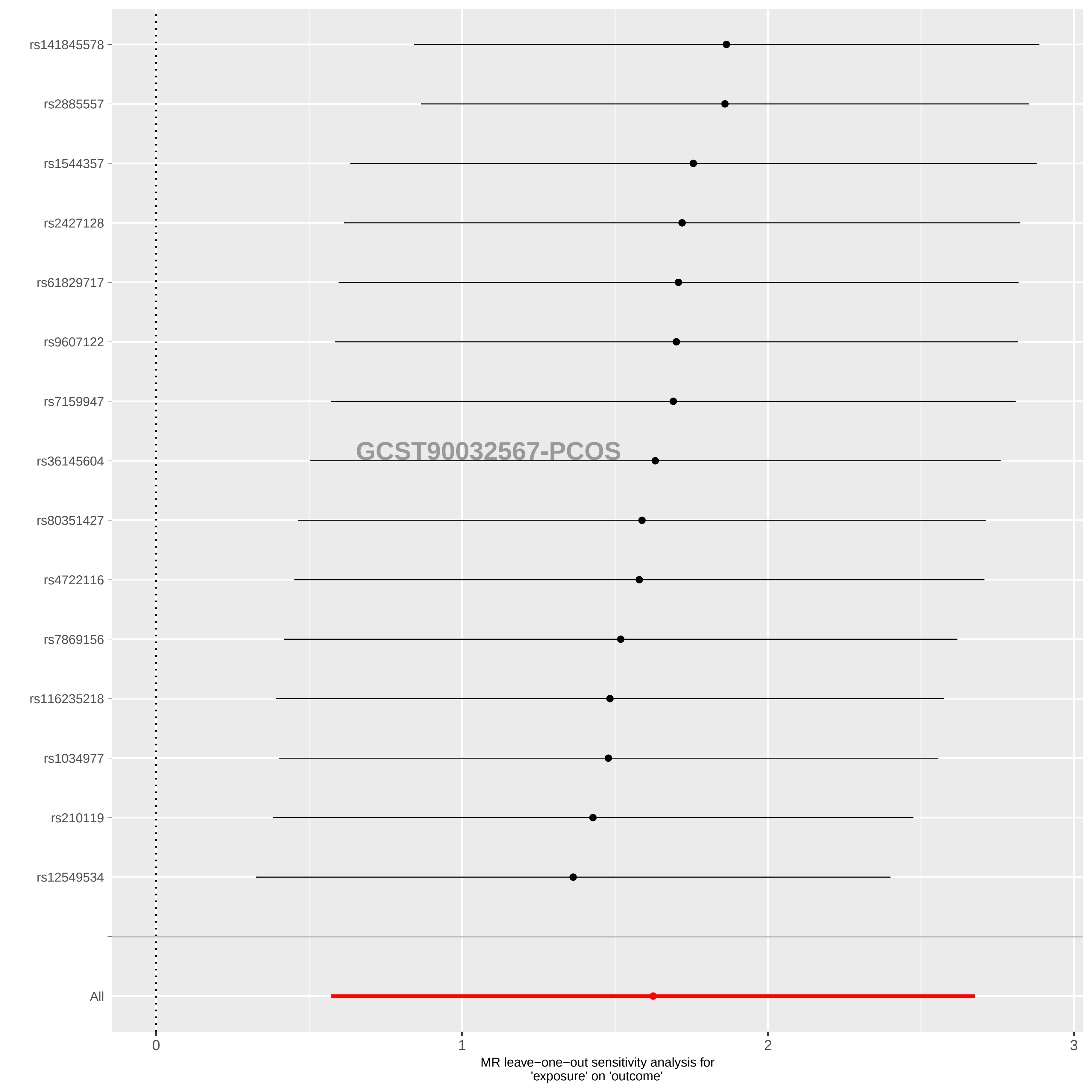
GCST90032567-PCOS
rs141845578
rs2885557
rs1544357
rs2427128
rs61829717
rs9607122
rs7159947
rs36145604
rs80351427
rs4722116
rs7869156
rs116235218
rs1034977
rs210119
rs12549534
All
0
1
2
3
MR leave−one−out sensitivity analysis for
'exposure' on 'outcome'

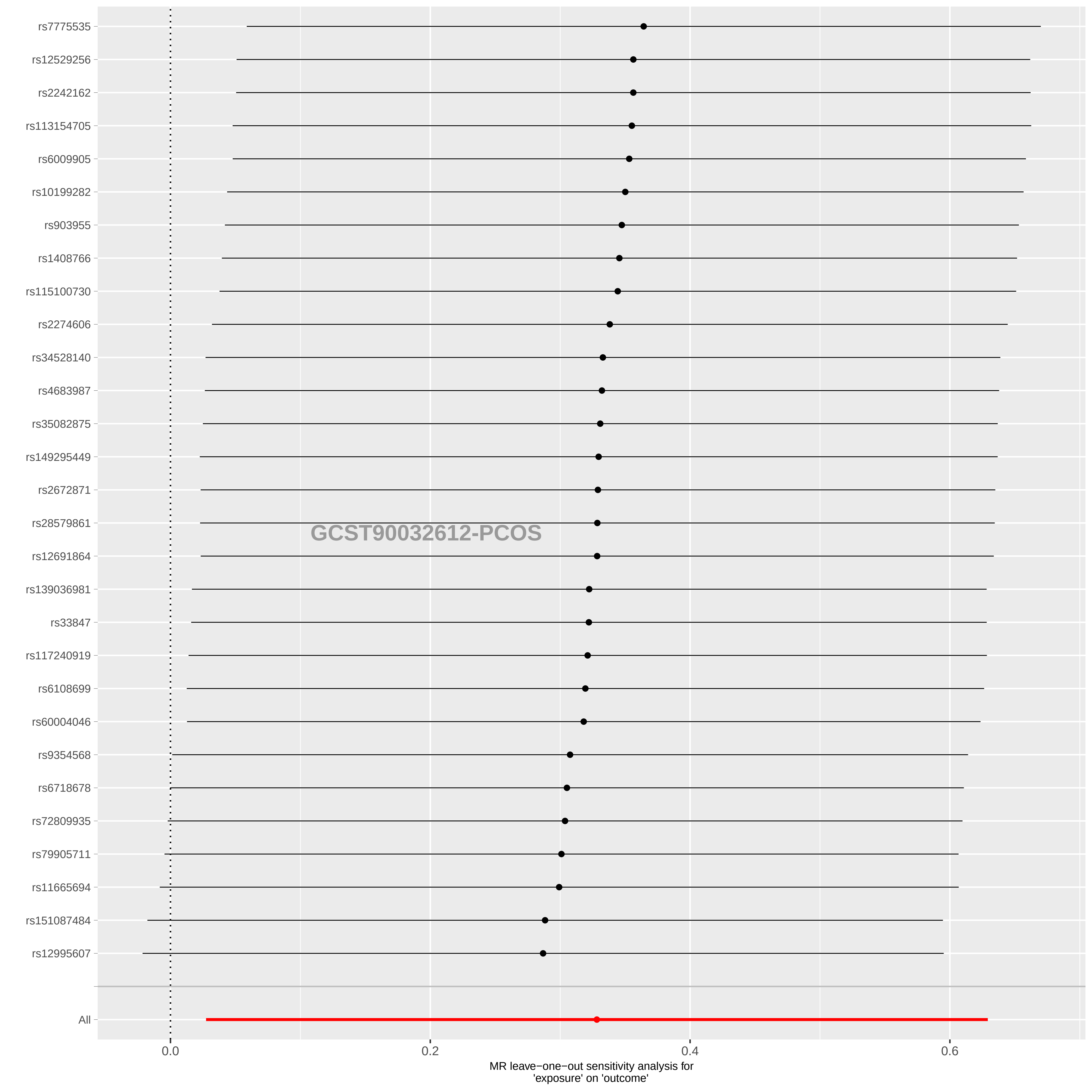
rs7775535
rs12529256
rs2242162
rs113154705
rs6009905
rs10199282
rs903955
rs1408766
rs115100730
rs2274606
rs34528140
rs4683987
rs35082875
rs149295449
rs2672871
rs28579861
rs12691864
rs139036981
rs33847
rs117240919
rs6108699
rs60004046
rs9354568
rs6718678
rs72809935
rs79905711
rs11665694
rs151087484
rs12995607
All
GCST90032612-PCOS
0.0
0.2
0.4
0.6
MR leave−one−out sensitivity analysis for
'exposure' on 'outcome'

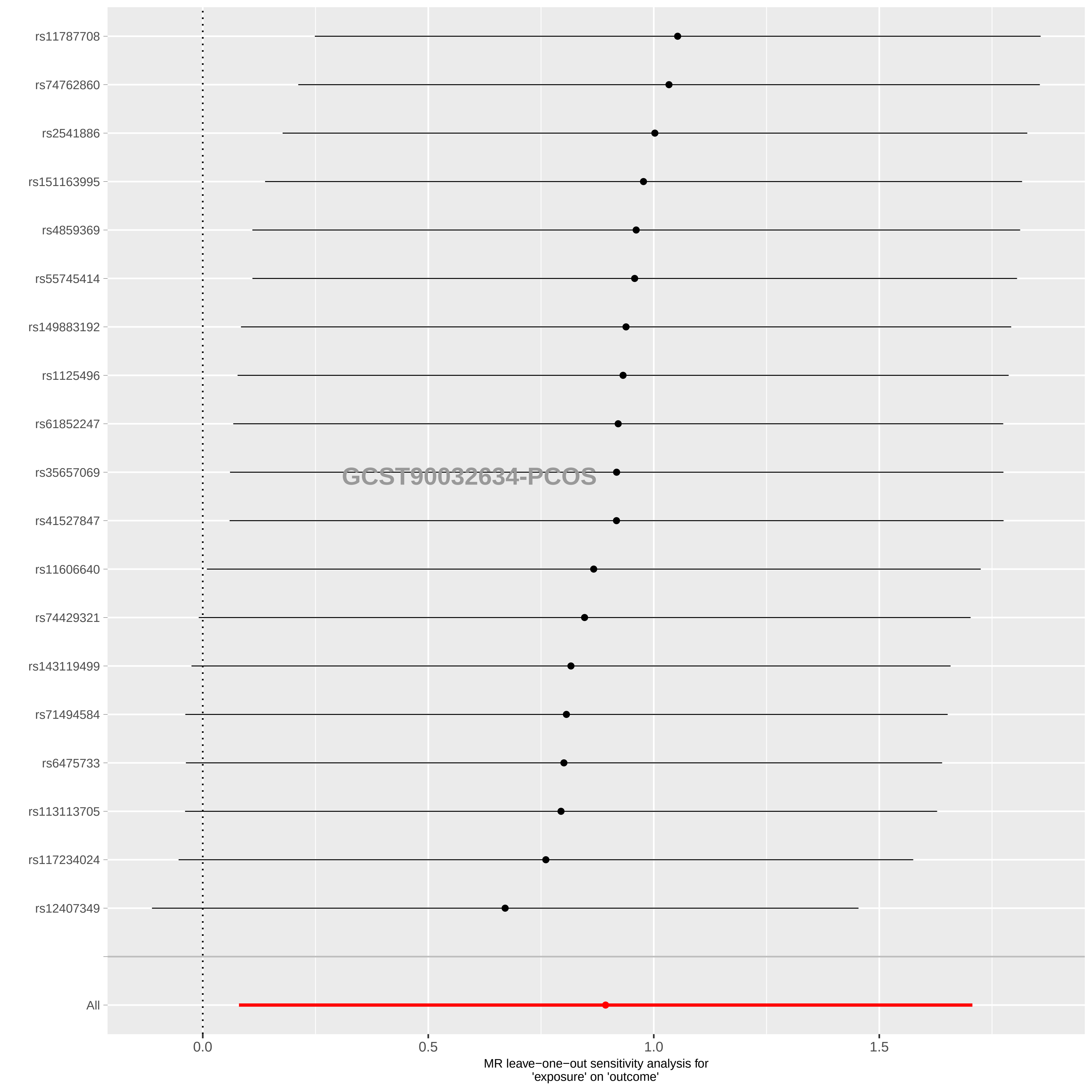

rs11787708
rs74762860
rs2541886
rs151163995
rs4859369
rs55745414
rs149883192
rs1125496
rs61852247
rs35657069
rs41527847
rs11606640
rs74429321
rs143119499
rs71494584
rs6475733
rs113113705
rs117234024
rs12407349
All
GCST90032634-PCOS
0.0
0.5
1.0
1.5
MR leave−one−out sensitivity analysis for
'exposure' on 'outcome'

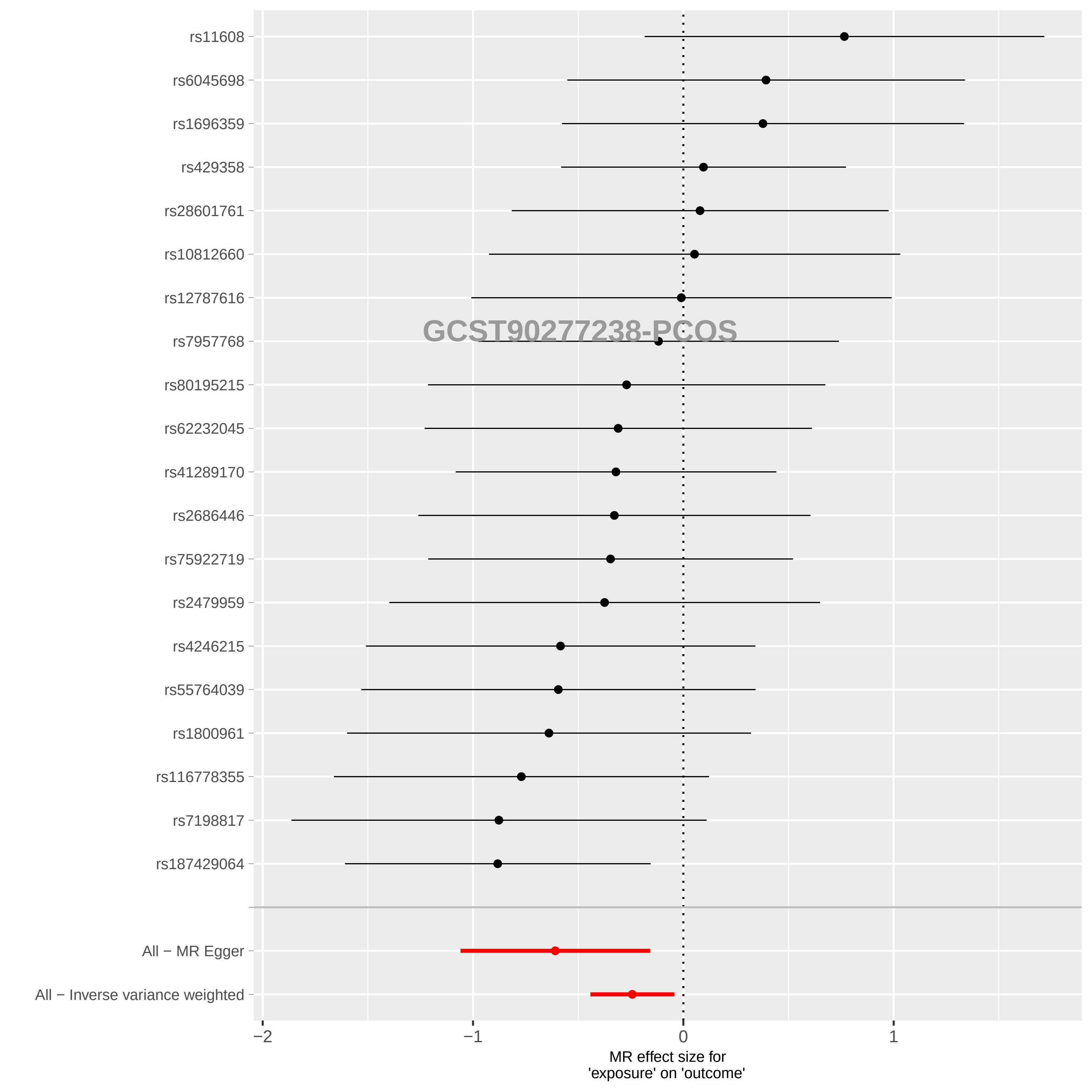

GCST90277238-PCOS
rs11608
rs6045698
rs1696359
rs429358
rs28601761
rs10812660
rs12787616
rs7957768
rs80195215
rs62232045
rs41289170
rs2686446
rs75922719
rs2479959
rs4246215
rs55764039
rs1800961
rs116778355
rs7198817
rs187429064
All − MR Egger
All − Inverse variance weighted
−2
−1
0
1
MR effect size for
'exposure' on 'outcome'

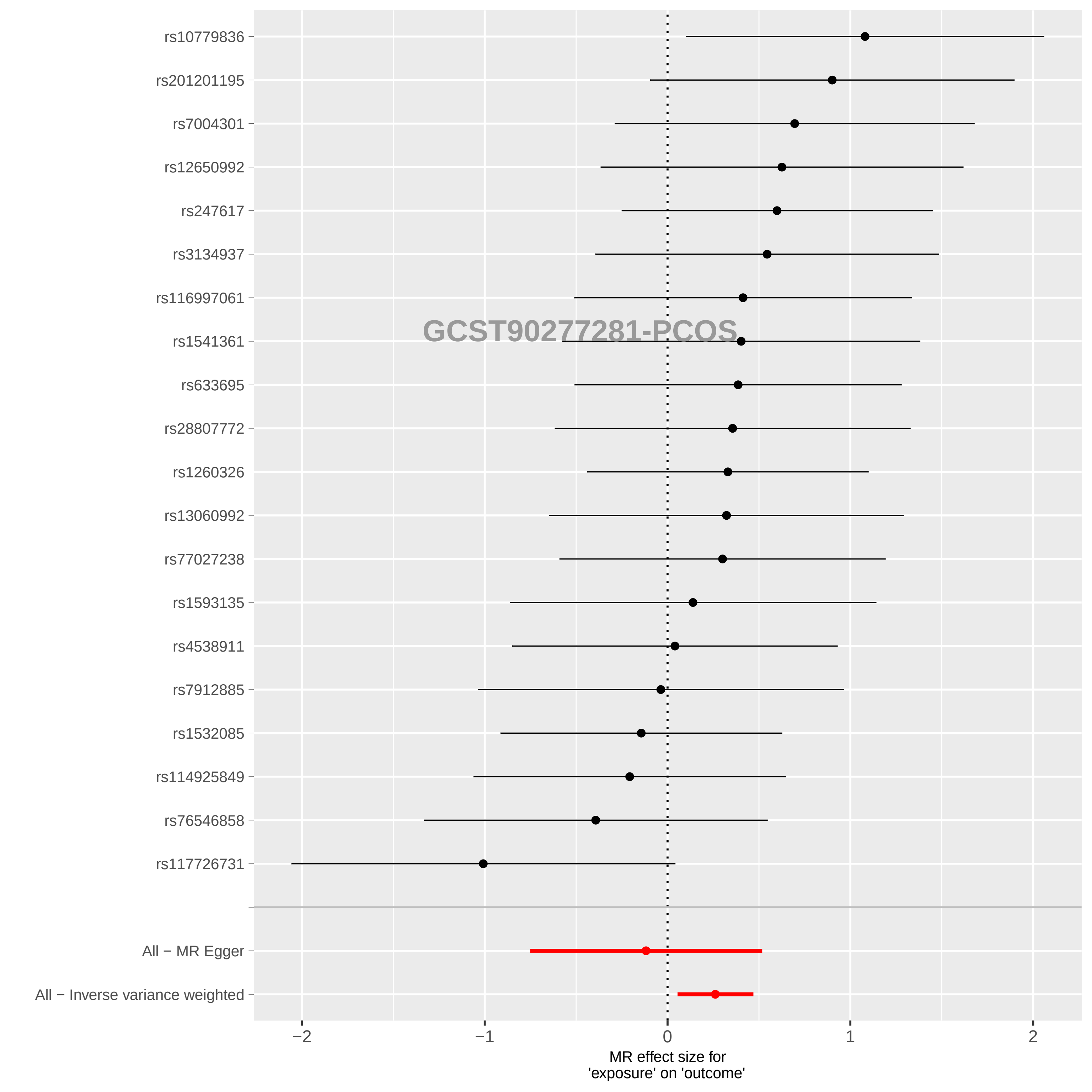
GCST90277281-PCOS
rs10779836
rs201201195
rs7004301
rs12650992
rs247617
rs3134937
rs116997061
rs1541361
rs633695
rs28807772
rs1260326
rs13060992
rs77027238
rs1593135
rs4538911
rs7912885
rs1532085
rs114925849
rs76546858
rs117726731
All − MR Egger
All − Inverse variance weighted
−2
−1
0
1
2
MR effect size for
'exposure' on 'outcome'

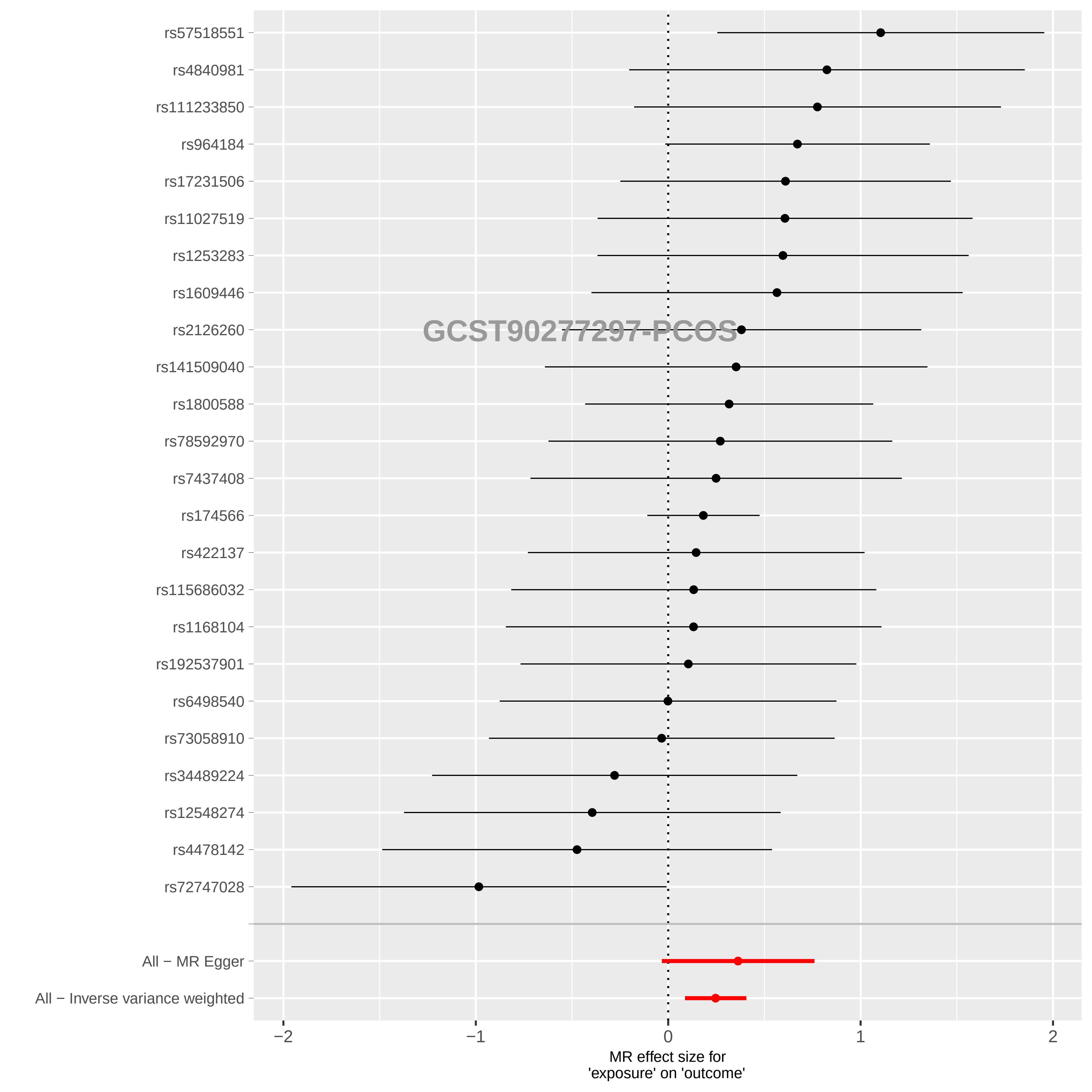
rs57518551
rs4840981
rs111233850
rs964184
rs17231506
rs11027519
rs1253283
rs1609446
rs2126260
rs141509040
rs1800588
rs78592970
rs7437408
rs174566
rs422137
rs115686032
rs1168104
rs192537901
rs6498540
rs73058910
rs34489224
rs12548274
rs4478142
rs72747028
All − MR Egger
All − Inverse variance weighted
GCST90277297-PCOS
−2
−1
0
1
2
MR effect size for
'exposure' on 'outcome'

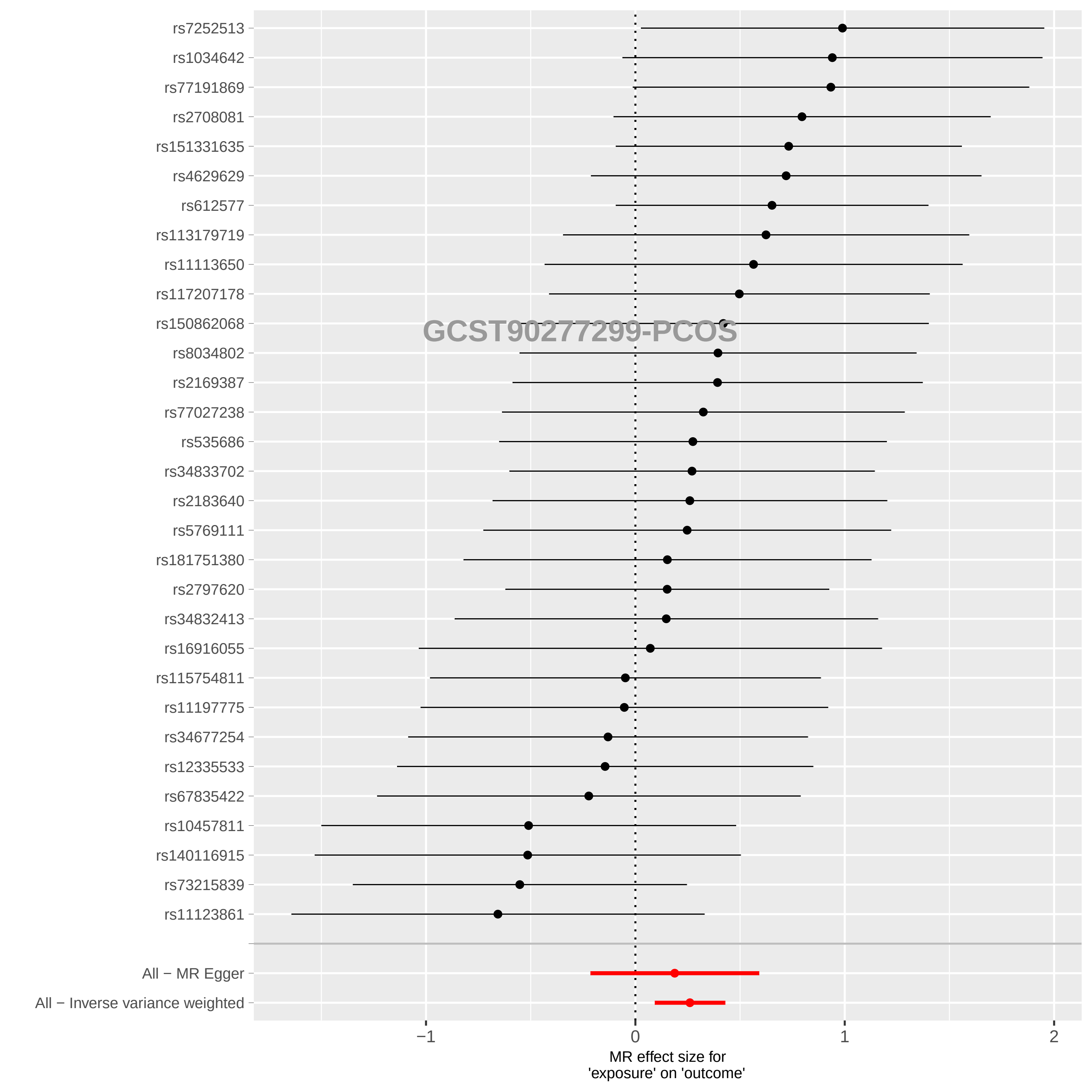

GCST90277299-PCOS
rs7252513
rs1034642
rs77191869
rs2708081
rs151331635
rs4629629
rs612577
rs113179719
rs11113650
rs117207178
rs150862068
rs8034802
rs2169387
rs77027238
rs535686
rs34833702
rs2183640
rs5769111
rs181751380
rs2797620
rs34832413
rs16916055
rs115754811
rs11197775
rs34677254
rs12335533
rs67835422
rs10457811
rs140116915
rs73215839
rs11123861
All − MR Egger
All − Inverse variance weighted
−1
0
1
2
MR effect size for
'exposure' on 'outcome'

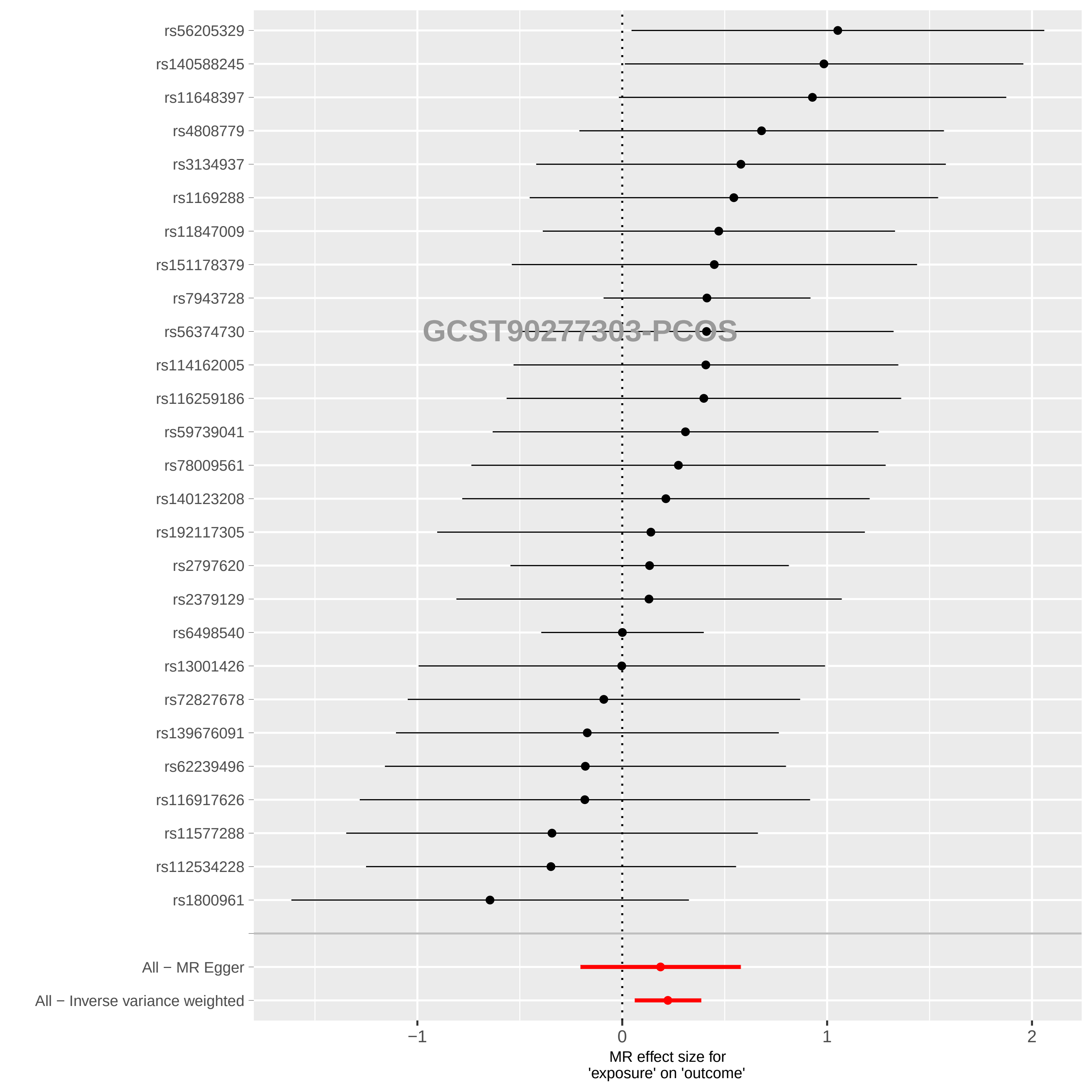

GCST90277303-PCOS
rs56205329
rs140588245
rs11648397
rs4808779
rs3134937
rs1169288
rs11847009
rs151178379
rs7943728
rs56374730
rs114162005
rs116259186
rs59739041
rs78009561
rs140123208
rs192117305
rs2797620
rs2379129
rs6498540
rs13001426
rs72827678
rs139676091
rs62239496
rs116917626
rs11577288
rs112534228
rs1800961
All − MR Egger
All − Inverse variance weighted
−1
0
1
2
MR effect size for
'exposure' on 'outcome'

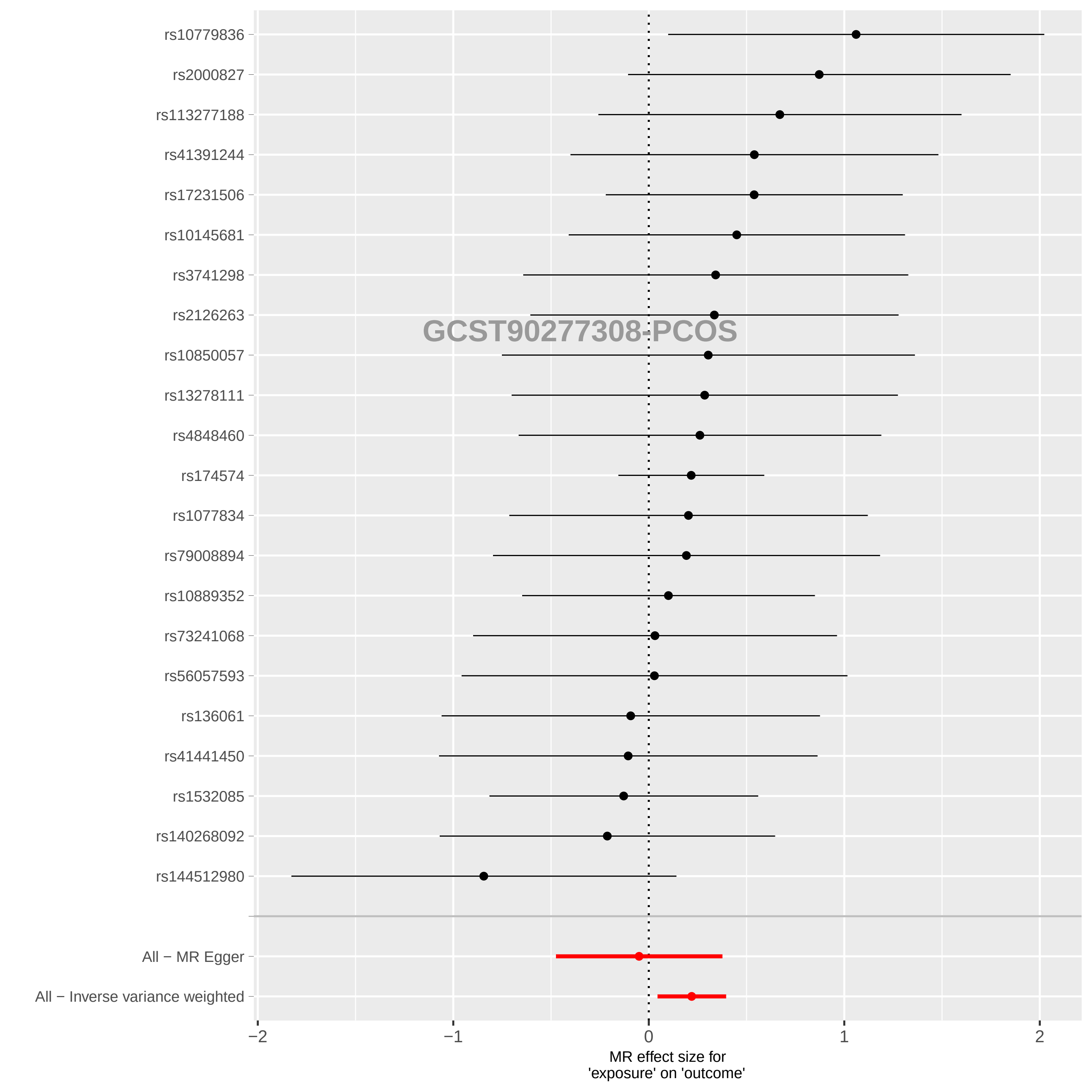

GCST90277308-PCOS
rs10779836
rs2000827
rs113277188
rs41391244
rs17231506
rs10145681
rs3741298
rs2126263
rs10850057
rs13278111
rs4848460
rs174574
rs1077834
rs79008894
rs10889352
rs73241068
rs56057593
rs136061
rs41441450
rs1532085
rs140268092
rs144512980
All − MR Egger
All − Inverse variance weighted
−2
−1
0
1
2
MR effect size for
'exposure' on 'outcome'

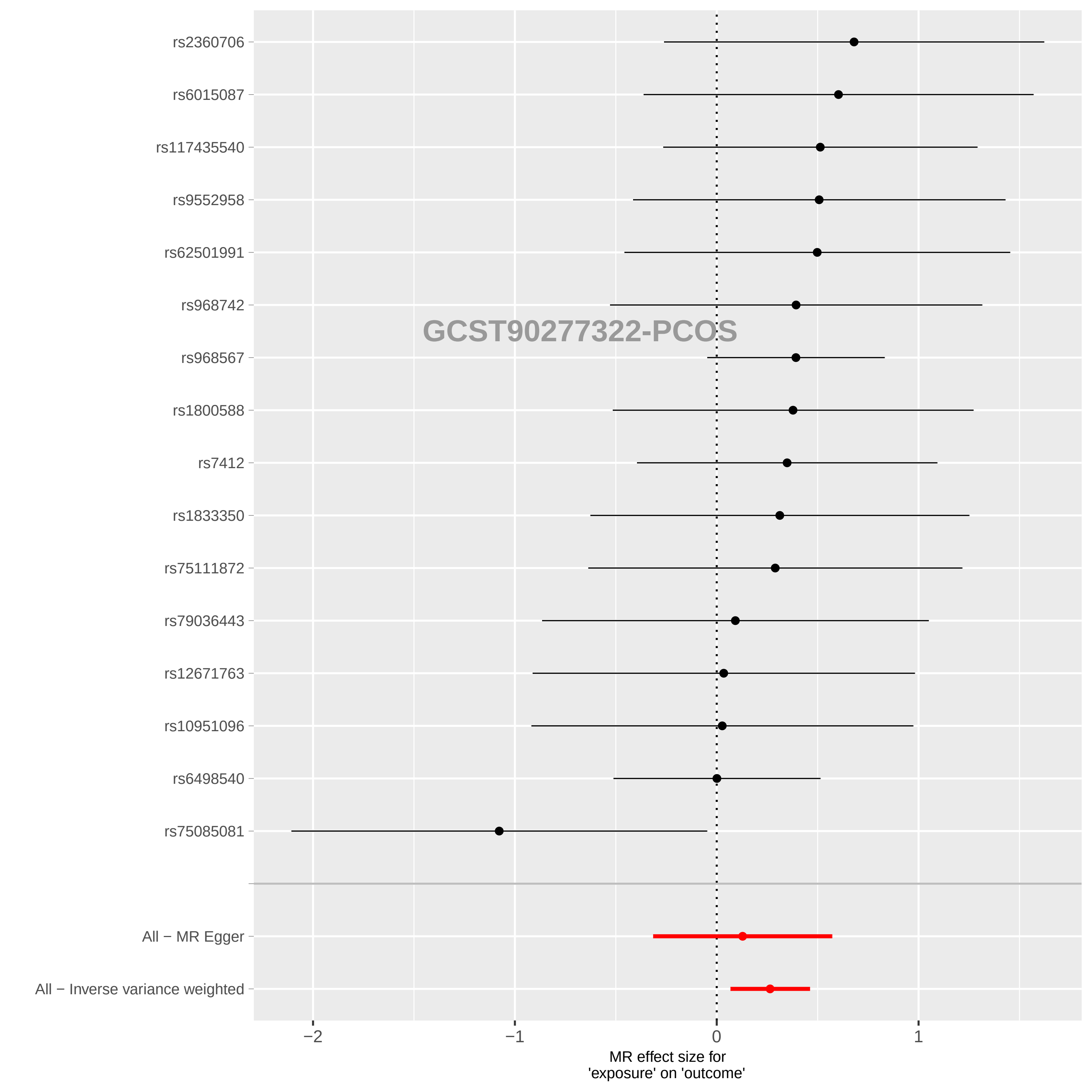

GCST90277322-PCOS
rs2360706
rs6015087
rs117435540
rs9552958
rs62501991
rs968742
rs968567
rs1800588
rs7412
rs1833350
rs75111872
rs79036443
rs12671763
rs10951096
rs6498540
rs75085081
All − MR Egger
All − Inverse variance weighted
−2
−1
0
1
MR effect size for
'exposure' on 'outcome'

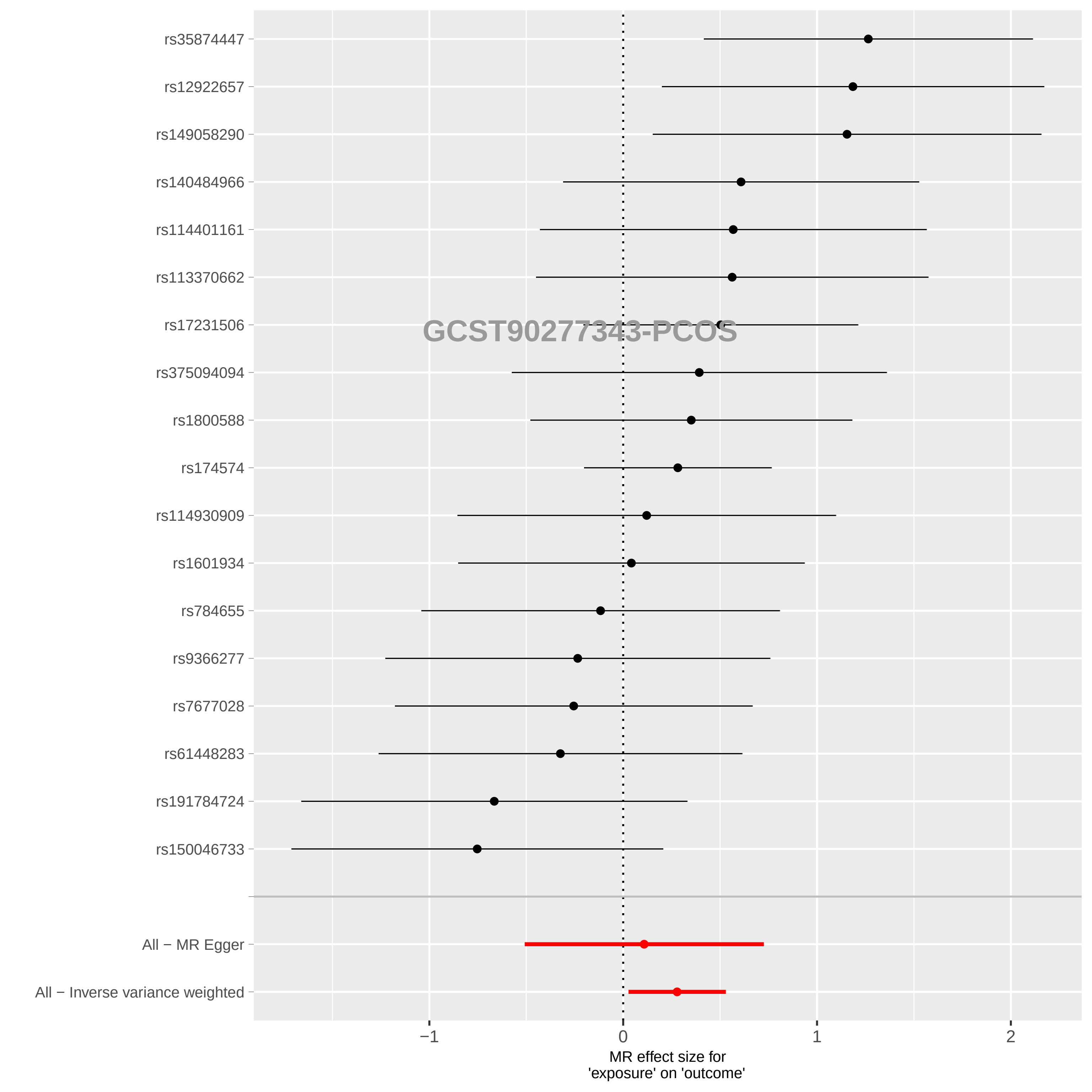
rs35874447
rs12922657
rs149058290
rs140484966
rs114401161
rs113370662
rs17231506
GCST90277343-PCOS
rs375094094
rs1800588
rs174574
rs114930909
rs1601934
rs784655
rs9366277
rs7677028
rs61448283
rs191784724
rs150046733
All − MR Egger
All − Inverse variance weighted
−1
0
1
2
MR effect size for
'exposure' on 'outcome'

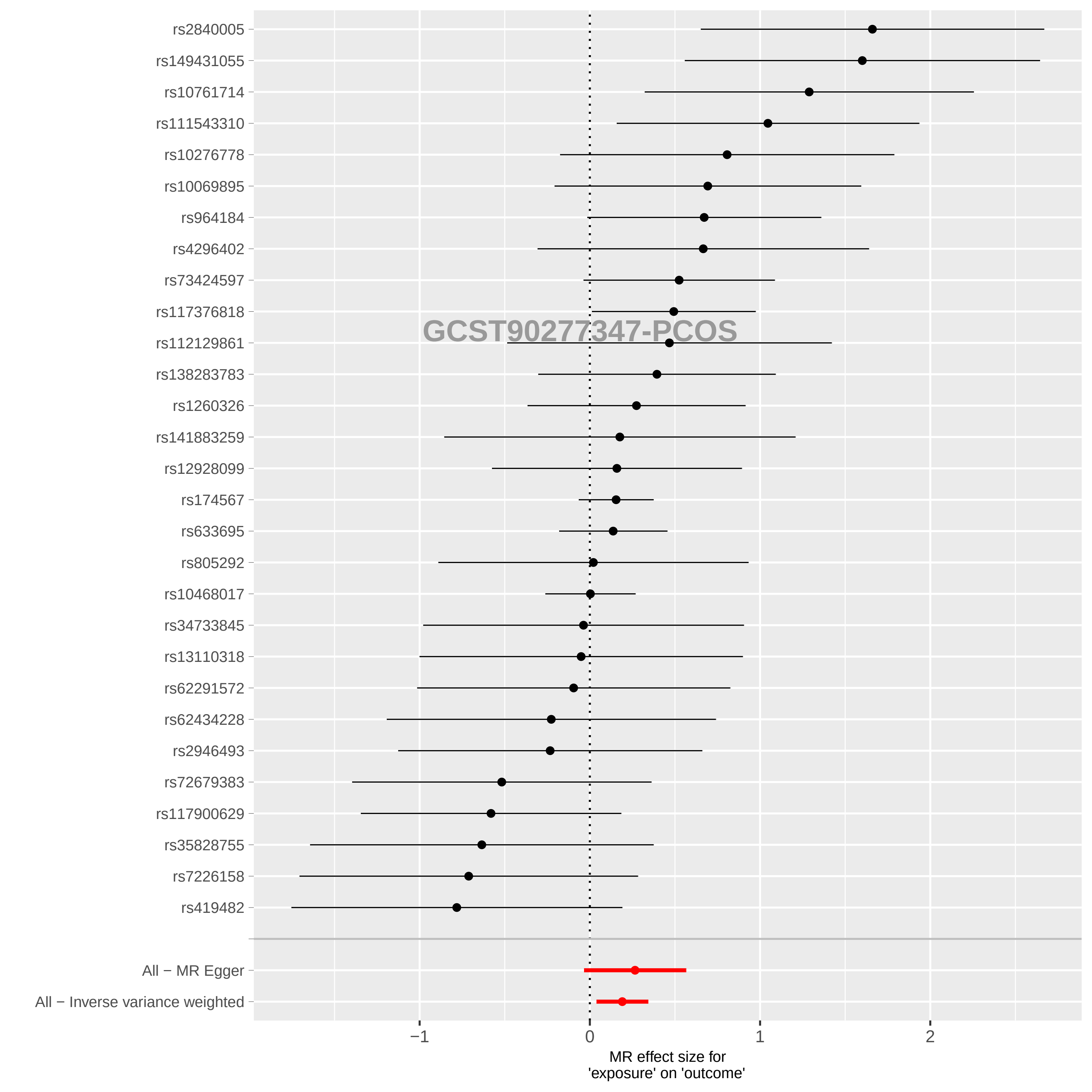
GCST90277347-PCOS
rs2840005
rs149431055
rs10761714
rs111543310
rs10276778
rs10069895
rs964184
rs4296402
rs73424597
rs117376818
rs112129861
rs138283783
rs1260326
rs141883259
rs12928099
rs174567
rs633695
rs805292
rs10468017
rs34733845
rs13110318
rs62291572
rs62434228
rs2946493
rs72679383
rs117900629
rs35828755
rs7226158
rs419482
All − MR Egger
All − Inverse variance weighted
−1
0
1
2
MR effect size for
'exposure' on 'outcome'

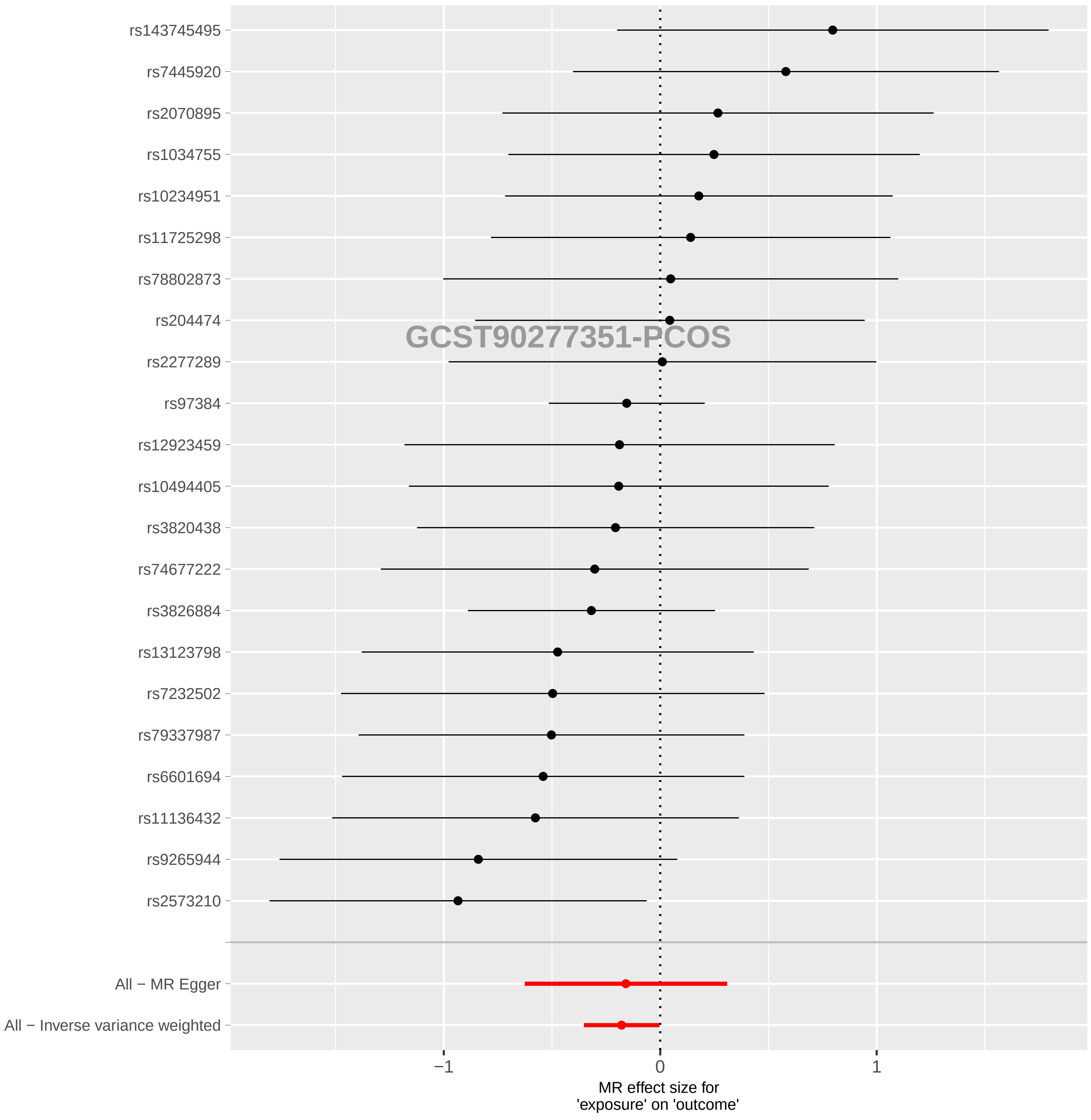
GCST90277351-PCOS
rs143745495
rs7445920
rs2070895
rs1034755
rs10234951
rs11725298
rs78802873
rs204474
rs2277289
rs97384
rs12923459
rs10494405
rs3820438
rs74677222
rs3826884
rs13123798
rs7232502
rs79337987
rs6601694
rs11136432
rs9265944
rs2573210
All − MR Egger
All − Inverse variance weighted
−1
0
1
MR effect size for
'exposure' on 'outcome'

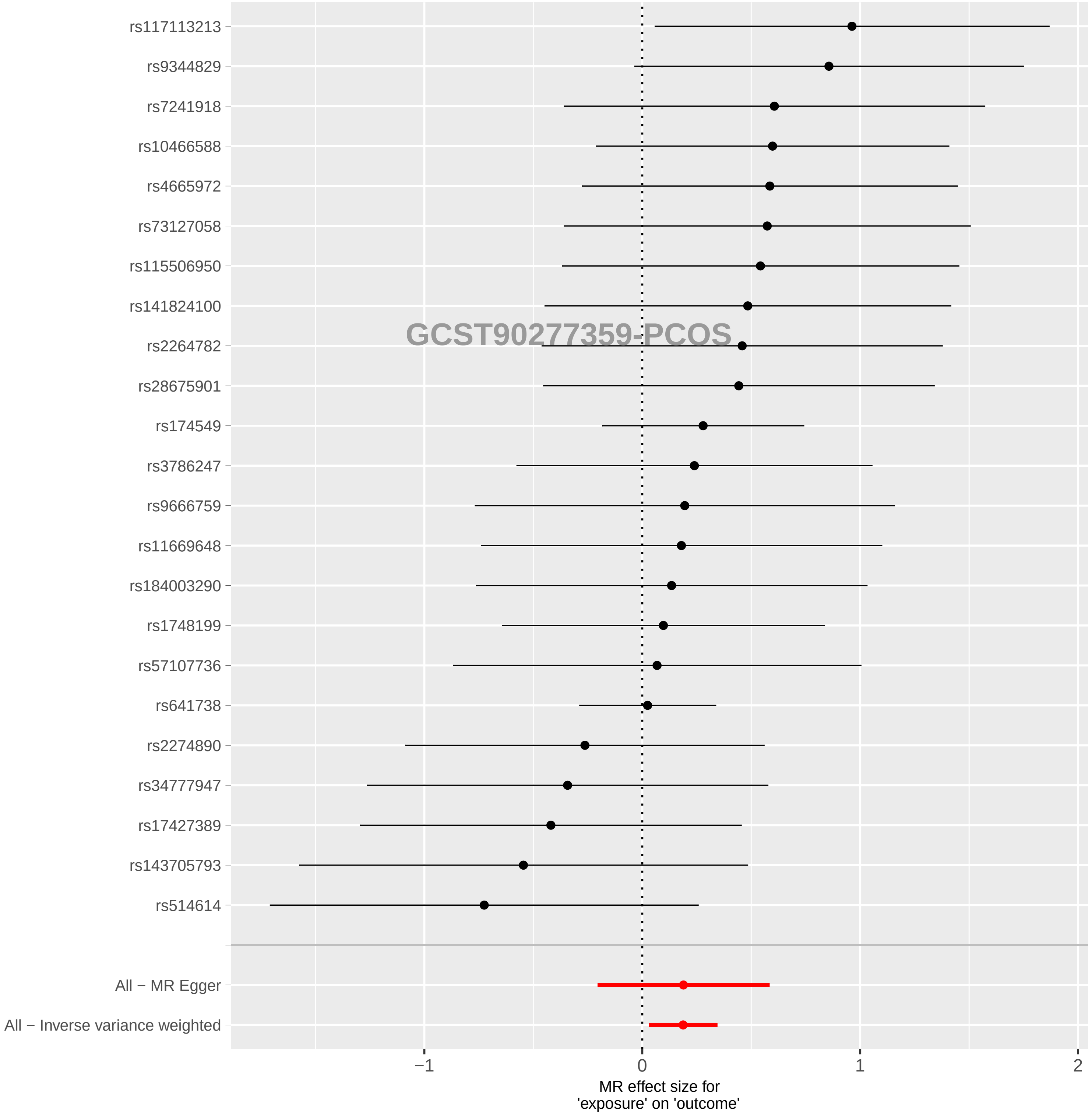
GCST90277359-PCOS
rs117113213
rs9344829
rs7241918
rs10466588
rs4665972
rs73127058
rs115506950
rs141824100
rs2264782
rs28675901
rs174549
rs3786247
rs9666759
rs11669648
rs184003290
rs1748199
rs57107736
rs641738
rs2274890
rs34777947
rs17427389
rs143705793
rs514614
All − MR Egger
All − Inverse variance weighted
−1
0
1
2
MR effect size for
'exposure' on 'outcome'

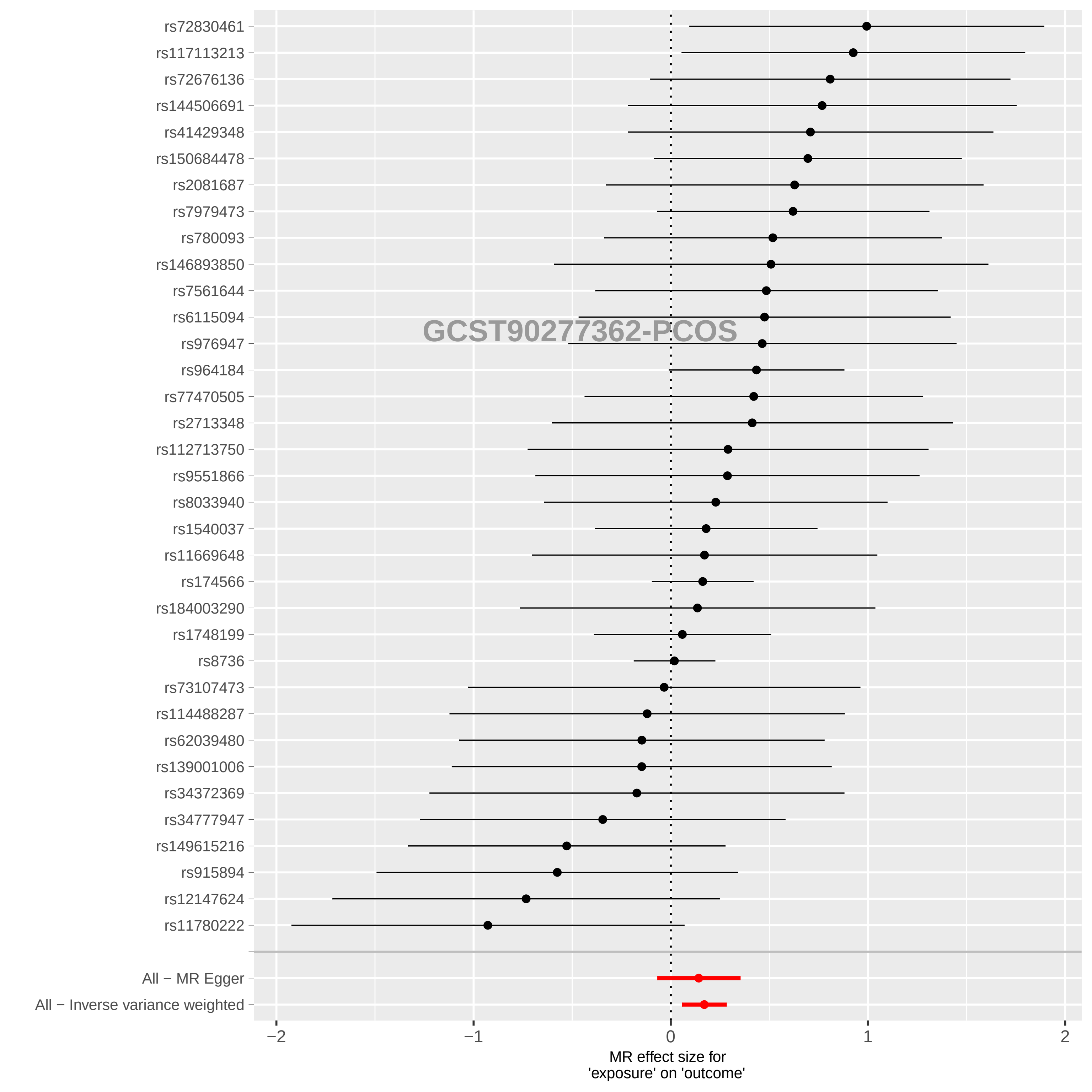
GCST90277362-PCOS
rs72830461
rs117113213
rs72676136
rs144506691
rs41429348
rs150684478
rs2081687
rs7979473
rs780093
rs146893850
rs7561644
rs6115094
rs976947
rs964184
rs77470505
rs2713348
rs112713750
rs9551866
rs8033940
rs1540037
rs11669648
rs174566
rs184003290
rs1748199
rs8736
rs73107473
rs114488287
rs62039480
rs139001006
rs34372369
rs34777947
rs149615216
rs915894
rs12147624
rs11780222
All − MR Egger
All − Inverse variance weighted
−2
−1
0
1
2
MR effect size for
'exposure' on 'outcome'

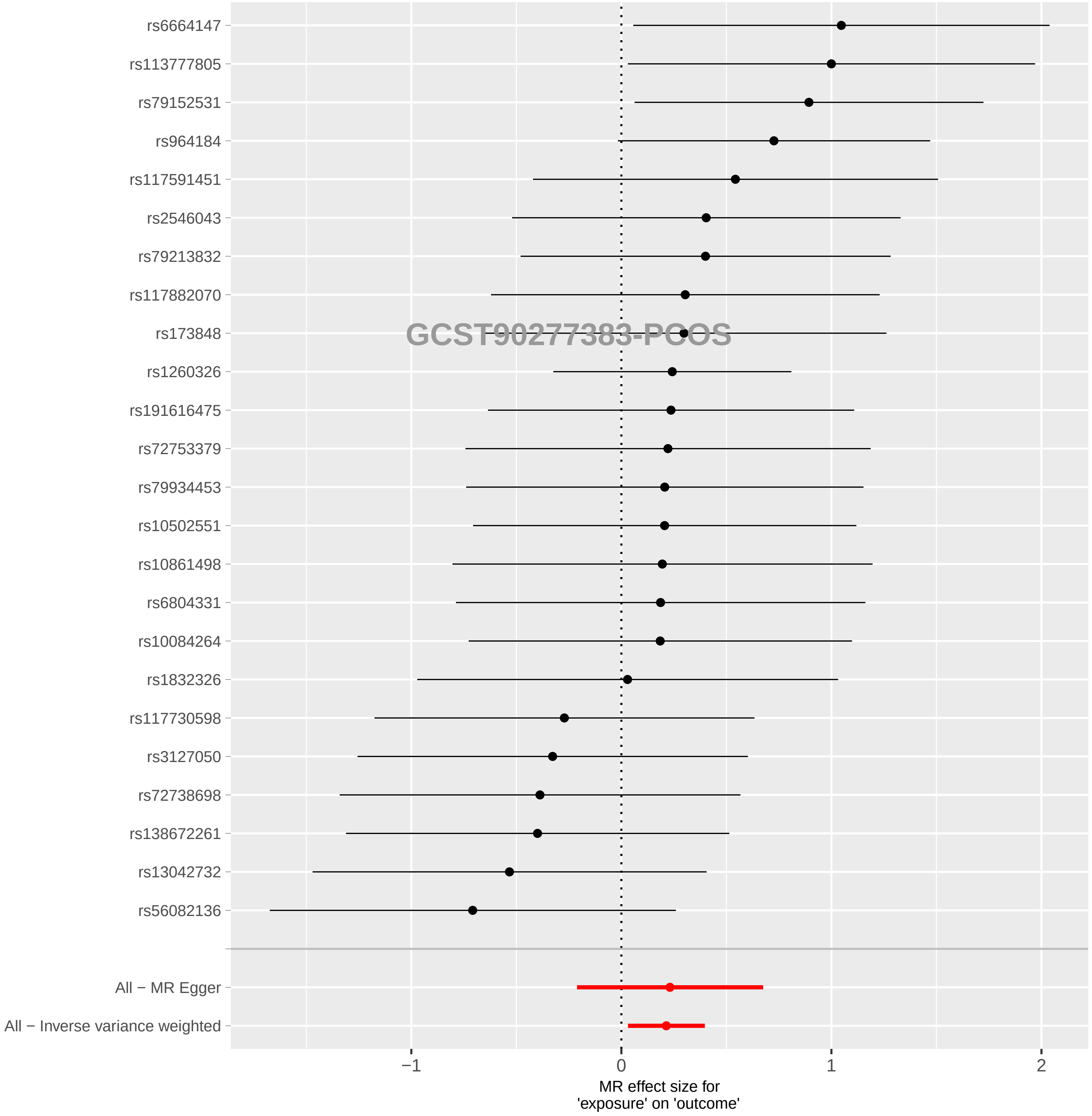

GCST90277383-PCOS
rs6664147
rs113777805
rs79152531
rs964184
rs117591451
rs2546043
rs79213832
rs117882070
rs173848
rs1260326
rs191616475
rs72753379
rs79934453
rs10502551
rs10861498
rs6804331
rs10084264
rs1832326
rs117730598
rs3127050
rs72738698
rs138672261
rs13042732
rs56082136
All − MR Egger
All − Inverse variance weighted
−1
0
1
2
MR effect size for
'exposure' on 'outcome'

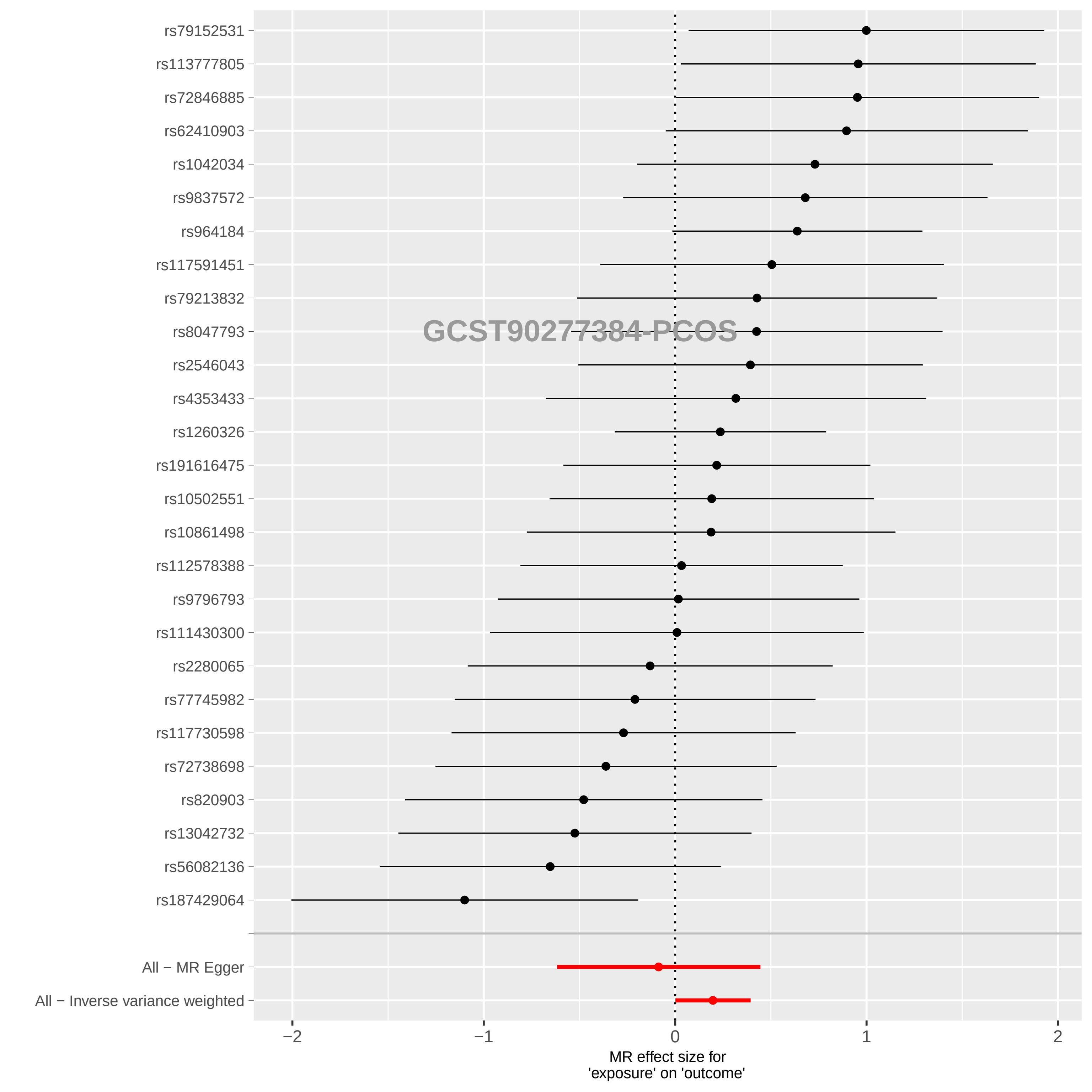

GCST90277384-PCOS
rs79152531
rs113777805
rs72846885
rs62410903
rs1042034
rs9837572
rs964184
rs117591451
rs79213832
rs8047793
rs2546043
rs4353433
rs1260326
rs191616475
rs10502551
rs10861498
rs112578388
rs9796793
rs111430300
rs2280065
rs77745982
rs117730598
rs72738698
rs820903
rs13042732
rs56082136
rs187429064
All − MR Egger
All − Inverse variance weighted
−2
−1
0
1
2
MR effect size for
'exposure' on 'outcome'

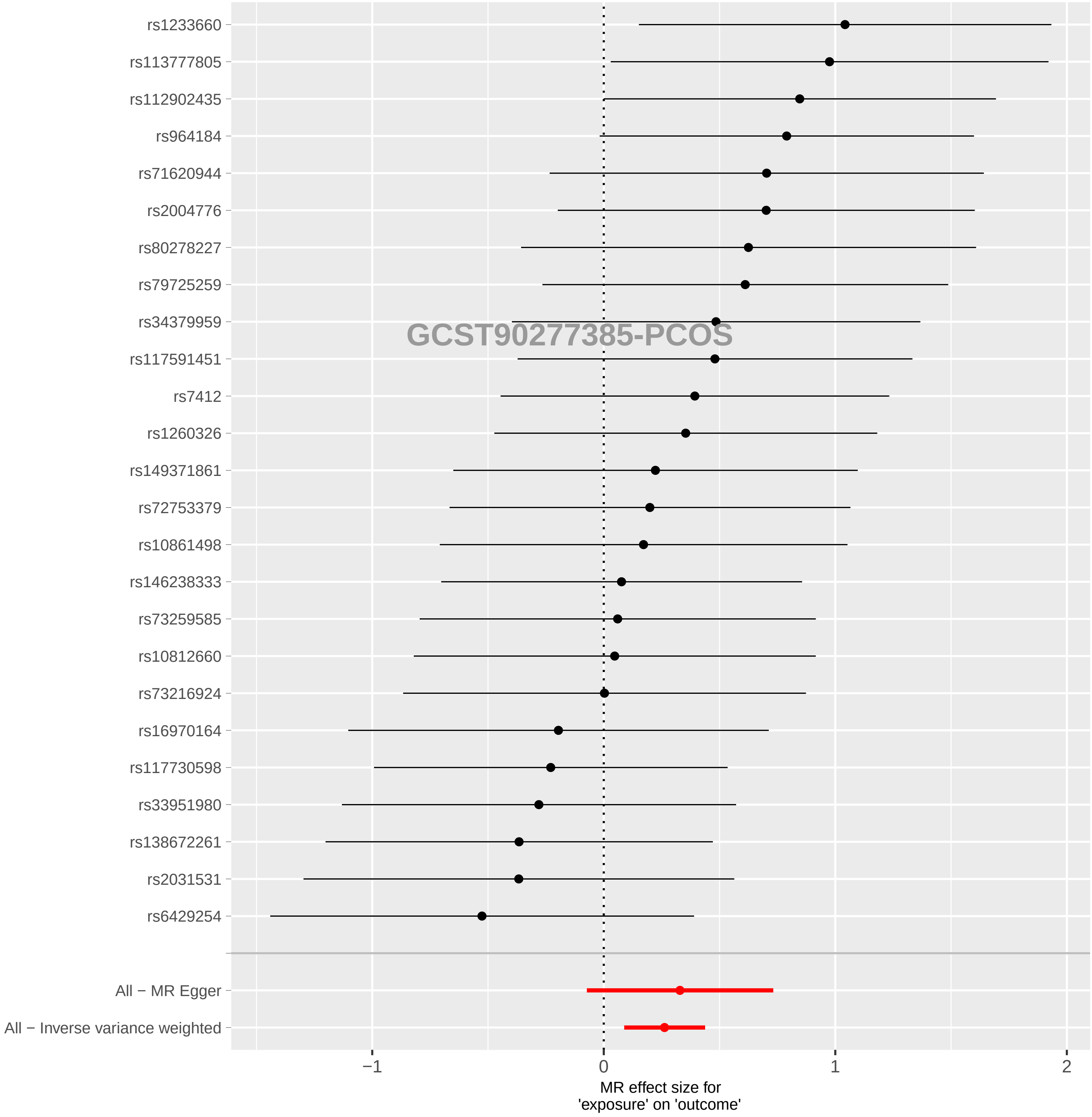

GCST90277385-PCOS
rs1233660
rs113777805
rs112902435
rs964184
rs71620944
rs2004776
rs80278227
rs79725259
rs34379959
rs117591451
rs7412
rs1260326
rs149371861
rs72753379
rs10861498
rs146238333
rs73259585
rs10812660
rs73216924
rs16970164
rs117730598
rs33951980
rs138672261
rs2031531
rs6429254
All − MR Egger
All − Inverse variance weighted
−1
0
1
2
MR effect size for
'exposure' on 'outcome'

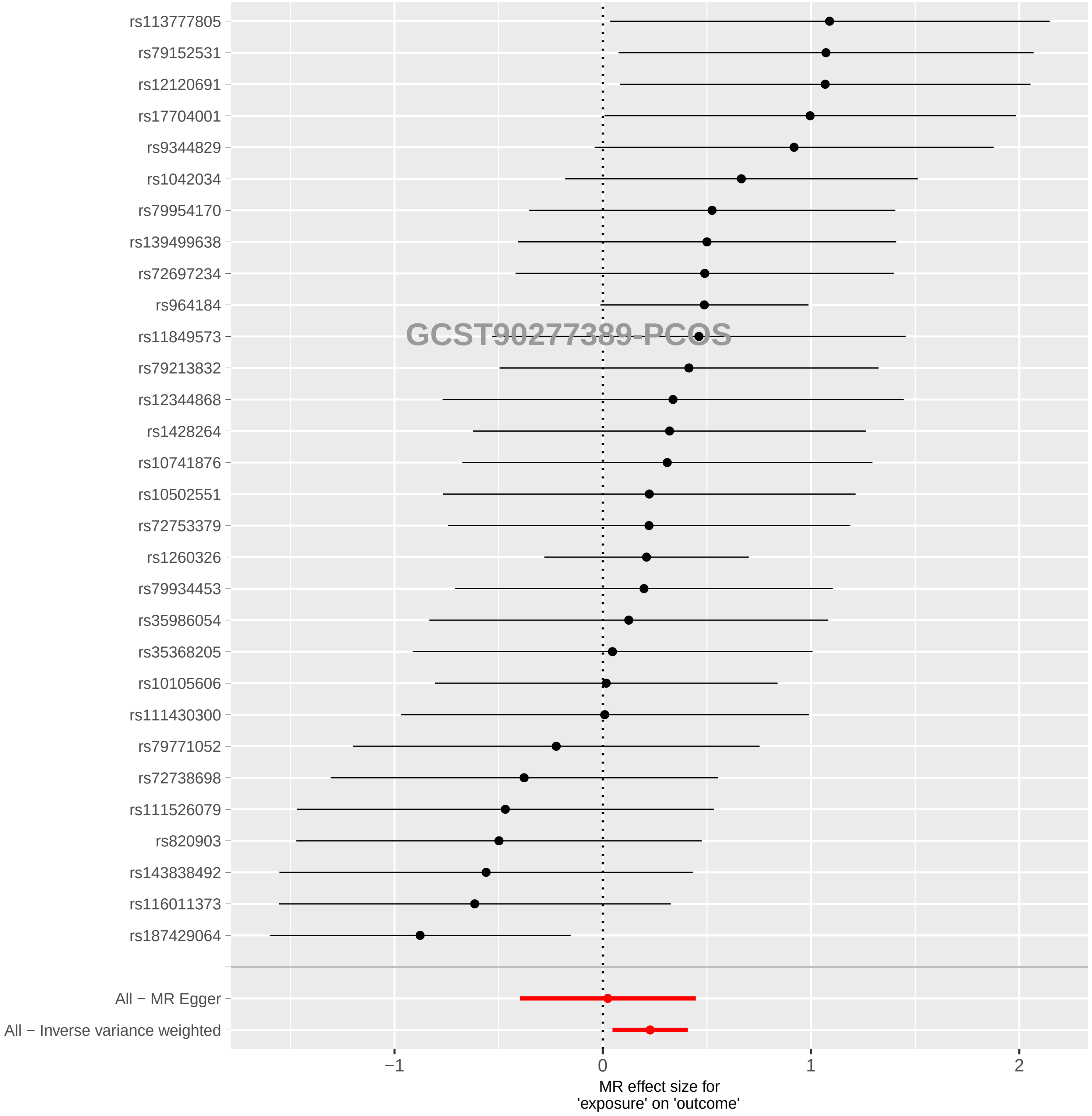

GCST90277389-PCOS
rs113777805
rs79152531
rs12120691
rs17704001
rs9344829
rs1042034
rs79954170
rs139499638
rs72697234
rs964184
rs11849573
rs79213832
rs12344868
rs1428264
rs10741876
rs10502551
rs72753379
rs1260326
rs79934453
rs35986054
rs35368205
rs10105606
rs111430300
rs79771052
rs72738698
rs111526079
rs820903
rs143838492
rs116011373
rs187429064
All − MR Egger
All − Inverse variance weighted
−1
0
1
2
MR effect size for
'exposure' on 'outcome'

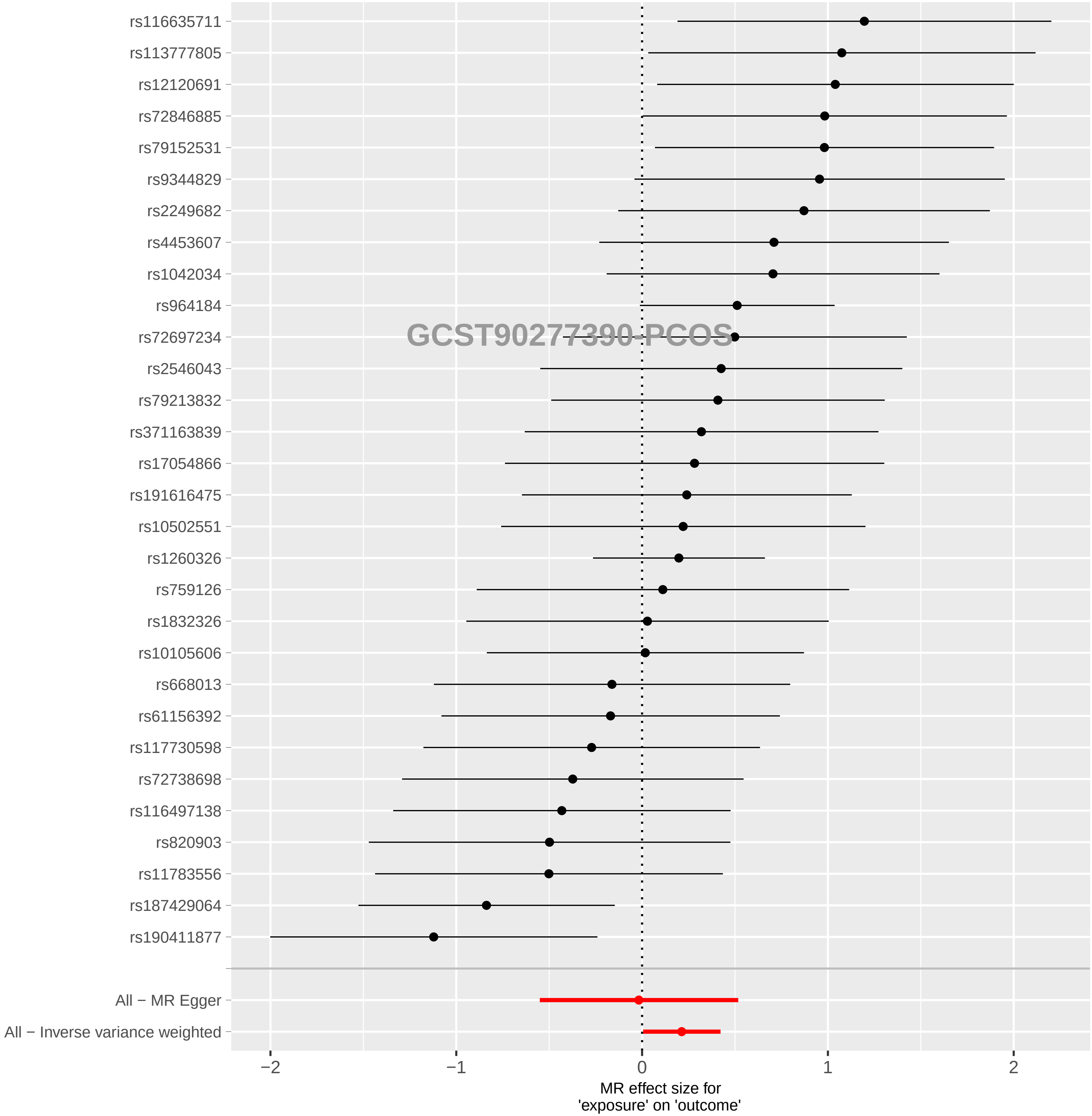
GCST90277390-PCOS
rs116635711
rs113777805
rs12120691
rs72846885
rs79152531
rs9344829
rs2249682
rs4453607
rs1042034
rs964184
rs72697234
rs2546043
rs79213832
rs371163839
rs17054866
rs191616475
rs10502551
rs1260326
rs759126
rs1832326
rs10105606
rs668013
rs61156392
rs117730598
rs72738698
rs116497138
rs820903
rs11783556
rs187429064
rs190411877
All − MR Egger
All − Inverse variance weighted
−2
−1
0
1
2
MR effect size for
'exposure' on 'outcome'

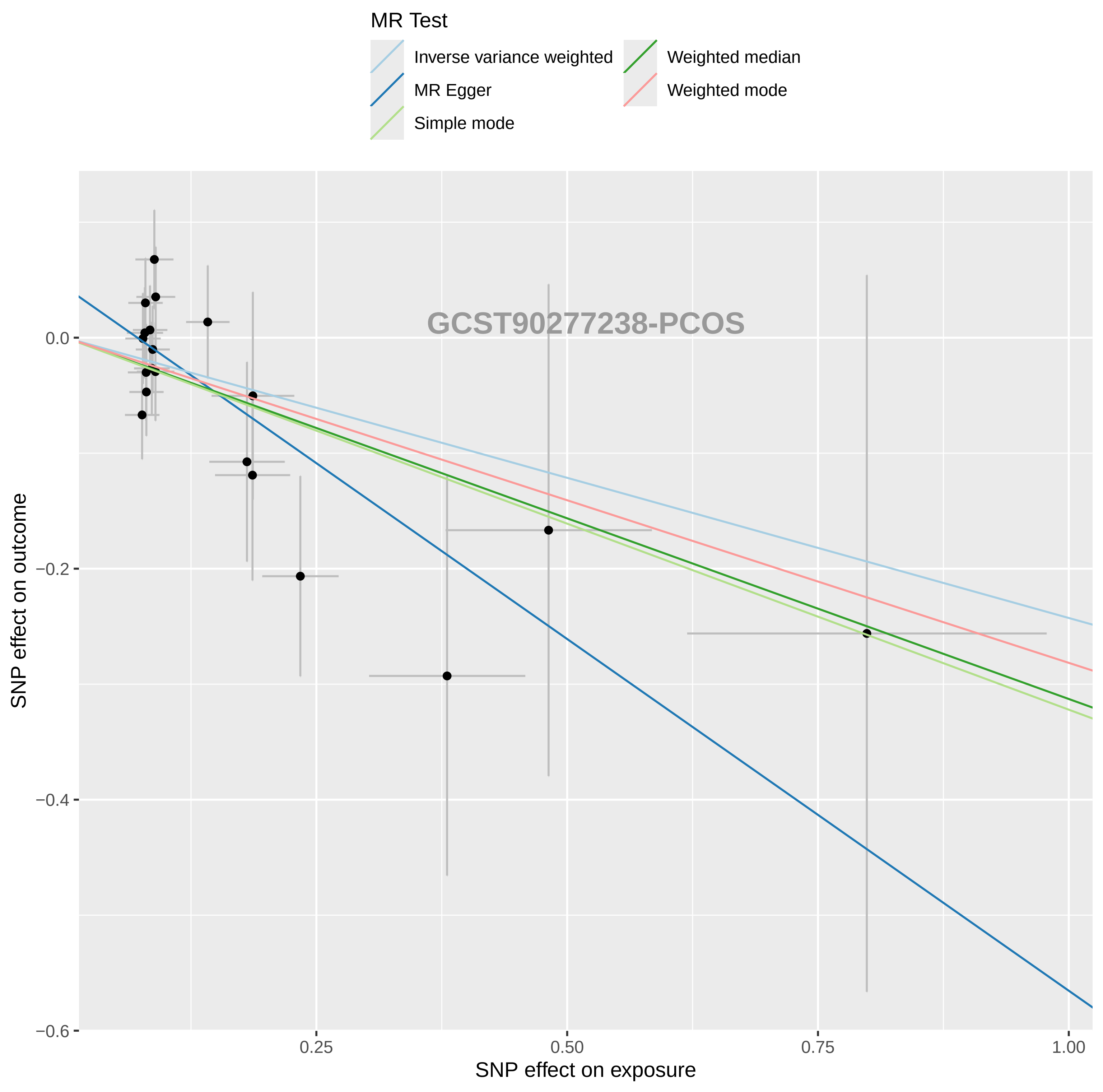
MR Test
Inverse variance weighted
MR Egger
Simple mode
Weighted median
Weighted mode
GCST90277238-PCOS
SNP effect on outcome
0.0
−0.2
−0.4
−0.6
0.25
0.50
0.75
1.00
SNP effect on exposure

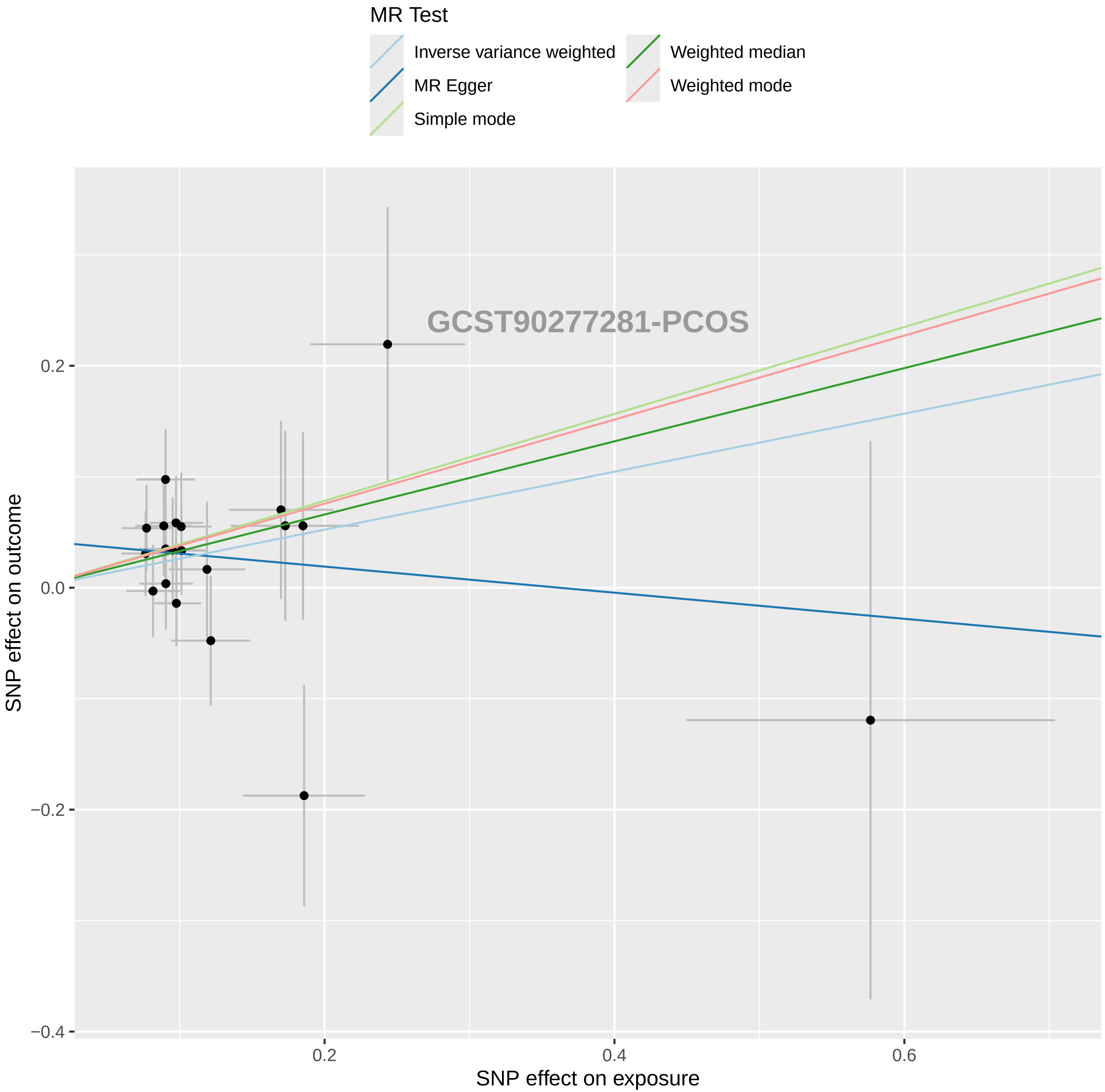

MR Test
Inverse variance weighted
MR Egger
Simple mode
Weighted median
Weighted mode
GCST90277281-PCOS
SNP effect on outcome
SNP effect on exposure
0.2
0.0
−0.2
−0.4
0.2
0.4
0.6

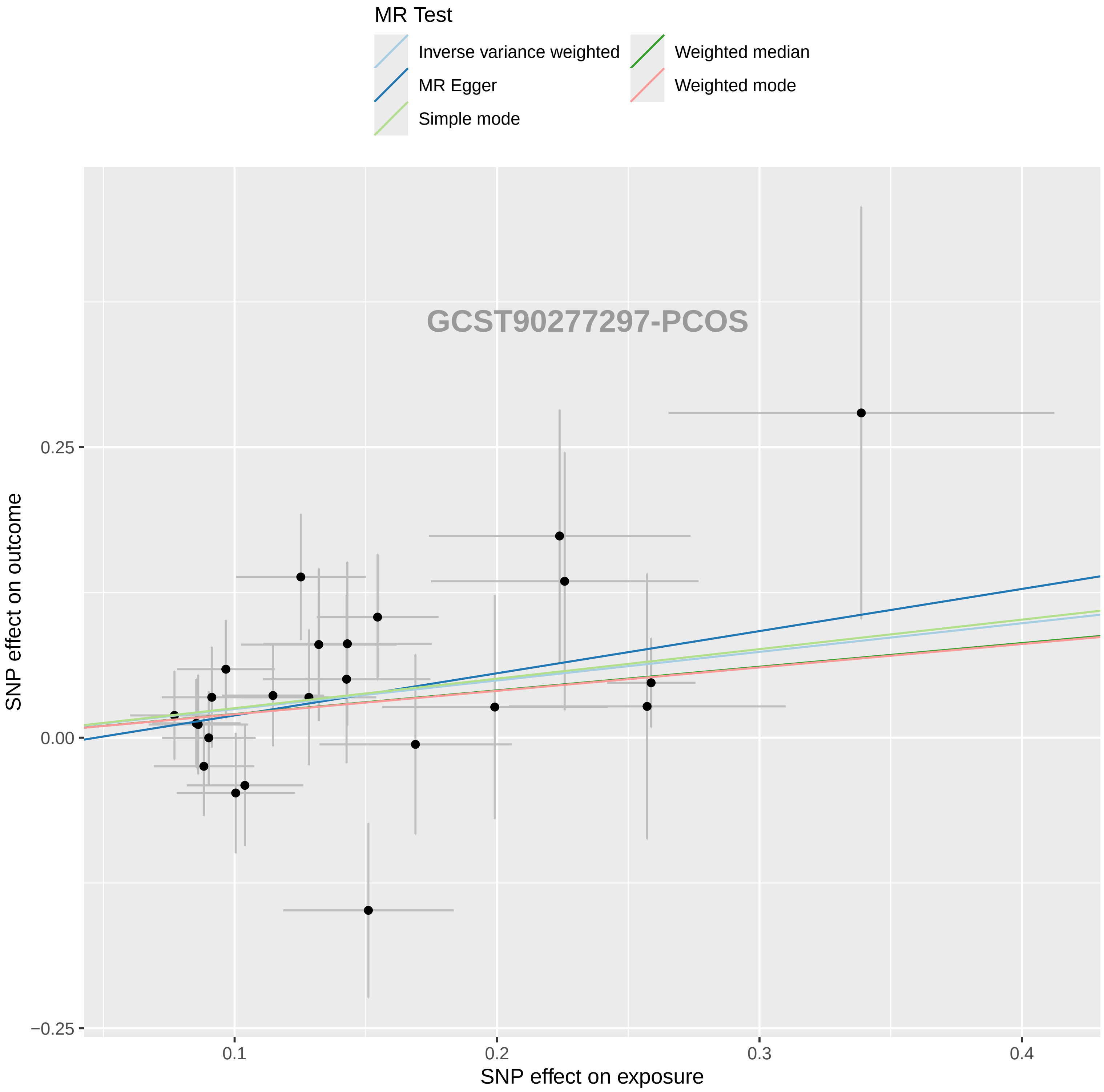
MR Test
Inverse variance weighted
MR Egger
Simple mode
Weighted median
Weighted mode
GCST90277297-PCOS
SNP effect on outcome
0.25
0.00
−0.25
SNP effect on exposure
0.1
0.2
0.3
0.4

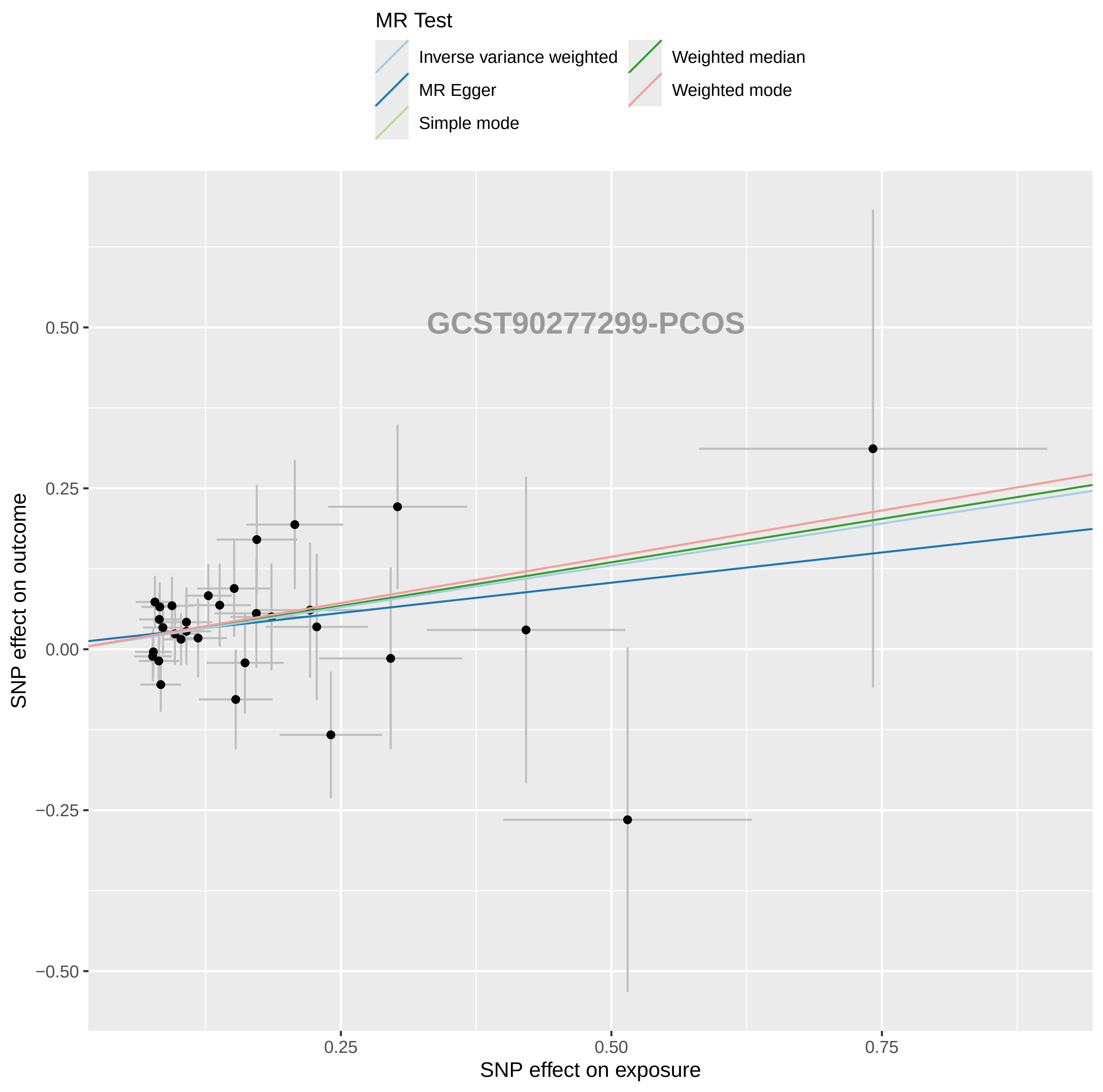
MR Test
Inverse variance weighted
MR Egger
Simple mode
Weighted median
Weighted mode
GCST90277299-PCOS
0.50
0.25
0.00
−0.25
−0.50
SNP effect on outcome
0.25
0.50
0.75
SNP effect on exposure

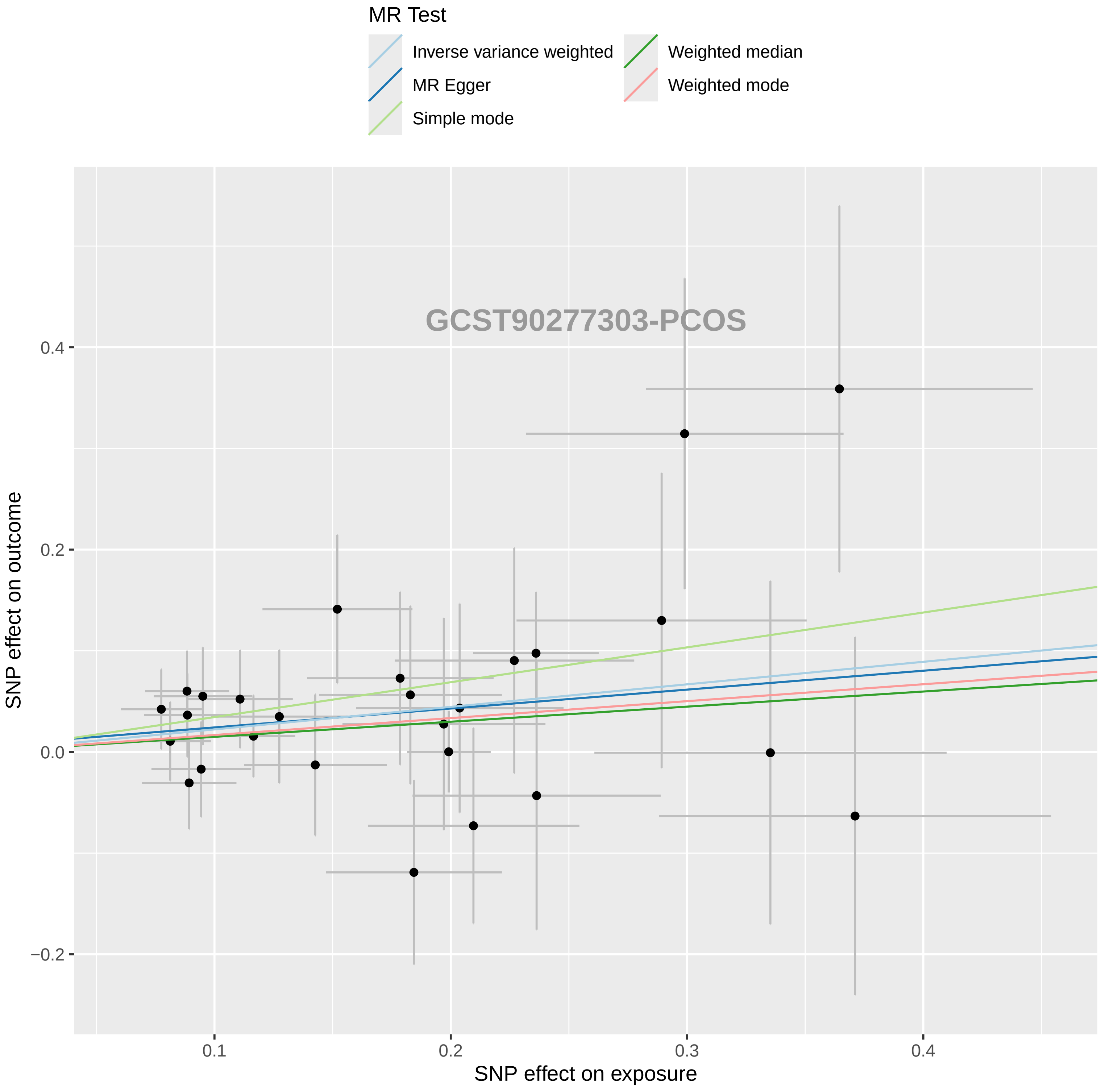
MR Test
Inverse variance weighted
MR Egger
Simple mode
Weighted median
Weighted mode
GCST90277303-PCOS
SNP effect on outcome
SNP effect on exposure
0.4
0.2
0.0
−0.2
0.1
0.2
0.3
0.4

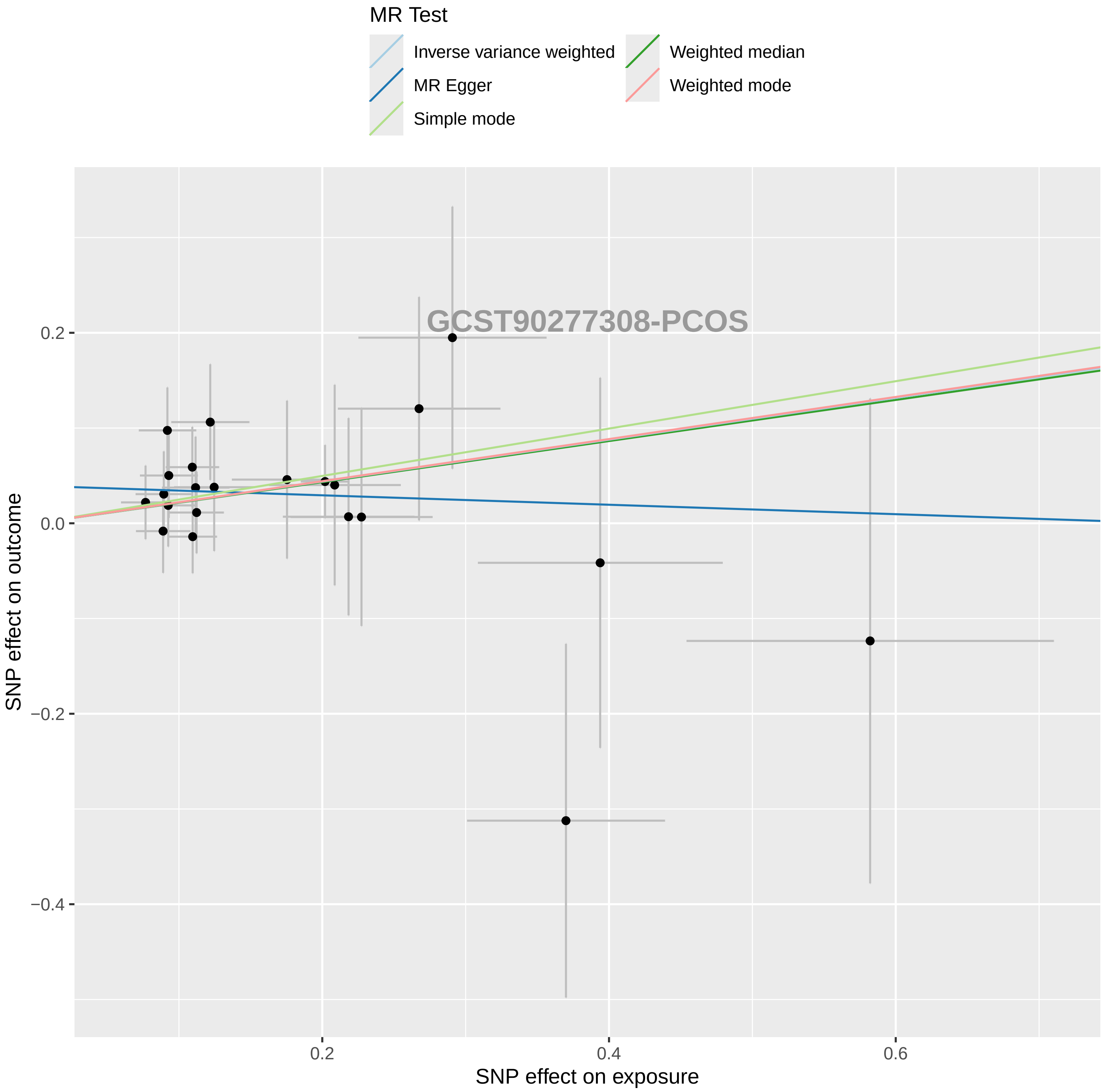
MR Test
Inverse variance weighted
MR Egger
Simple mode
Weighted median
Weighted mode
GCST90277308-PCOS
0.2
0.0
−0.2
−0.4
SNP effect on outcome
0.2
0.4
0.6
SNP effect on exposure

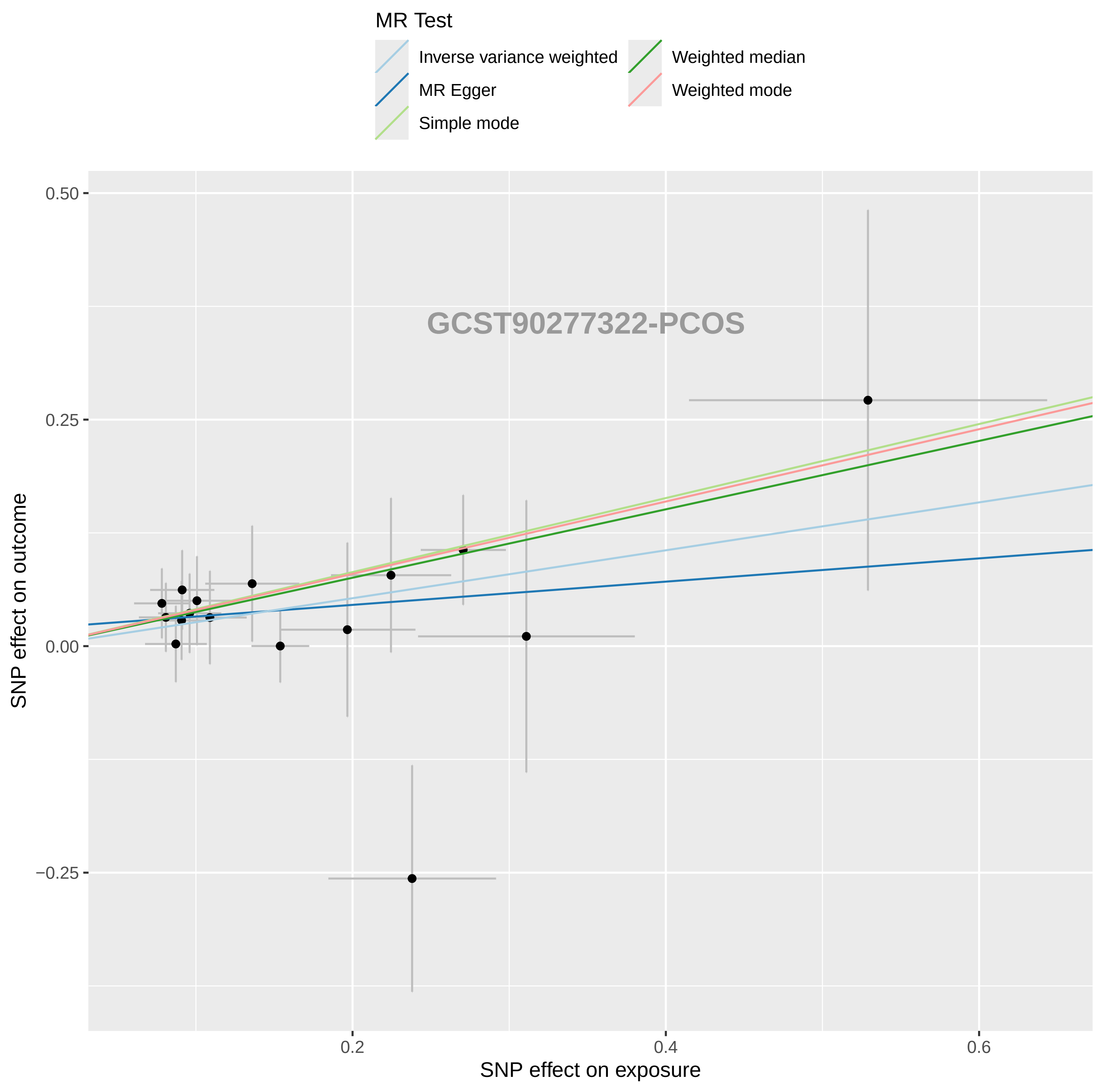
MR Test
Inverse variance weighted
MR Egger
Simple mode
Weighted median
Weighted mode
GCST90277322-PCOS
0.50
0.25
0.00
−0.25
SNP effect on outcome
0.2
0.4
0.6
SNP effect on exposure

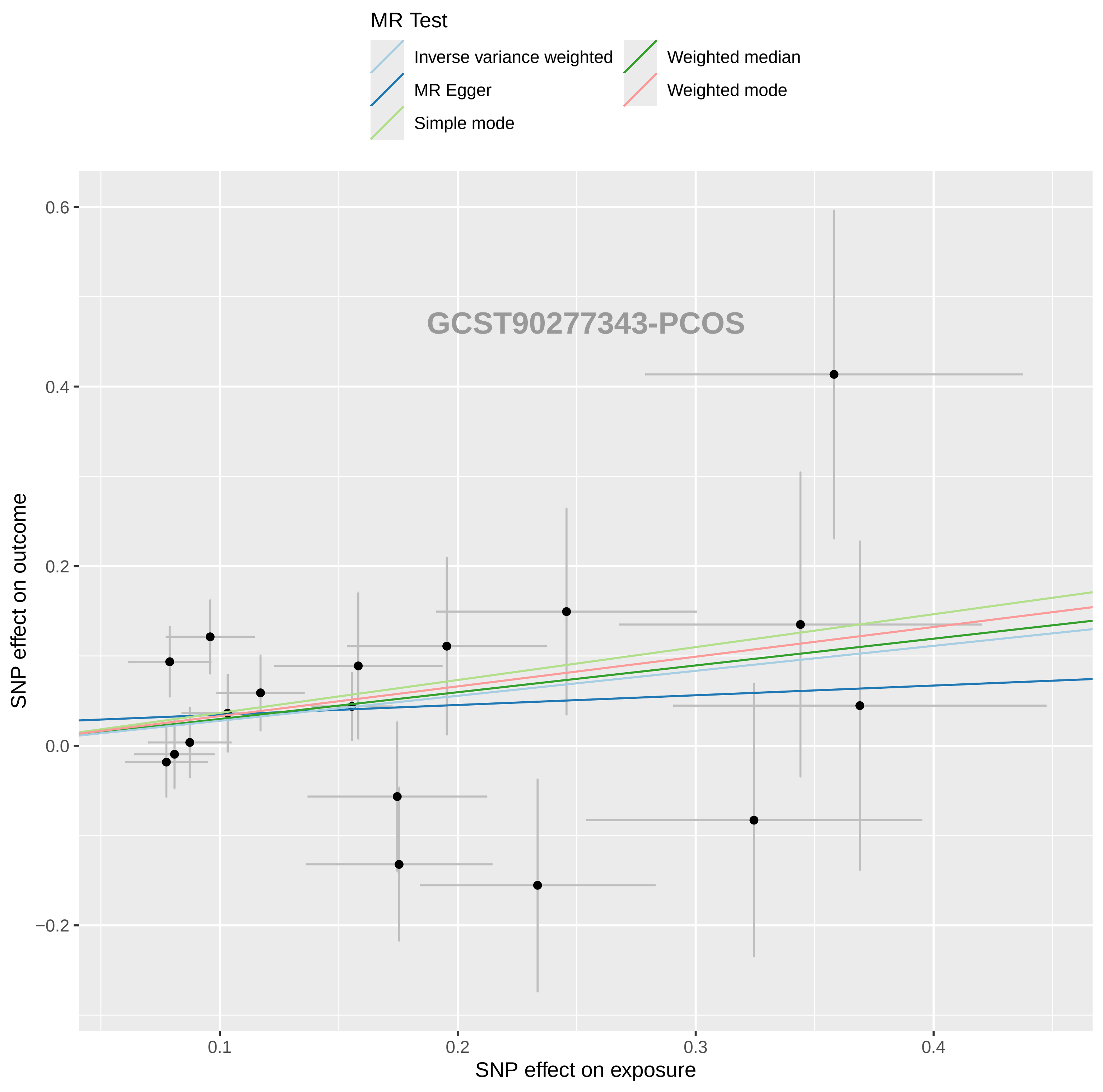

MR Test
Inverse variance weighted
MR Egger
Simple mode
Weighted median
Weighted mode
GCST90277343-PCOS
0.6
0.4
0.2
0.0
−0.2
0.1
0.2
0.3
0.4
SNP effect on outcome
SNP effect on exposure

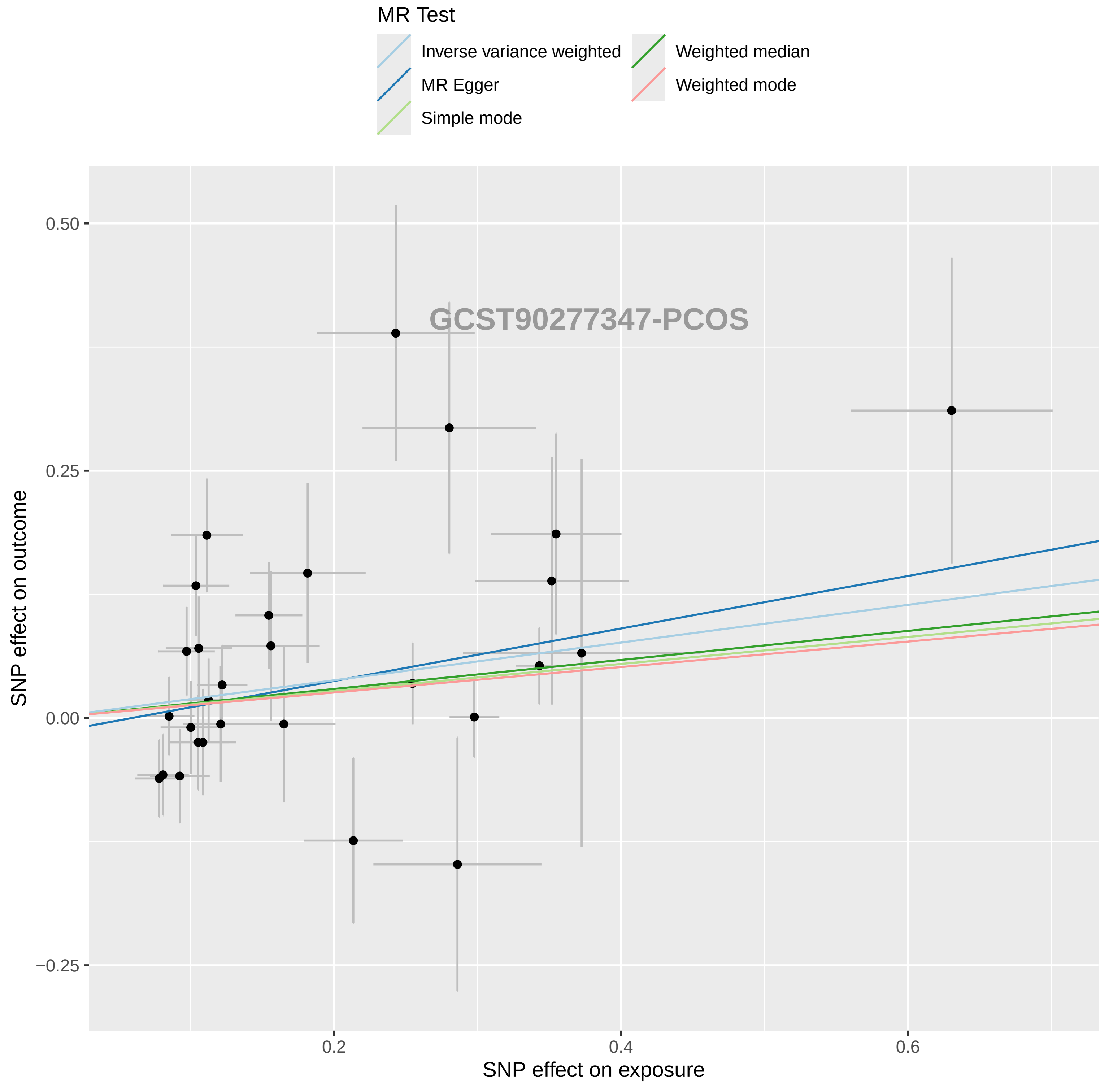
MR Test
Inverse variance weighted
MR Egger
Simple mode
Weighted median
Weighted mode
GCST90277347-PCOS
SNP effect on outcome
0.50
0.25
0.00
−0.25
0.2
0.4
0.6
SNP effect on exposure

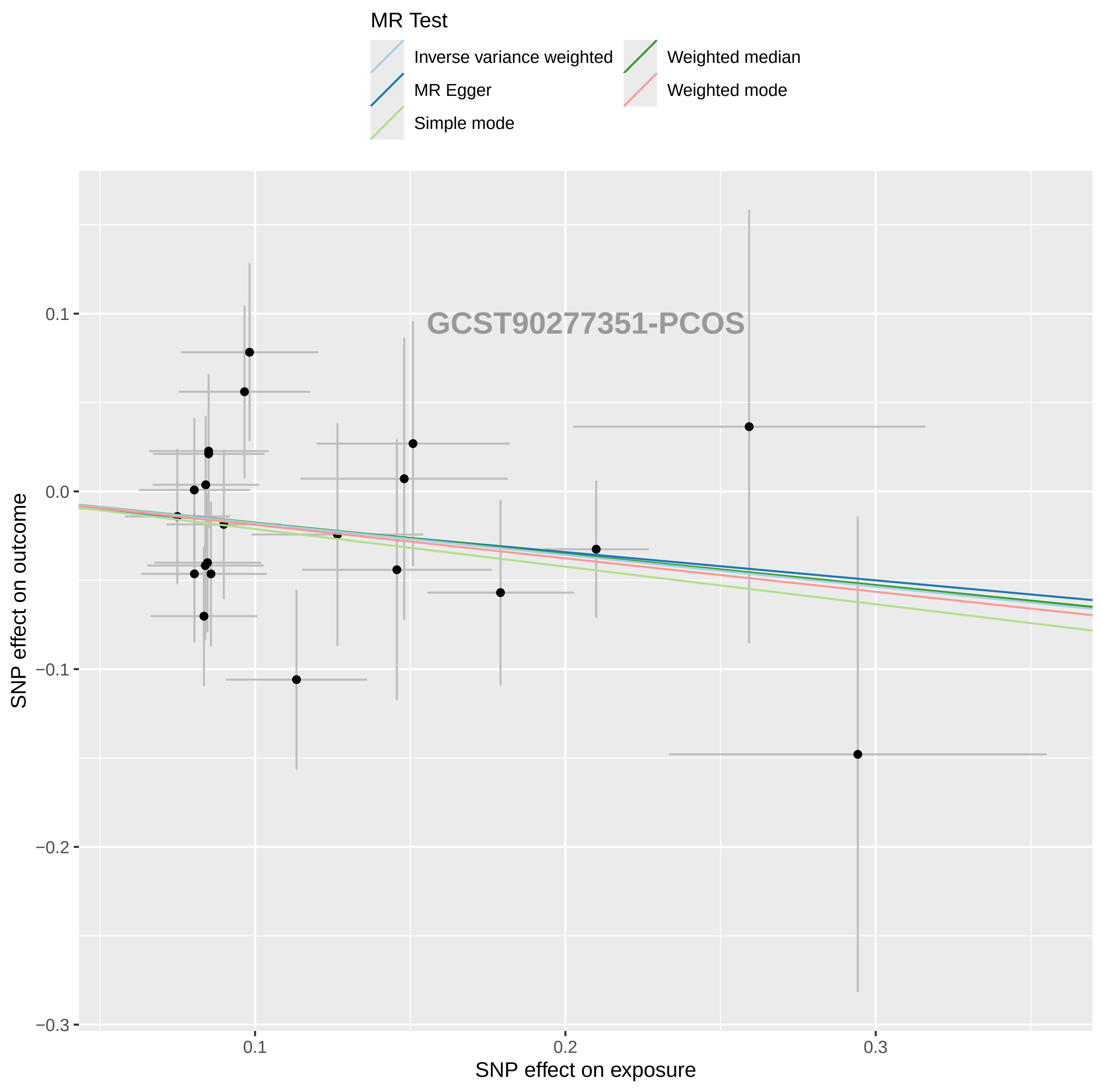

MR Test
Inverse variance weighted
MR Egger
Simple mode
Weighted median
Weighted mode
GCST90277351-PCOS
SNP effect on outcome
SNP effect on exposure
0.1
0.0
−0.1
−0.2
−0.3
0.1
0.2
0.3

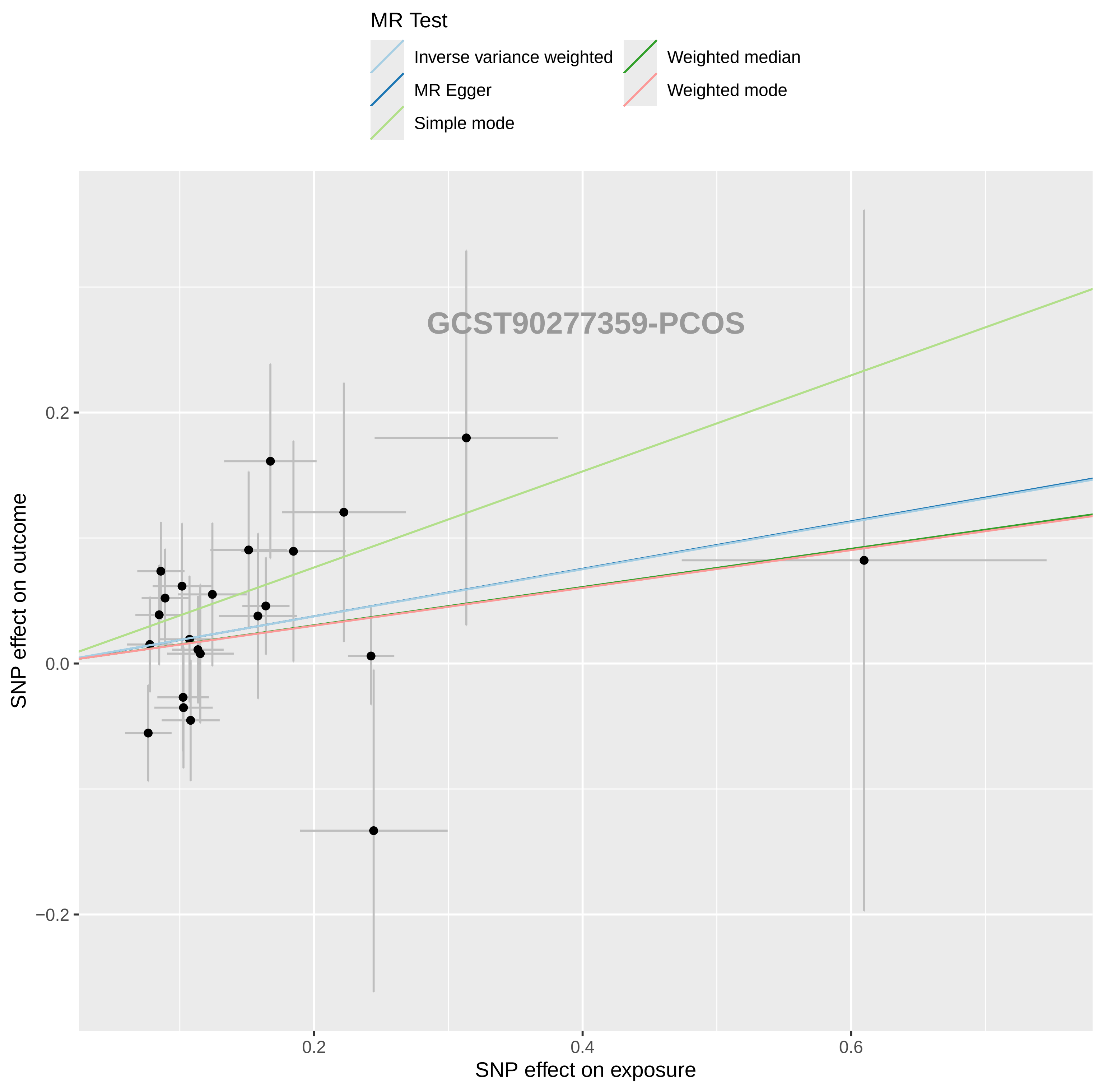
MR Test
Inverse variance weighted
MR Egger
Simple mode
Weighted median
Weighted mode
GCST90277359-PCOS
0.2
0.0
−0.2
SNP effect on outcome
0.2
0.4
0.6
SNP effect on exposure

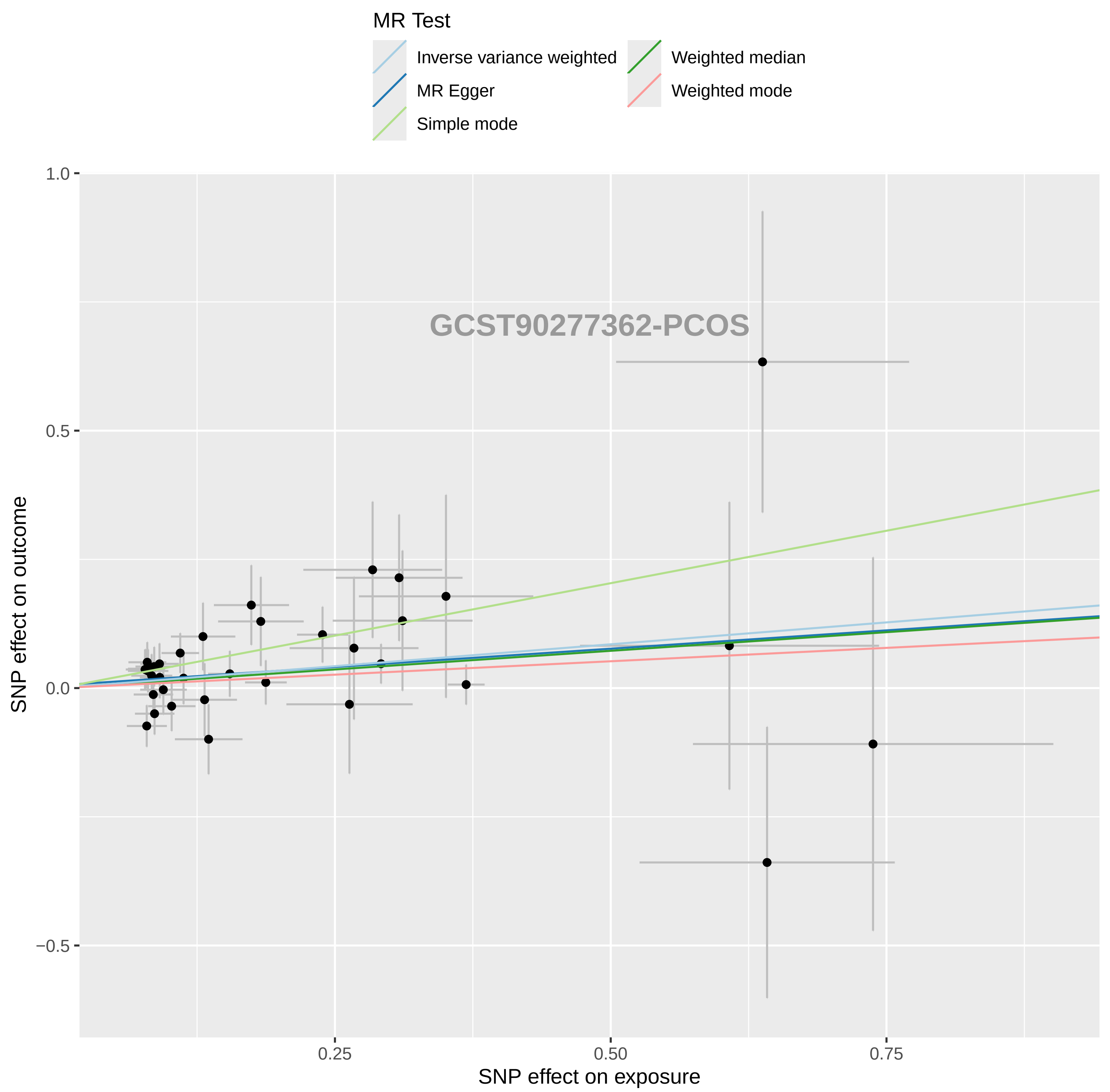
MR Test
Inverse variance weighted
MR Egger
Simple mode
Weighted median
Weighted mode
GCST90277362-PCOS
1.0
0.5
0.0
−0.5
0.25
0.50
0.75
SNP effect on outcome
SNP effect on exposure

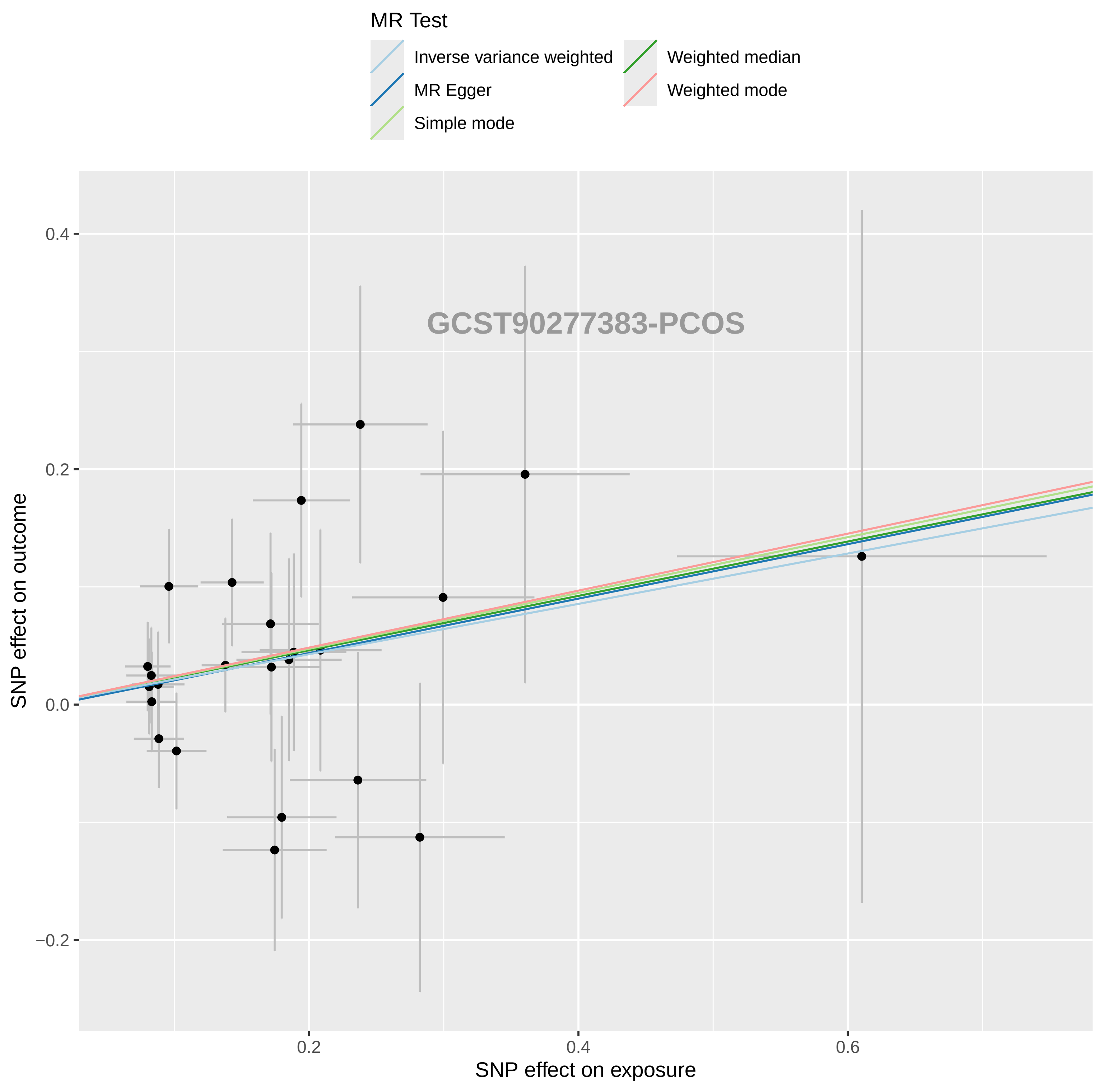
MR Test
Inverse variance weighted
MR Egger
Simple mode
Weighted median
Weighted mode
GCST90277383-PCOS
SNP effect on outcome
SNP effect on exposure
0.4
0.2
0.0
−0.2
0.2
0.4
0.6

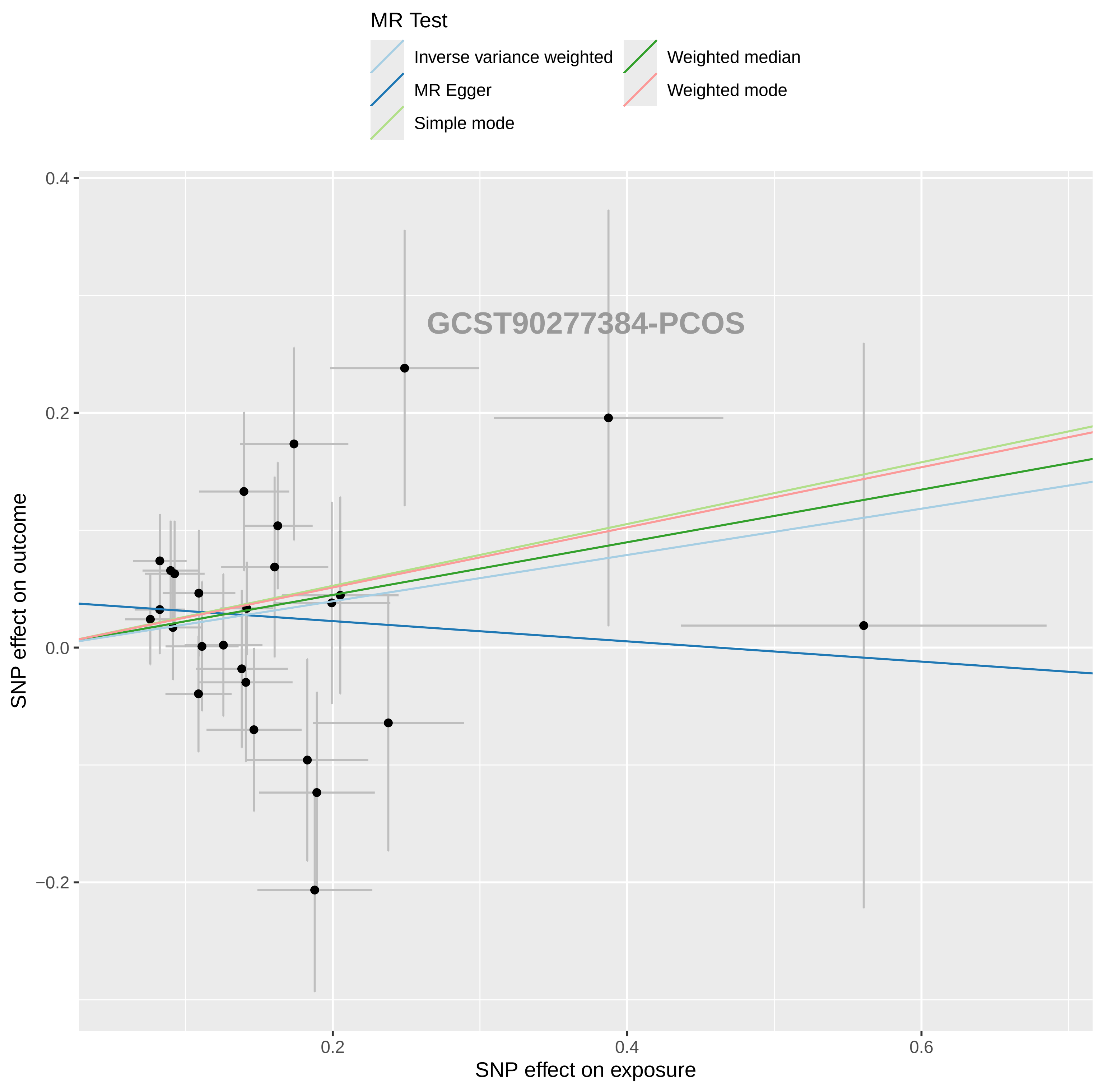
MR Test
Inverse variance weighted
MR Egger
Simple mode
Weighted median
Weighted mode
GCST90277384-PCOS
0.4
0.2
0.0
−0.2
SNP effect on outcome
0.2
0.4
0.6
SNP effect on exposure

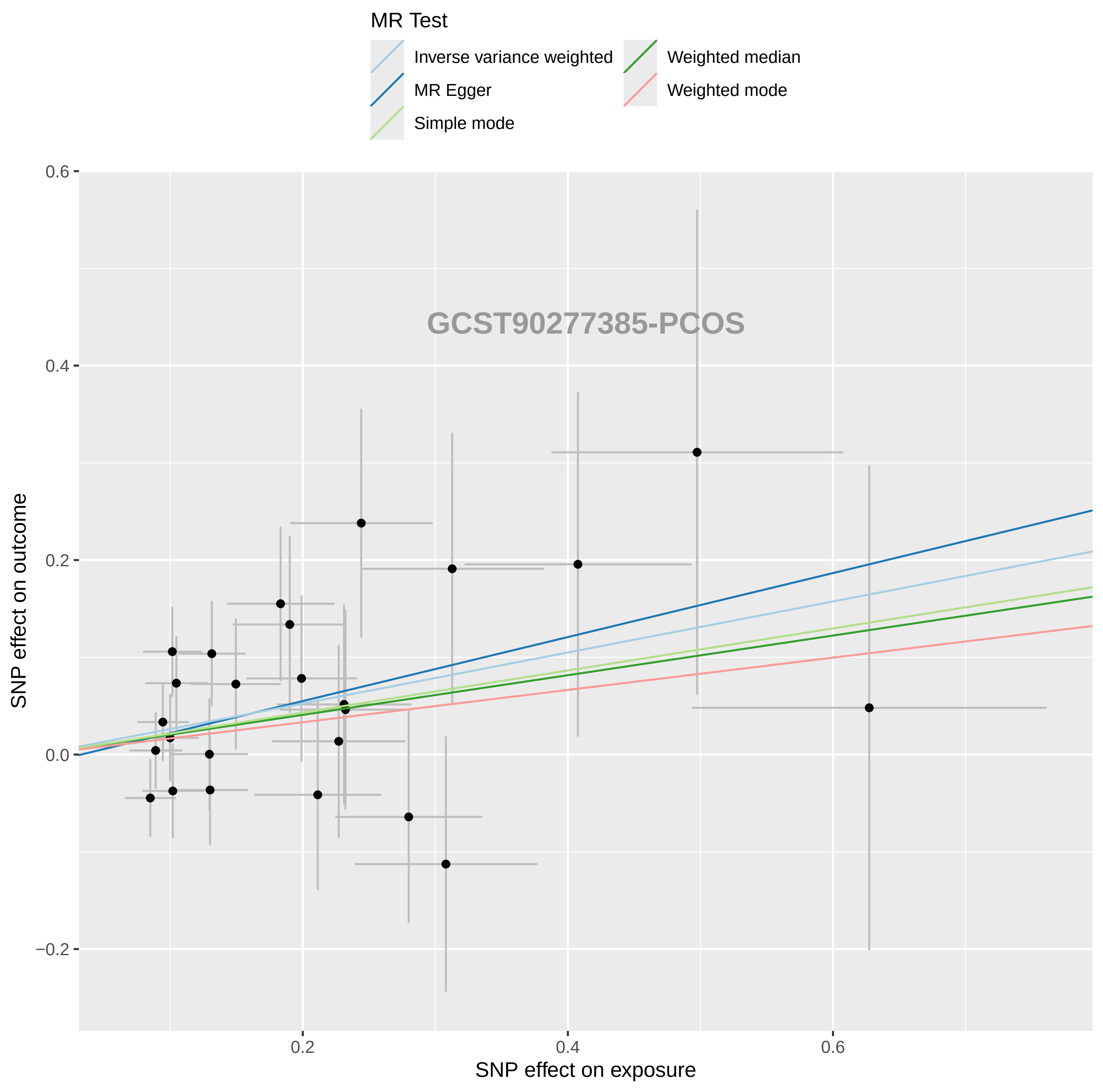
MR Test
Inverse variance weighted
MR Egger
Simple mode
Weighted median
Weighted mode
GCST90277385-PCOS
0.6
0.4
0.2
0.0
−0.2
0.2
0.4
0.6
SNP effect on outcome
SNP effect on exposure

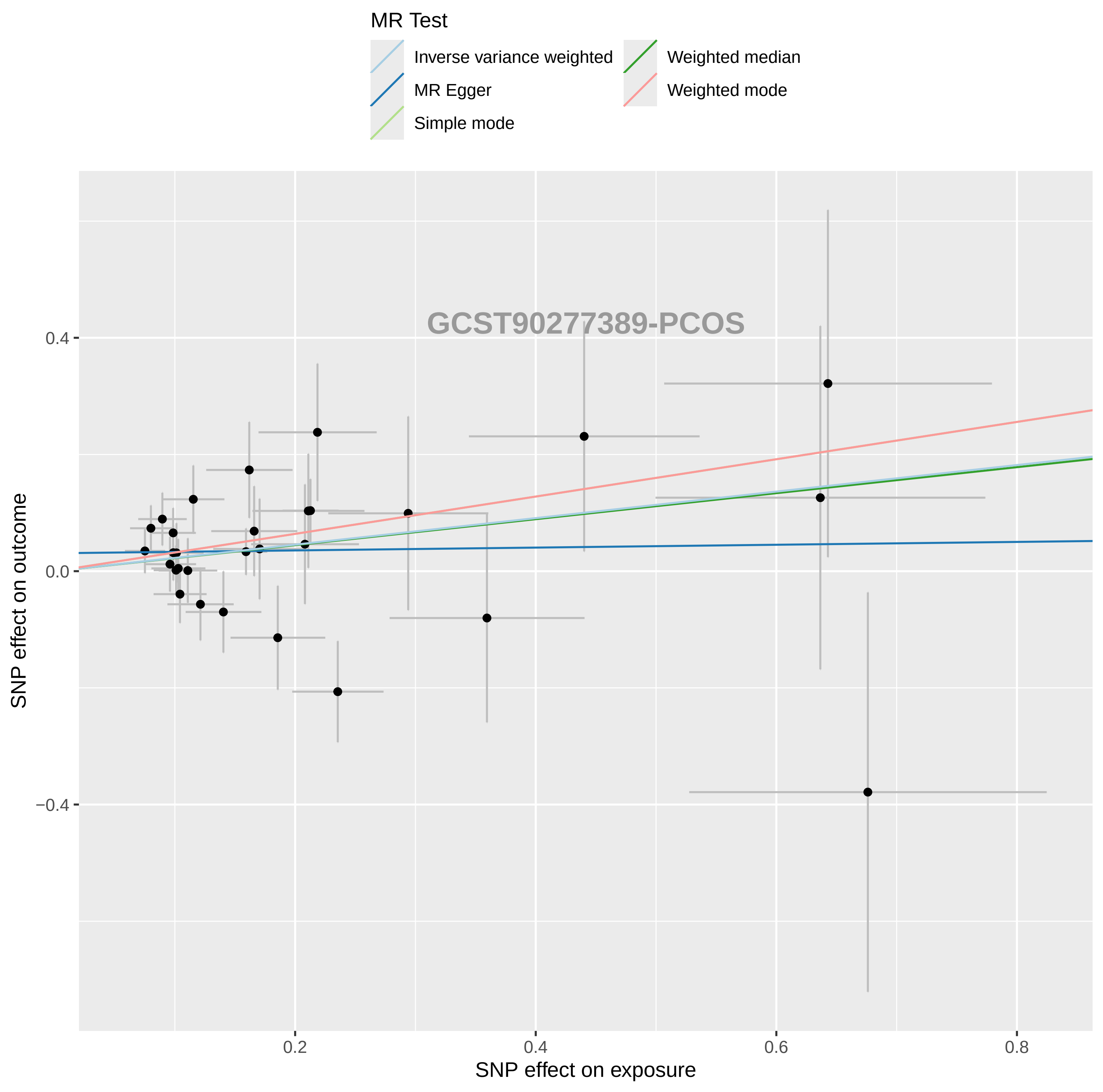
MR Test
Inverse variance weighted
MR Egger
Simple mode
Weighted median
Weighted mode
GCST90277389-PCOS
0.4
0.0
−0.4
0.2
0.4
0.6
0.8
SNP effect on outcome
SNP effect on exposure

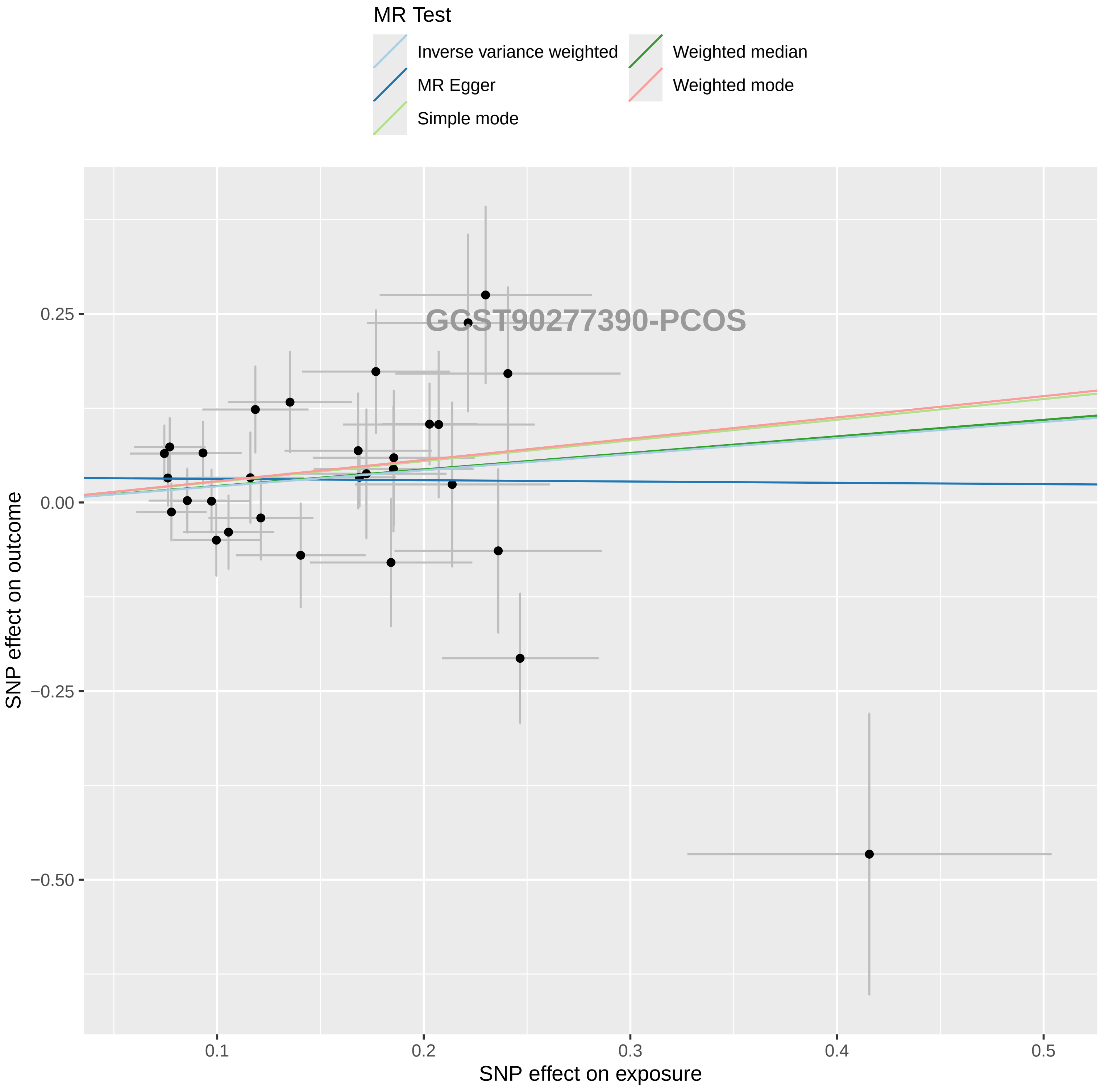
MR Test
Inverse variance weighted
MR Egger
Simple mode
Weighted median
Weighted mode
GCST90277390-PCOS
0.25
0.00
−0.25
−0.50
SNP effect on outcome
0.1
0.2
0.3
0.4
0.5
SNP effect on exposure

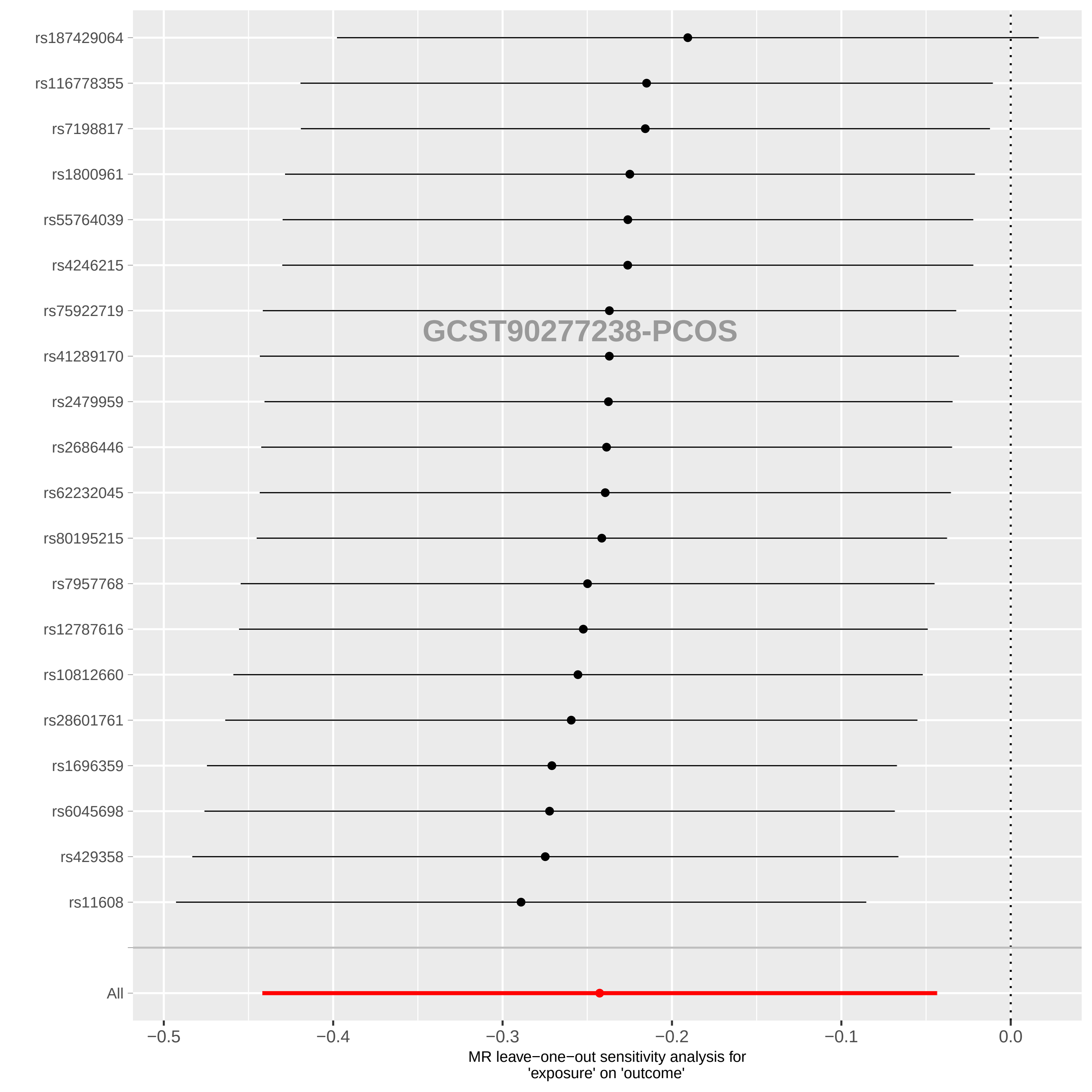
GCST90277238-PCOS
rs187429064
rs116778355
rs7198817
rs1800961
rs55764039
rs4246215
rs75922719
rs41289170
rs2479959
rs2686446
rs62232045
rs80195215
rs7957768
rs12787616
rs10812660
rs28601761
rs1696359
rs6045698
rs429358
rs11608
All
−0.5
−0.4
−0.3
−0.2
−0.1
0.0
MR leave−one−out sensitivity analysis for
'exposure' on 'outcome'

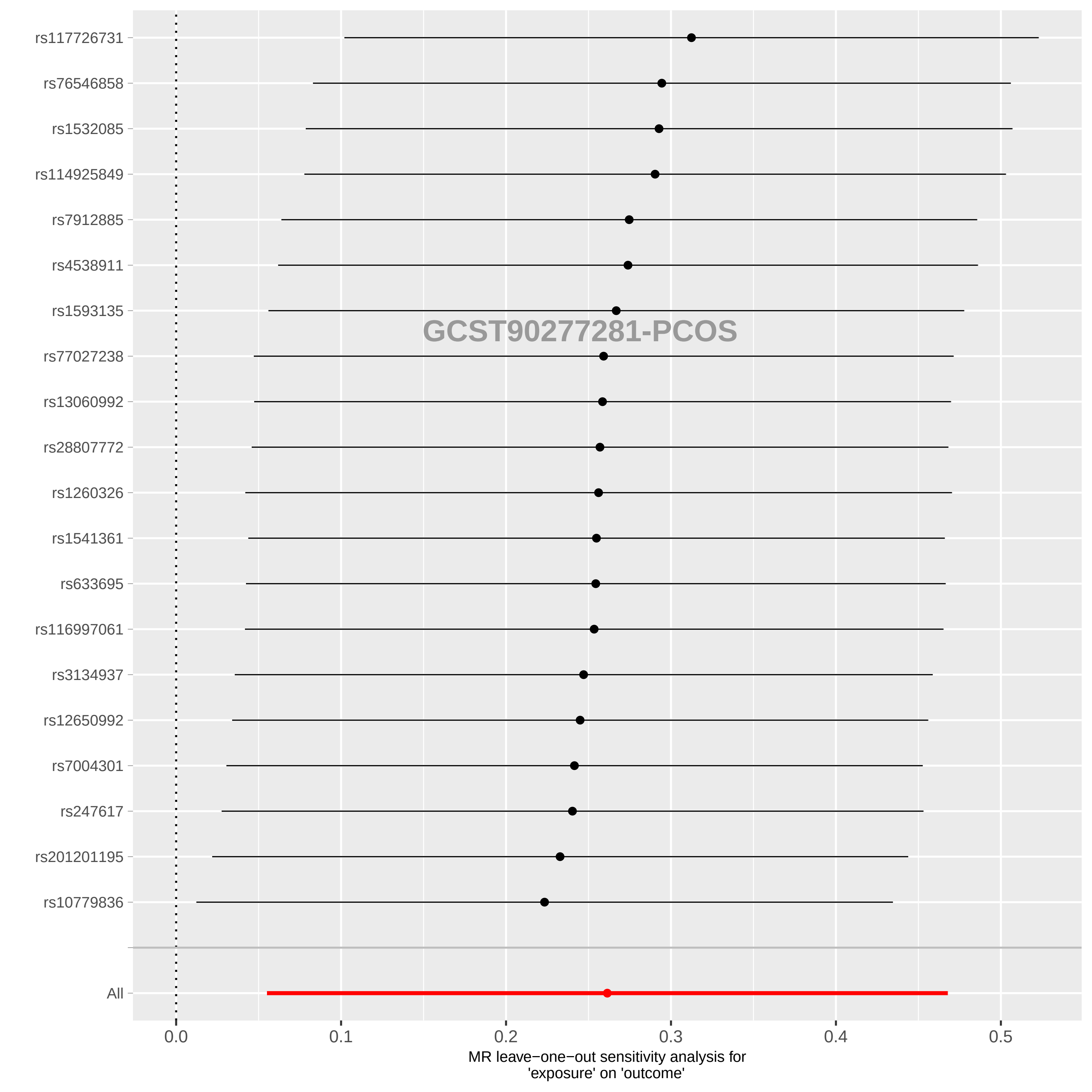

GCST90277281-PCOS
rs117726731
rs76546858
rs1532085
rs114925849
rs7912885
rs4538911
rs1593135
rs77027238
rs13060992
rs28807772
rs1260326
rs1541361
rs633695
rs116997061
rs3134937
rs12650992
rs7004301
rs247617
rs201201195
rs10779836
All
0.0
0.1
0.2
0.3
0.4
0.5
MR leave−one−out sensitivity analysis for
'exposure' on 'outcome'

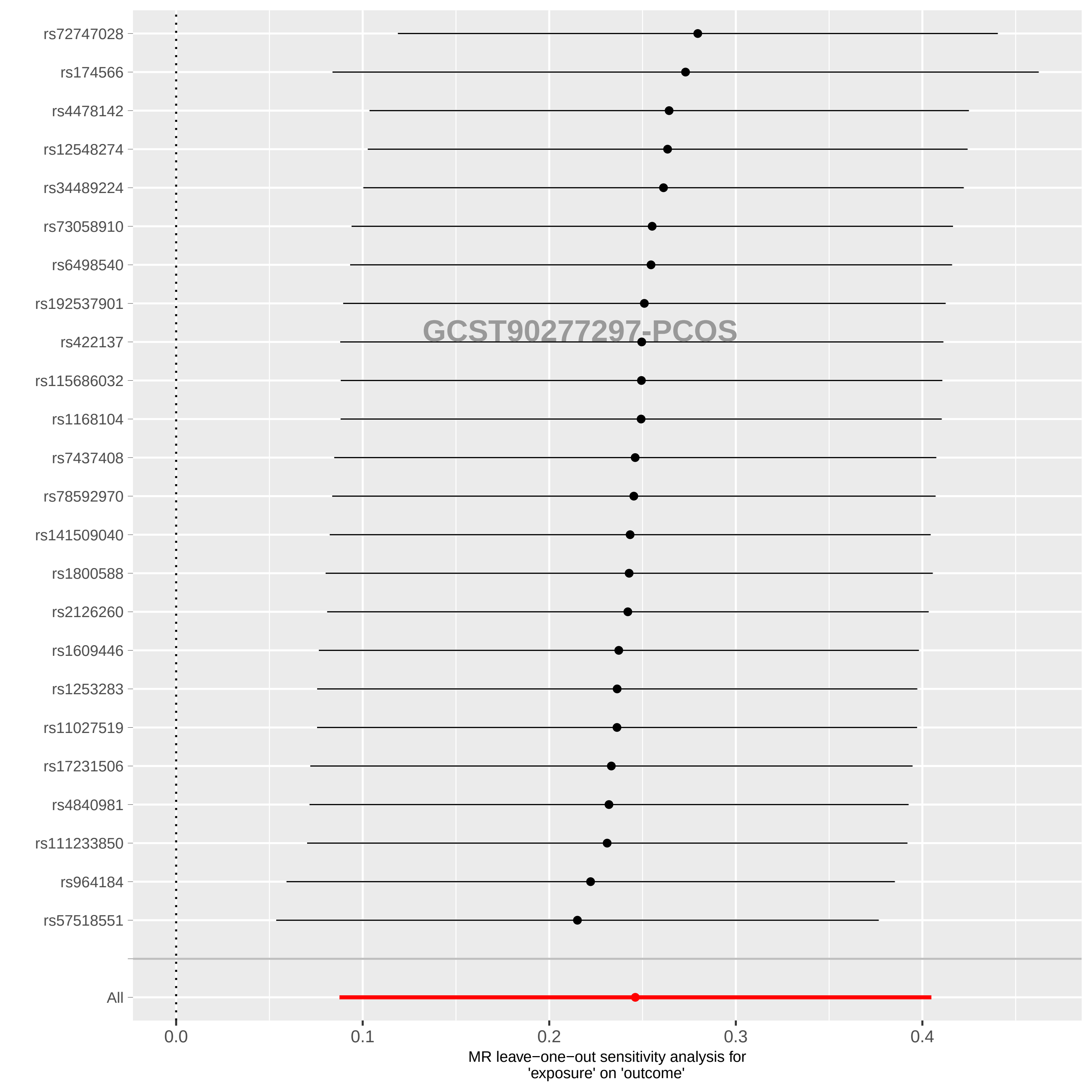

GCST90277297-PCOS
rs72747028
rs174566
rs4478142
rs12548274
rs34489224
rs73058910
rs6498540
rs192537901
rs422137
rs115686032
rs1168104
rs7437408
rs78592970
rs141509040
rs1800588
rs2126260
rs1609446
rs1253283
rs11027519
rs17231506
rs4840981
rs111233850
rs964184
rs57518551
All
0.0
0.1
0.2
0.3
0.4
MR leave−one−out sensitivity analysis for
'exposure' on 'outcome'

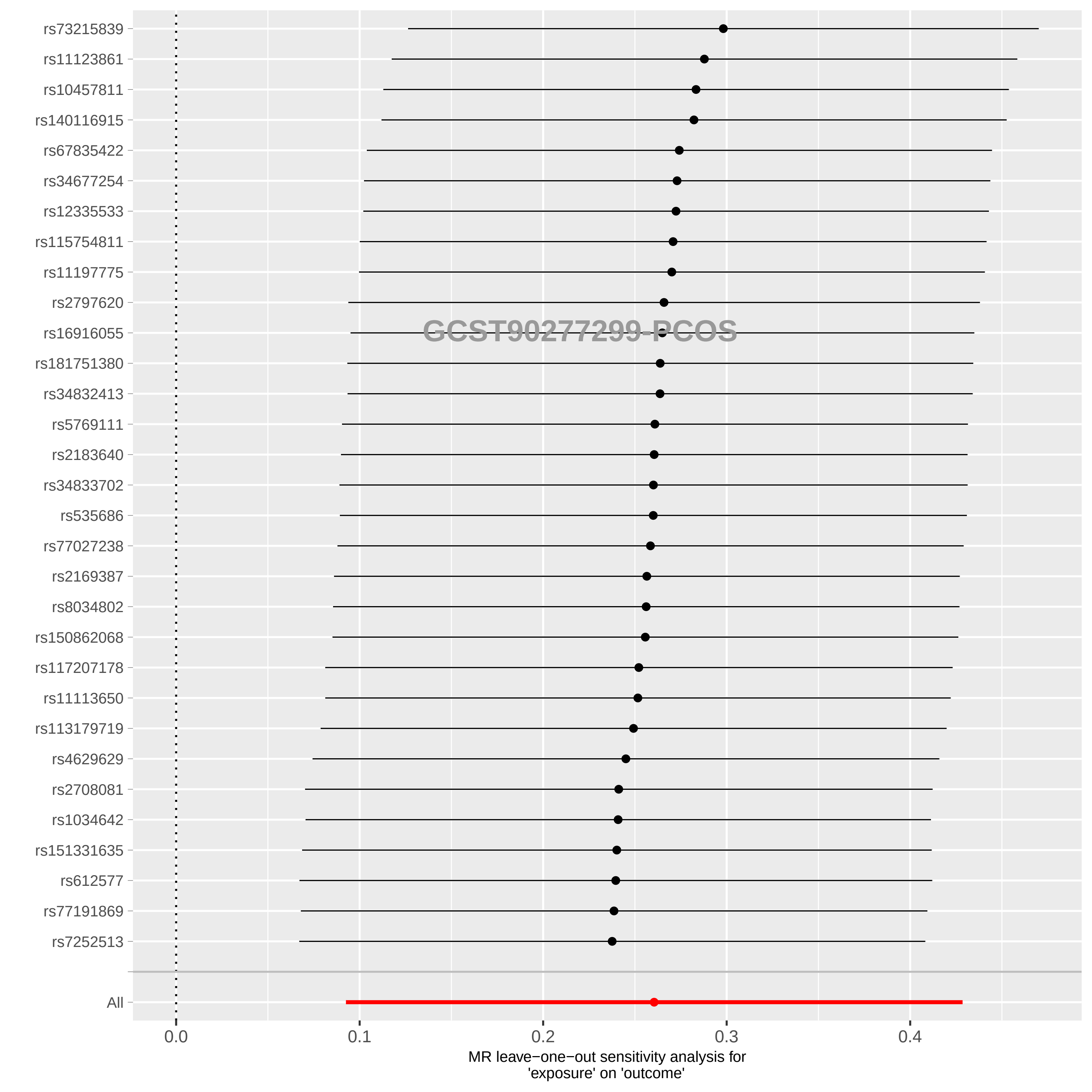
GCST90277299-PCOS
rs73215839
rs11123861
rs10457811
rs140116915
rs67835422
rs34677254
rs12335533
rs115754811
rs11197775
rs2797620
rs16916055
rs181751380
rs34832413
rs5769111
rs2183640
rs34833702
rs535686
rs77027238
rs2169387
rs8034802
rs150862068
rs117207178
rs11113650
rs113179719
rs4629629
rs2708081
rs1034642
rs151331635
rs612577
rs77191869
rs7252513
All
0.0
0.1
0.2
0.3
0.4
MR leave−one−out sensitivity analysis for
'exposure' on 'outcome'

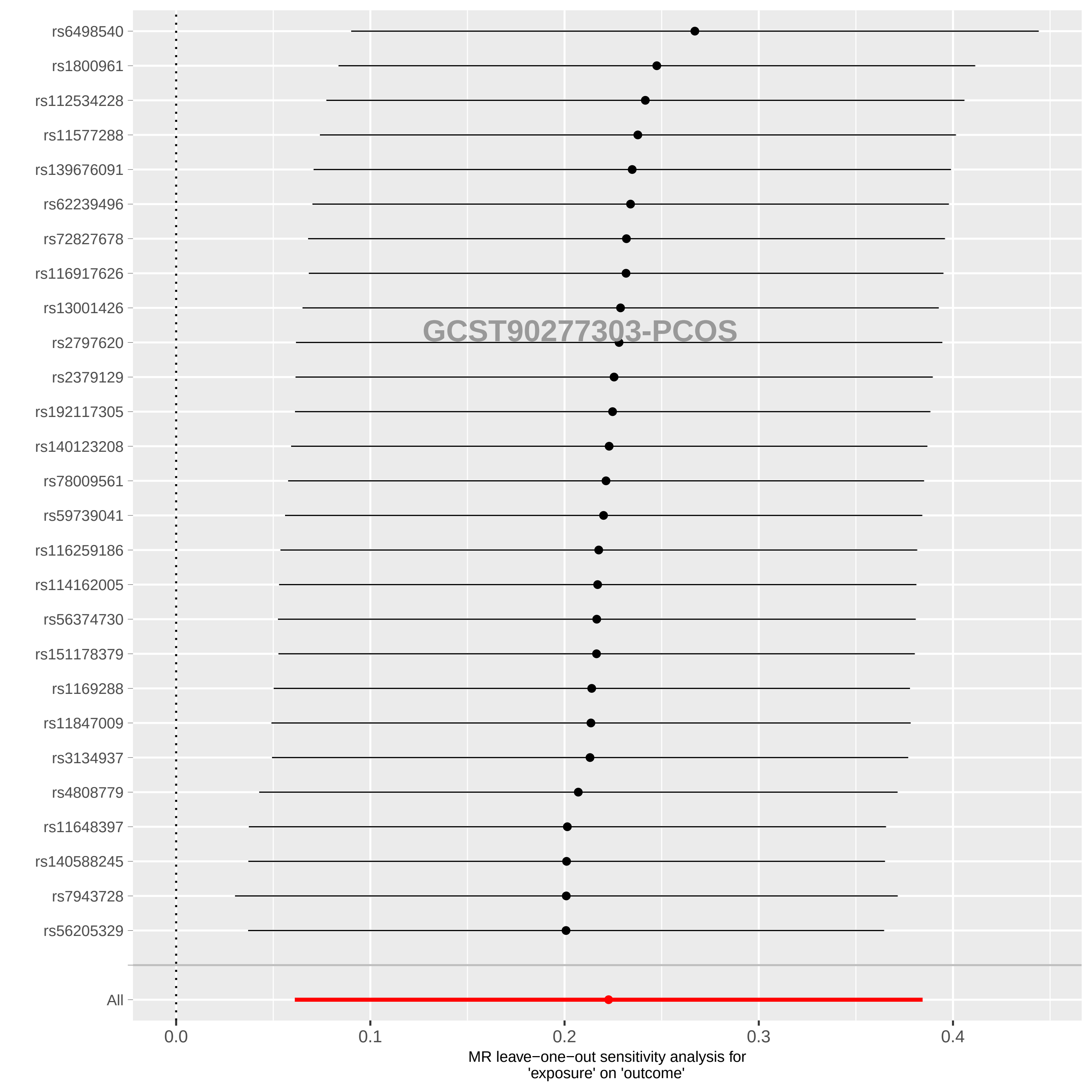

GCST90277303-PCOS
rs6498540
rs1800961
rs112534228
rs11577288
rs139676091
rs62239496
rs72827678
rs116917626
rs13001426
rs2797620
rs2379129
rs192117305
rs140123208
rs78009561
rs59739041
rs116259186
rs114162005
rs56374730
rs151178379
rs1169288
rs11847009
rs3134937
rs4808779
rs11648397
rs140588245
rs7943728
rs56205329
All
0.0
0.1
0.2
0.3
0.4
MR leave−one−out sensitivity analysis for
'exposure' on 'outcome'

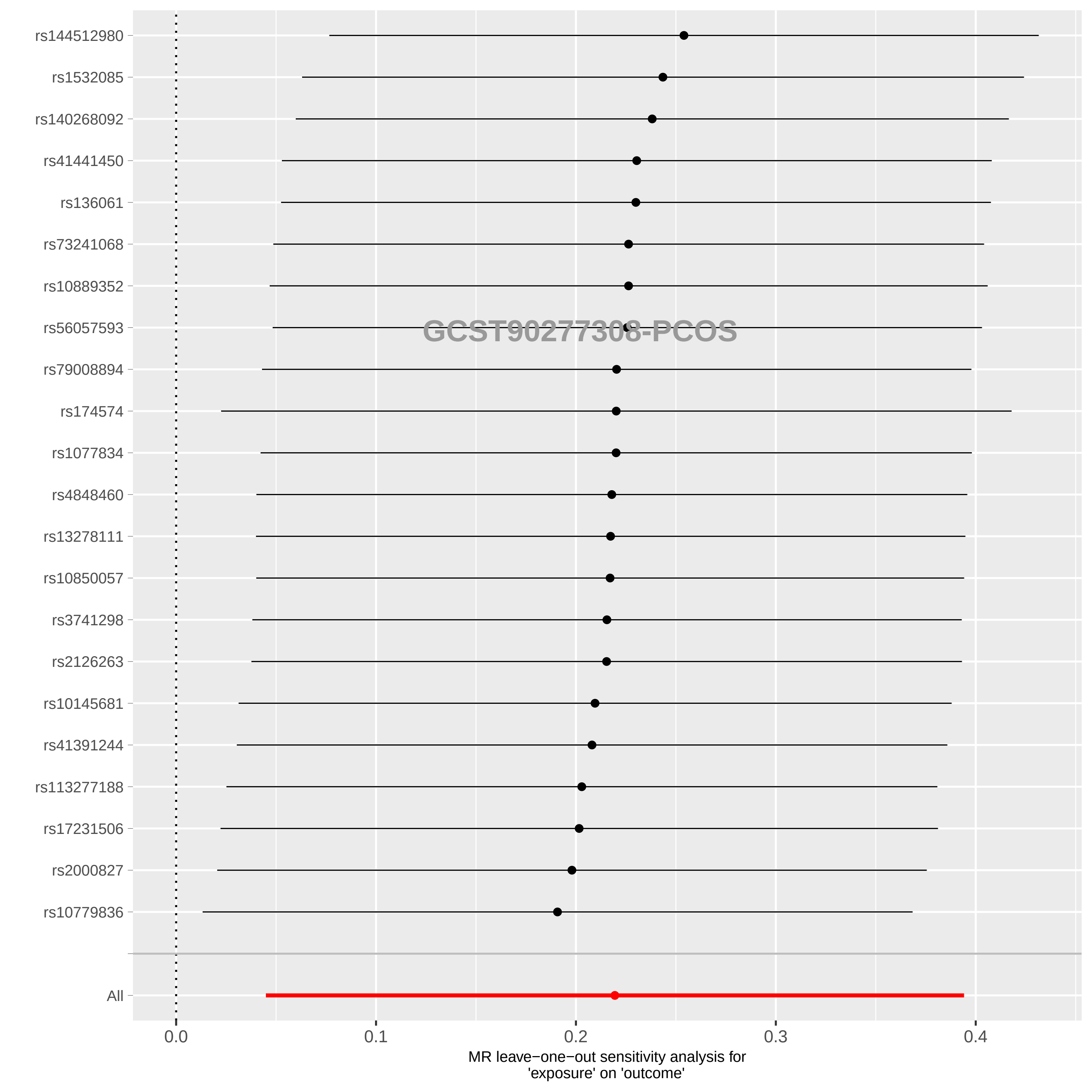

rs144512980
rs1532085
rs140268092
rs41441450
rs136061
rs73241068
rs10889352
rs56057593
rs79008894
rs174574
rs1077834
rs4848460
rs13278111
rs10850057
rs3741298
rs2126263
rs10145681
rs41391244
rs113277188
rs17231506
rs2000827
rs10779836
All
GCST90277308-PCOS
0.0
0.1
0.2
0.3
0.4
MR leave−one−out sensitivity analysis for
'exposure' on 'outcome'

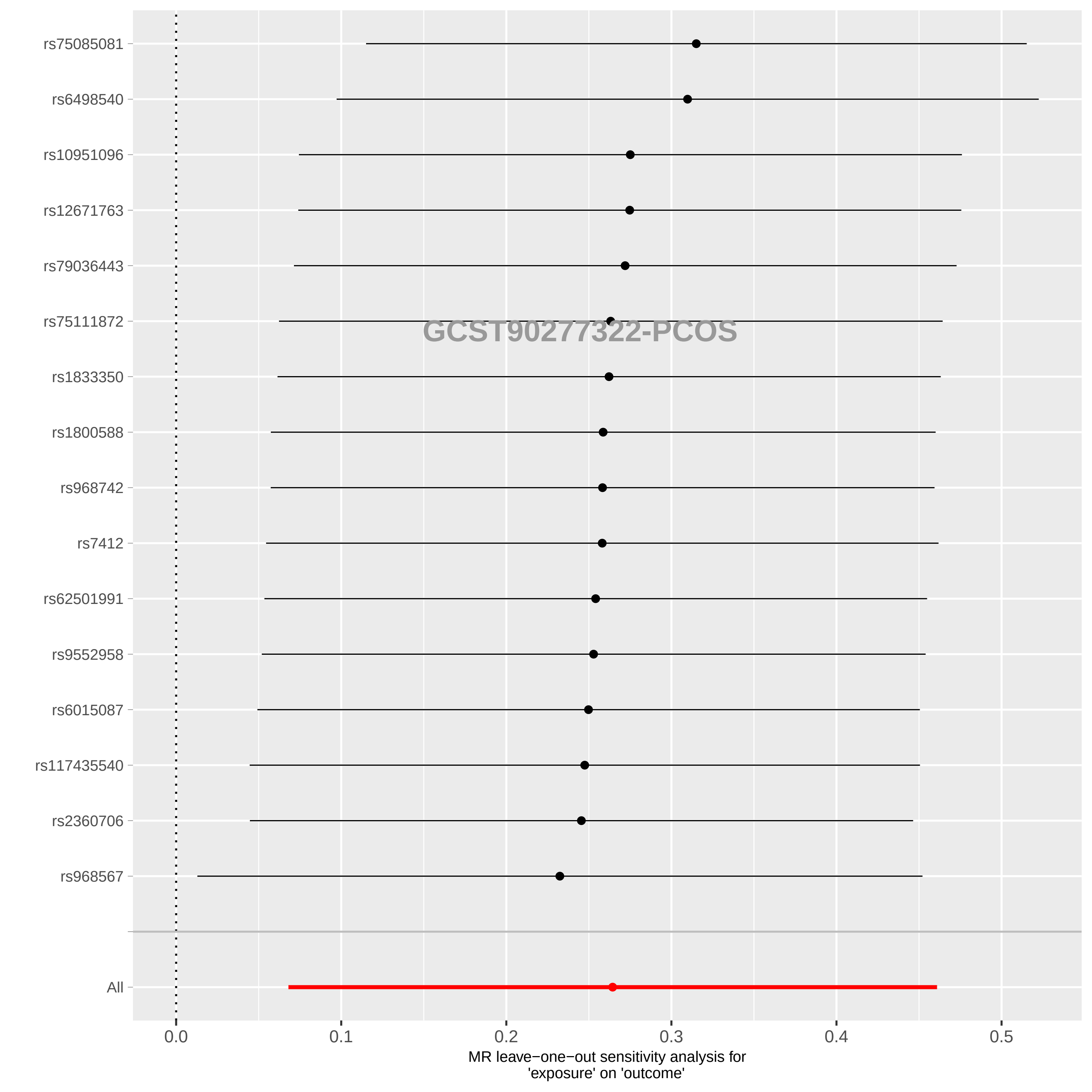
rs75085081
rs6498540
rs10951096
rs12671763
rs79036443
rs75111872
GCST90277322-PCOS
rs1833350
rs1800588
rs968742
rs7412
rs62501991
rs9552958
rs6015087
rs117435540
rs2360706
rs968567
All
0.0
0.1
0.2
0.3
0.4
0.5
MR leave−one−out sensitivity analysis for
'exposure' on 'outcome'

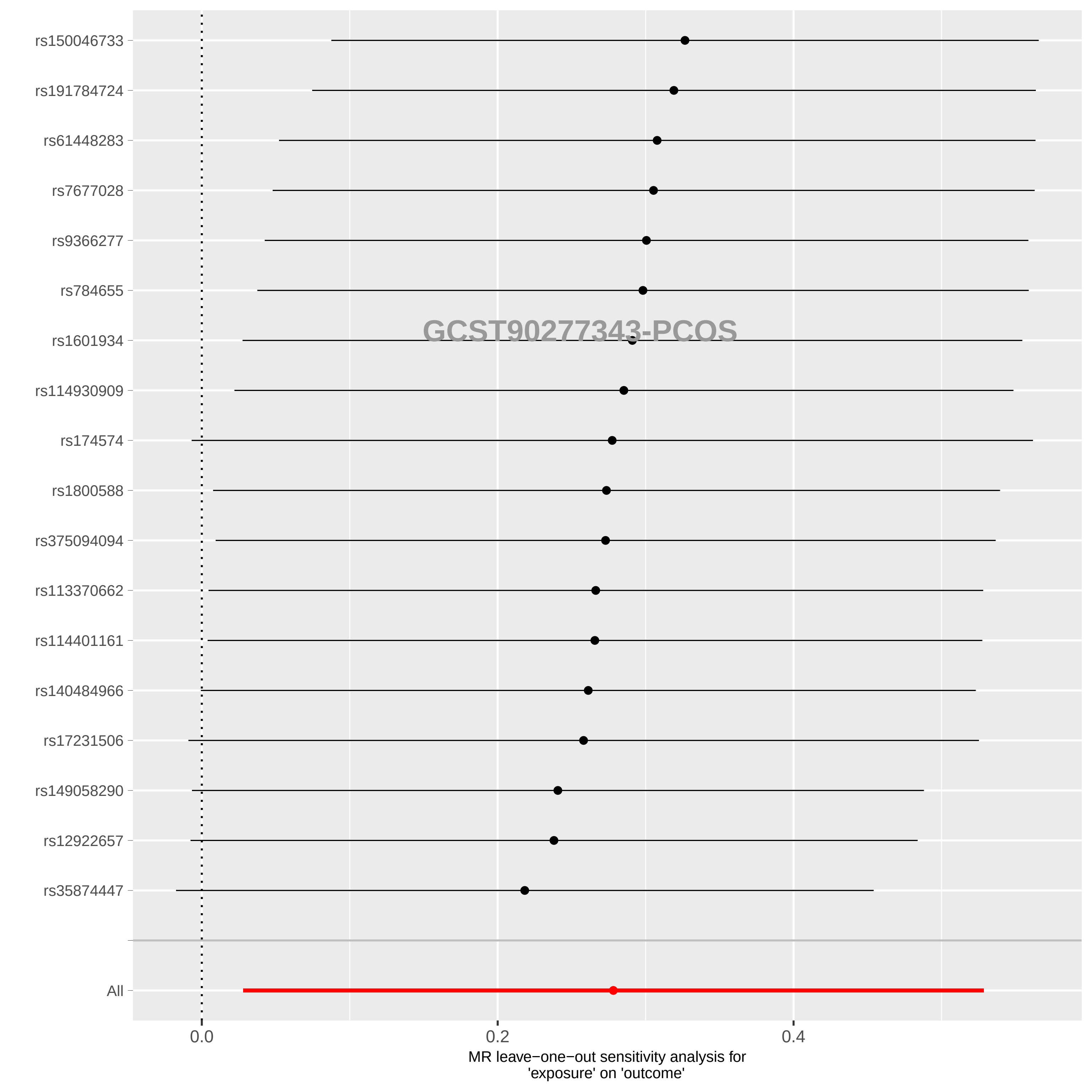
GCST90277343-PCOS
rs150046733
rs191784724
rs61448283
rs7677028
rs9366277
rs784655
rs1601934
rs114930909
rs174574
rs1800588
rs375094094
rs113370662
rs114401161
rs140484966
rs17231506
rs149058290
rs12922657
rs35874447
All
0.0
0.2
0.4
MR leave−one−out sensitivity analysis for
'exposure' on 'outcome'

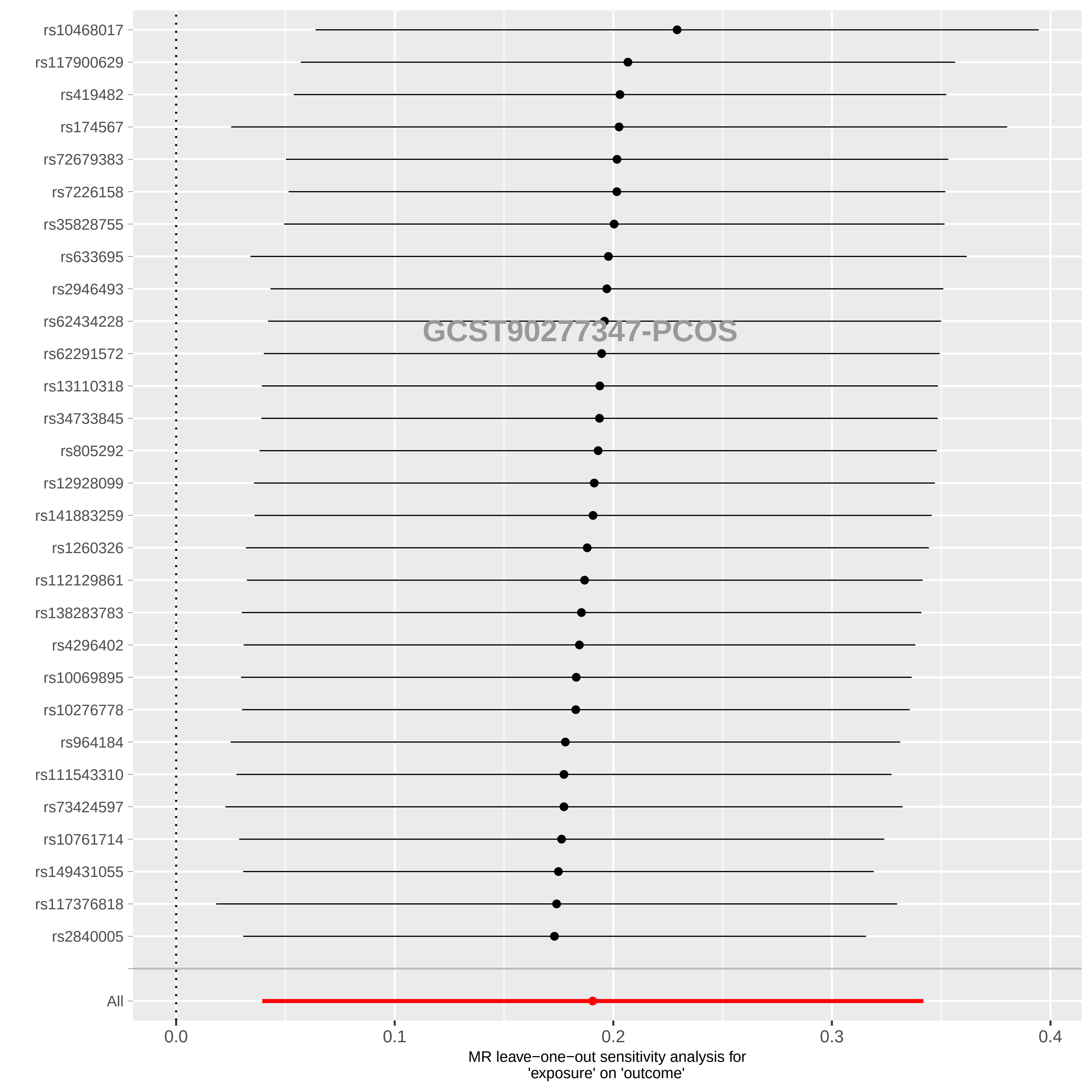
GCST90277347-PCOS
rs10468017
rs117900629
rs419482
rs174567
rs72679383
rs7226158
rs35828755
rs633695
rs2946493
rs62434228
rs62291572
rs13110318
rs34733845
rs805292
rs12928099
rs141883259
rs1260326
rs112129861
rs138283783
rs4296402
rs10069895
rs10276778
rs964184
rs111543310
rs73424597
rs10761714
rs149431055
rs117376818
rs2840005
All
0.0
0.1
0.2
0.3
0.4
MR leave−one−out sensitivity analysis for
'exposure' on 'outcome'

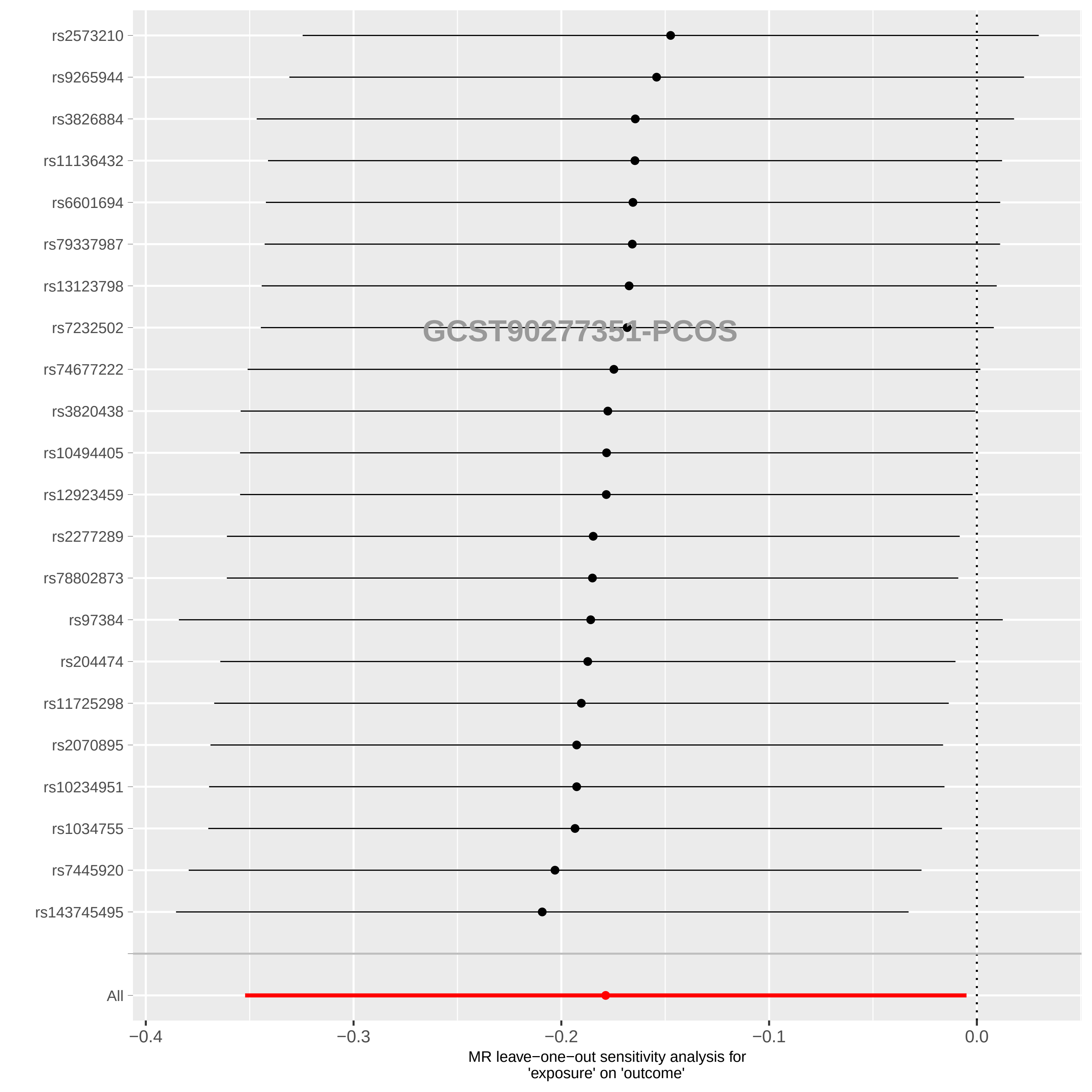
rs2573210
rs9265944
rs3826884
rs11136432
rs6601694
rs79337987
rs13123798
rs7232502
rs74677222
rs3820438
rs10494405
rs12923459
rs2277289
rs78802873
rs97384
rs204474
rs11725298
rs2070895
rs10234951
rs1034755
rs7445920
rs143745495
All
GCST90277351-PCOS
−0.4
−0.3
−0.2
−0.1
0.0
MR leave−one−out sensitivity analysis for
'exposure' on 'outcome'

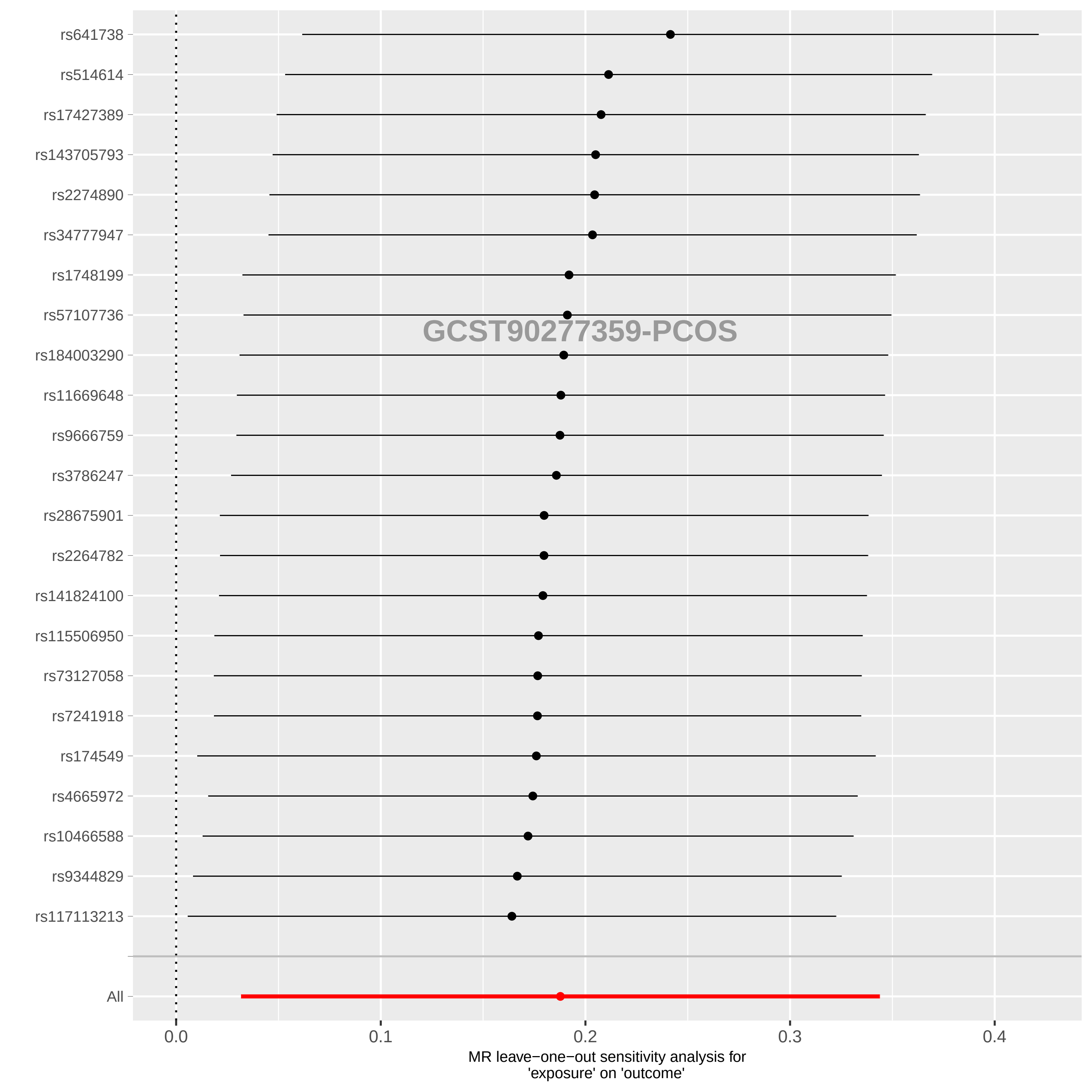

rs641738
rs514614
rs17427389
rs143705793
rs2274890
rs34777947
rs1748199
rs57107736
rs184003290
rs11669648
rs9666759
rs3786247
rs28675901
rs2264782
rs141824100
rs115506950
rs73127058
rs7241918
rs174549
rs4665972
rs10466588
rs9344829
rs117113213
All
GCST90277359-PCOS
0.0
0.1
0.2
0.3
0.4
MR leave−one−out sensitivity analysis for
'exposure' on 'outcome'

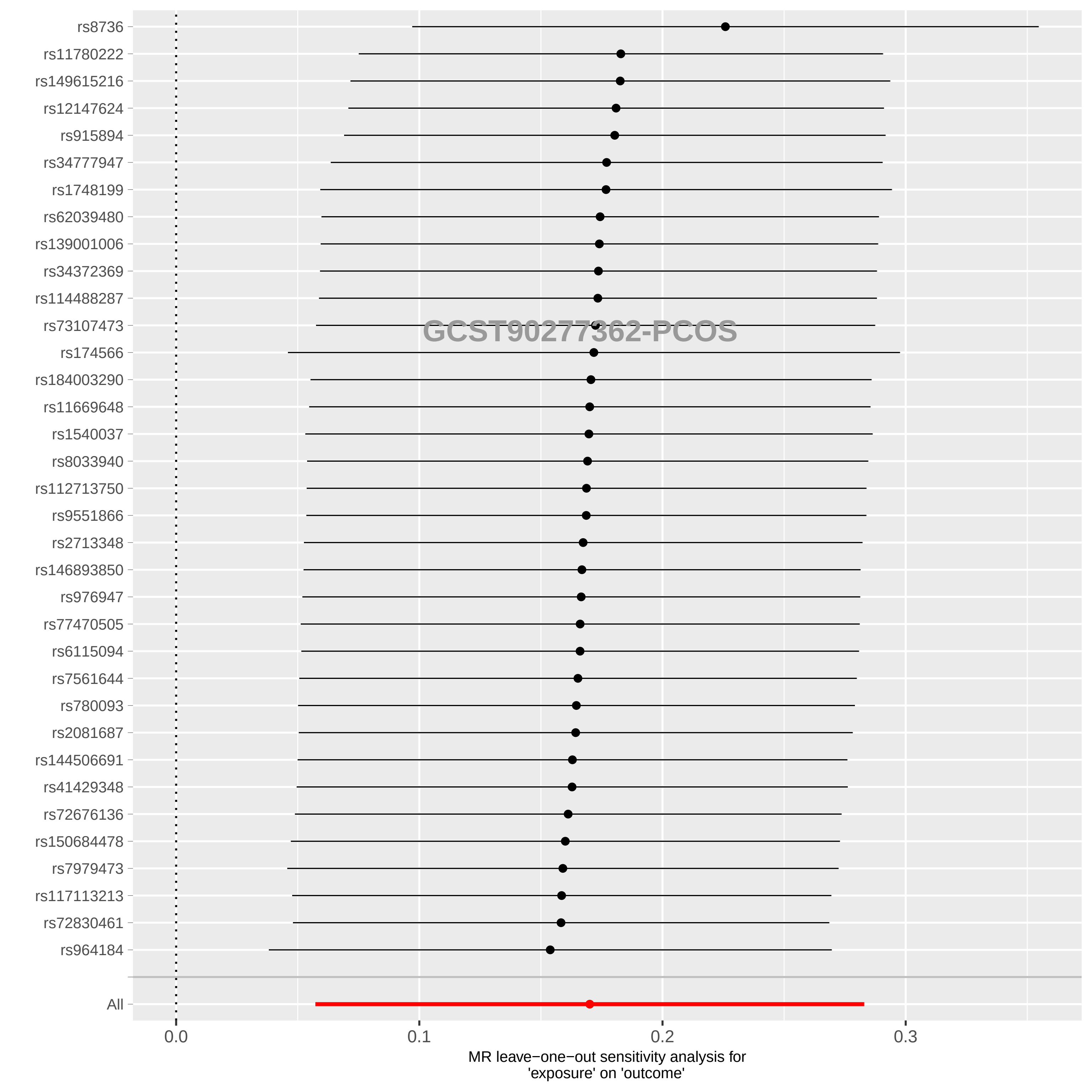
rs8736
rs11780222
rs149615216
rs12147624
rs915894
rs34777947
rs1748199
rs62039480
rs139001006
rs34372369
rs114488287
rs73107473
rs174566
rs184003290
rs11669648
rs1540037
rs8033940
rs112713750
rs9551866
rs2713348
rs146893850
rs976947
rs77470505
rs6115094
rs7561644
rs780093
rs2081687
rs144506691
rs41429348
rs72676136
rs150684478
rs7979473
rs117113213
rs72830461
rs964184
All
GCST90277362-PCOS
0.0
0.1
0.2
0.3
MR leave−one−out sensitivity analysis for
'exposure' on 'outcome'

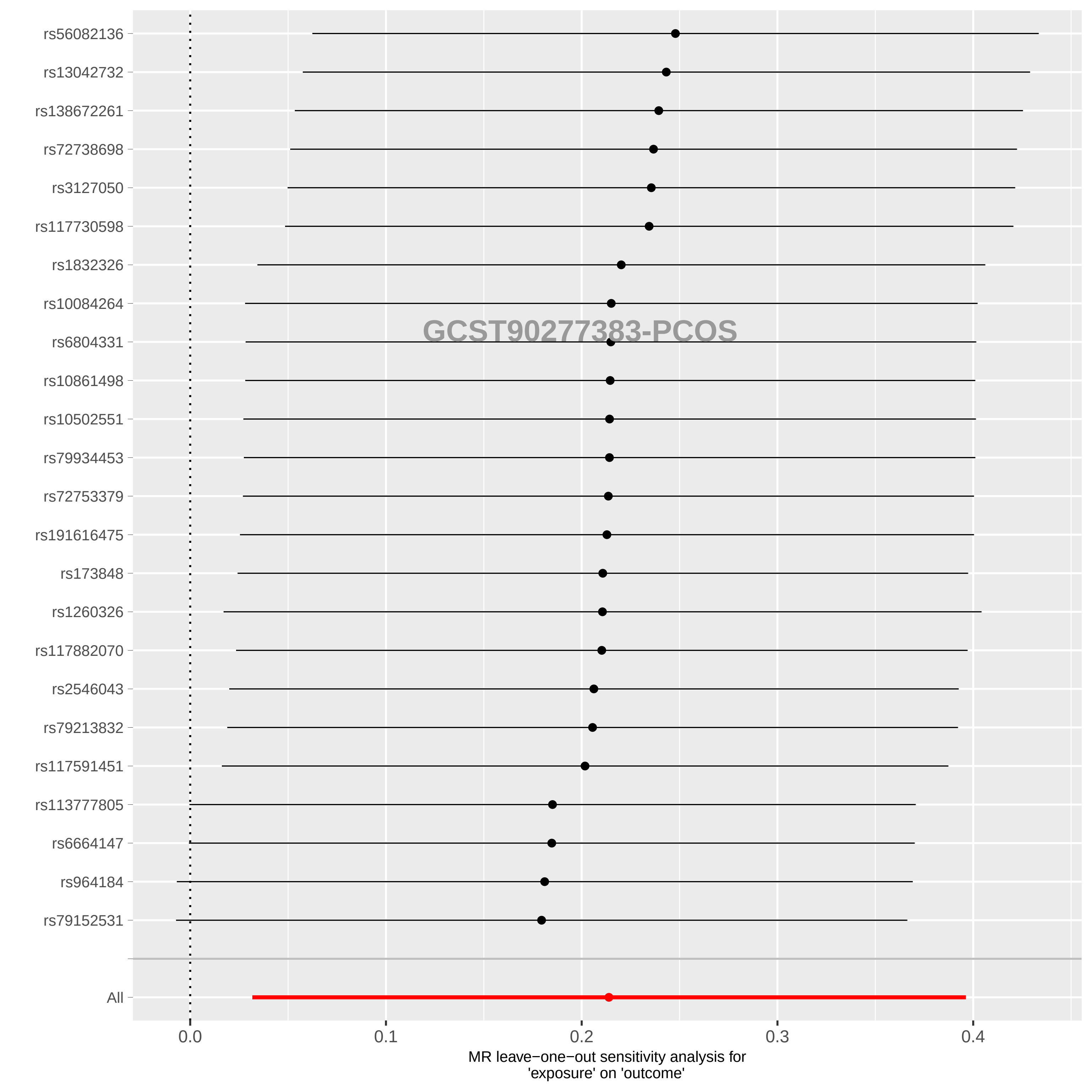

rs56082136
rs13042732
rs138672261
rs72738698
rs3127050
rs117730598
rs1832326
rs10084264
rs6804331
rs10861498
rs10502551
rs79934453
rs72753379
rs191616475
rs173848
rs1260326
rs117882070
rs2546043
rs79213832
rs117591451
rs113777805
rs6664147
rs964184
rs79152531
All
GCST90277383-PCOS
0.0
0.1
0.2
0.3
0.4
MR leave−one−out sensitivity analysis for
'exposure' on 'outcome'

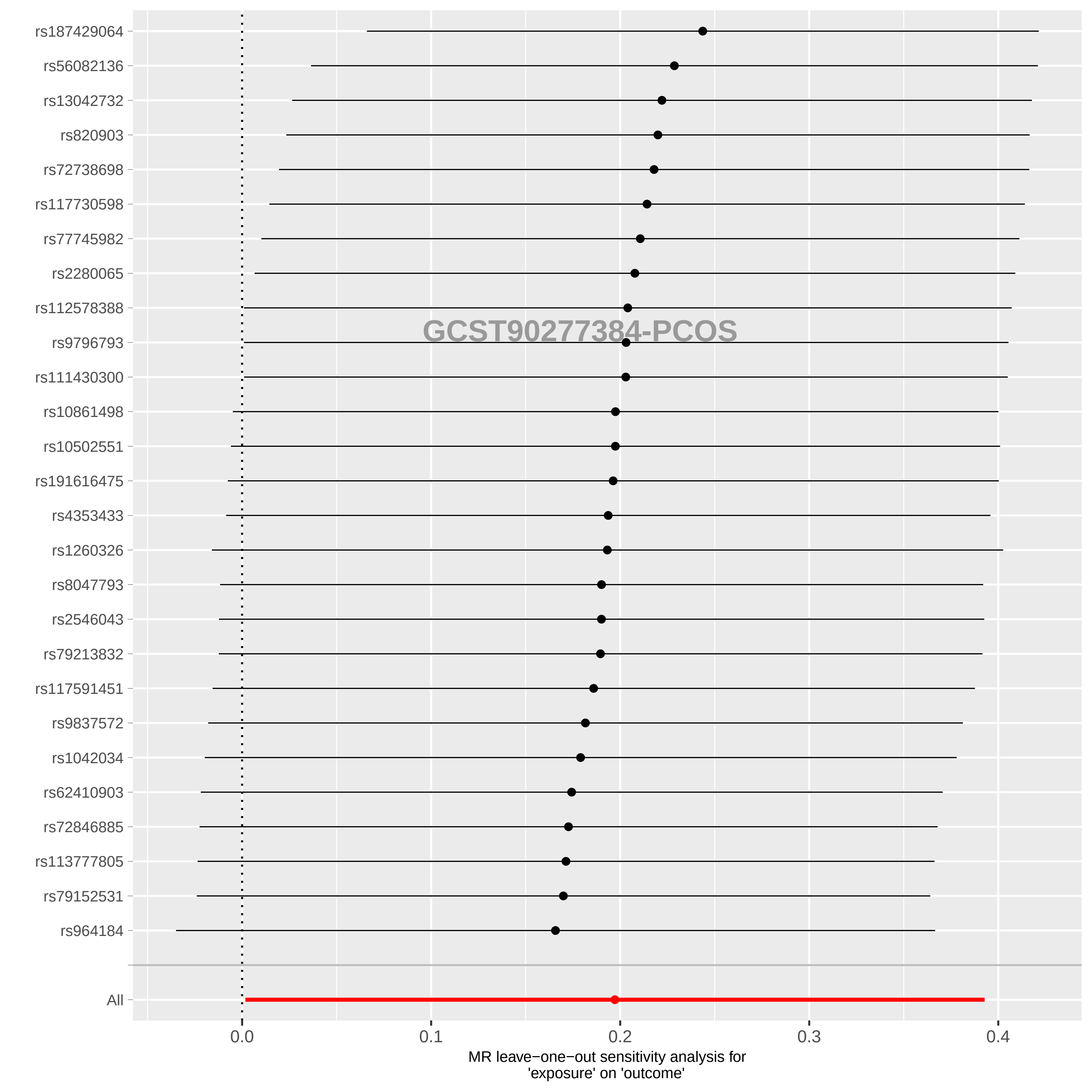

rs187429064
rs56082136
rs13042732
rs820903
rs72738698
rs117730598
rs77745982
rs2280065
rs112578388
rs9796793
rs111430300
rs10861498
rs10502551
rs191616475
rs4353433
rs1260326
rs8047793
rs2546043
rs79213832
rs117591451
rs9837572
rs1042034
rs62410903
rs72846885
rs113777805
rs79152531
rs964184
All
GCST90277384-PCOS
0.0
0.1
0.2
0.3
0.4
MR leave−one−out sensitivity analysis for
'exposure' on 'outcome'

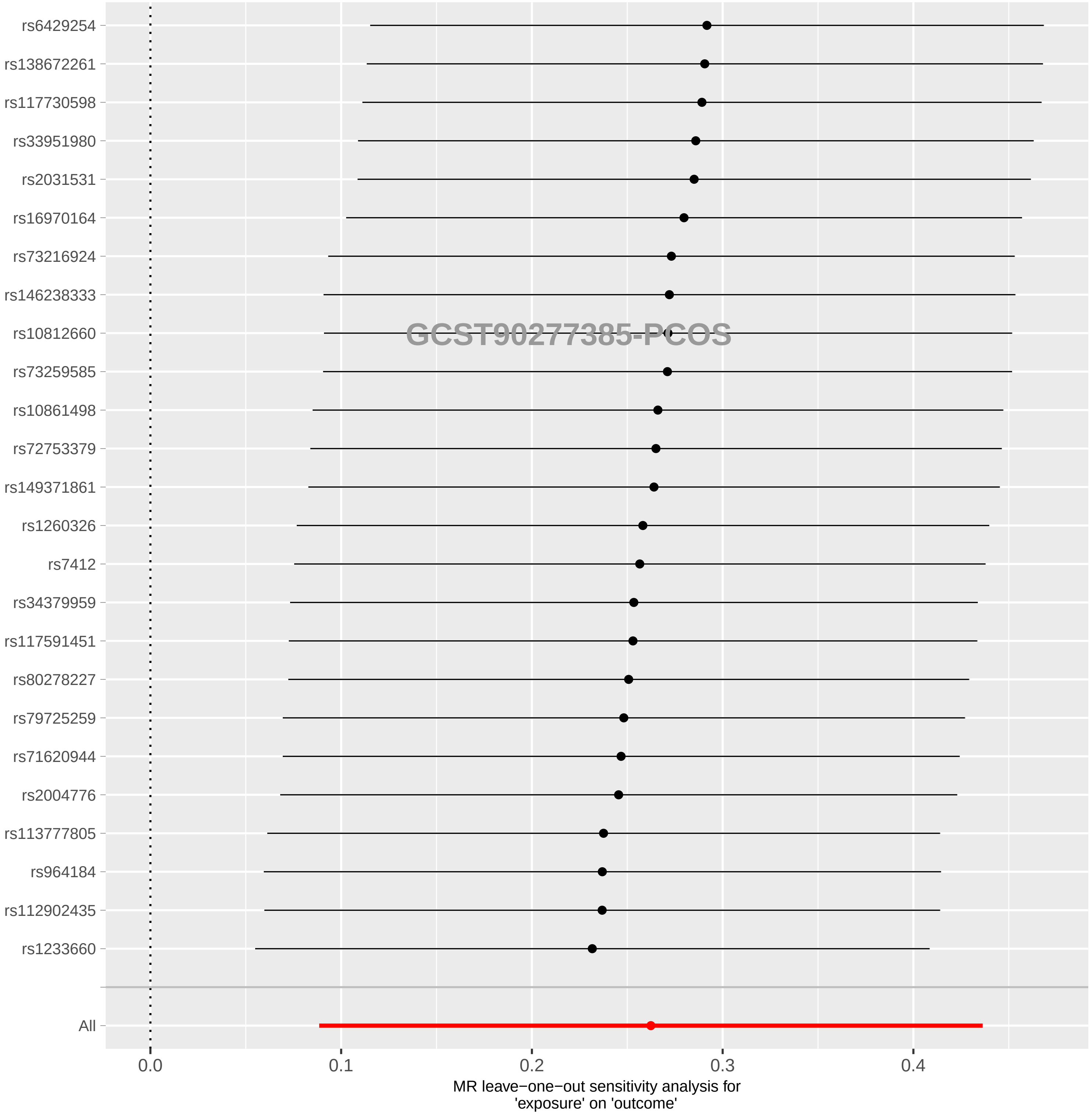
GCST90277385-PCOS
rs6429254
rs138672261
rs117730598
rs33951980
rs2031531
rs16970164
rs73216924
rs146238333
rs10812660
rs73259585
rs10861498
rs72753379
rs149371861
rs1260326
rs7412
rs34379959
rs117591451
rs80278227
rs79725259
rs71620944
rs2004776
rs113777805
rs964184
rs112902435
rs1233660
All
0.0
0.1
0.2
0.3
0.4
MR leave−one−out sensitivity analysis for
'exposure' on 'outcome'

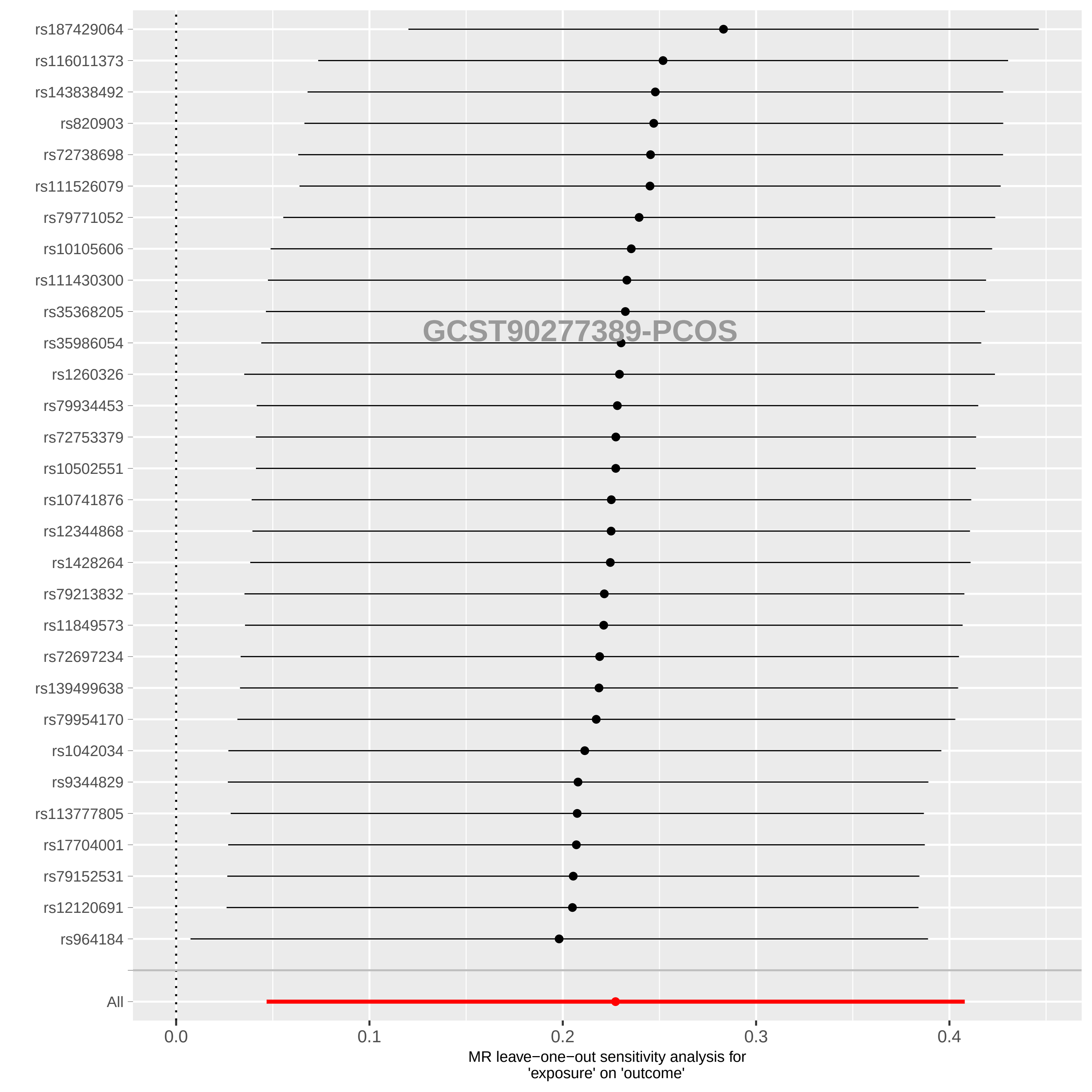
GCST90277389-PCOS
rs187429064
rs116011373
rs143838492
rs820903
rs72738698
rs111526079
rs79771052
rs10105606
rs111430300
rs35368205
rs35986054
rs1260326
rs79934453
rs72753379
rs10502551
rs10741876
rs12344868
rs1428264
rs79213832
rs11849573
rs72697234
rs139499638
rs79954170
rs1042034
rs9344829
rs113777805
rs17704001
rs79152531
rs12120691
rs964184
All
0.0
0.1
0.2
0.3
0.4
MR leave−one−out sensitivity analysis for
'exposure' on 'outcome'

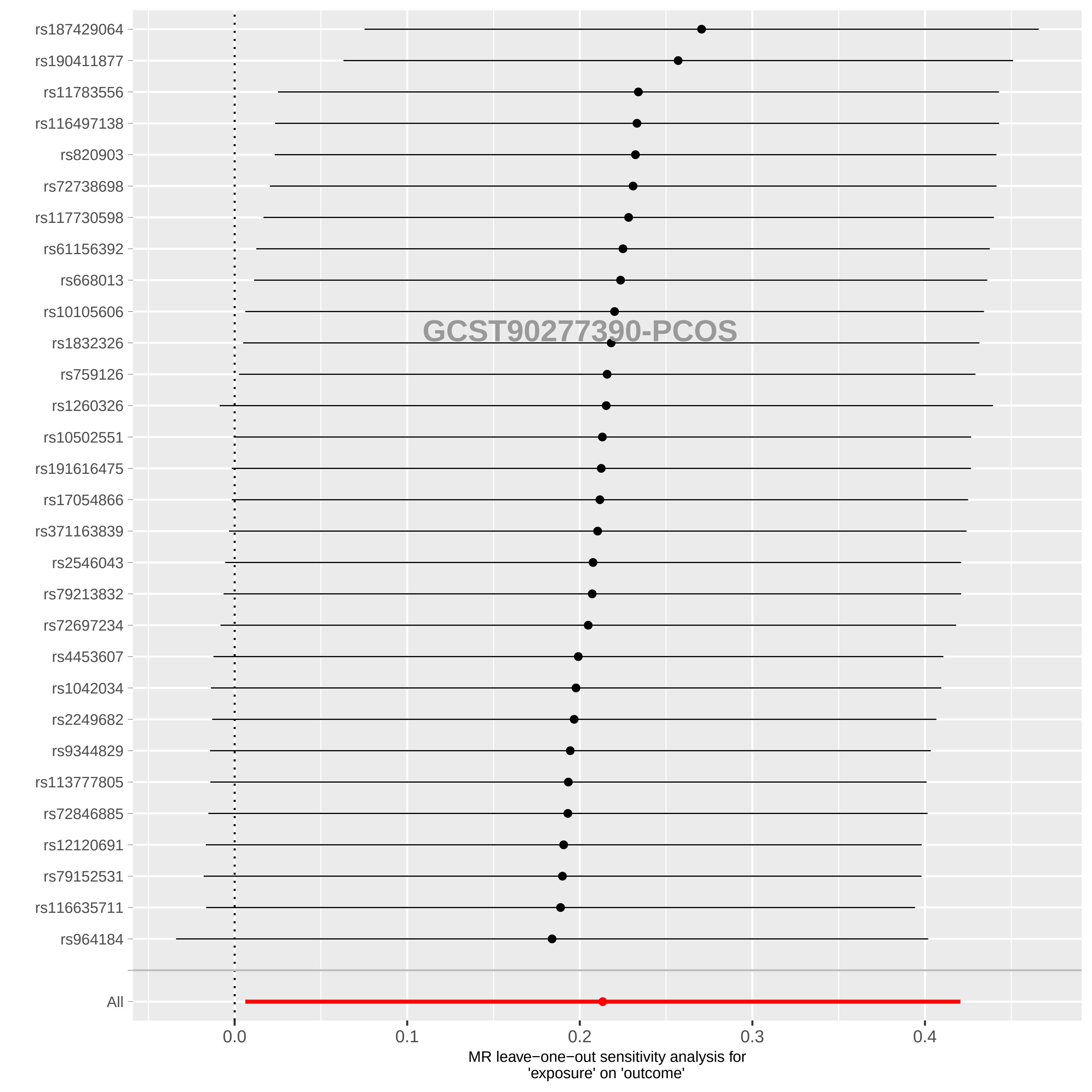
rs187429064
rs190411877
rs11783556
rs116497138
rs820903
rs72738698
rs117730598
rs61156392
rs668013
rs10105606
rs1832326
rs759126
rs1260326
rs10502551
rs191616475
rs17054866
rs371163839
rs2546043
rs79213832
rs72697234
rs4453607
rs1042034
rs2249682
rs9344829
rs113777805
rs72846885
rs12120691
rs79152531
rs116635711
rs964184
All
GCST90277390-PCOS
0.0
0.1
0.2
0.3
0.4
MR leave−one−out sensitivity analysis for
'exposure' on 'outcome'
